# Supplementary material for: The Derlin-1-Stat5b axis maintains homeostasis of adult hippocampal neurogenesis
Source: EMBO Rep. 2024 Jul 30;25(8):26. doi: 10.1038/s44319-024-00205-7 (PMC11316036; doi:10.1038/s44319-024-00205-7)
Supplement: Supplementary file 1 — Appendix [file 44319_2024_205_MOESM1_ESM.pdf]

Appendix

The Derlin-1-Stat5b Axis Maintains Homeostasis of Adult Hippocampal Neurogenesis

Naoya Murao[1], Taito Matsuda[2], Hisae Kadowaki[1], Yosuke Matsushita[3,4], Kousuke Tanimoto[5], Toyomasa Katagiri[3,4], Kinichi Nakashima[2,*], and Hideki Nishitoh[1,6,7,*]

[1] Laboratory of Biochemistry and Molecular Biology, Department of Medical Sciences, University of Miyazaki, Miyazaki, Japan
[2] Department of Stem Cell Biology and Medicine, Graduate School of Medical Sciences, Kyushu University, Fukuoka, Japan
[3] Division of Genome Medicine, Tokushima University, Tokushima, Japan
[4] National Institutes of Biomedical Innovation, Health and Nutrition, Osaka, Japan
[5] High-risk Infectious Disease Control, Graduate School of Medical and Dental Sciences, Tokyo Medical and Dental University, Tokyo, Japan
[6] Frontier Science Research Center, University of Miyazaki, Miyazaki, Japan
[7] Lead contact

*Correspondence: Kinichi Nakashima and Hideki Nishitoh

Email: nakashima.kinichi.718@m.kyushu-u.ac.jp (K.N.), nishitoh@med.miyazaki-u.ac.jp (H.N.)

Table of Contents:

1. Appendix Table S1 .... pages 2–6
2. Appendix Table S2 .... page 7
3. Appendix Table S3 .... pages 8–9
4. References .... page 10

Appendix Table S1. List of genes with altered expression in siDerl1 NSCs.

Gene list whose expression level is >1.5-fold increased in siDerl1 NSCs

Refseq ID	GeneSymbol	Description	Fold change	P value
NM_053789	Il17b	interleukin 17B	28.72369561	0.003845464
NM_017154	Xdh	xanthine dehydrogenase	28.01189405	2.90E-05
NM_138854	Slc38a5	solute carrier family 38, member 5	27.30578913	0.016246065
NM_001106277	Saxo2	stabilizer of axonemal microtubules 2	23.00916878	0.012429043
NM_001105749	Il16	interleukin 16	19.66806675	0.033194297
NM_001011971	Xkr4	XK related 4	18.38027887	0.03431546
NM_020101	Adap2	ArfGAP with dual PH domains 2	18.18781236	0.026796782
NM_001271361	St6galnac5	ST6 N-acetylgalactosaminide alpha-2,6-sialyltransferase 5	18.14166765	0.046077641
NM_001106906	Gpr45	G protein-coupled receptor 45	17.83467498	0.046662042
NM_001107021	Hic1	HIC ZBTB transcriptional repressor 1	17.64655338	0.0399173
NM_001305243	Eda	ectodysplasin-A	17.4413855	0.044453758
NM_001108279	Bcl6b	BCL6B, transcription repressor	13.01440305	0.000679595
NM_130741	Lcn2	lipocalin 2	12.265538	2.40E-05
NM_024354	Chrna4	cholinergic receptor nicotinic alpha 4 subunit	11.6910145	0.003002521
NM_001007011	Zpbp2	zona pellucida binding protein 2	11.25355364	0.002292234
NM_001108979	Atp6v1e2	ATPase H+ transporting V1 subunit E2	9.452958238	0.009062017
NM_016994	C3	complement C3	9.355076006	0.000269583
NM_001271398	Kirrel2	kirre like nephrin family adhesion molecule 2	9.002633066	0.006733583
NM_001013084	Akr1b10	aldo-keto reductase family 1 member B10	8.853624237	0.010955202
NM_001044304	Eid3	EP300 interacting inhibitor of differentiation 3	8.502484989	0.001164639
NM_207607	Ns5atp4		8.199592209	0.029474077
NM_194353	Setl1		8.0750904	0.028780778
NM_001033957,N	Kcng3	potassium voltage-gated channel modifier subfamily G member 3	8.062650312	0.018451264
NM_031055	Mmp9	matrix metallopeptidase 9	7.4276006	1.55E-06
NM_001109536	Ptx3	pentraxin 3	7.249857943	0.032714643
NM_013107	Bmp6	bone morphogenetic protein 6	7.167416648	0.000135371
NM_001105739	Prrx2	paired related homeobox 2	6.075331872	0.013136782
NM_001108550	Postn	periostin	6.012517256	0.005051686
NM_001007612	Ccl7	C-C motif chemokine ligand 7	5.660284185	0.005588881
NM_001104527	Prr15		5.651010713	0.004505096
NM_001109996,N	Apoc1	apolipoprotein C1	5.634992027	0.020834602
NM_020305	Adam4	a disintegrin and metalloprotease domain 4	5.245130505	0.001996655
NM_022931	Rims3	regulating synaptic membrane exocytosis 3	4.824446032	0.02447858
NM_001106664	Tyrp1	tyrosinase-related protein 1	4.823241381	0.005019777
NM_139216,NM_1	Kcnc2	potassium voltage-gated channel subfamily C member 2	4.649878817	0.006359266
NM_001108161	Cilp	cartilage intermediate layer protein	4.609237491	0.000349139
NM_030845	Cxcl1	C-X-C motif chemokine ligand 1	4.450594639	0.001632821
NM_001191861	Cdk6	cyclin-dependent kinase 6	4.304971631	0.018957314
NM_153722	Mrgprf	MAS related GPR family member F	4.274727183	0.011359421
NM_031530	Ccl2	C-C motif chemokine ligand 2	4.189269844	0.000561563
NM_207613	Cdh15	cadherin 15	4.084365822	0.000908681
NM_001107763	Ltk	leukocyte receptor tyrosine kinase	4.018737034	0.023537516
NM_021589	Ntrk1	neurotrophic receptor tyrosine kinase 1	3.855032183	0.024274736
NM_001109419	Apoc4	apolipoprotein C4	3.813419178	0.005206018
NM_001191822	Rhbdl1	rhomboid like 1	3.498096885	0.006871344
NM_172047	Eaf2	ELL associated factor 2	3.470704647	0.013662039
NM_001134562	Rasal3	RAS protein activator like 3	3.462527467	0.01411219
NM_001107572	Ovol1	ovo like transcriptional repressor 1	3.372839189	0.013790267
NM_001024360	Lrrc73		3.294792389	0.011273135
NM_022234	Asic4	acid sensing ion channel subunit family member 4	3.289974713	0.048996677
NM_017193	Aadat	aminoadipate aminotransferase	3.254592587	0.032884732
NM_001002804	C1rl	complement C1r subcomponent like	3.198154658	0.031540286
NM_001301812,N	Crhr1	corticotropin releasing hormone receptor 1	3.173932237	0.02947819
NM_031525	Pdgfrb	platelet derived growth factor receptor beta	3.155538577	9.50E-06
NM_001109049	Ccdc103	coiled-coil domain containing 103	3.136847761	0.044093415
NM_133298	Gpnmb	glycoprotein nmb	3.10844331	7.89E-05
NM_001127540	Lmntd2	lamin tail domain containing 2	3.050451149	0.003044703
NM_017250	Htr2b	5-hydroxytryptamine receptor 2B	2.983068279	0.015934826
NM_031056	Mmp14	matrix metallopeptidase 14	2.950376309	0.000231049
NM_001108469	Cdc42ep5	CDC42 effector protein 5	2.92404232	0.005596293
NM_001025772	Stpg1	sperm-tail PG-rich repeat containing 1	2.918903105	0.007027428
NM_001277264,N	Apoa5	apolipoprotein A5	2.748737726	0.015127821
NM_053356,NM_0	Col1a2	collagen type I alpha 2 chain	2.748650951	0.030197987
NM_030868	Nov	cellular communication network factor 3	2.736157254	0.002250091
NM_001024342	Dnai1	dynein, axonemal, intermediate chain 1	2.735957272	0.002344052
NM_019370	Enpp3	ectonucleotide pyrophosphatase/phosphodiesterase 3	2.670984226	0.024219051
NR_024118	Tnxa-ps1		2.661907108	0.031623118
NM_001107623	Tmem26	transmembrane protein 26	2.650181119	0.015224534
NM_001137622	Adamts2	ADAM metallopeptidase with thrombospondin type 1 motif, 2	2.649746367	0.03013333
NM_001014043	Sgms2	sphingomyelin synthase 2	2.642897195	0.025468204
NM_001017514	Izumo1	izumo sperm-egg fusion 1	2.58878294	0.004952221
NM_001108816	Piga	phosphatidylinositol glycan anchor biosynthesis, class A	2.551822261	0.003771704
NM_001106777	RGD1561102	similar to ribosomal protein S12	2.527781034	0.039001418
NM_001014041	Fam46c	terminal nucleotidyltransferase 5C	2.509155365	0.013181522

NM_001135600	Cyp4v3	cytochrome P450, family 4, subfamily v, polypeptide 3	2.488351317	0.006319003
NM_001013171	Gulp1	GULP PTB domain containing engulfment adaptor 1	2.480867472	0.030827376
NM_001002805	C4b	complement C4B (Chido blood group)	2.477015965	0.004774573
NM_001013102	Theg	theg spermatid protein	2.430575423	0.002380573
NM_001127541,N	Cracr2b	calcium release activated channel regulator 2B	2.430109739	0.04603197
NM_001013137	Cxcl14	C-X-C motif chemokine ligand 14	2.417848843	0.000194173
NM_001106702	Angptl6	angiopoietin-like 6	2.382867386	0.005397008
NM_001107182	Crb1	crumbs cell polarity complex component 1	2.380761092	0.007339484
NM_001011984	Asb2	ankyrin repeat and SOCS box-containing 2	2.34818169	0.007910021
NM_053338	Rrad	RRAD, Ras related glycolysis inhibitor and calcium channel regulator	2.345985376	0.020271895
NM_031091	Rab3b	RAB3B, member RAS oncogene family	2.321269667	0.00021119
NM_133559	Pcsk4	proprotein convertase subtilisin/kexin type 4	2.316766164	0.003306001
NM_001013160	Trim69	tripartite motif-containing 69	2.30811084	0.015223422
NM_001271115	Dnaaf3	dynein, axonemal, assembly factor 3	2.276079767	0.017373821
NM_053955	Crym	crystallin, mu	2.271364757	0.007842237
NM_022675	Fkbp1b	FKBP prolyl isomerase 1B	2.249641496	0.03546005
NM_001108569	Gbp5	guanylate binding protein 5	2.199890284	0.03821303
NM_012851	Hsd17b1	hydroxysteroid (17-beta) dehydrogenase 1	2.198366294	0.034091885
NM_053442	Slc7a8	solute carrier family 7 member 8	2.181775538	0.001543119
NM_001134736	Wdr63	WD repeat domain 63	2.181497882	0.017739466
NM_001106231	Ppm1n	protein phosphatase, Mg2+/Mn2+ dependent 1N	2.166303157	0.010587944
NM_001025044	Ccdc146	coiled-coil domain containing 146	2.149838594	0.010747091
NM_001014068	Hpdl	4-hydroxyphenylpyruvate dioxygenase-like	2.119886636	0.034994583
NM_001107246	Mfsd7	solute carrier family 49 member 3	2.111358152	0.015000457
NM_013180	Itgb4	integrin subunit beta 4	2.09286365	0.007606486
NM_001013243	Slc30a3	solute carrier family 30 member 3	2.054755316	0.030171361
NM_001012059	Mcoln3	mucolipin 3	2.053841022	0.007810665
NM_001038615	Fndc1	fibronectin type III domain containing 1	2.034485922	0.04126362
NM_001008880	Scn4b	sodium voltage-gated channel beta subunit 4	2.010282603	0.035700654
NM_001100741	Col6a2	collagen type VI alpha 2 chain	2.003400756	0.000663071
NM_001134695	Fam229b		2.002878271	0.001449445
NM_175592	Cacna2d2	calcium voltage-gated channel auxiliary subunit alpha2delta 2	1.996318288	0.031750248
NM_053870	Kcnj4	potassium inwardly-rectifying channel, subfamily J, member 4	1.991211741	0.036379663
NM_001109226	Prrt4	proline-rich transmembrane protein 4	1.985564856	0.029506408
NM_001271179	Slc25a45	solute carrier family 25, member 45	1.985034297	0.027917631
NM_001105800	Zmynd15	zinc finger, MYND-type containing 15	1.973386679	0.041187248
NM_001109585	Trim47	tripartite motif-containing 47	1.968640757	0.044714619
NM_012912	Atf3	activating transcription factor 3	1.960600604	0.038414275
NM_057201	Gpr37	G protein-coupled receptor 37	1.948128409	0.035046166
NM_021680	Nxph4	neurexophilin 4	1.944355321	0.023273056
NM_001010965	Mok	MOK protein kinase	1.938018071	0.034434393
NM_134432	Agt	angiotensinogen	1.931336455	8.33E-05
NM_001108226	Wnt6	Wnt family member 6	1.917570778	0.00254144
NM_181380	Rtn4rl2	reticulon 4 receptor-like 2	1.909858343	0.006000854
NM_053500	Slc25a27	solute carrier family 25, member 27	1.905523196	0.030927106
NM_001106348	Mlana	melan-A	1.885160314	0.001502623
NM_001108227	Wnt10a	Wnt family member 10A	1.884199995	0.030626645
NM_019386	Tgm2	transglutaminase 2	1.877021487	0.043717816
NM_001008724,N	Fga	fibrinogen alpha chain	1.869408476	0.039353688
NM_001172103	Pim2		1.863151801	0.005622438
NM_001271272	Ndufa4l2	NDUFA4, mitochondrial complex associated like 2	1.862467872	0.034804663
NM_001109655	Car14	carbonic anhydrase 14	1.85928131	0.027378896
NM_001107767	Duoxa1	dual oxidase maturation factor 1	1.85857763	0.021093079
NM_001107857	Ephb6	Eph receptor B6	1.850186624	0.010932276
NM_012676	Tnnt2	troponin T2, cardiac type	1.848115508	0.041574584
NR_130129	LOC104940696		1.844136133	0.00428513
NM_001172079	Icam5	intercellular adhesion molecule 5	1.841237579	0.004512566
NM_001109227	Tspan33	tetraspanin 33	1.833629547	0.004641388
NM_001014051	Ttll9	tubulin tyrosine ligase like 9	1.829841639	0.014392166
NM_001013072	Sfxn2	sideroflexin 2	1.817197797	0.018140056
NM_001077680	Bpifb1	BPI fold containing family B, member 1	1.812205628	0.006398882
NM_199085	Serpinb6	serpin family B member 6A	1.805367245	0.021569098
NM_013166	Cntf	ciliary neurotrophic factor	1.792471147	0.008812104
NM_031504	C4a	complement C4A	1.784968478	0.000609862
NM_001001514,N	Ablim2	actin binding LIM protein family, member 2	1.775598327	0.004350853
NM_019363	Aox1	aldehyde oxidase 1	1.762008497	0.001723391
NM_001271346	Ddb2	damage specific DNA binding protein 2	1.745584361	0.009380517
NM_001308302,N	Cacna1g	calcium voltage-gated channel subunit alpha1 G	1.744346994	0.000546722
NM_001128152	Rwdd3	RWD domain containing 3	1.743366983	0.014968507
NM_001109574	Tmem169	transmembrane protein 169	1.740324097	0.007131359
NM_013015	Ptgds	prostaglandin D2 synthase	1.731518979	0.048804518
NM_080688	Plcd4	phospholipase C, delta 4	1.728447396	0.018280326
NM_001270681,N	Apoe	apolipoprotein E	1.721278189	0.000340202
NM_001107724	Tram1l1	translocation associated membrane protein 1-like 1	1.719045671	0.026881683
NM_012868	Npr3	natriuretic peptide receptor 3	1.71065961	0.001402506
NM_031548	Scnn1a	sodium channel epithelial 1 subunit alpha	1.707042679	0.03063938
NM_001270855,N	Stmn4	stathmin 4	1.701357133	0.028656736
NM_001134845	LOC688613	hypothetical protein LOC688613	1.695775326	0.01551456

NM_001276304,N	Dlgap3	DLG associated protein 3	1.69455377	0.009316655
NM_181636	Col23a1	collagen type XXIII alpha 1 chain	1.690521862	0.019923778
NM_031597	Kcnq3	potassium voltage-gated channel subfamily Q member 3	1.683511045	0.035645631
NM_012650	Shbg	sex hormone binding globulin	1.677562282	0.043817765
NM_001108978	Pik3cd	phosphatidylinositol-4,5-bisphosphate 3-kinase, catalytic subunit delta	1.671466662	0.032534986
NM_001109160	Flrt1	fibronectin leucine rich transmembrane protein 1	1.664846035	0.012806989
NM_001106713	Klhl29		1.662999796	0.023035955
NM_001106877	Rfx2	regulatory factor X2	1.661784625	0.019386213
NM_001107248	Vcl	vinculin	1.64233194	0.003558683
NM_017014	Gstm1	glutathione S-transferase mu 1	1.641508853	0.006104227
NM_001025048	Setmar	SET domain and mariner transposase fusion gene	1.640641275	0.04200108
NM_001009709	Tmem140	transmembrane protein 140	1.635276991	0.023632814
NM_053304	Col1a1	collagen type I alpha 1 chain	1.619038929	0.024643617
NM_001191778	Aldh1l2	aldehyde dehydrogenase 1 family, member L2	1.611367111	0.029039592
NM_130411	Coro1a	coronin 1A	1.607898287	0.027062065
NM_031154	Gstm7	glutathione S-transferase, mu 7	1.590868524	0.003336554
NM_001108065	Shc2	SHC adaptor protein 2	1.577211924	0.041894813
NM_001160162,N	Scn5a	sodium voltage-gated channel alpha subunit 5	1.574973314	0.011586839
NM_019161	Cdh22	cadherin 22	1.568801176	0.025028534
NM_001106276	Cpeb1	cytoplasmic polyadenylation element binding protein 1	1.565659685	0.002065137
NM_133606	Ehhadh	enoyl-CoA hydratase and 3-hydroxyacyl CoA dehydrogenase	1.564130814	0.03642322
NM_023981	Csf1	colony stimulating factor 1	1.56389721	0.002249108
NM_001011976	Wdr31		1.558293089	0.027430515
NM_017009	Gfap	glial fibrillary acidic protein	1.550004168	0.004017337
NM_001030042	Rad9b	RAD9 checkpoint clamp component B	1.541249498	0.019168947
NM_001109183	Lhfp	LHFPL tetraspan subfamily member 6	1.533454143	0.002255867
NM_138858	Slc9a5	solute carrier family 9 member A5	1.524382113	0.033650301
NM_001108098	Cpm	carboxypeptidase M	1.517368103	0.008216459
NM_001017478	Cxcl16	C-X-C motif chemokine ligand 16	1.51671187	0.012903395
NM_080782	Cdkn1a	cyclin-dependent kinase inhibitor 1A	1.512067924	0.029627177
NM_001130499	Ttc38		1.510134741	0.036211686
NM_001108987	Diras1	DIRAS family GTPase 1	1.509655659	0.042750169

Gene list whose expression level is <0.8-fold reduced in siDerl1 NSCs

Refseq ID	GeneSymbol	Description	Fold change	P value
NM_001017510	LOC498750		0.011912108	0.007776697
NM_031560	Ctsk	cathepsin K	0.022471789	0.000146434
NM_012841	Dcc	DCC netrin 1 receptor	0.026872545	0.002358742
NM_001107671	Plcxd3	phosphatidylinositol-specific phospholipase C, X domain containing 3	0.032147715	0.002587457
NM_001108996	Ap1m2	adaptor related protein complex 1 subunit mu 2	0.033645063	0.014430631
NM_001024907	MGC114499		0.044566363	0.029718305
NM_001191843	Atp6ap1l	ATPase H+ transporting accessory protein 1 like	0.046196993	0.012600929
NM_001105737	Tek	TEK receptor tyrosine kinase	0.047187309	0.01270479
NM_133420	Chrna2	cholinergic receptor nicotinic alpha 2 subunit	0.051363696	0.04169532
NM_022636	Vax1	ventral anterior homeobox 1	0.052576863	0.026075399
NM_138897	Gabrr3	gamma-aminobutyric acid type A receptor rho3 subunit	0.053784985	0.026759072
NM_001191728	Lancl3	LanC like 3	0.054034641	0.046316639
NM_053423	Tert	telomerase reverse transcriptase	0.060818771	0.046825904
NM_001109130	Tsnaxip1	translin-associated factor X interacting protein 1	0.061169113	0.039675912
NM_053934	Pcdha13	protocadherin alpha 13	0.061314119	0.047254662
NM_001107056	Cyb561	cytochrome b-561	0.061762371	0.04386896
NM_001109641	Hist3h2bb	H2B.U histone 1	0.062856005	0.044362396
NM_031796	Galnt5	polypeptide N-acetylgalactosaminyltransferase 5	0.072997826	0.00677792
NM_031828	Kcnma1	potassium calcium-activated channel subfamily M alpha 1	0.094163072	0.000210535
NM_053744	Dlk1	delta like non-canonical Notch ligand 1	0.103349815	0.003838884
NM_001108051	Slc24a4	solute carrier family 24 member 4	0.109592958	0.000174471
NM_012636	Pthlh	parathyroid hormone-like hormone	0.116185855	0.000506216
NM_001100523	Otp	orthopedia homeobox	0.12013391	0.007099
NM_001111114,N	Grik1	glutamate ionotropic receptor kainate type subunit 1	0.120892009	0.031771457
NM_001107755	Pamr1	peptidase domain containing associated with muscle regeneration 1	0.128402805	0.011777393
NM_013120	Gckr	glucokinase regulator	0.129700017	0.01277519
NM_022407	Aldh1a1	aldehyde dehydrogenase 1 family, member A1	0.139129865	0.001992054
NM_001305138,N	Synpo2l	synaptopodin 2-like	0.144837167	0.041216021
NM_053318	Hpx	hemopexin	0.159402072	0.045434139
NM_031741	Slc2a5	solute carrier family 2 member 5	0.17238264	0.03198011
NM_023100	Nmur1	neuromedin U receptor 1	0.177634398	0.011291368
NM_019190	Cd46	CD46 molecule	0.185976367	0.027572512
NM_031012	Anpep	alanyl aminopeptidase, membrane	0.207542493	0.021753365
NM_138530	Pbld1	phenazine biosynthesis-like protein domain containing 1	0.22379207	0.004746682
NM_001127650	Anks4b	ankyrin repeat and sterile alpha motif domain containing 4B	0.225229537	0.043559439
NM_053977	Cdh17	cadherin 17	0.230005421	0.024744727
NM_198748	Scin	scinderin	0.240124233	0.028676846
NM_001108568	Dapp1	dual adaptor of phosphotyrosine and 3-phosphoinositides 1	0.240432876	0.003788887
NM_031117	Snrpn	small nuclear ribonucleoprotein polypeptide N	0.250782252	0.031408865
NM_001130502	Fam83f		0.251396067	0.003843109

NM_031549	Tagln	transgelin	0.268809702	0.003157927
NM_001033687	Ushbp1	USH1 protein network component harmonin binding protein 1	0.27018035	0.015159021
NM_053441	Slco1c1	solute carrier organic anion transporter family, member 1c1	0.270762242	0.042160854
NM_001109309	Cdk5r2	cyclin-dependent kinase 5 regulatory subunit 2	0.273806725	0.013966022
NM_001106267	Tm2d3	TM2 domain containing 3	0.288605669	0.015286944
NM_001077677	Pacrg	parkin coregulated	0.30723147	0.000451762
NM_001127557	Rtdr1		0.314455776	0.010457361
NM_032070	Hmga2	high mobility group AT-hook 2	0.334480841	0.000995459
NM_001076553,N	Pkib	cAMP-dependent protein kinase inhibitor beta	0.350935159	0.001112807
NM_001008838	RT1-CE15		0.36549479	0.031009856
NM_001047914	Sdhaf3	succinate dehydrogenase complex assembly factor 3	0.372738155	0.01523939
NM_001191694	Nebl	nebulette	0.373426558	0.008008049
NM_031347	Ppargc1a	PPARG coactivator 1 alpha	0.377660729	0.01916138
NM_133285	Hist1h1d	H1.4 linker histone, cluster member	0.382868558	0.008112707
NM_001127602	Slc25a53		0.383742202	0.000379422
NM_001108859	Irf6	interferon regulatory factor 6	0.400224974	0.00349448
NM_019265	Scn11a	sodium voltage-gated channel alpha subunit 11	0.400917748	0.005851973
NM_001009695	Wnt7b	Wnt family member 7B	0.403075487	0.031225081
NM_001127640	PCOLCE2	procollagen C-endopeptidase enhancer 2	0.406032106	0.000170092
NM_138524	A3galt2	alpha 1,3-galactosyltransferase 2	0.418470472	0.000934804
NM_001024282	Hist1h2af	histone cluster 1 H2A family member F	0.42040864	0.044597569
NM_173136	Akr1b8	aldo-keto reductase family 1, member B8	0.421725801	0.005579554
NM_001012460	Septin1	septin 1	0.422672974	0.030943282
NM_001107170	Tfcp2l1	transcription factor CP2-like 1	0.431626543	0.012843802
NM_021658	Hcn4	hyperpolarization activated cyclic nucleotide-gated potassium channel 4	0.433367586	0.022050108
NM_024141	Duox2	dual oxidase 2	0.443565483	0.002512099
NM_053294	Adora2a	adenosine A2a receptor	0.448932684	0.006610831
NM_001135710	Sec14l5		0.45319148	0.023124069
NM_053544	Sfrp4	secreted frizzled-related protein 4	0.464254873	0.004762944
NM_080894	Pde7b	phosphodiesterase 7B	0.466425359	0.002076483
NM_001107400	Celf4	CUGBP, Elav-like family member 4	0.471613245	0.019419984
NM_053882	Ecm1	extracellular matrix protein 1	0.473367502	2.55E-07
NM_001033961,N	Kcnip2	potassium voltage-gated channel interacting protein 2	0.482784906	0.013140678
NM_030875	Scn1a	sodium voltage-gated channel alpha subunit 1	0.487866666	0.009273831
NM_001107533	Adamtsl3	ADAMTS-like 3	0.491757199	0.04063176
NM_175595	Cacna2d3	calcium voltage-gated channel auxiliary subunit alpha2delta 3	0.493075025	0.017503562
NM_031720	Dio2	iodothyronine deiodinase 2	0.50477183	0.007950121
NM_001105884	Hoxd1	homeo box D1	0.505886421	0.022356274
NM_001108750	Cpne8	copine 8	0.508941304	0.013103961
NM_019345	Slc12a3	solute carrier family 12 member 3	0.512209036	0.049055449
NM_001190459	LOC100361087	hypothetical LOC100361087	0.518491737	0.039681662
NM_001007726	Dnai2	dynein, axonemal, intermediate chain 2	0.523199014	0.009104589
NM_001108061	Amn	amnion associated transmembrane protein	0.54810817	0.038741379
NM_001276707,N	Srebf1	sterol regulatory element binding transcription factor 1	0.556245434	0.011812783
NM_001008320	Rhoj	ras homolog family member J	0.580693316	0.011184995
NM_001108881	Rnf144b	ring finger protein 144B	0.585156181	0.011539822
NM_199105	Fam198b	golgi associated kinase 1B	0.58923031	0.000228659
NM_024371	Slc6a1	solute carrier family 6 member 1	0.589839295	0.009989473
NM_001100512	Gpld1	glycosylphosphatidylinositol specific phospholipase D1	0.597979202	0.019668805
NM_001112716,N	Grik3	glutamate ionotropic receptor kainate type subunit 3	0.598879544	0.010845673
NM_012994	Nxph1	neurexophilin 1	0.599853605	0.001405973
NM_053352	Ackr3	atypical chemokine receptor 3	0.600064841	0.006864116
NM_001012215	Pcdhgb7	protocadherin gamma subfamily B, 7	0.605833803	0.037492738
NM_173151	Pcyt1b	phosphate cytidylyltransferase 1, choline, beta	0.618230805	0.032019948
NM_001107949	Dnajc6	DnaJ heat shock protein family (Hsp40) member C6	0.620788341	0.029973309
NM_031779	Apba1	amyloid beta precursor protein binding family A member 1	0.629392479	0.018761387
NM_001108036	Plekhh1	pleckstrin homology, MyTH4 and FERM domain containing H1	0.63146503	0.025616984
NM_031672	Slc15a2	solute carrier family 15 member 2	0.636243788	0.045034058
NM_001108897	Pskh1	protein serine kinase H1	0.640305324	0.012230094
NM_001109333	Lrrc14b	leucine rich repeat containing 14B	0.641081459	0.042831529
NM_001191757	Frem1	Fras1 related extracellular matrix 1	0.645182518	0.008141812
NM_199504	Pcdha2	protocadherin alpha 2	0.645954922	0.032206219
NM_023025	Cyp2j4	cytochrome P450, family 2, subfamily j, polypeptide 4	0.669988788	0.015458869
NM_001106578	Sema3c	semaphorin 3C	0.675334069	0.027766582
NM_001270807,N	Sphk1	sphingosine kinase 1	0.676104448	0.046936434
NM_001105932	Vps29	VPS29 retromer complex component	0.676354549	0.000360785
NM_001114602	Pcdhb5	protocadherin beta 5	0.679670139	0.012462884
NM_001004277	Pla2g15	phospholipase A2, group XV	0.684140728	0.013277384
NM_001106648	Rpp25l	ribonuclease P/MRP subunit p25 like	0.684741275	0.009506729
NM_001008292	Diablo	diablo, IAP-binding mitochondrial protein	0.685857547	0.000179142
NM_053986	Myo1b	myosin Ib	0.68883597	0.041102114
NM_001109278	Isca2	iron-sulfur cluster assembly 2	0.692051787	0.035691068
NM_001106668	Slc35d1	solute carrier family 35 member D1	0.694338971	0.019767079
NM_001007802	Cmtm6	CKLF-like MARVEL transmembrane domain containing 6	0.701815684	0.038073015
NM_173094	Hmgcs2	3-hydroxy-3-methylglutaryl-CoA synthase 2	0.704058672	0.033591612
NM_001191821	Frmd4a	FERM domain containing 4A	0.705340341	0.004320738
NM_001289778	Map7d2	MAP7 domain containing 2	0.705621497	0.035780989
NM_001271026	Zcchc10	zinc finger CCHC-type containing 10	0.71239681	0.034268636

NM_017218	Erbb3	erb-b2 receptor tyrosine kinase 3	0.712891704	0.009875152
NM_023989	Senp2	SUMO specific peptidase 2	0.713996586	0.013879892
NM_001106095	Lig4	DNA ligase 4	0.714377204	0.019108192
NM_030994	Itga1	integrin subunit alpha 1	0.714391939	0.036724595
NM_024154	Asic1	acid sensing ion channel subunit 1	0.717460566	0.014014056
NM_053725	Itga6	integrin subunit alpha 6	0.718220959	0.000573662
NM_001109014	Tmem164	transmembrane protein 164	0.721948612	0.034035998
NM_013119	Scn3a	sodium voltage-gated channel alpha subunit 3	0.722367879	0.021964174
NM_001107275	Slc39a14	solute carrier family 39 member 14	0.724295488	0.024309902
NM_053886	Lman1	lectin, mannose-binding, 1	0.727318009	0.010286139
NM_053379	Dcx	doublecortin	0.729502957	0.006864881
NM_012802	Pdgfra	platelet derived growth factor receptor alpha	0.730678335	0.038514844
NM_017206	Slc6a6	solute carrier family 6 member 6	0.731732421	0.004388351
NM_001107596	Tmem2	cell migration inducing hyaluronidase 2	0.734671323	0.033125403
NM_019147	Jag1	jagged canonical Notch ligand 1	0.741250877	0.008053911
NM_201990	Pgap1	post-GPI attachment to proteins inositol deacylase 1	0.742931636	0.038386969
NM_001014076	Nol10	nucleolar protein 10	0.743576006	0.011393955
NM_001012201	Cadm1	cell adhesion molecule 1	0.745122006	0.002454913
NM_001127527	Yeats4	YEATS domain containing 4	0.746002878	0.010673263
NM_022380	Stat5b	signal transducer and activator of transcription 5B	0.751412835	0.009365889
NM_001271344	Lpcat2	lysophosphatidylcholine acyltransferase 2	0.751488425	0.039880658
NM_017022	Itgb1	integrin subunit beta 1	0.751658059	0.000656344
NM_053943	Pcdhgc3	protocadherin gamma subfamily C, 3	0.752165614	0.001079378
NM_001100863	Galnt16	polypeptide N-acetylgalactosaminyltransferase 16	0.752214747	0.02731447
NM_001108892	Sall3	spalt-like transcription factor 3	0.753699165	0.025606453
NM_001107548	Usp31	ubiquitin specific peptidase 31	0.755223977	0.030982569
NM_001013990	Srd5a3	steroid 5 alpha-reductase 3	0.760789945	0.015359354
NM_024486	Acvr1	activin A receptor type 1	0.761452399	0.028917579
NM_001126283	Ankrd49	ankyrin repeat domain 49	0.763885647	0.027363235
NM_053467	Tmed10	transmembrane p24 trafficking protein 10	0.765917187	0.002965834
NM_199407	Unc5c	unc-5 netrin receptor C	0.766019514	0.035271861
NM_001006980	Bcap29	B-cell receptor-associated protein 29	0.767537301	0.027649899
NM_001128079	Ctdsp1	CTD small phosphatase 1	0.768993761	0.004059327
NM_001107374	RGD1308601	similar to hypothetical protein	0.76949287	0.031424918
NM_031800	Dedd	death effector domain-containing	0.770677262	0.047690615
NM_013057	F3	coagulation factor III, tissue factor	0.771051135	0.027718463
NM_001173972	Itga8	integrin subunit alpha 8	0.771638824	0.022346724
NM_001015027	Crebl2	cAMP responsive element binding protein-like 2	0.771934622	0.024998338
NM_001171177	Tmtc2	transmembrane O-mannosyltransferase targeting cadherins 2	0.772256114	0.009702114
NM_001191704	Fndc3b	fibronectin type III domain containing 3B	0.775460136	0.018101049
NM_001173472	RGD1562987		0.778326151	0.022140786
NM_001025118	Fam63a	MINDY lysine 48 deubiquitinase 1	0.778336803	0.047343985
NM_053883	Dusp6	dual specificity phosphatase 6	0.779737615	0.039424181
NM_001037139	Pcdhga2	protocadherin gamma subfamily A, 2	0.782777366	0.019784649
NM_001111127	Hist3h2ba	histone cluster 3, H2ba	0.783624983	0.025444052
NM_012880	Sod3	superoxide dismutase 3	0.78420287	0.030056506
NM_080904_1	Arf3		0.784775936	0.006176481
NM_001024303	Lix1l	limb and CNS expressed 1 like	0.785039391	0.006945989
NM_022624	Slc22a23	solute carrier family 22, member 23	0.787541463	0.026849625
NM_001108390	Ndfip2	Nedd4 family interacting protein 2	0.788380182	0.022321693
NM_053502	Abcg1	ATP binding cassette subfamily G member 1	0.788567648	0.042874501
NM_053948	Polr2g	RNA polymerase II subunit G	0.788991936	0.033518327
NM_001191715	Slc30a7	solute carrier family 30 member 7	0.790483615	0.03924914
NM_001005907,N	Efemp2	EGF containing fibulin extracellular matrix protein 2	0.790985604	0.028163392
NM_001037979	Adipor2	adiponectin receptor 2	0.79209672	0.041523386
NM_001127639	Gba	glucosylceramidase beta	0.793632646	0.032446844
NM_001037159	Pcdhgb8	protocadherin gamma subfamily B, 8	0.794921326	0.020106543
NM_001007641	Rnd3	Rho family GTPase 3	0.79509397	0.041960276
NR_130147,NR_13	Tug1		0.795641052	0.011961472
NM_001108747	Rassf3	Ras association domain family member 3	0.797306936	0.044655465
NM_134356	Ptprg	protein tyrosine phosphatase, receptor type, G	0.799474486	0.029473699
NM_021266	Fzd1	frizzled class receptor 1	0.799715791	0.015379829

Appendix Table S2. List of oligonucleotides used in this study.

Oligonucleotides	SOURCE	IDENTIFIER
Stealth RNAi™ siRNA Derl1-MSS289837, target sequence: 5’-CAGACUAUUUAUUCAUGCUUCUCUU-3’	Invitrogen	Cat# 1320001
Stealth RNAi™ siRNA Stat5b-RSS332572, target sequence: 5’-CCCACUUCAGAAACAUGUCCCUGAA-3’	Invitrogen	Cat# 1330001
Stealth RNAi™ siRNA Negative Control Med GC Duplex #2	Invitrogen	Cat# 12935112
Primer: S18 Forward: 5′-TCCAGCACATTTTGCGAGTA-3′	(Hattori *et al*, 2016)	N/A
Primer: S18 Reverse: 5′-CAGTGATGGCGAAGGCTATT-3′	(Hattori *et al.*, 2016)	N/A
Primer: Chop Forward: 5′-CCACCACACCTGAAAGCAGAA-3′	(Feng *et al*, 2003)	N/A
Primer: Chop Reverse: 5′-AGGTGAAAGGCAGGGACTCA-3′	(Feng *et al.*, 2003)	N/A
Primer: β-actin Forward: 5′-TCCTCCCTGGAGAAGAGCTAT-3′	(Kondo *et al*, 2005)	N/A
Primer: β-actin Reverse: 5′-TCCTGCTTGCTGATCCACAT-3′	(Kondo *et al.*, 2005)	N/A
Primer: Stat5b (rat) Forward: 5′-GGGCATCACCATTGCTTGGAAG-3′	(Barkai *et al*, 2000)	N/A
Primer: Stat5b (rat) Reverse: 5′-CCGGATAGAGAAGTCTCTTGTGG-3′	This paper	N/A
Primer: Stat5b (mouse) Forward: 5′-TACTACACACCGGTCCCCTG-3′	This paper	N/A
Primer: Stat5b (mouse) Reverse: 5′-ATGCATTTGCAAACTCGGGG-3′	This paper	N/A

Appendix Table S3. List of primary and secondary antibodies used in this study.

Antibodies	SOURCE	IDENTIFIER
Mouse-anti-Derlin-1	Sigma-Aldrich	SAB4200148; RRID: AB_10624068
Rat-anti-BrdU	AbD Serotec	OBT0030; RRID: AB_609568
Goat-anti-DCX	Santa Cruz	sc-8066; RRID: AB_2088494
Rabbit-anti-Prox1	Millipore	AB5475; RRID: AB_177485
Mouse-anti-NeuN	Millipore	MAB377; RRID: AB_2298772
Mouse-anti-Ki67	BD PharMingen	550609; RRID: AB_393778
Rabbit-anti-Tbr2	Abcam	ab23345; RRID: AB_778267
Mouse-anti-Nestin	Millipore	MAB353; RRID: AB_94911
Rabbit-anti-GFAP	Dako	N1506; RRID: AB_10013482
Chicken-anti-GFAP	Millipore	AB5541; RRID: AB_177521
Mouse-anti-S100β	Sigma–Aldrich	s2532; RRID: AB_477499
Rabbit-anti-Iba1	Wako	019-19741; RRID: AB_839504
Goat-anti-Sox2	Santa Cruz	Sc-17320; RRID: AB_2286684
Rabbit-anti-Active caspase3	R and D Systems	AF835; RRID: AB_2243952
Rat-anti-Somatostatin	Millipore	MAB354; RRID: AB_2255365
Mouse-anti-Parvalbumin	Millipore	MAB1572; RRID: AB_2174013
Rabbit-anti-HA-Tag	Cell Signaling	3724; RRID: AB_1549585
Mouse-anti-Stat5b	Santa Cruz	Sc1656-; RRID: AB_2197067
Mouse-anti-Actin	Sigma–Aldrich	A4700; RRID: AB_476730
Anti-Mouse IgG, HRP-linked antibody	GE Healthcare	NA931; RRID: AB_772210
CF®555, Donkey Anti-Mouse IgG (H+L), Highly Cross-Adsorbed	Biotium	20037; RRID: AB_10559035
CF®555, Donkey Anti-Rabbit IgG (H+L), Highly Cross-Adsorbed	Biotium	20038; RRID: AB_10558011
CF®488A, Donkey Anti-Chicken IgY (H+L), Highly Cross-Adsorbed	Biotium	20166; RRID: AB_10854387
CF®488A, Donkey Anti-Mouse IgG (H+L), Highly Cross-Adsorbed	Biotium	20014; RRID: AB_10561327

CF®568, Donkey Anti-Rat IgG (H+L), Highly Cross-Adsorbed	Biotium	20092; RRID: AB_10559037
CF®647, Donkey Anti-Rabbit IgG (H+L), Highly Cross-Adsorbed	Biotium	20047; RRID: AB_10853792
CF®647, Donkey Anti-Goat IgG (H+L), Highly Cross-Adsorbed	Biotium	20048; RRID: AB_10853455

References

Barkai U, Prigent-Tessier A, Tessier C, Gibori GB, Gibori G (2000) Involvement of SOCS-1, the suppressor of cytokine signaling, in the prevention of prolactin-responsive gene expression in decidual cells. *Mol Endocrinol* 14: 554-563

Feng B, Yao PM, Li Y, Devlin CM, Zhang D, Harding HP, Sweeney M, Rong JX, Kuriakose G, Fisher EA *et al* (2003) The endoplasmic reticulum is the site of cholesterol-induced cytotoxicity in macrophages. *Nat Cell Biol* 5: 781-792

Hattori K, Naguro I, Okabe K, Funatsu T, Furutani S, Takeda K, Ichijo H (2016) ASK1 signalling regulates brown and beige adipocyte function. *Nat Commun* 7: 11158

Kondo S, Murakami T, Tatsumi K, Ogata M, Kanemoto S, Otori K, Iseki K, Wanaka A, Imaizumi K (2005) OASIS, a CREB/ATF-family member, modulates UPR signalling in astrocytes. *Nat Cell Biol* 7: 186-194
