# Supplementary figures and images for: The Derlin-1-Stat5b axis maintains homeostasis of adult hippocampal neurogenesis (part 1 of 2)
Source: EMBO Rep. 2024 Jul 30;25(8):26. doi: 10.1038/s44319-024-00205-7 (PMC11316036; doi:10.1038/s44319-024-00205-7)

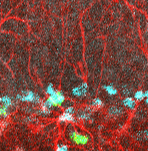

Supplement: Supplementary file 3 — Source data Fig. 1 [file 44319_2024_205_MOESM3_ESM.zip › Source_data_Figure1/1N/Left images.tif]

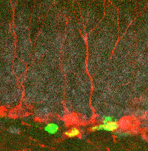

Supplement: Supplementary file 3 — Source data Fig. 1 [file 44319_2024_205_MOESM3_ESM.zip › Source_data_Figure1/1N/Right images.tif]

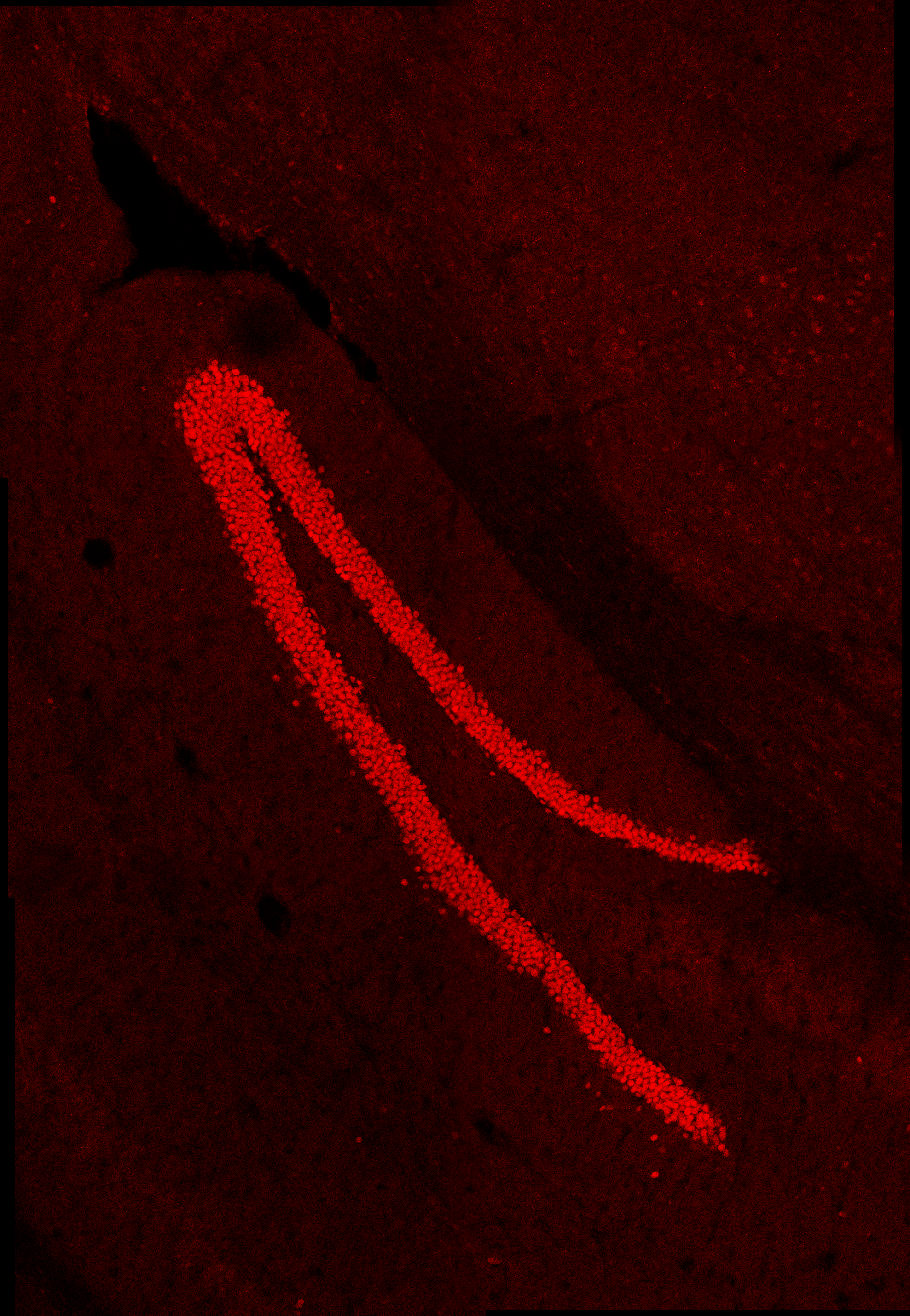

Supplement: Supplementary file 3 — Source data Fig. 1 [file 44319_2024_205_MOESM3_ESM.zip › Source_data_Figure1/1H/Derl1f:f/Prox1.tif]

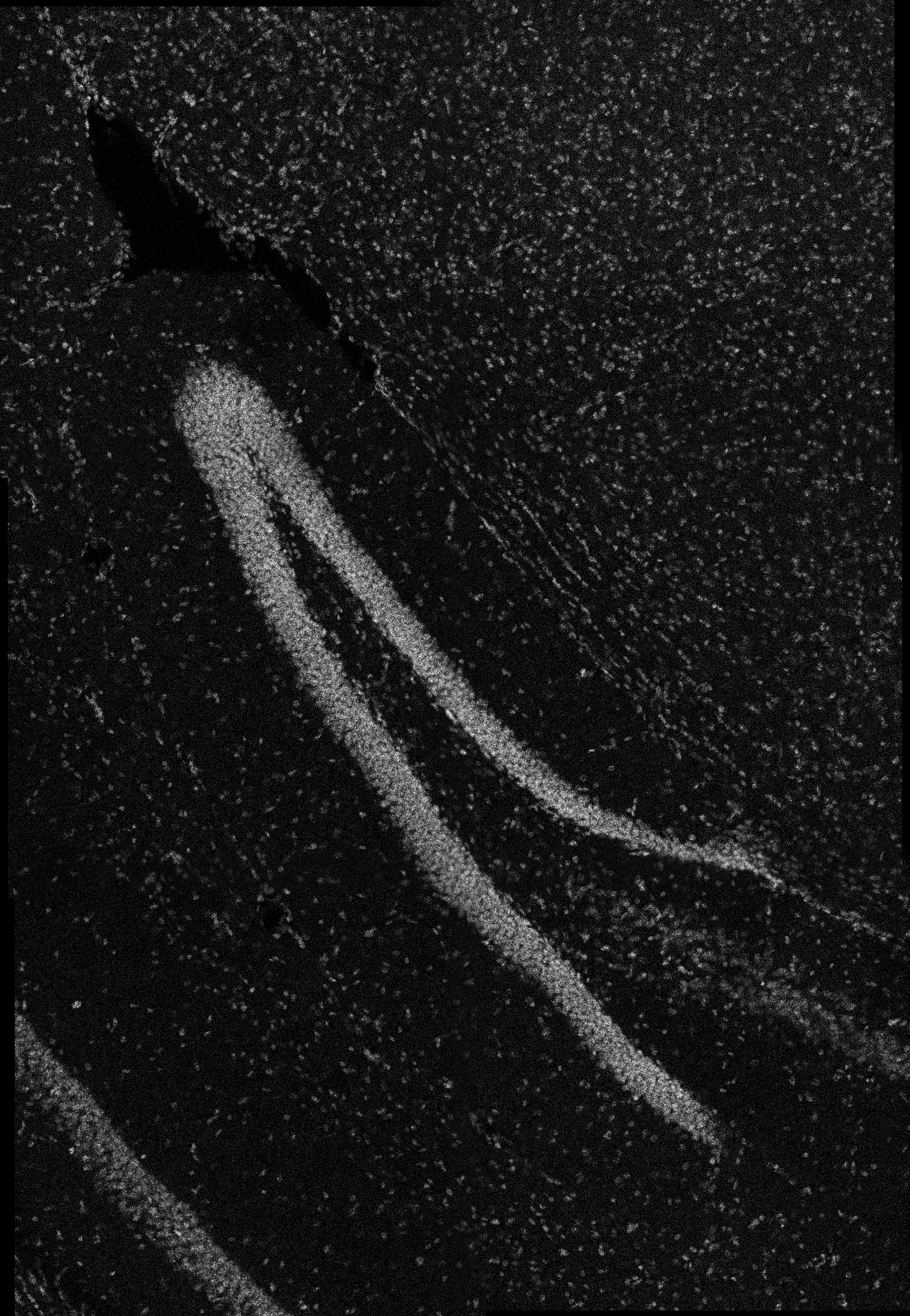

Supplement: Supplementary file 3 — Source data Fig. 1 [file 44319_2024_205_MOESM3_ESM.zip › Source_data_Figure1/1H/Derl1f:f/Hoechst.tif]

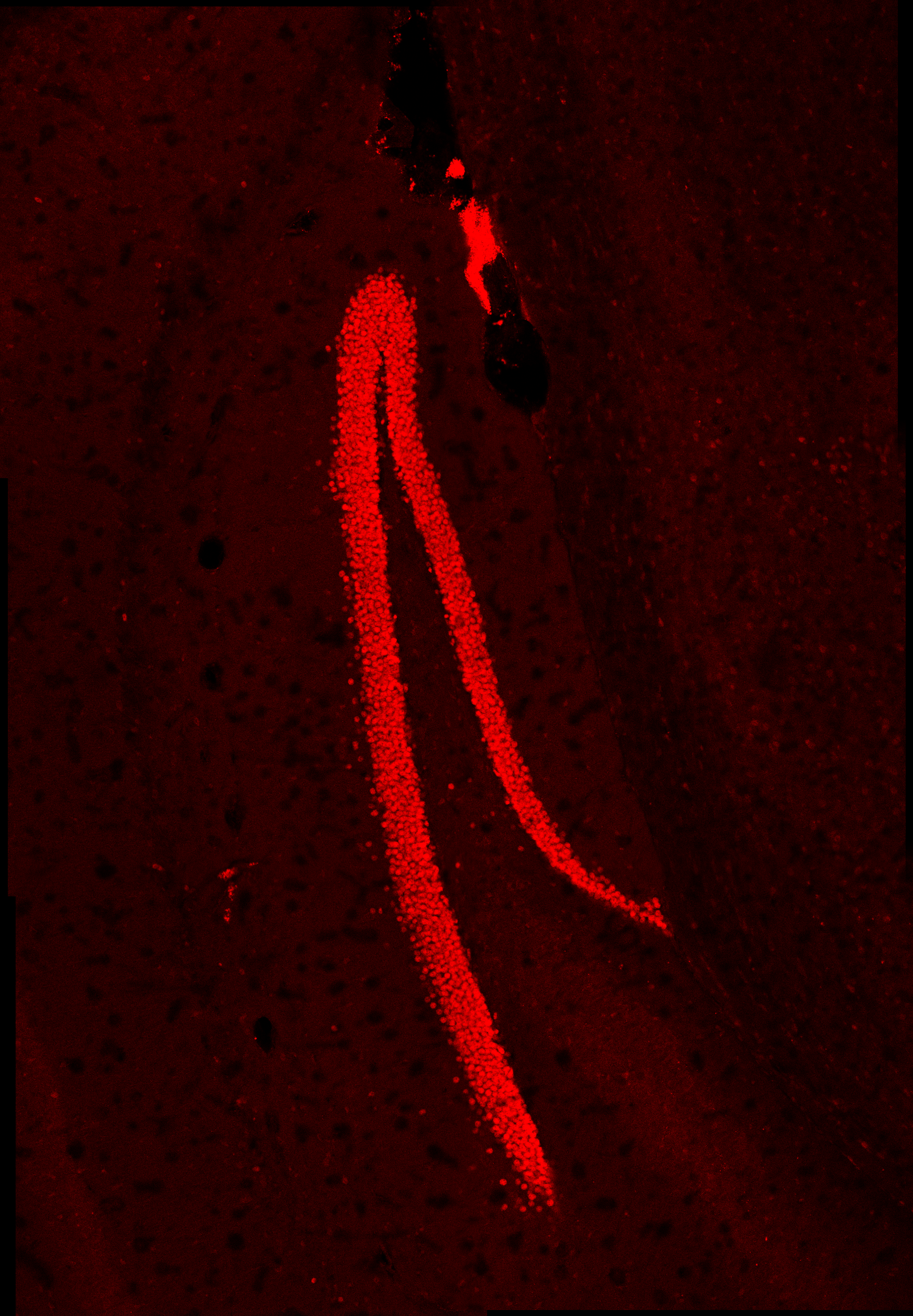

Supplement: Supplementary file 3 — Source data Fig. 1 [file 44319_2024_205_MOESM3_ESM.zip › Source_data_Figure1/1H/Derl1NesCre/Prox1.tif]

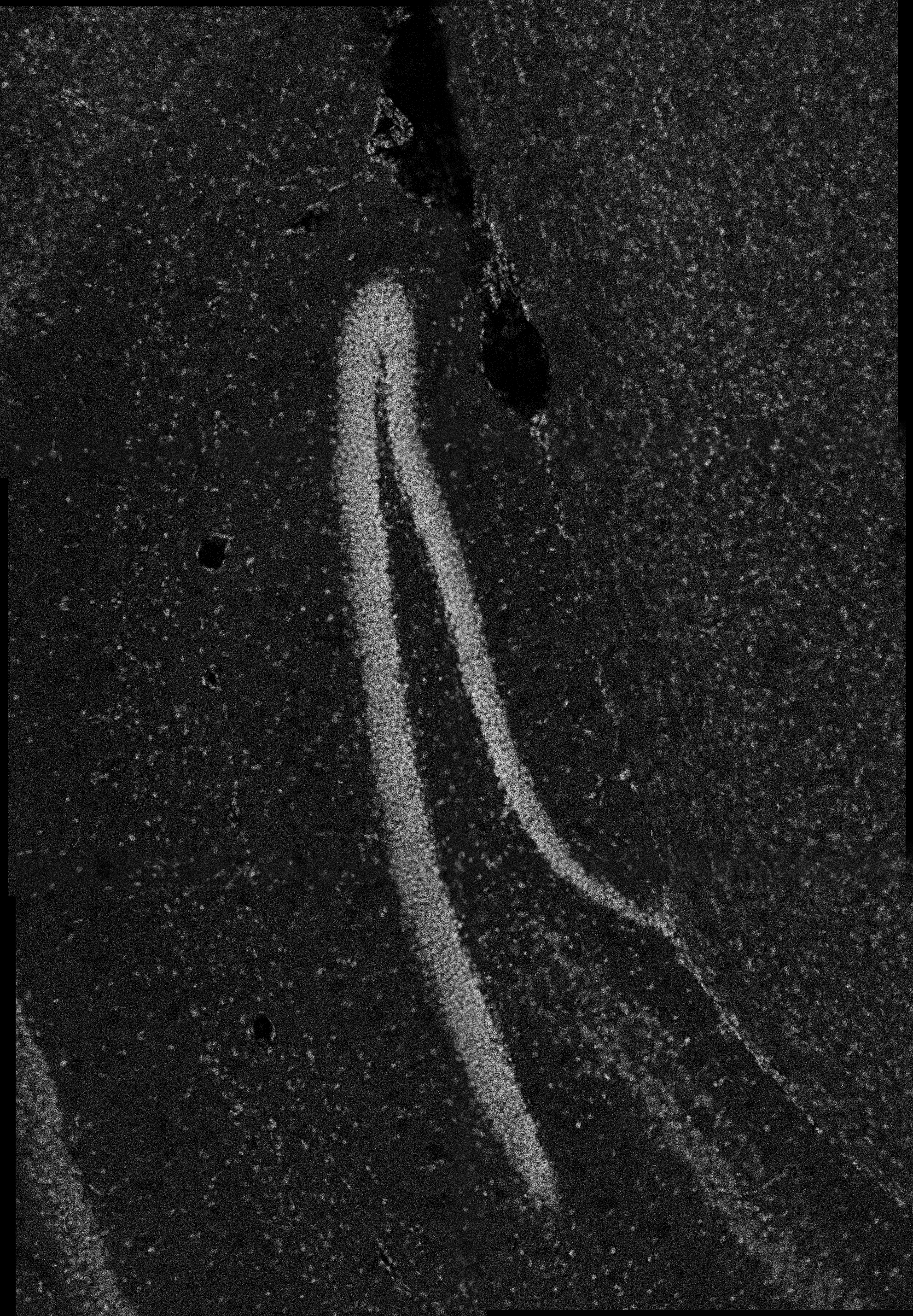

Supplement: Supplementary file 3 — Source data Fig. 1 [file 44319_2024_205_MOESM3_ESM.zip › Source_data_Figure1/1H/Derl1NesCre/Hoechst.tif]

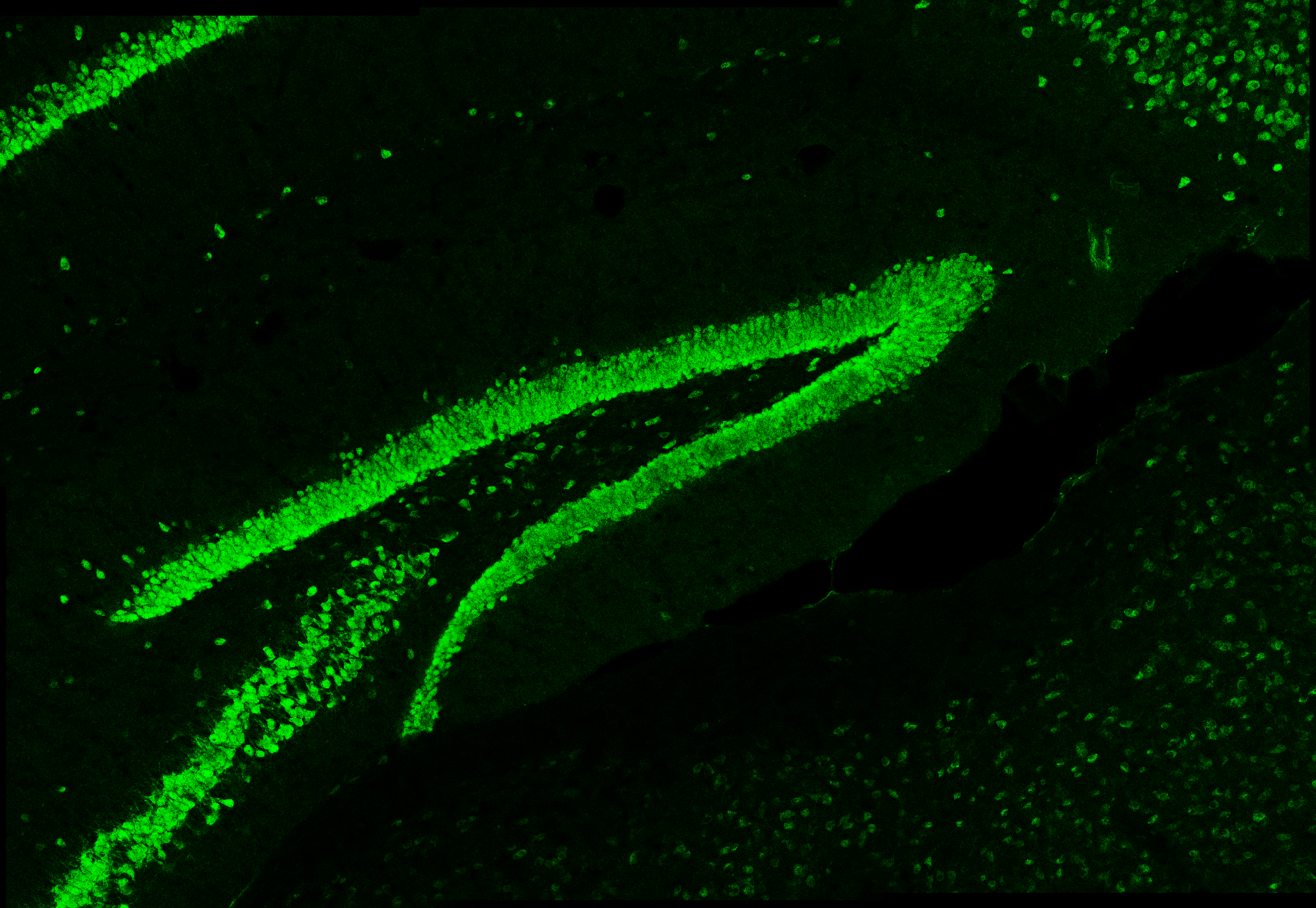

Supplement: Supplementary file 3 — Source data Fig. 1 [file 44319_2024_205_MOESM3_ESM.zip › Source_data_Figure1/1K/Derl1f:f/NeuN.tif]

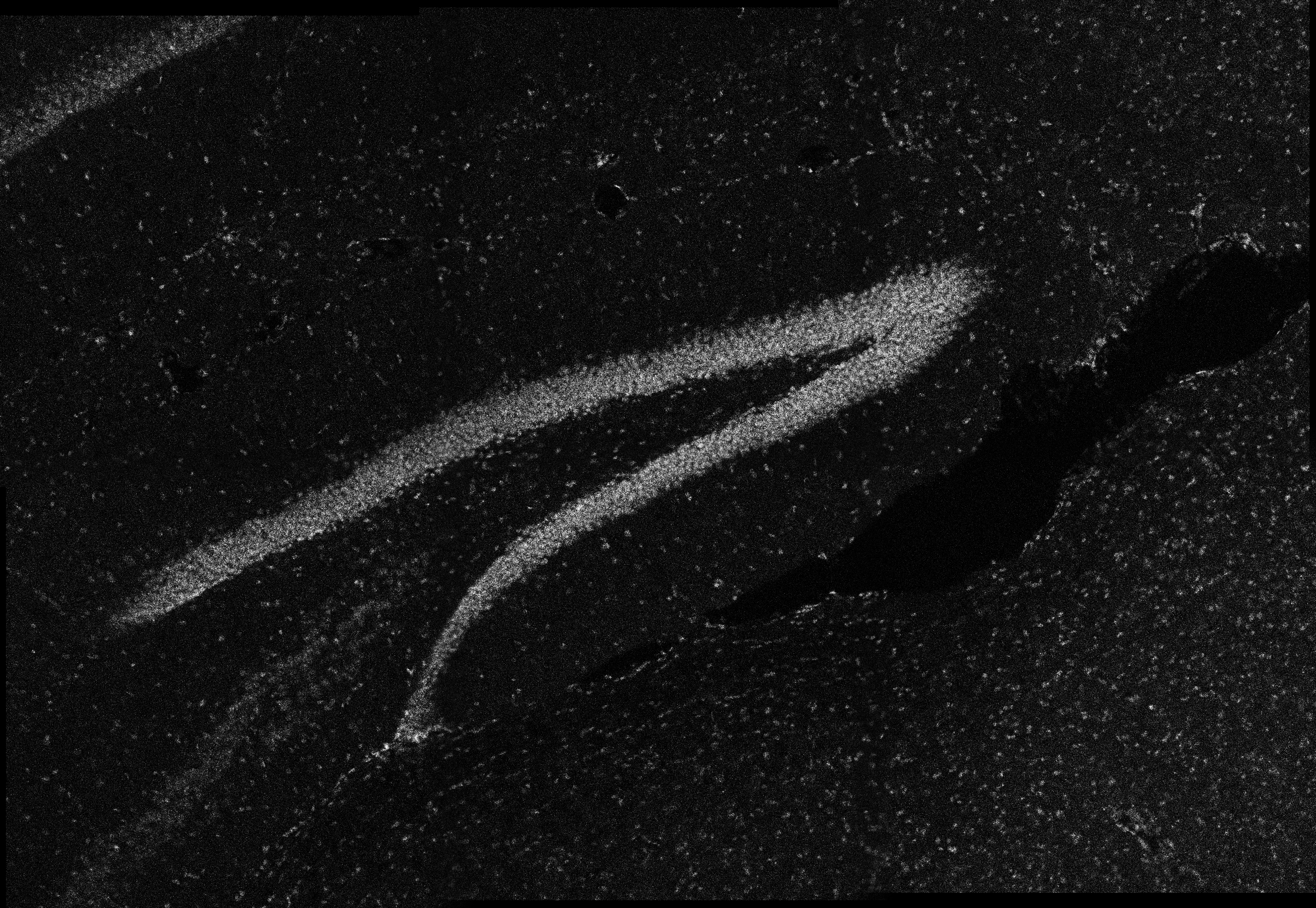

Supplement: Supplementary file 3 — Source data Fig. 1 [file 44319_2024_205_MOESM3_ESM.zip › Source_data_Figure1/1K/Derl1f:f/Hoechst.tif]

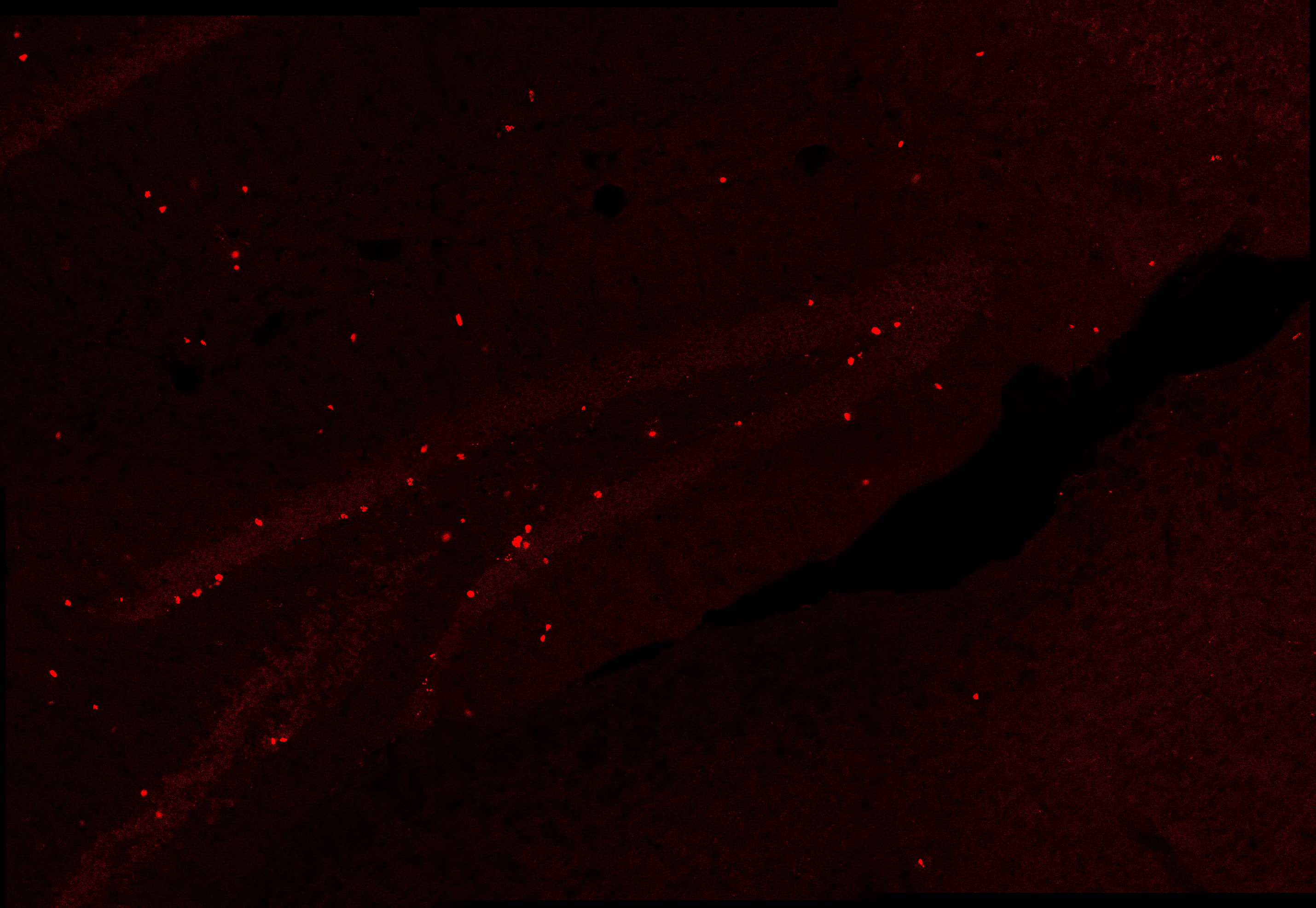

Supplement: Supplementary file 3 — Source data Fig. 1 [file 44319_2024_205_MOESM3_ESM.zip › Source_data_Figure1/1K/Derl1f:f/BrdU.tif]

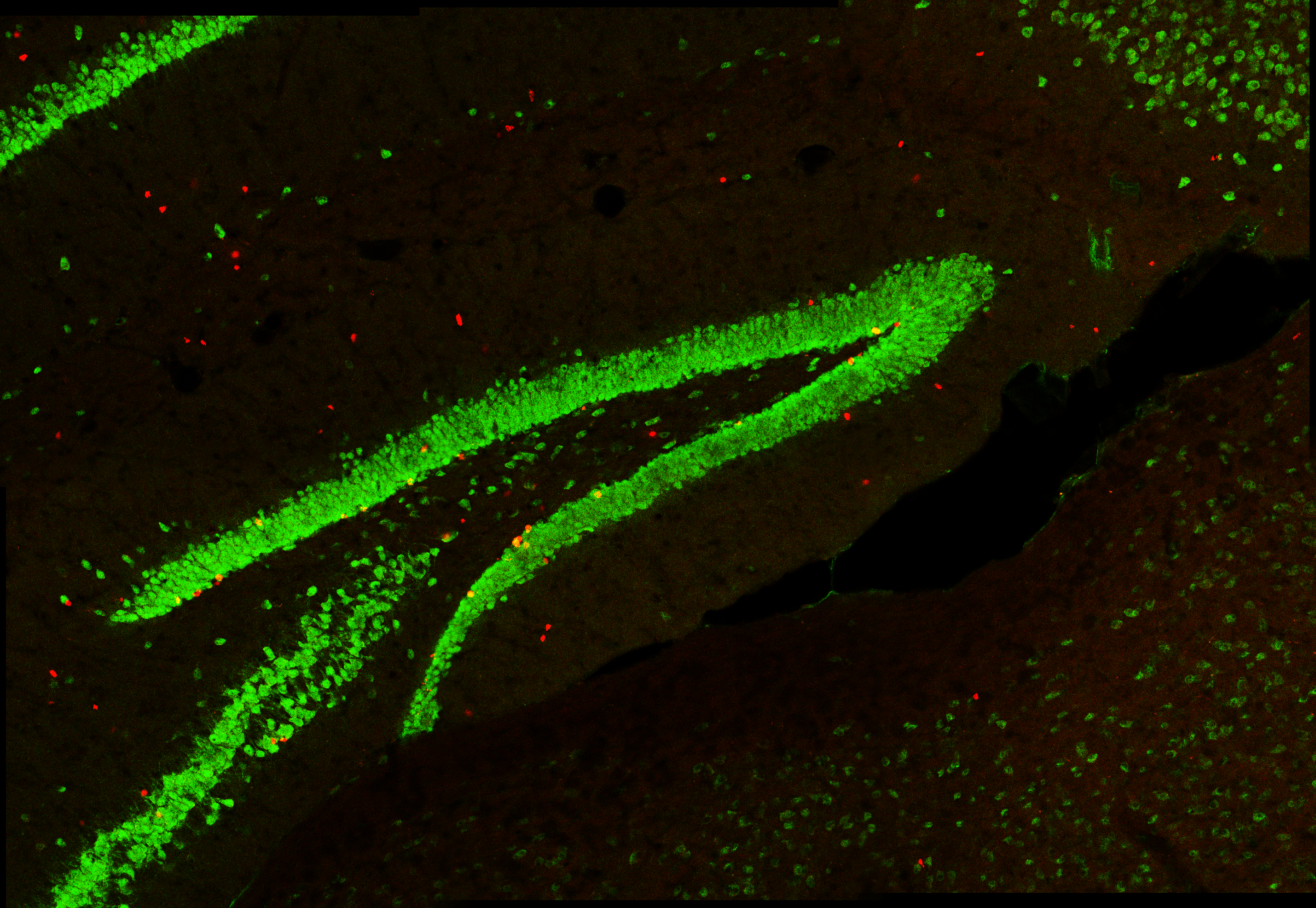

Supplement: Supplementary file 3 — Source data Fig. 1 [file 44319_2024_205_MOESM3_ESM.zip › Source_data_Figure1/1K/Derl1f:f/Merge.tif]

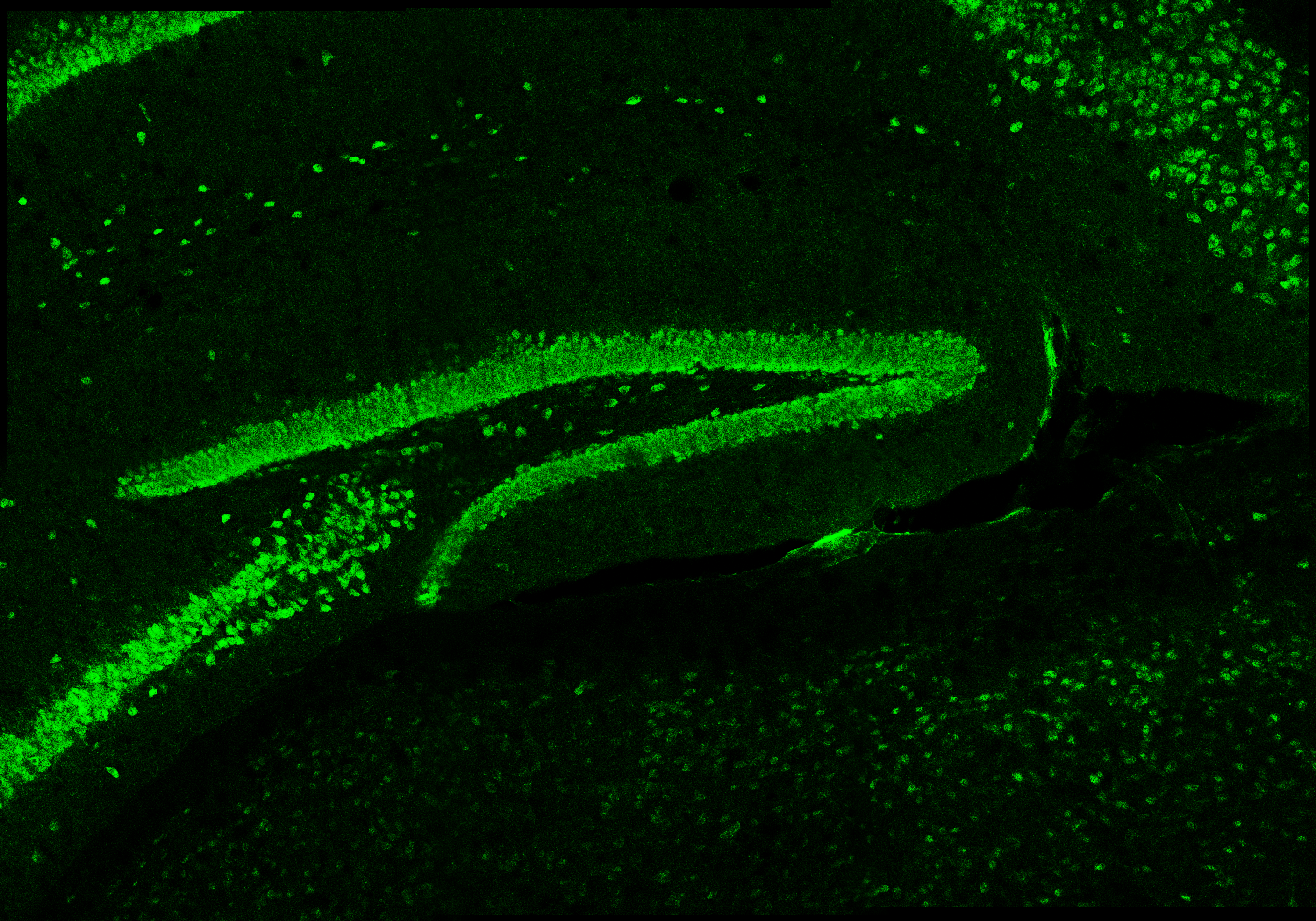

Supplement: Supplementary file 3 — Source data Fig. 1 [file 44319_2024_205_MOESM3_ESM.zip › Source_data_Figure1/1K/Derl1NesCre/NeuN.tif]

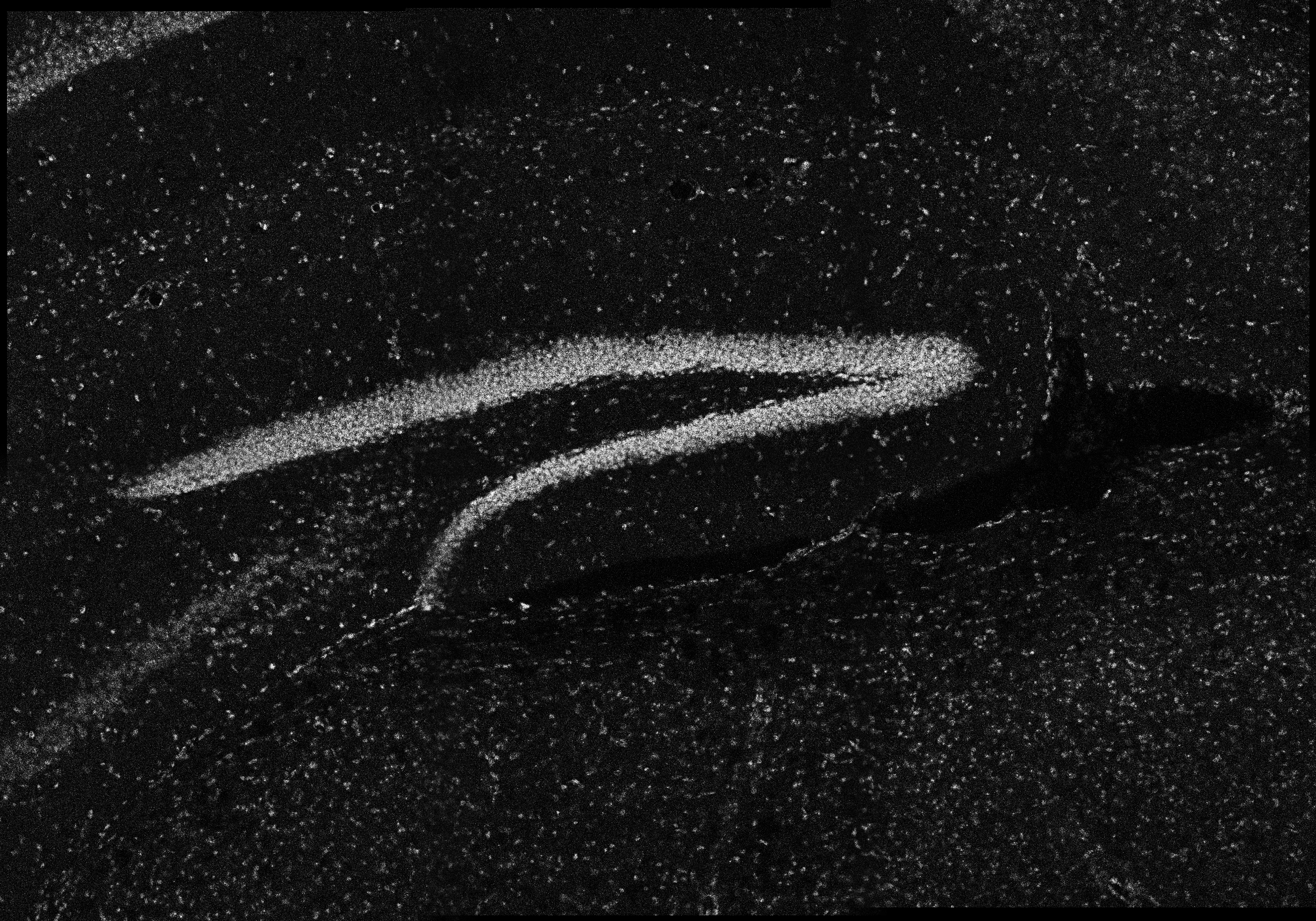

Supplement: Supplementary file 3 — Source data Fig. 1 [file 44319_2024_205_MOESM3_ESM.zip › Source_data_Figure1/1K/Derl1NesCre/Hoechst.tif]

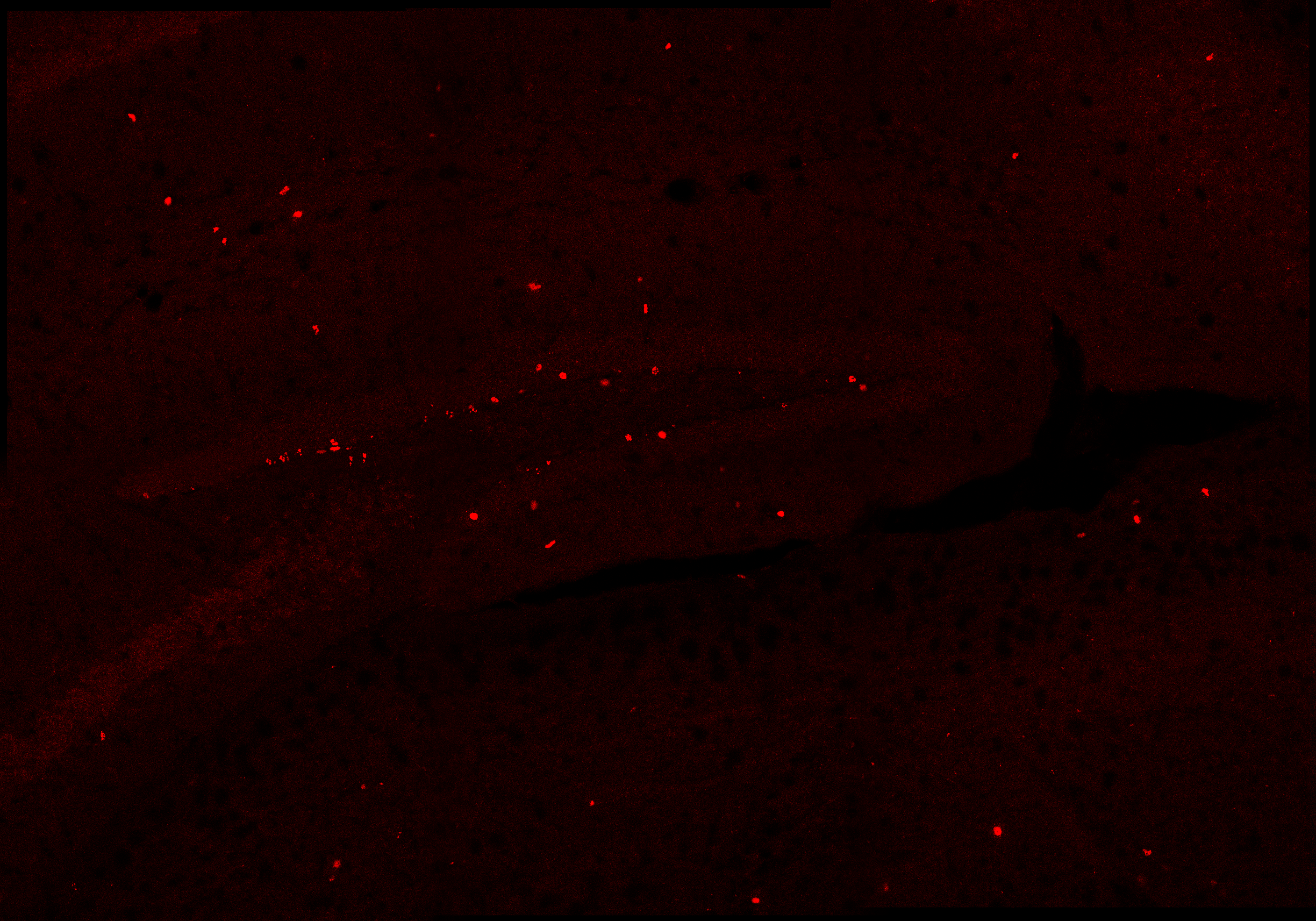

Supplement: Supplementary file 3 — Source data Fig. 1 [file 44319_2024_205_MOESM3_ESM.zip › Source_data_Figure1/1K/Derl1NesCre/BrdU.tif]

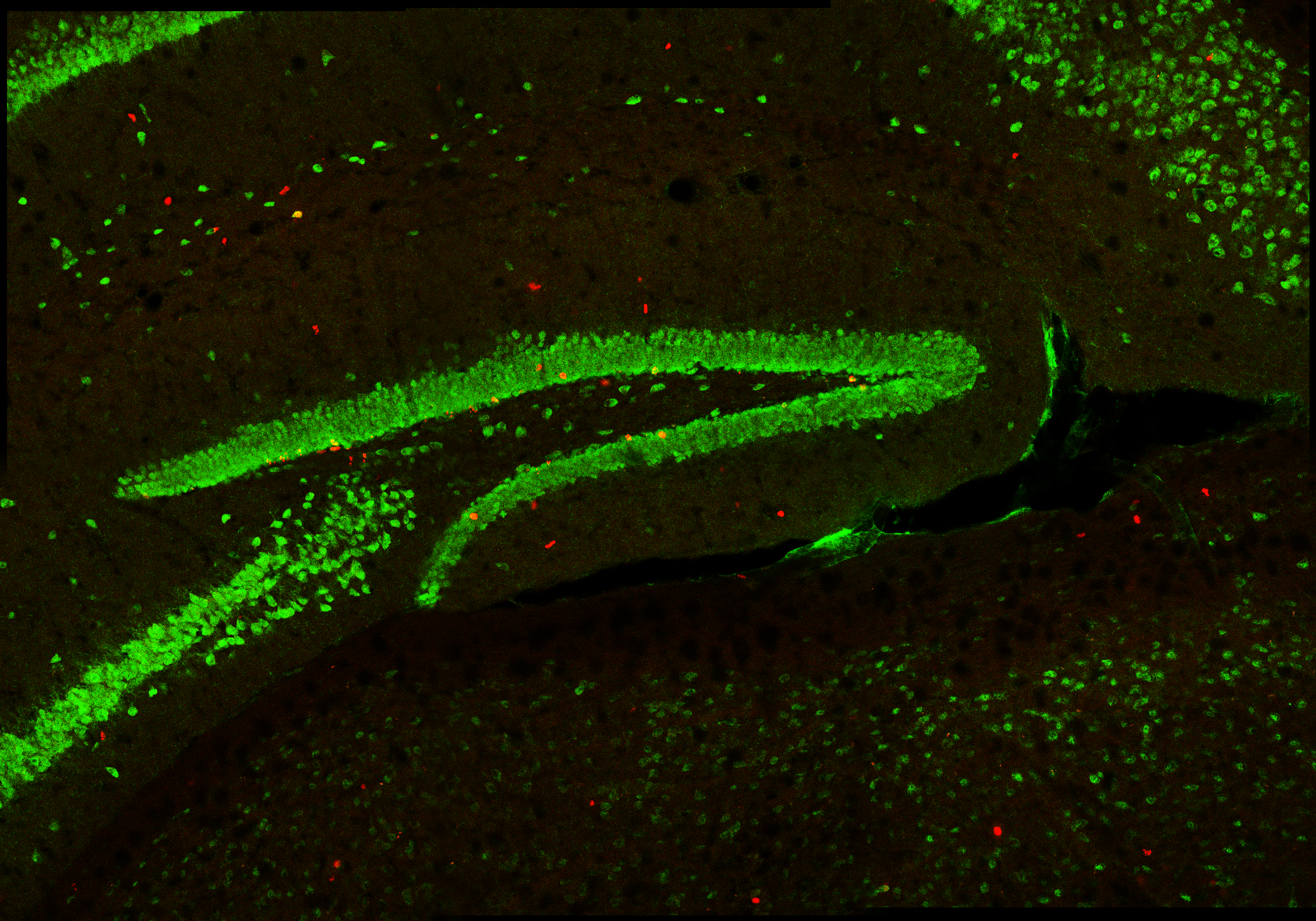

Supplement: Supplementary file 3 — Source data Fig. 1 [file 44319_2024_205_MOESM3_ESM.zip › Source_data_Figure1/1K/Derl1NesCre/Merge.tif]

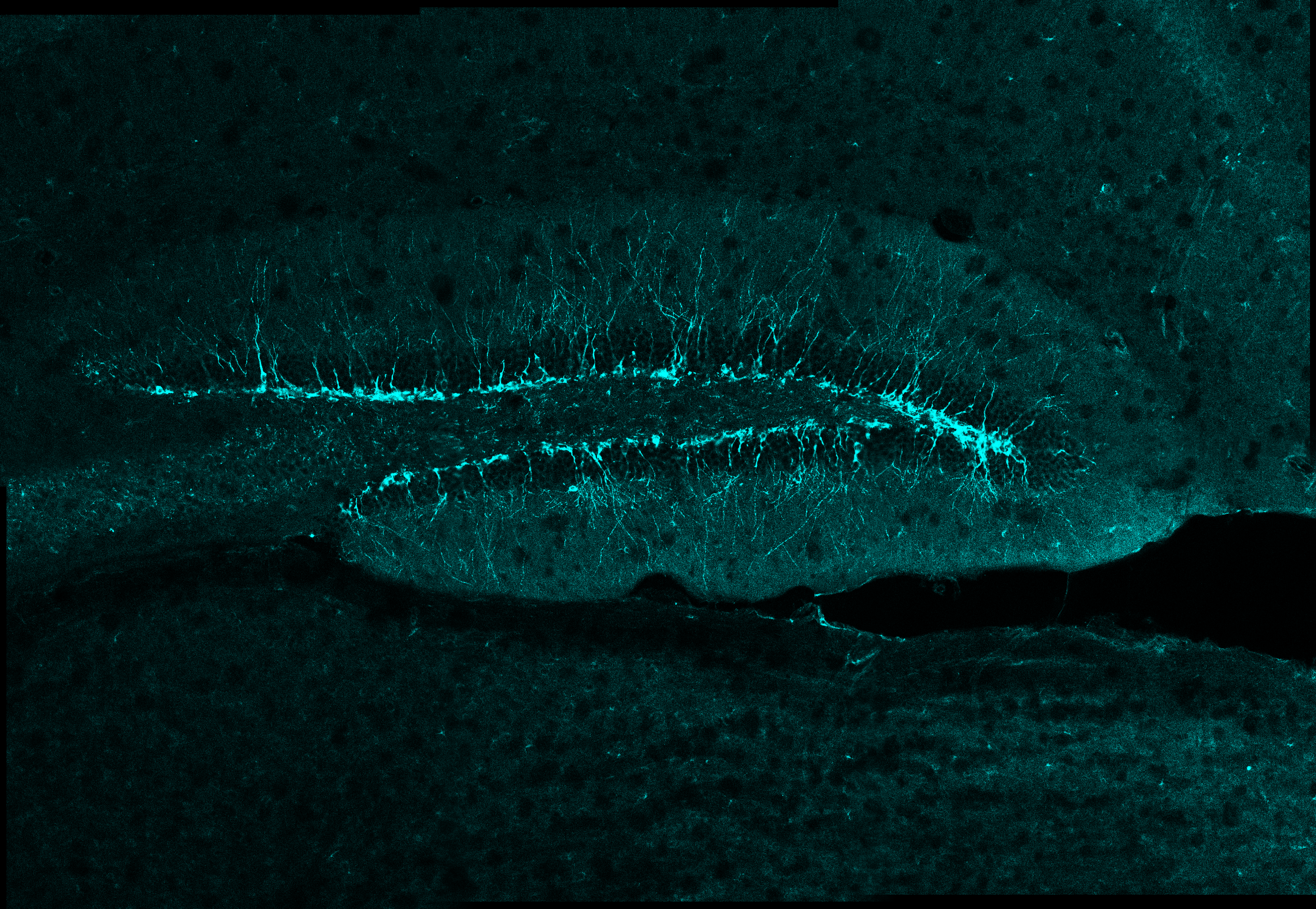

Supplement: Supplementary file 3 — Source data Fig. 1 [file 44319_2024_205_MOESM3_ESM.zip › Source_data_Figure1/1E/Derl1f:f/DCX.tif]

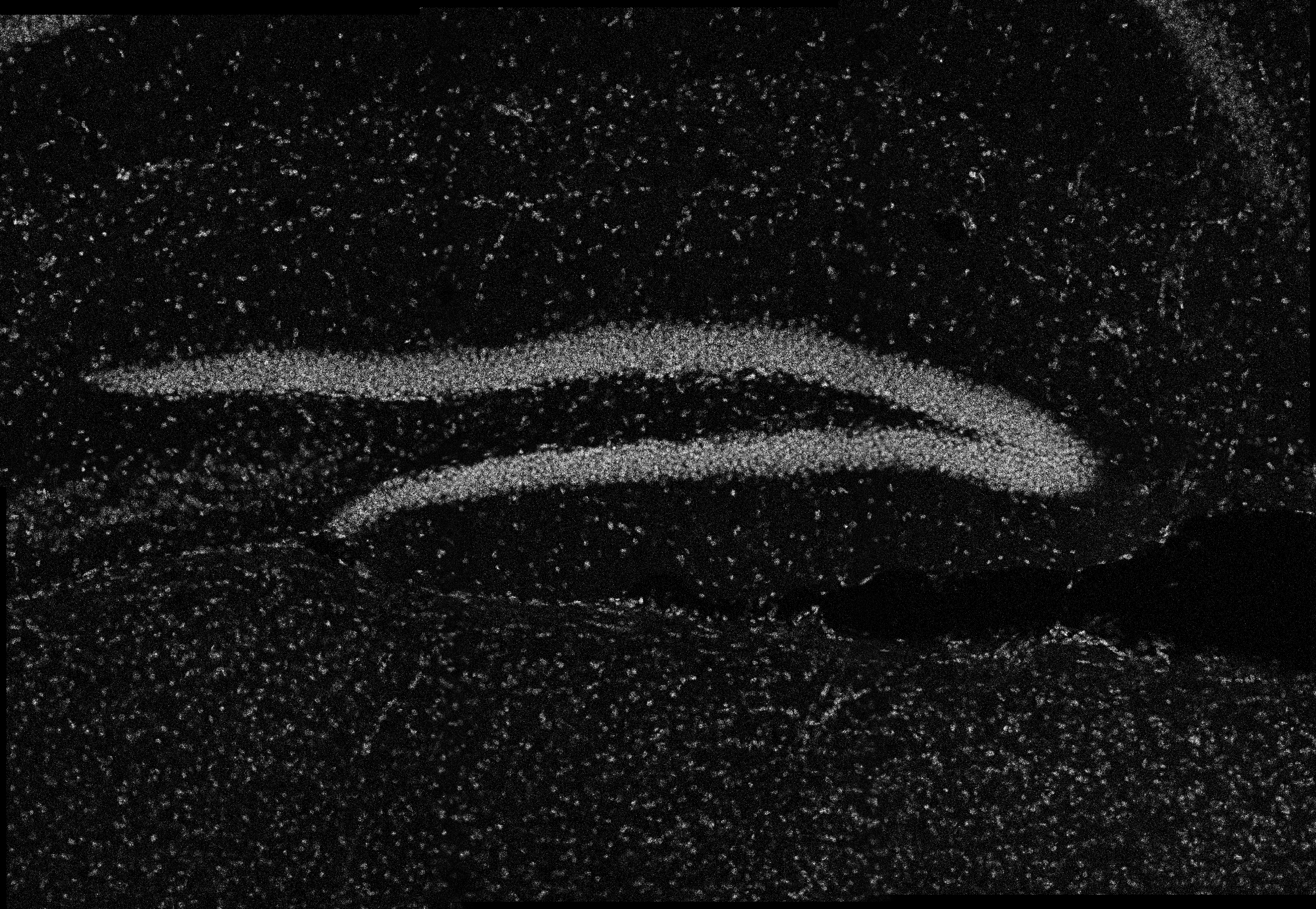

Supplement: Supplementary file 3 — Source data Fig. 1 [file 44319_2024_205_MOESM3_ESM.zip › Source_data_Figure1/1E/Derl1f:f/Hoechst.tif]

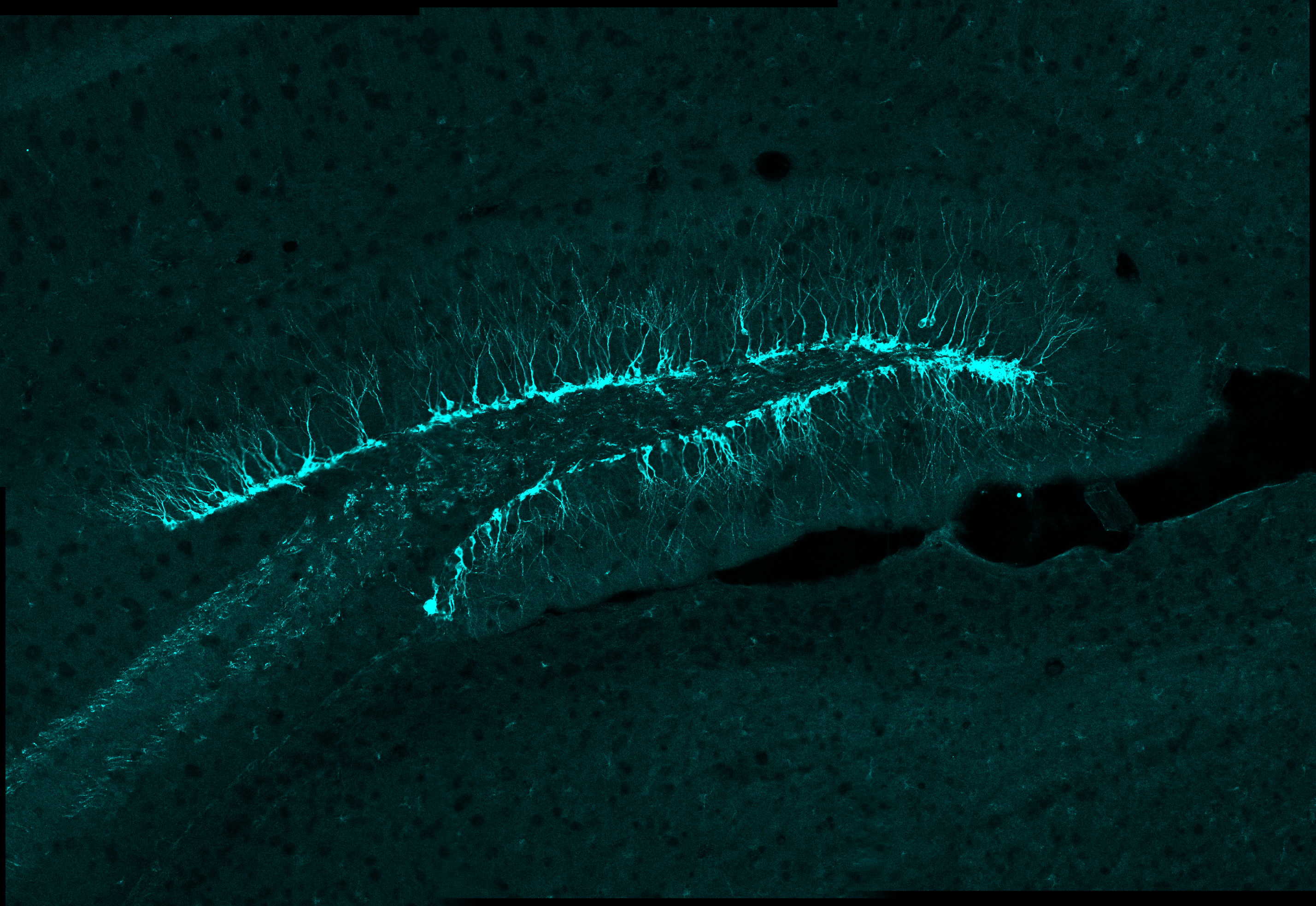

Supplement: Supplementary file 3 — Source data Fig. 1 [file 44319_2024_205_MOESM3_ESM.zip › Source_data_Figure1/1E/Derl1NesCre/DCX.tif]

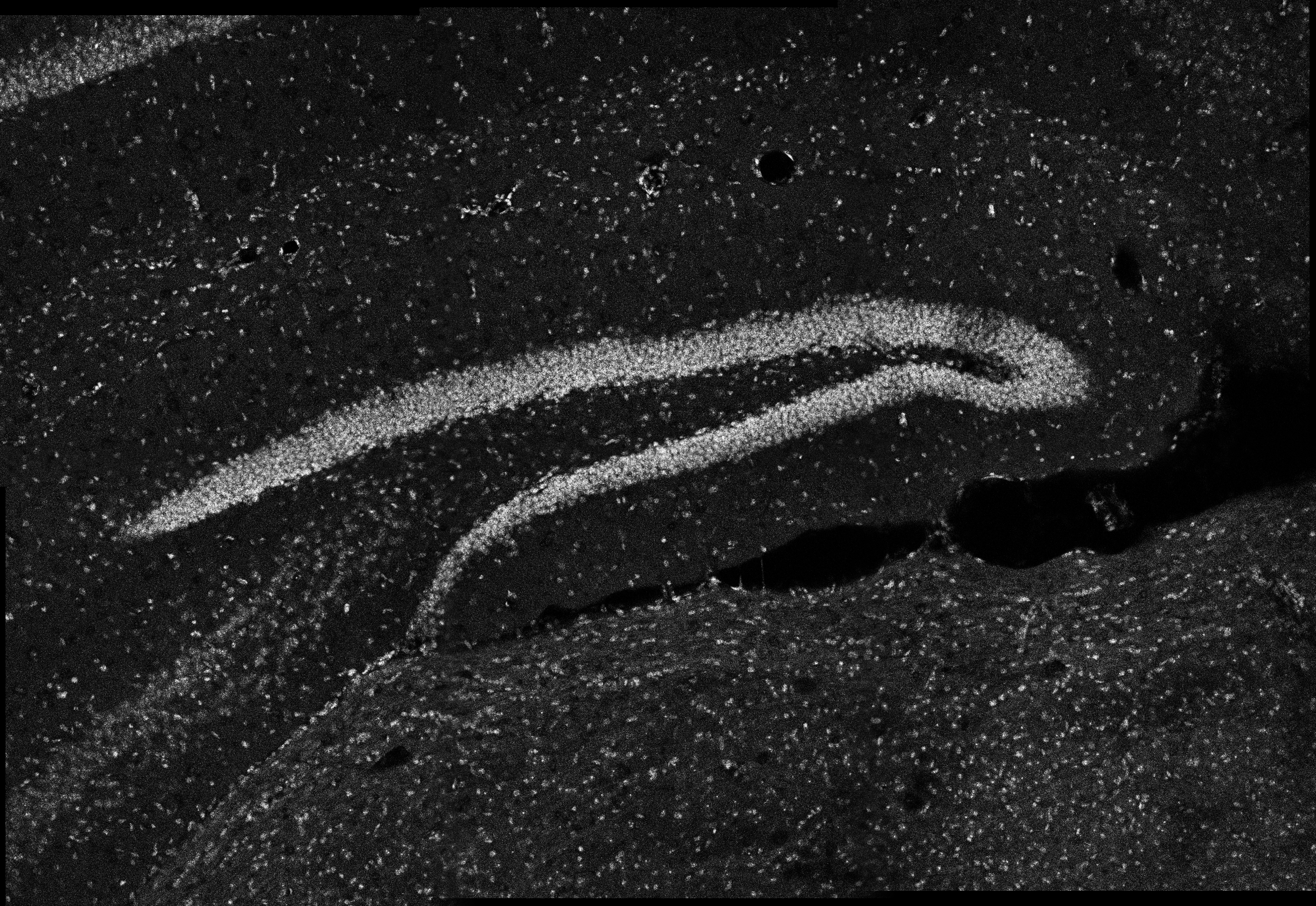

Supplement: Supplementary file 3 — Source data Fig. 1 [file 44319_2024_205_MOESM3_ESM.zip › Source_data_Figure1/1E/Derl1NesCre/Hoechst.tif]

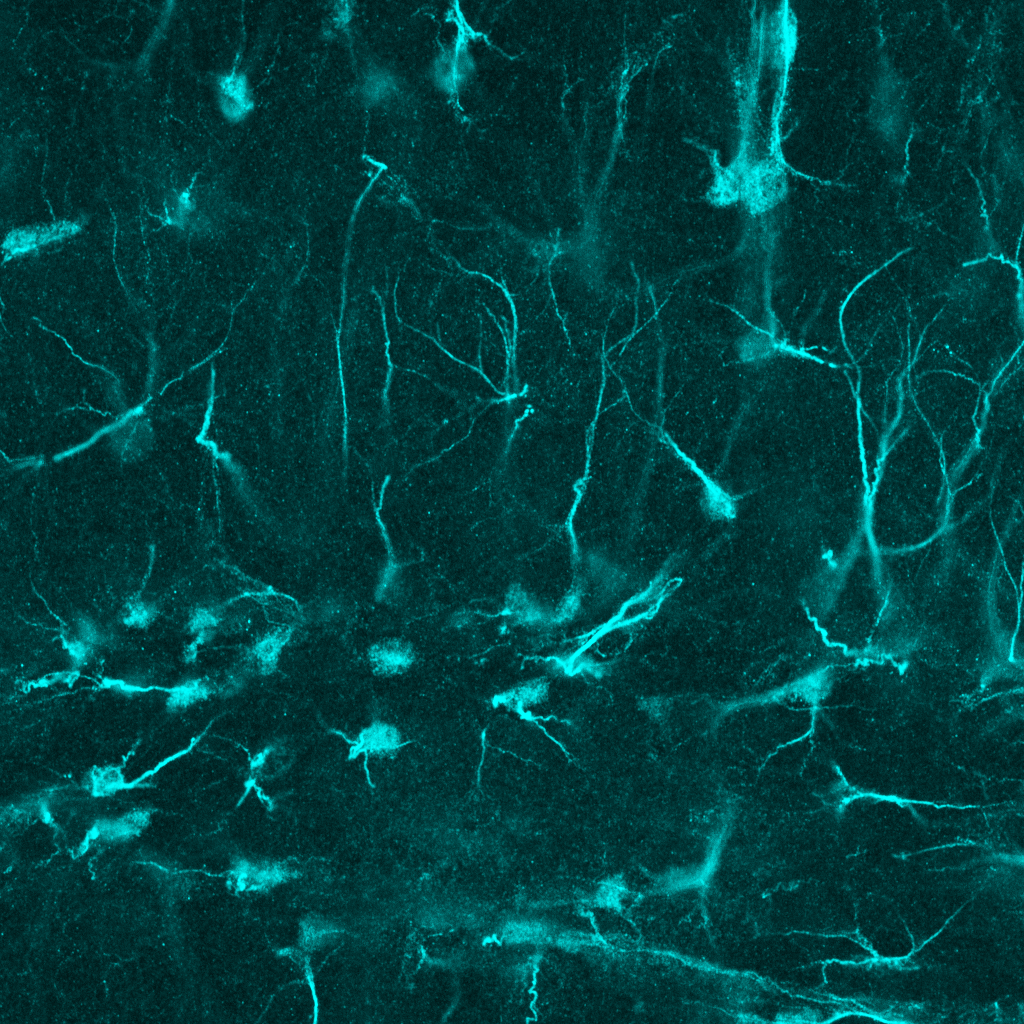

Supplement: Supplementary file 3 — Source data Fig. 1 [file 44319_2024_205_MOESM3_ESM.zip › Source_data_Figure1/1Q/Derl1f:f/GFAPsox2.tif]

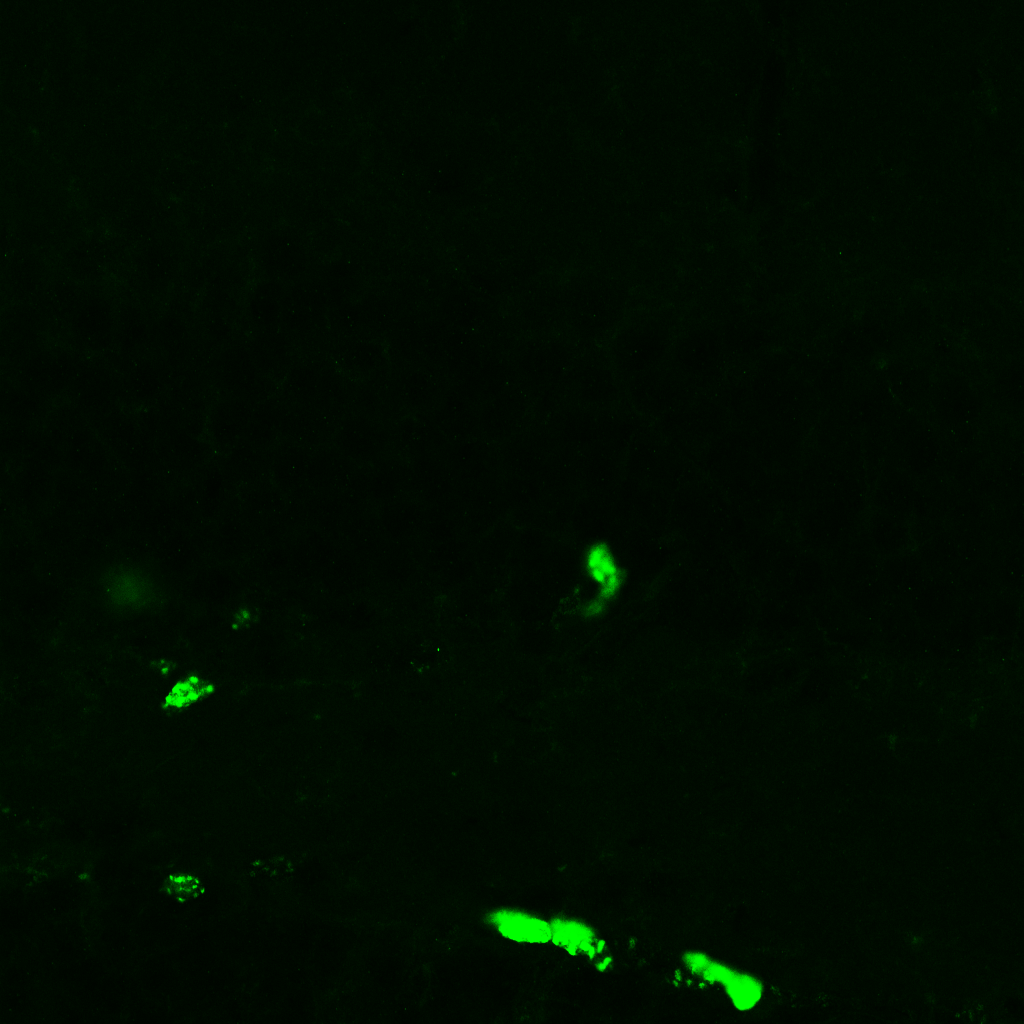

Supplement: Supplementary file 3 — Source data Fig. 1 [file 44319_2024_205_MOESM3_ESM.zip › Source_data_Figure1/1Q/Derl1f:f/Ki67.tif]

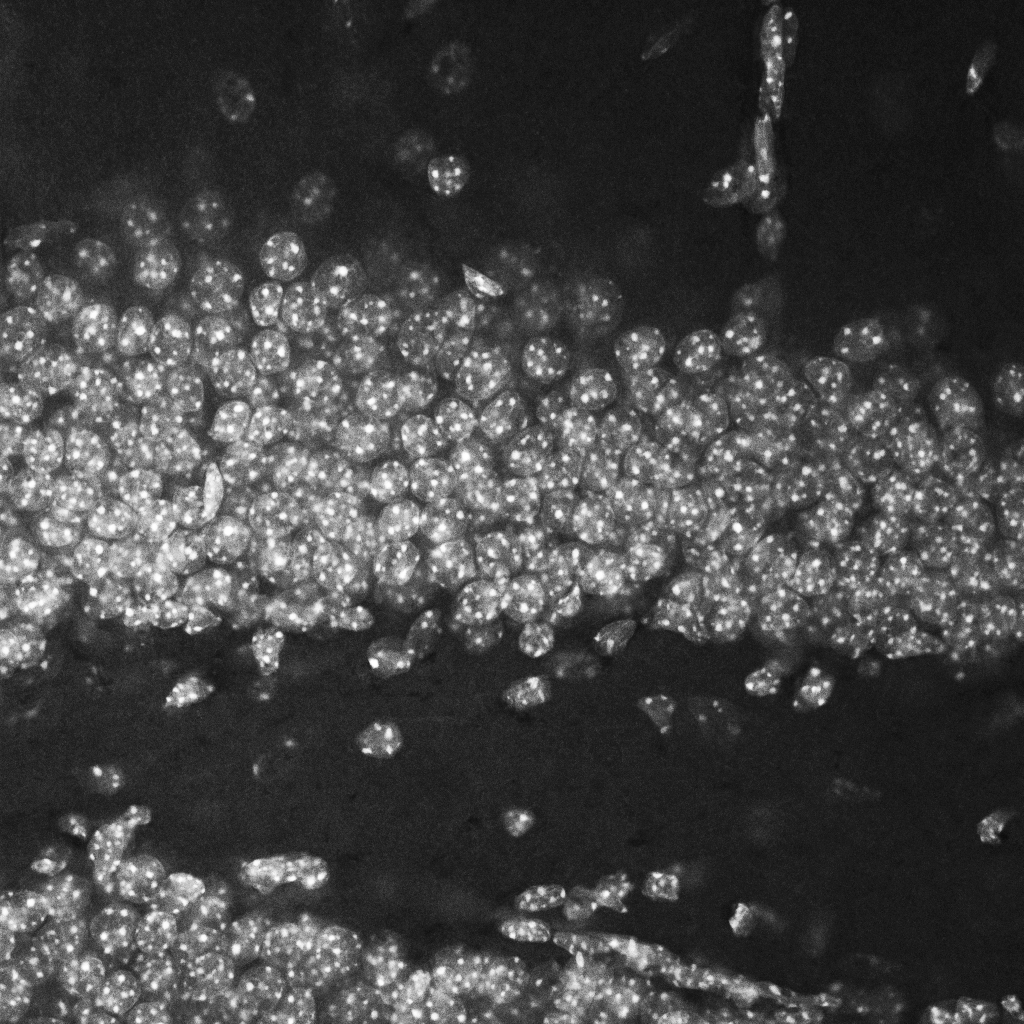

Supplement: Supplementary file 3 — Source data Fig. 1 [file 44319_2024_205_MOESM3_ESM.zip › Source_data_Figure1/1Q/Derl1f:f/Hoechst.tif]

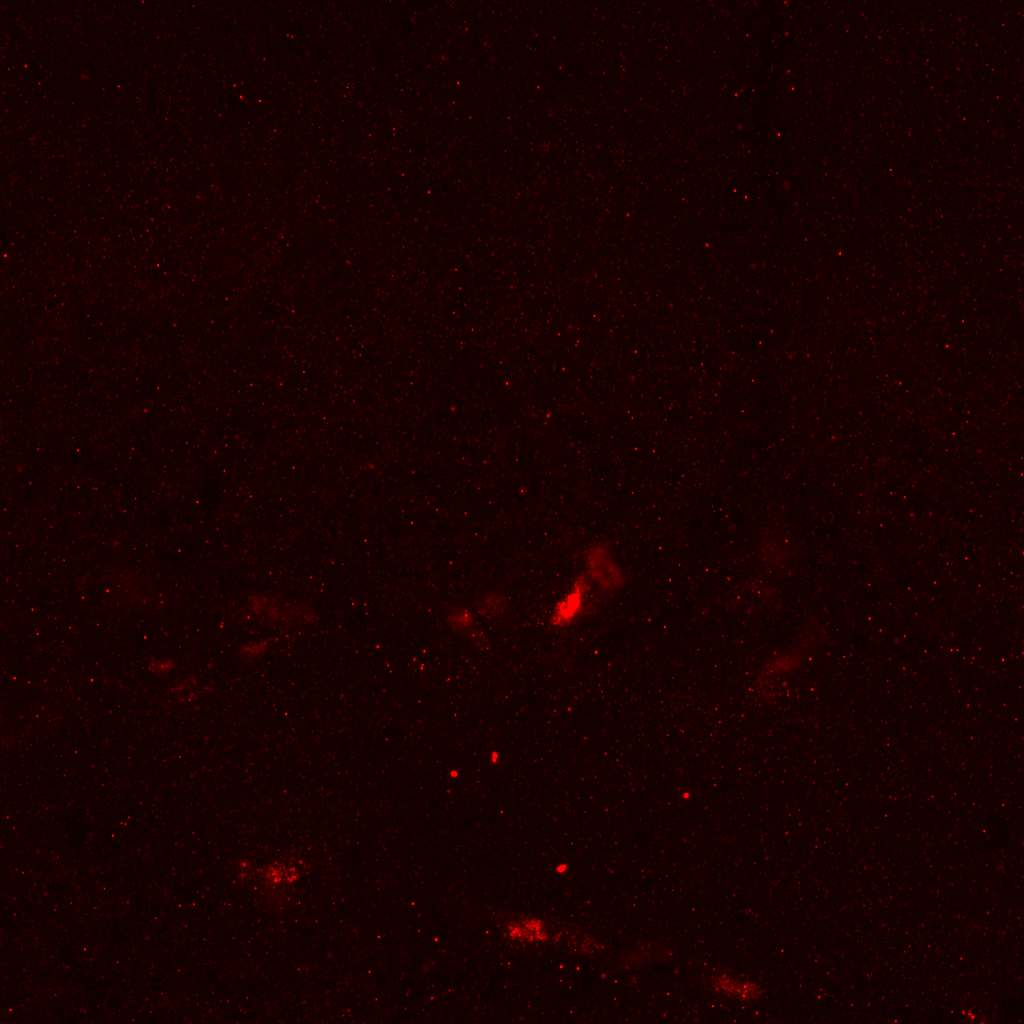

Supplement: Supplementary file 3 — Source data Fig. 1 [file 44319_2024_205_MOESM3_ESM.zip › Source_data_Figure1/1Q/Derl1f:f/BrdU.tif]

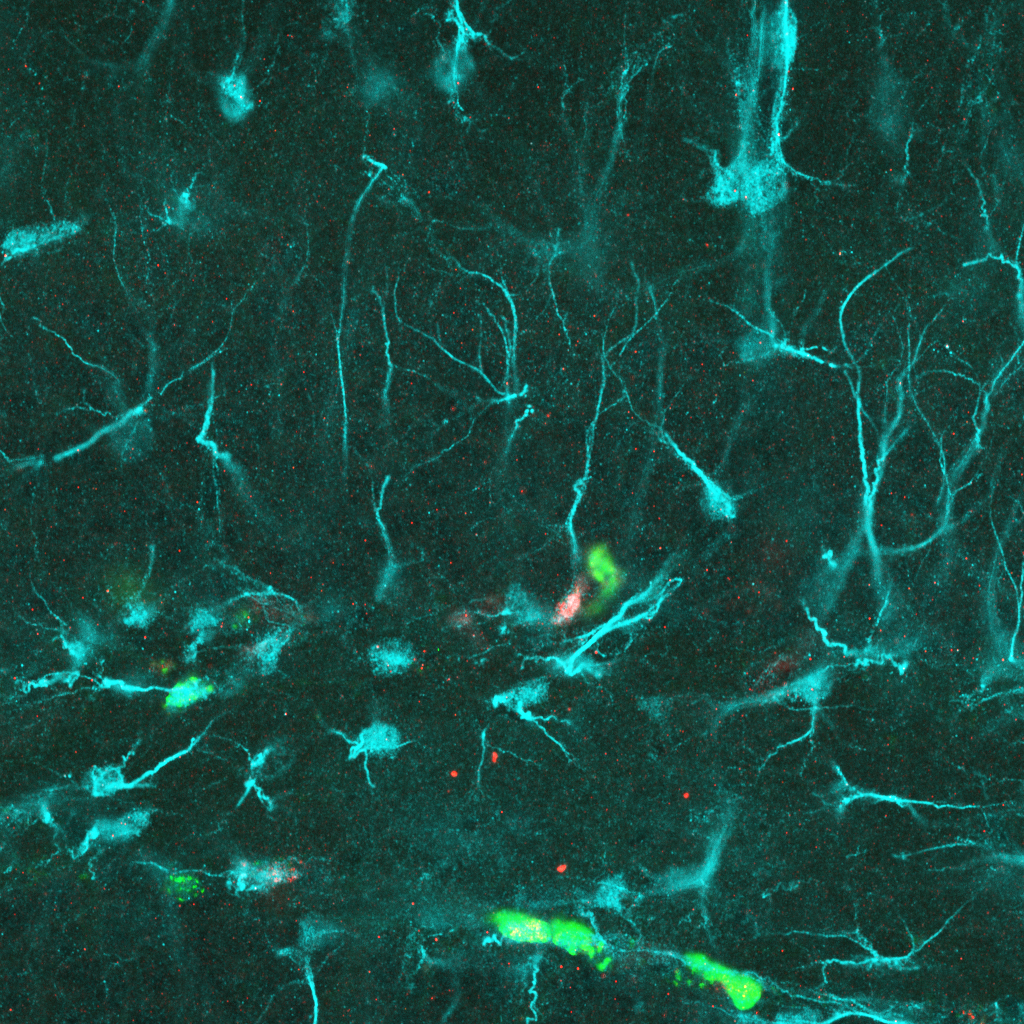

Supplement: Supplementary file 3 — Source data Fig. 1 [file 44319_2024_205_MOESM3_ESM.zip › Source_data_Figure1/1Q/Derl1f:f/Merge.tif]

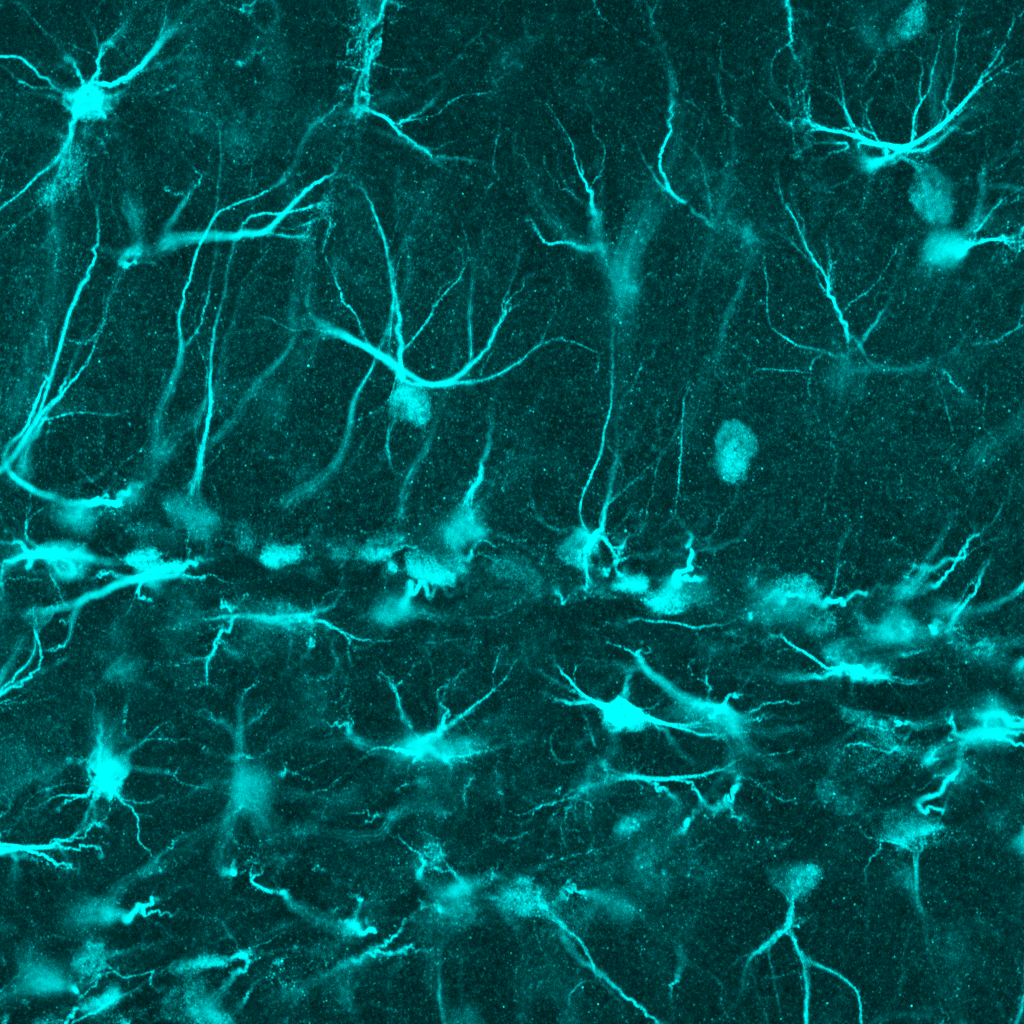

Supplement: Supplementary file 3 — Source data Fig. 1 [file 44319_2024_205_MOESM3_ESM.zip › Source_data_Figure1/1Q/Derl1NesCre/GFAPsox2.tif]

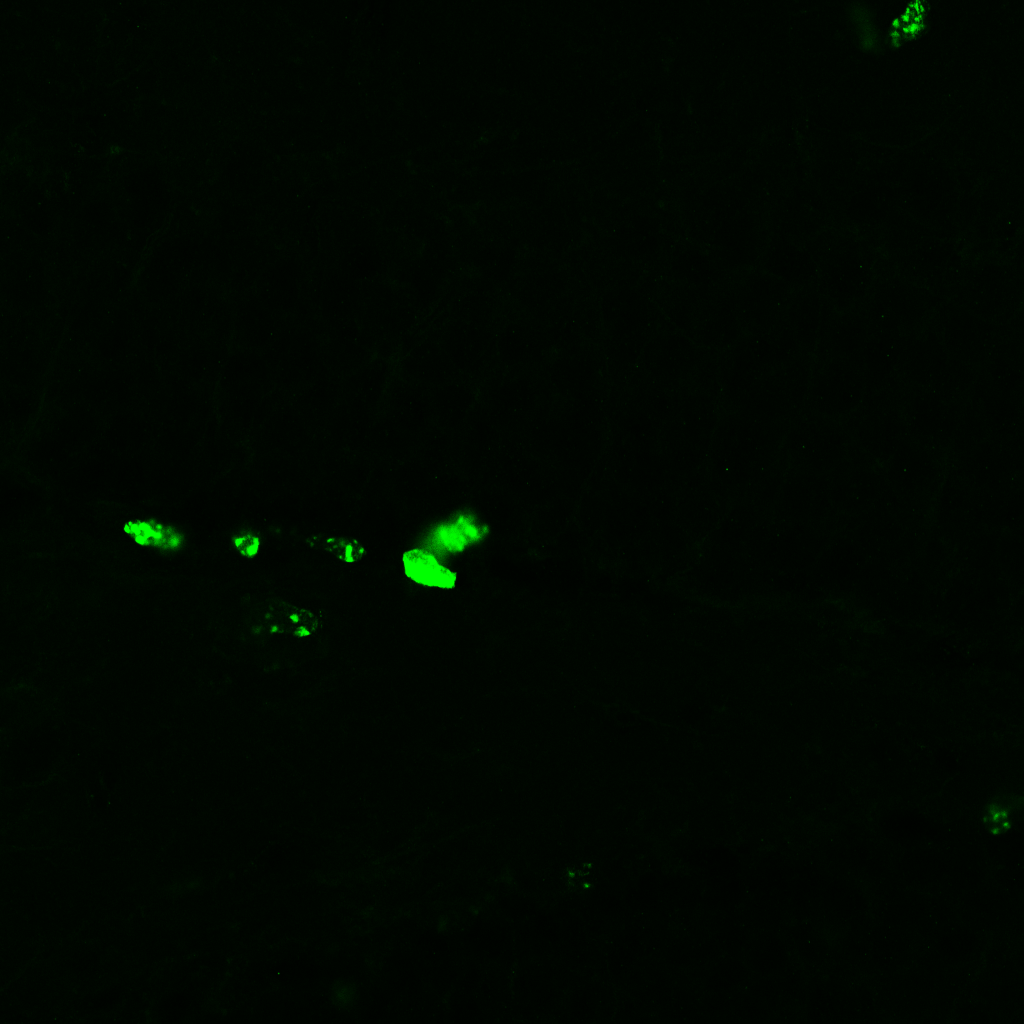

Supplement: Supplementary file 3 — Source data Fig. 1 [file 44319_2024_205_MOESM3_ESM.zip › Source_data_Figure1/1Q/Derl1NesCre/Ki67.tif]

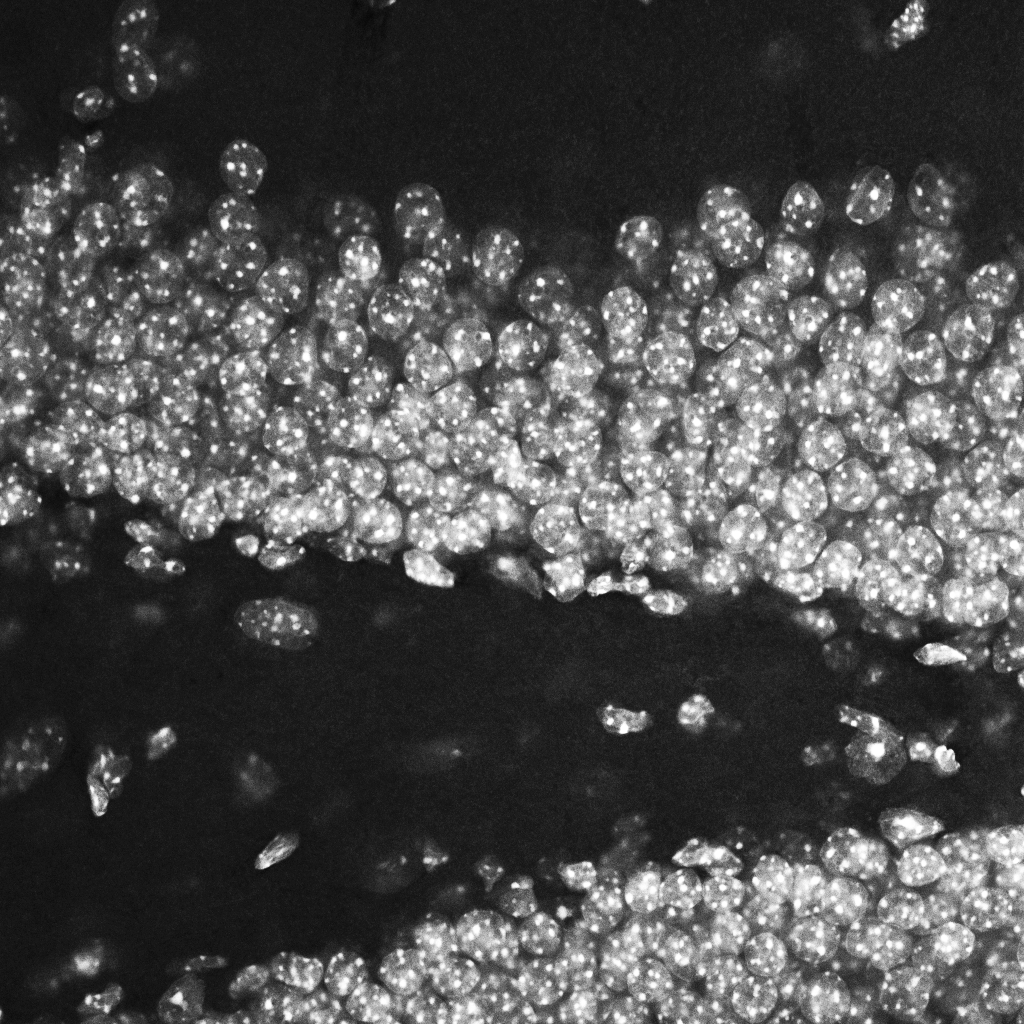

Supplement: Supplementary file 3 — Source data Fig. 1 [file 44319_2024_205_MOESM3_ESM.zip › Source_data_Figure1/1Q/Derl1NesCre/Hoechst.tif]

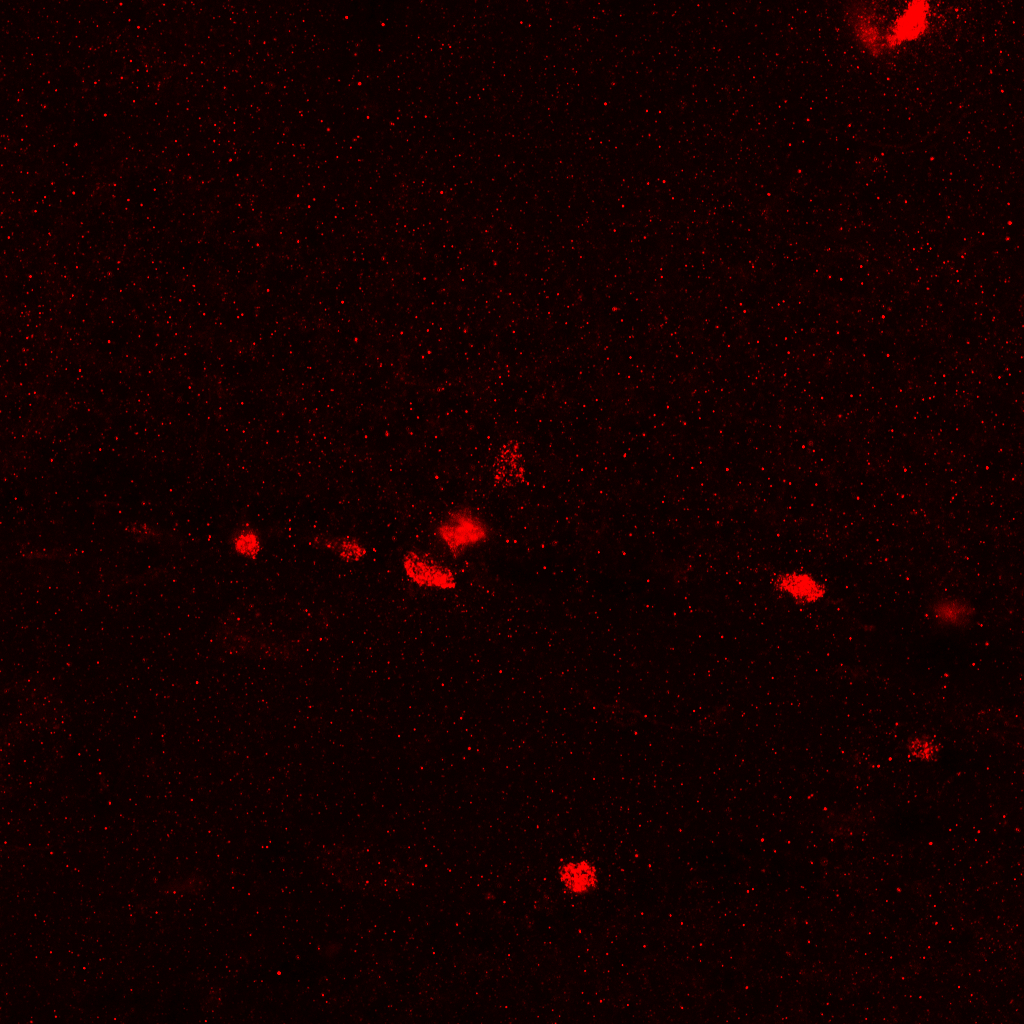

Supplement: Supplementary file 3 — Source data Fig. 1 [file 44319_2024_205_MOESM3_ESM.zip › Source_data_Figure1/1Q/Derl1NesCre/BrdU.tif]

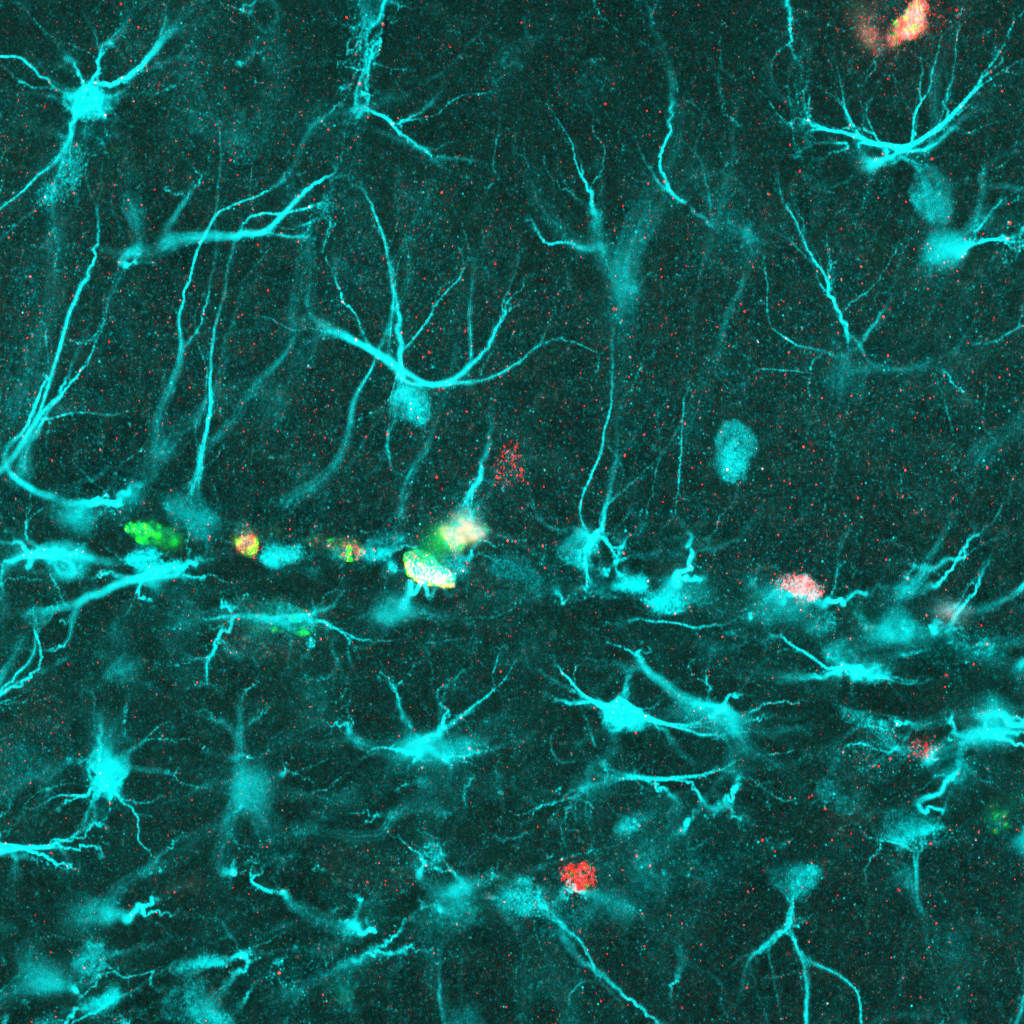

Supplement: Supplementary file 3 — Source data Fig. 1 [file 44319_2024_205_MOESM3_ESM.zip › Source_data_Figure1/1Q/Derl1NesCre/Merge.tif]

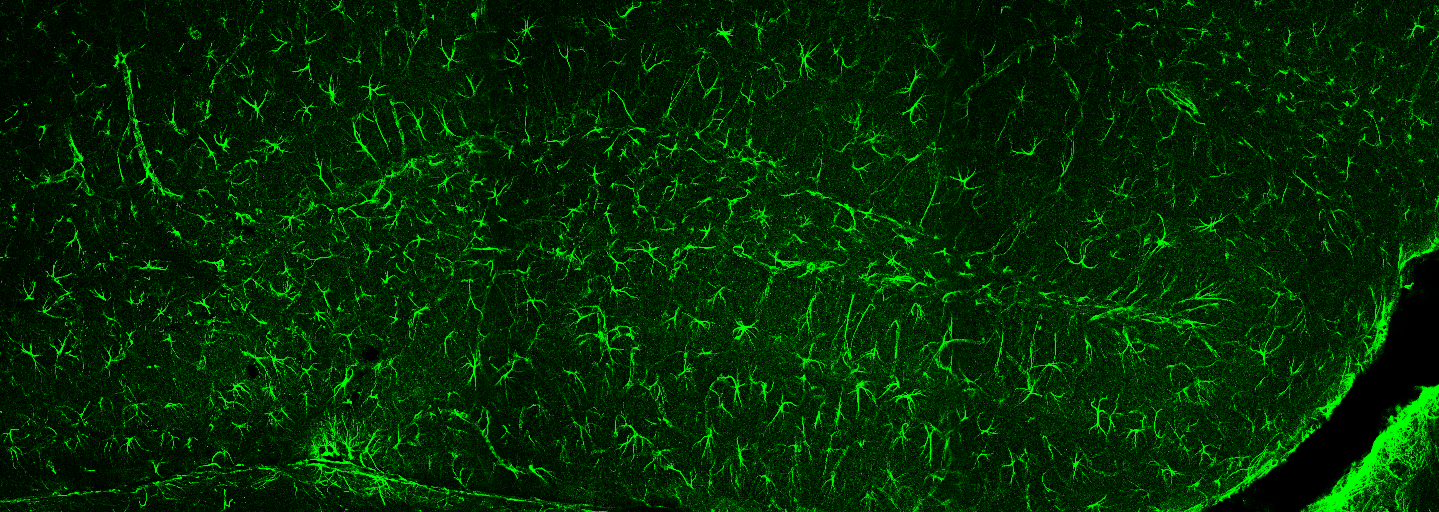

Supplement: Supplementary file 4 — Source data Fig. 2 [file 44319_2024_205_MOESM4_ESM.zip › Source_data_Figure2/2C/Derl1f:f/GFAP.tif]

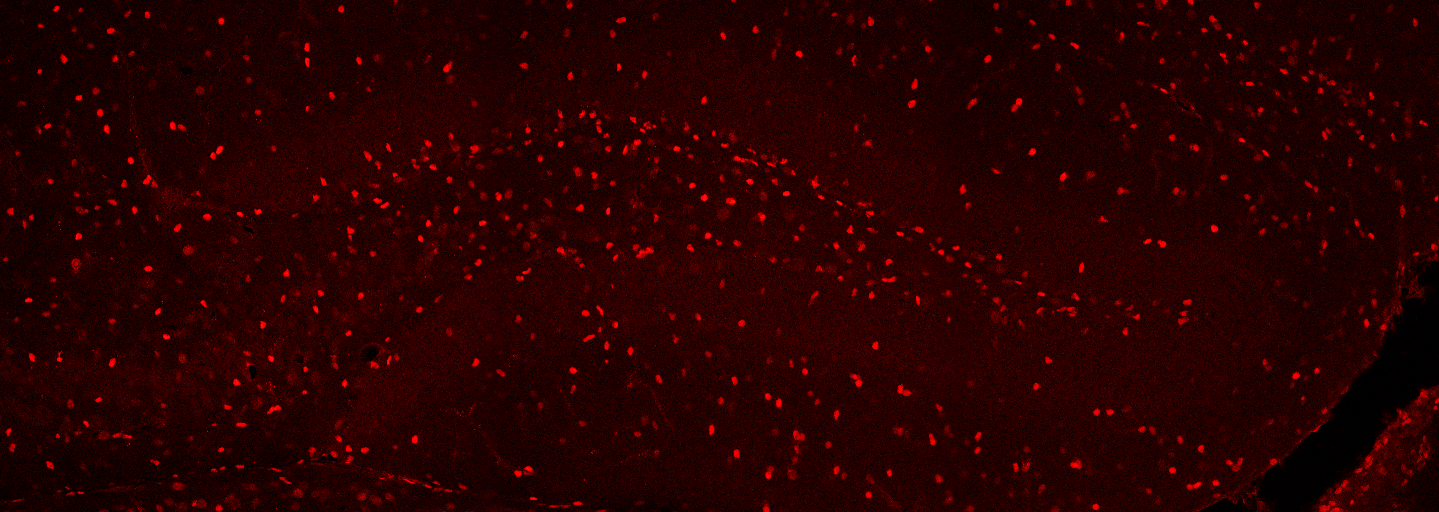

Supplement: Supplementary file 4 — Source data Fig. 2 [file 44319_2024_205_MOESM4_ESM.zip › Source_data_Figure2/2C/Derl1f:f/Sox2.tif]

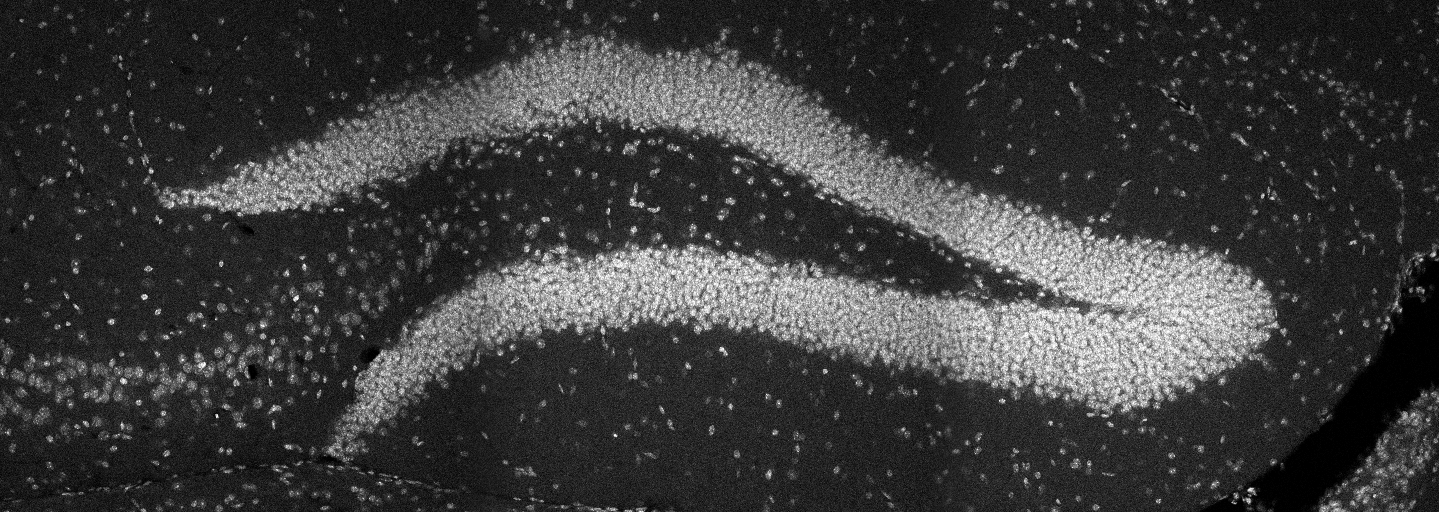

Supplement: Supplementary file 4 — Source data Fig. 2 [file 44319_2024_205_MOESM4_ESM.zip › Source_data_Figure2/2C/Derl1f:f/Hoechst.tif]

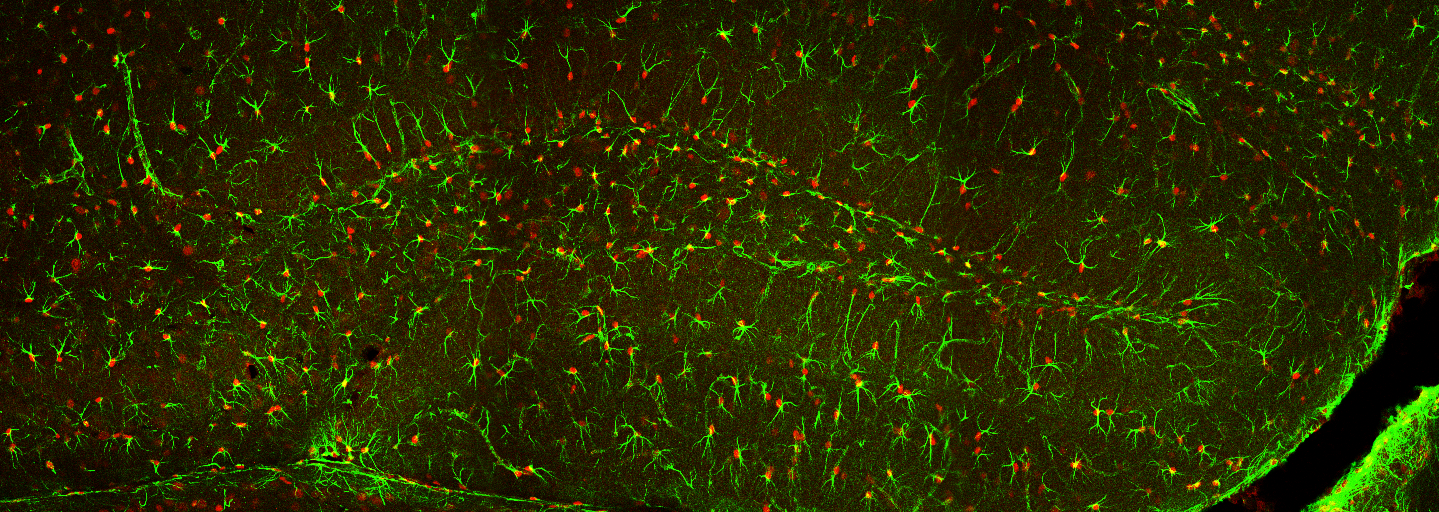

Supplement: Supplementary file 4 — Source data Fig. 2 [file 44319_2024_205_MOESM4_ESM.zip › Source_data_Figure2/2C/Derl1f:f/Merge.tif]

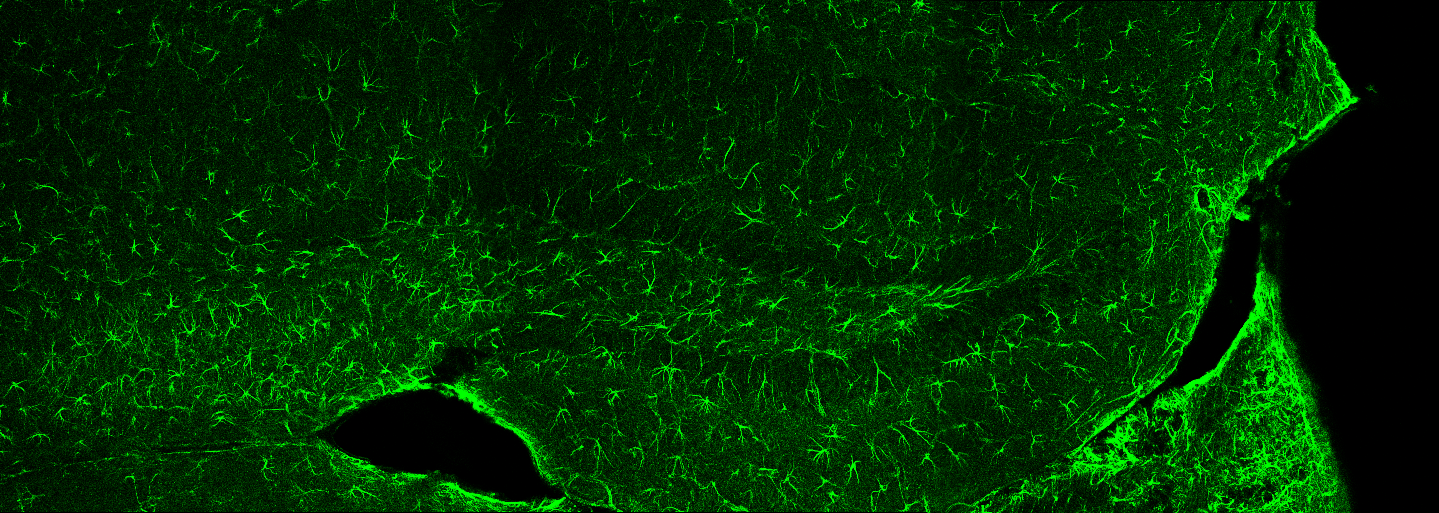

Supplement: Supplementary file 4 — Source data Fig. 2 [file 44319_2024_205_MOESM4_ESM.zip › Source_data_Figure2/2C/Derl1NesCre/GFAP.tif]

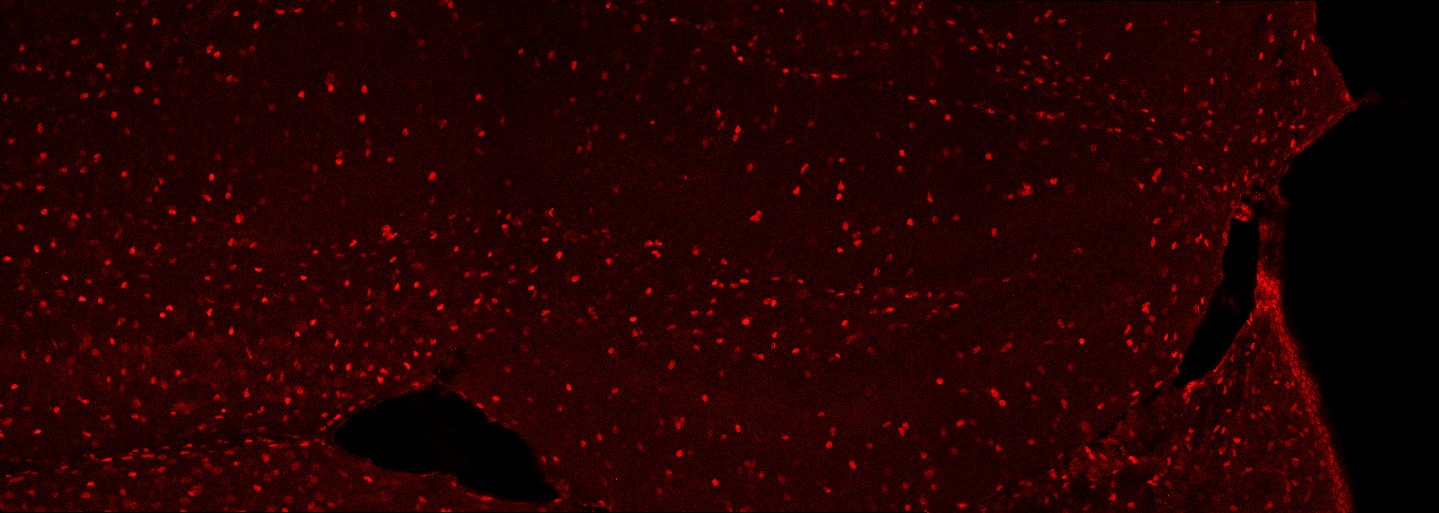

Supplement: Supplementary file 4 — Source data Fig. 2 [file 44319_2024_205_MOESM4_ESM.zip › Source_data_Figure2/2C/Derl1NesCre/Sox2.tif]

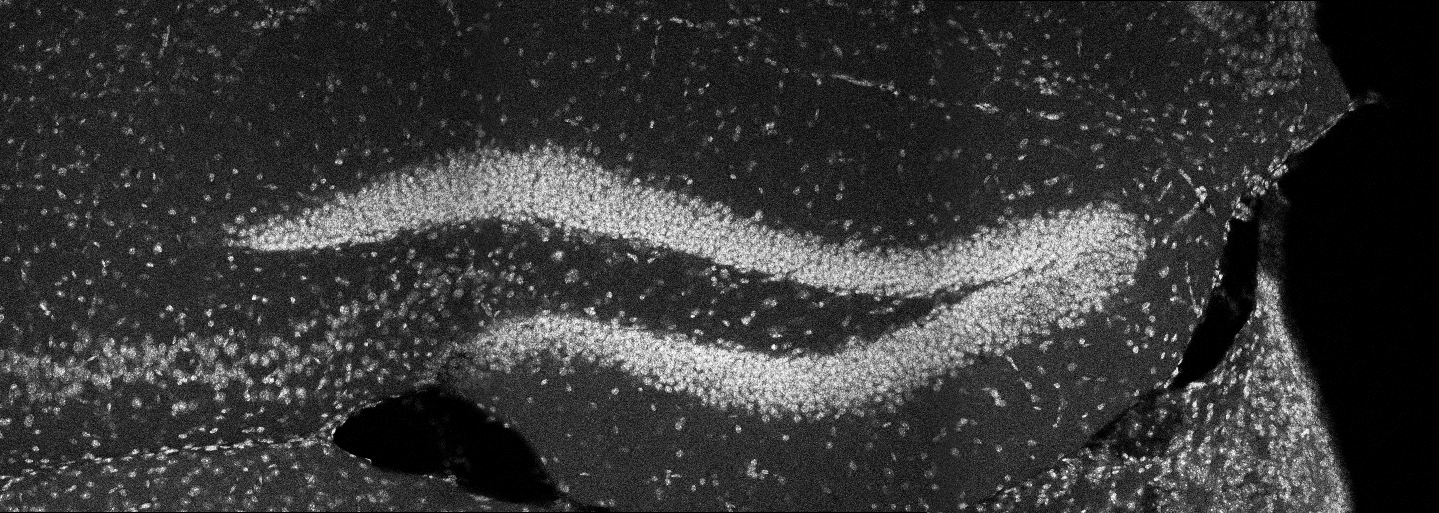

Supplement: Supplementary file 4 — Source data Fig. 2 [file 44319_2024_205_MOESM4_ESM.zip › Source_data_Figure2/2C/Derl1NesCre/Hoechst.tif]

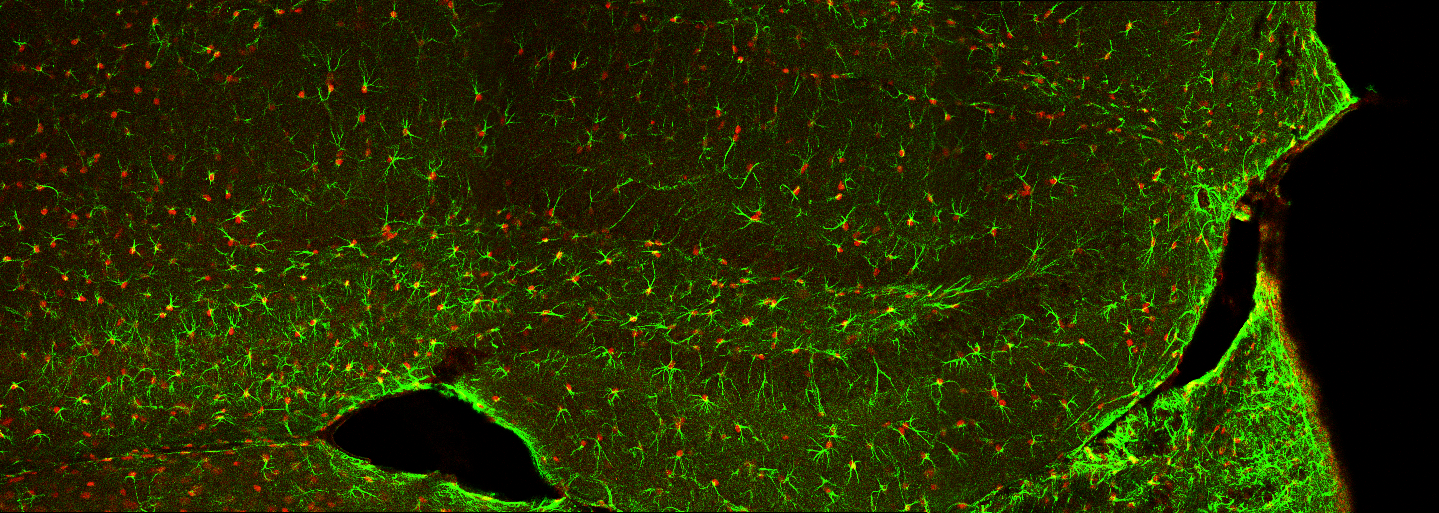

Supplement: Supplementary file 4 — Source data Fig. 2 [file 44319_2024_205_MOESM4_ESM.zip › Source_data_Figure2/2C/Derl1NesCre/Merge.tif]

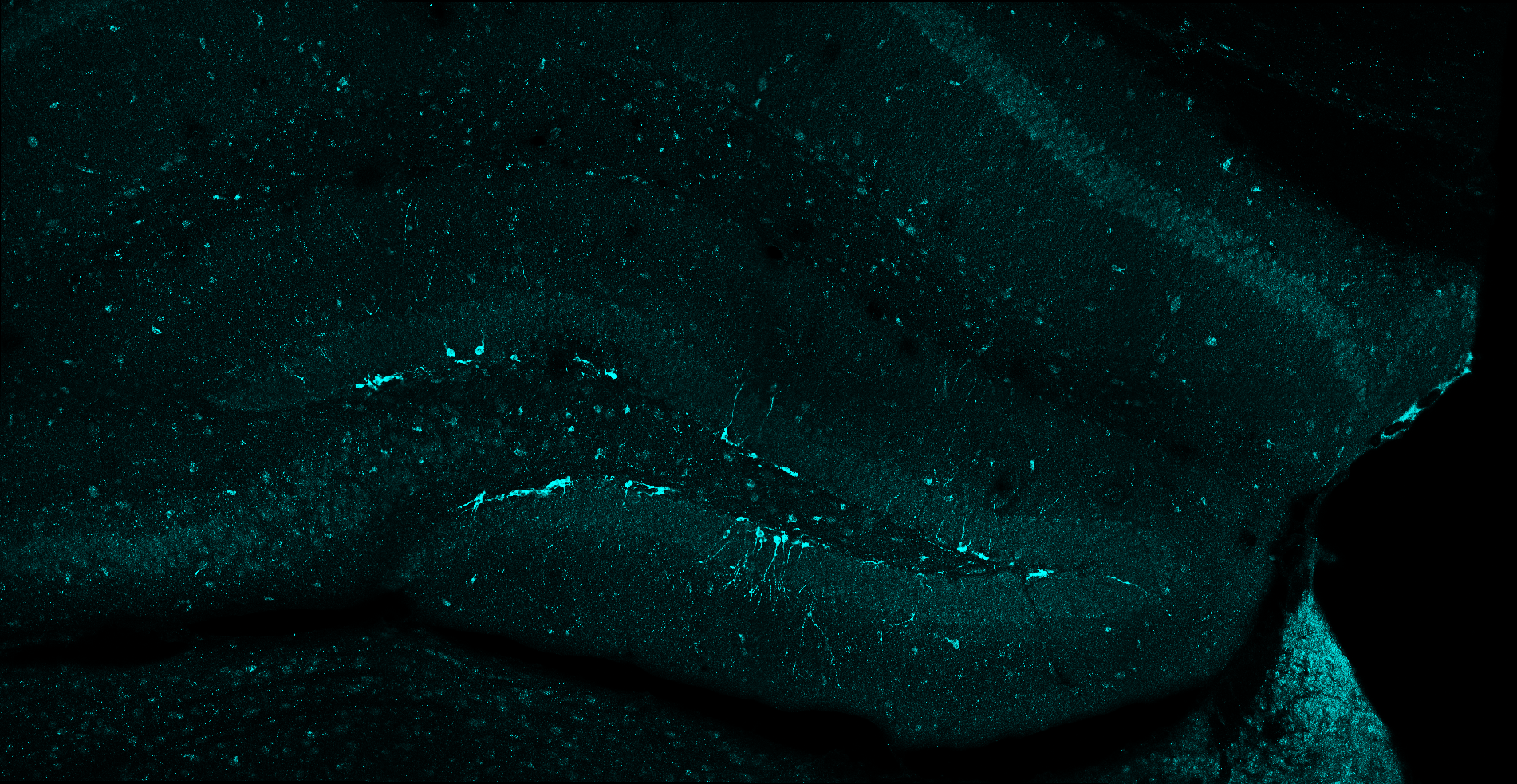

Supplement: Supplementary file 4 — Source data Fig. 2 [file 44319_2024_205_MOESM4_ESM.zip › Source_data_Figure2/2E/Derl1f:f/DCX.tif]

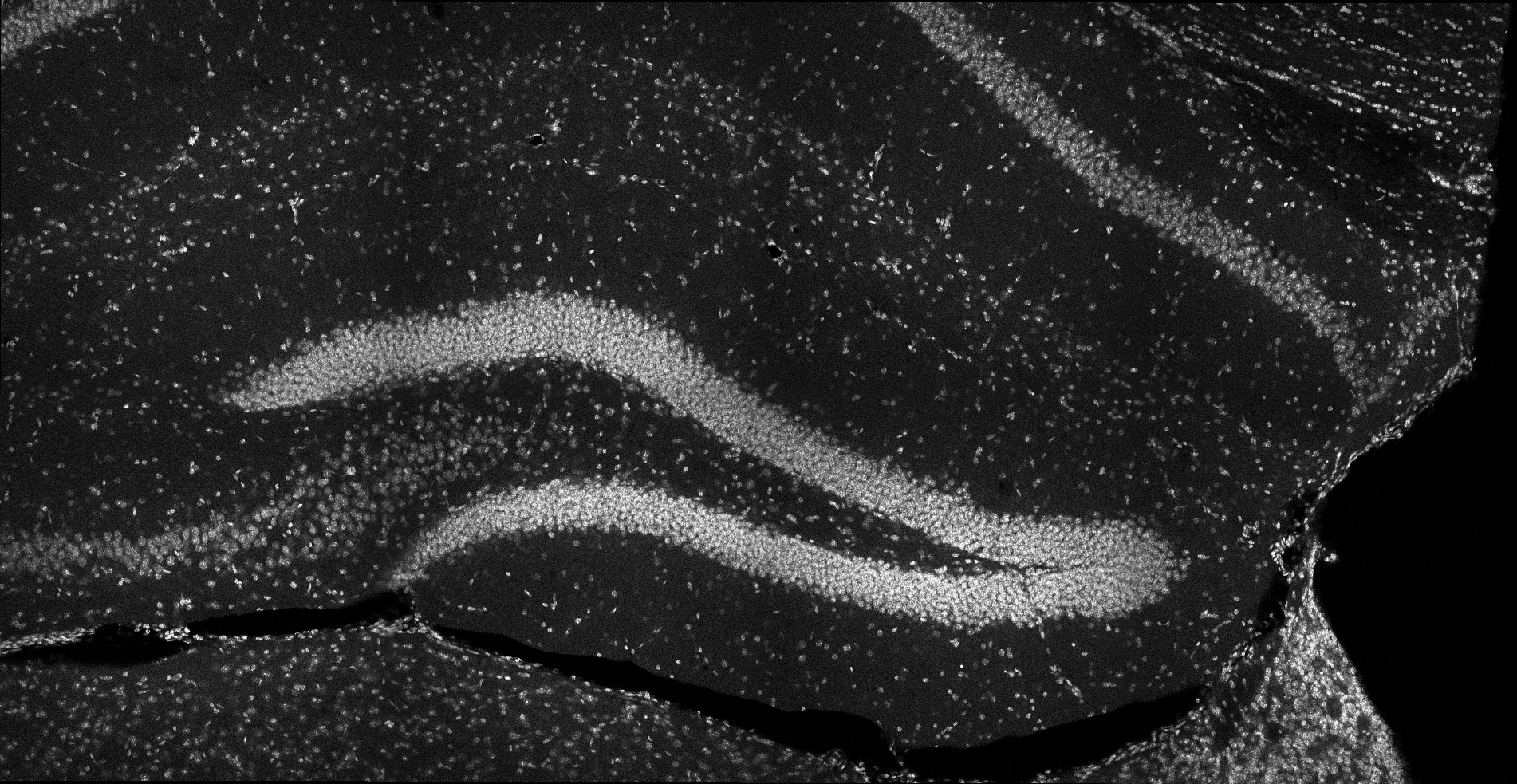

Supplement: Supplementary file 4 — Source data Fig. 2 [file 44319_2024_205_MOESM4_ESM.zip › Source_data_Figure2/2E/Derl1f:f/Hoechst.tif]

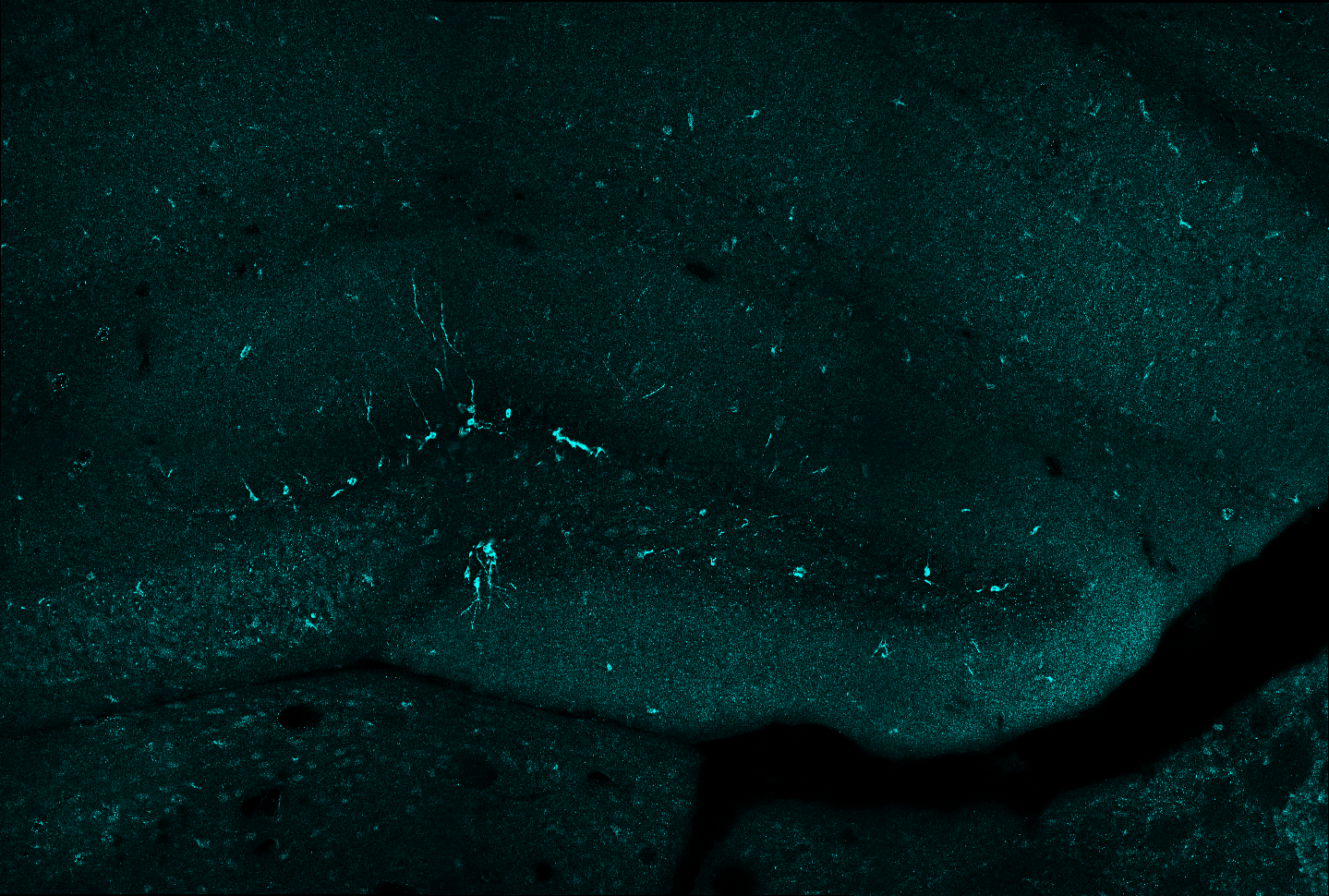

Supplement: Supplementary file 4 — Source data Fig. 2 [file 44319_2024_205_MOESM4_ESM.zip › Source_data_Figure2/2E/Derl1NesCre/DCX.tif]

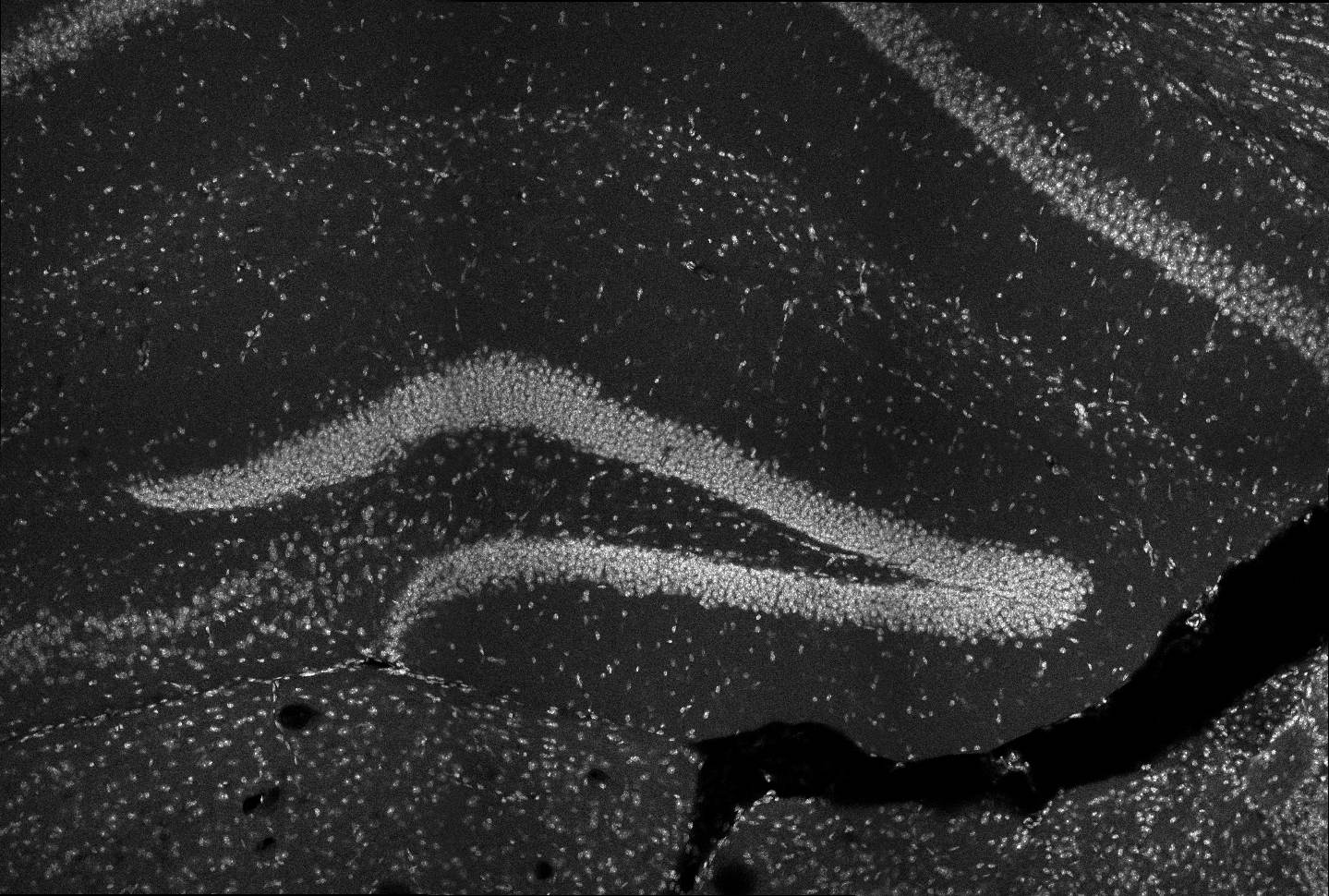

Supplement: Supplementary file 4 — Source data Fig. 2 [file 44319_2024_205_MOESM4_ESM.zip › Source_data_Figure2/2E/Derl1NesCre/Hoechst.tif]

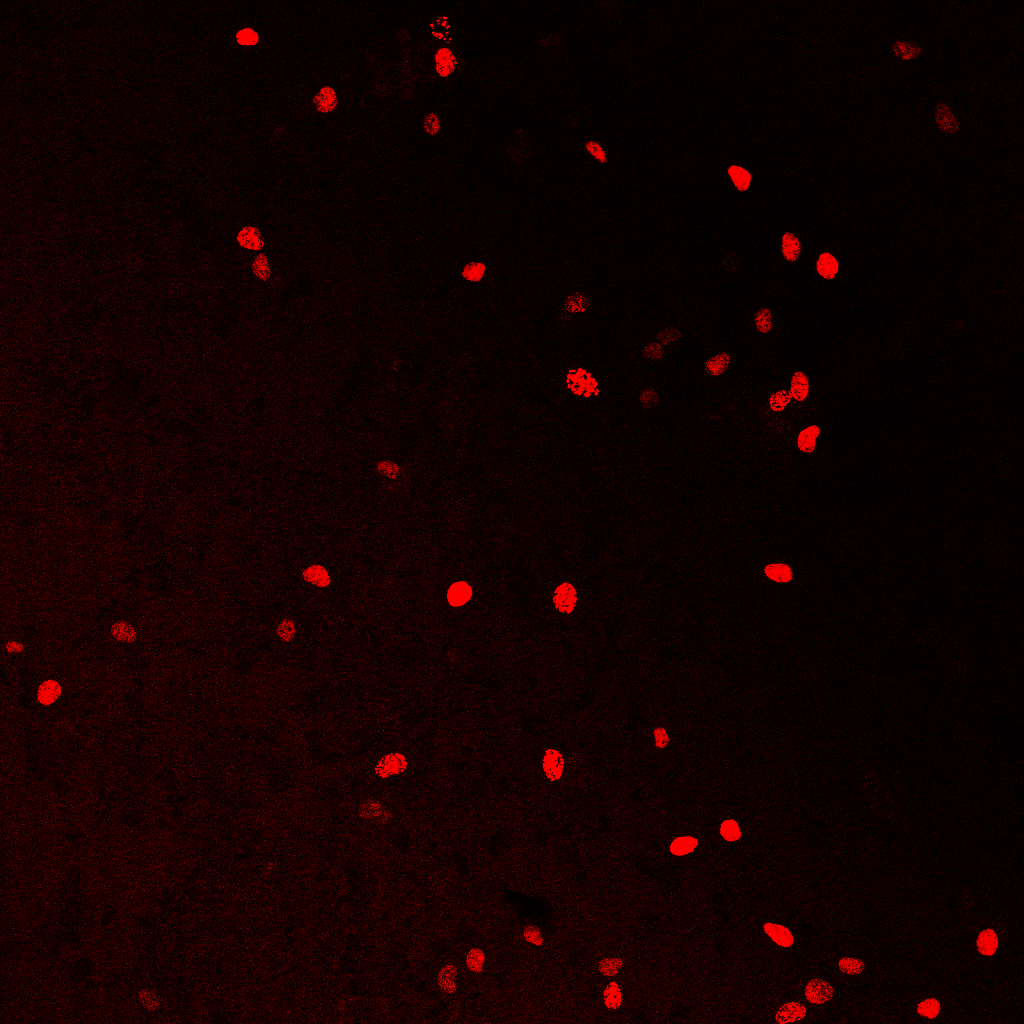

Supplement: Supplementary file 5 — Source data Fig. 3 [file 44319_2024_205_MOESM5_ESM.zip › Source_data_Figure3/3B/BMP4/siControl/EdU.tif]

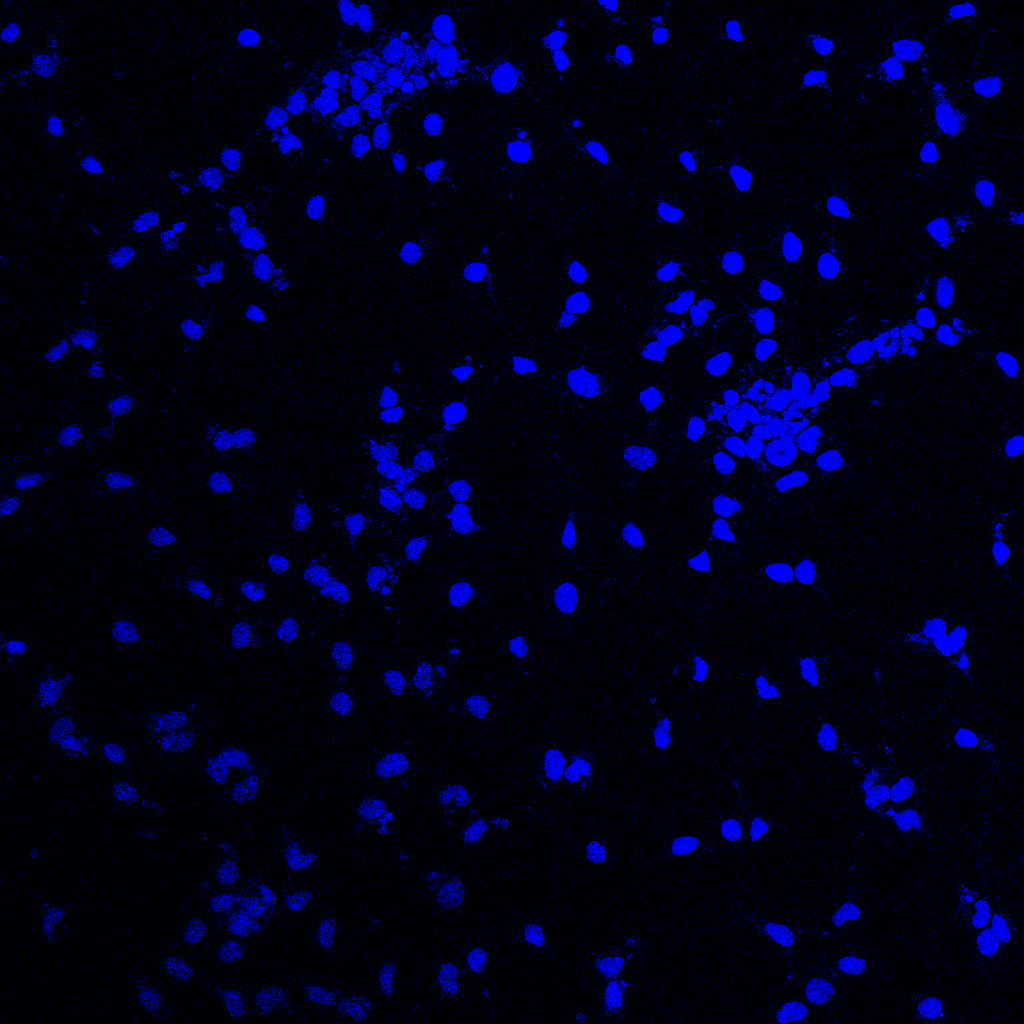

Supplement: Supplementary file 5 — Source data Fig. 3 [file 44319_2024_205_MOESM5_ESM.zip › Source_data_Figure3/3B/BMP4/siControl/Hoechst.tif]

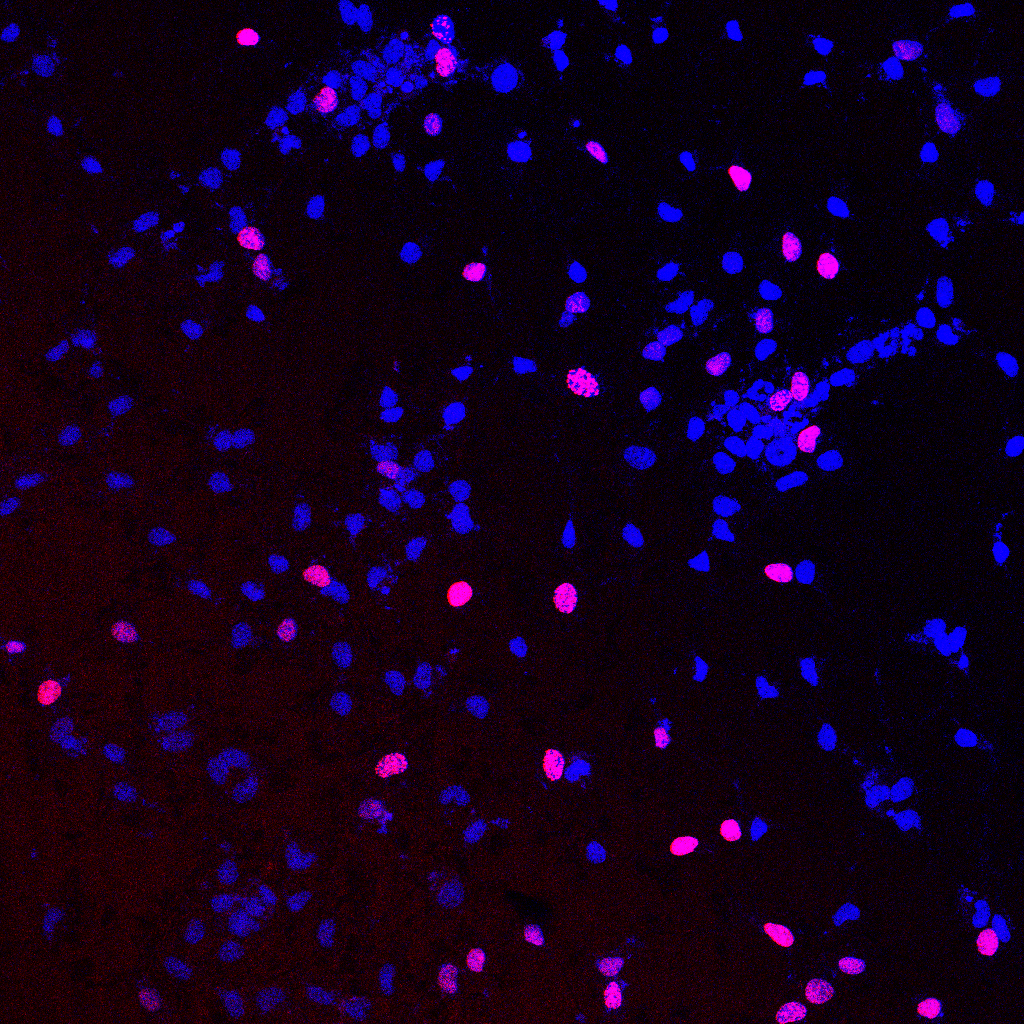

Supplement: Supplementary file 5 — Source data Fig. 3 [file 44319_2024_205_MOESM5_ESM.zip › Source_data_Figure3/3B/BMP4/siControl/Merge.tif]

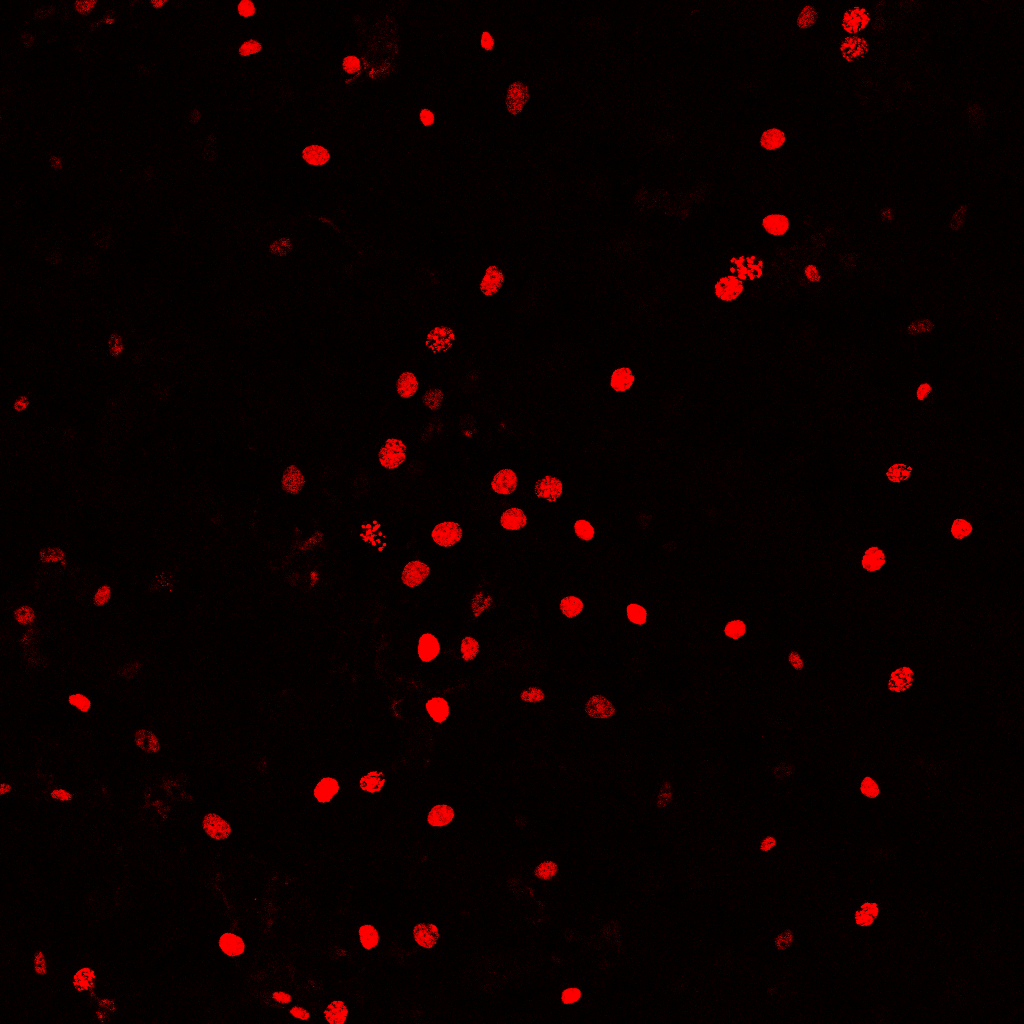

Supplement: Supplementary file 5 — Source data Fig. 3 [file 44319_2024_205_MOESM5_ESM.zip › Source_data_Figure3/3B/BMP4/siDerl1/EdU.tif]

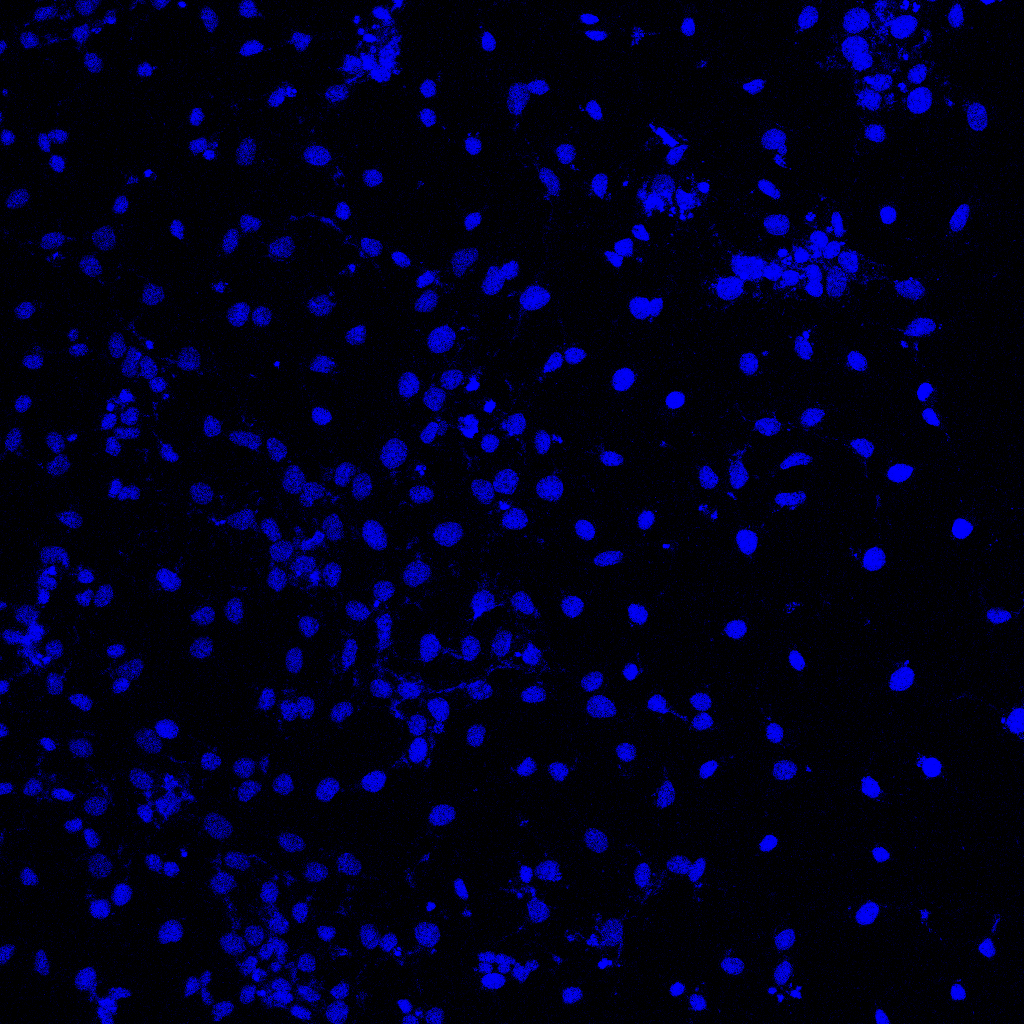

Supplement: Supplementary file 5 — Source data Fig. 3 [file 44319_2024_205_MOESM5_ESM.zip › Source_data_Figure3/3B/BMP4/siDerl1/Hoechst.tif]

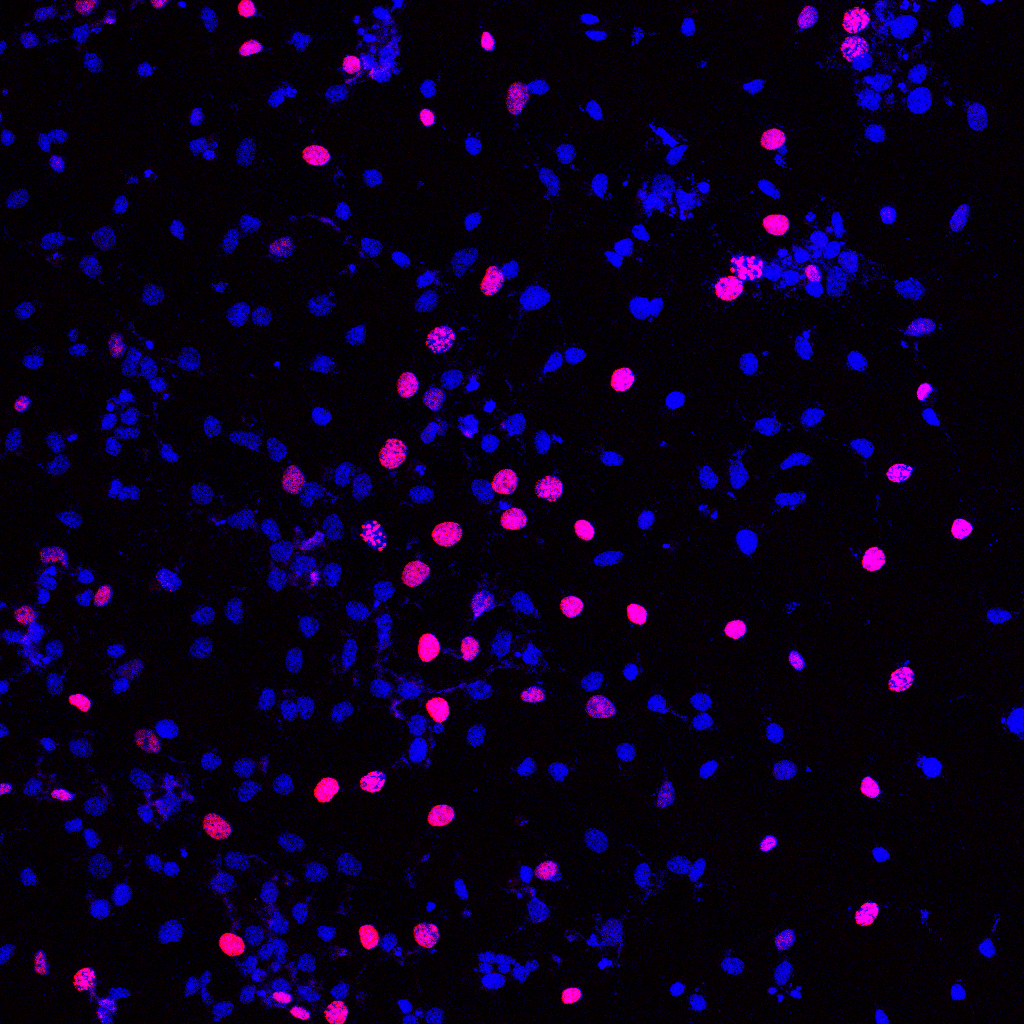

Supplement: Supplementary file 5 — Source data Fig. 3 [file 44319_2024_205_MOESM5_ESM.zip › Source_data_Figure3/3B/BMP4/siDerl1/Merge.tif]

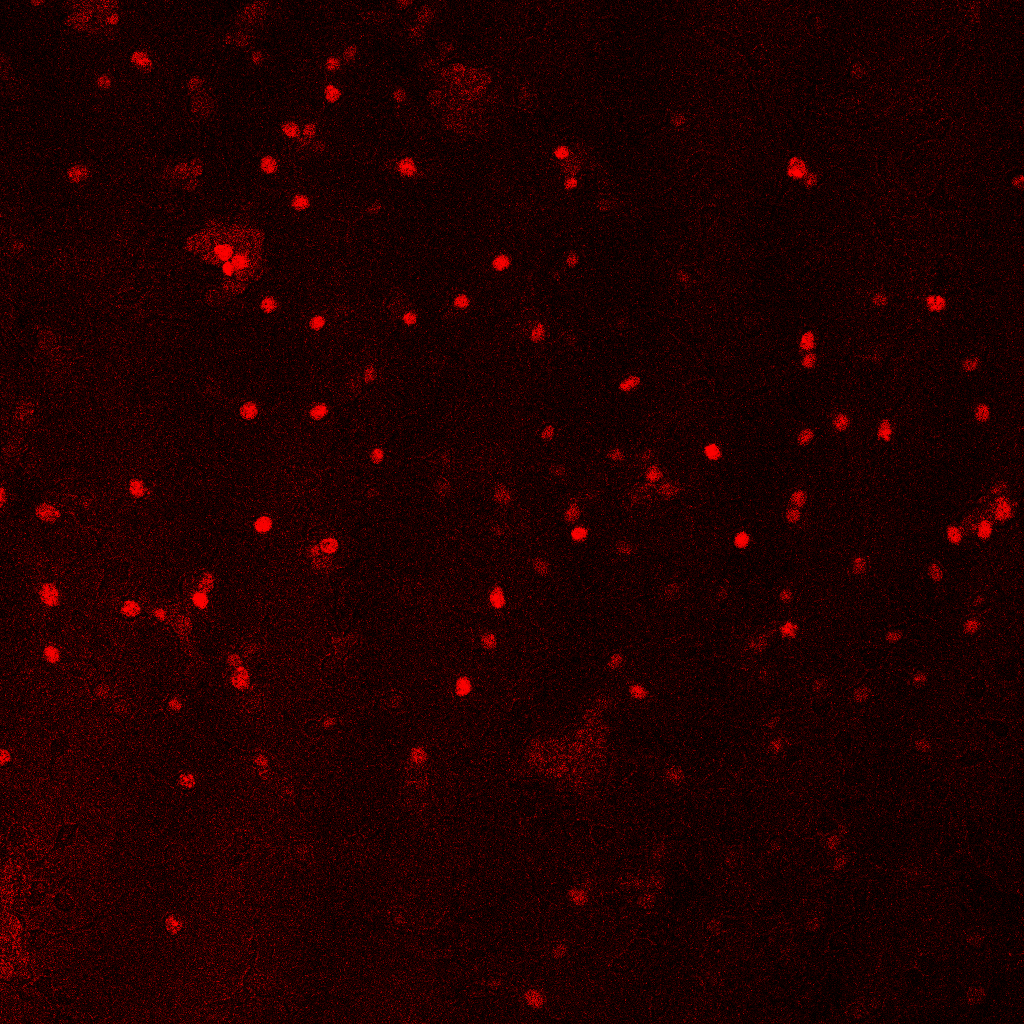

Supplement: Supplementary file 5 — Source data Fig. 3 [file 44319_2024_205_MOESM5_ESM.zip › Source_data_Figure3/3B/Diazepam/siControl/EdU.tif]

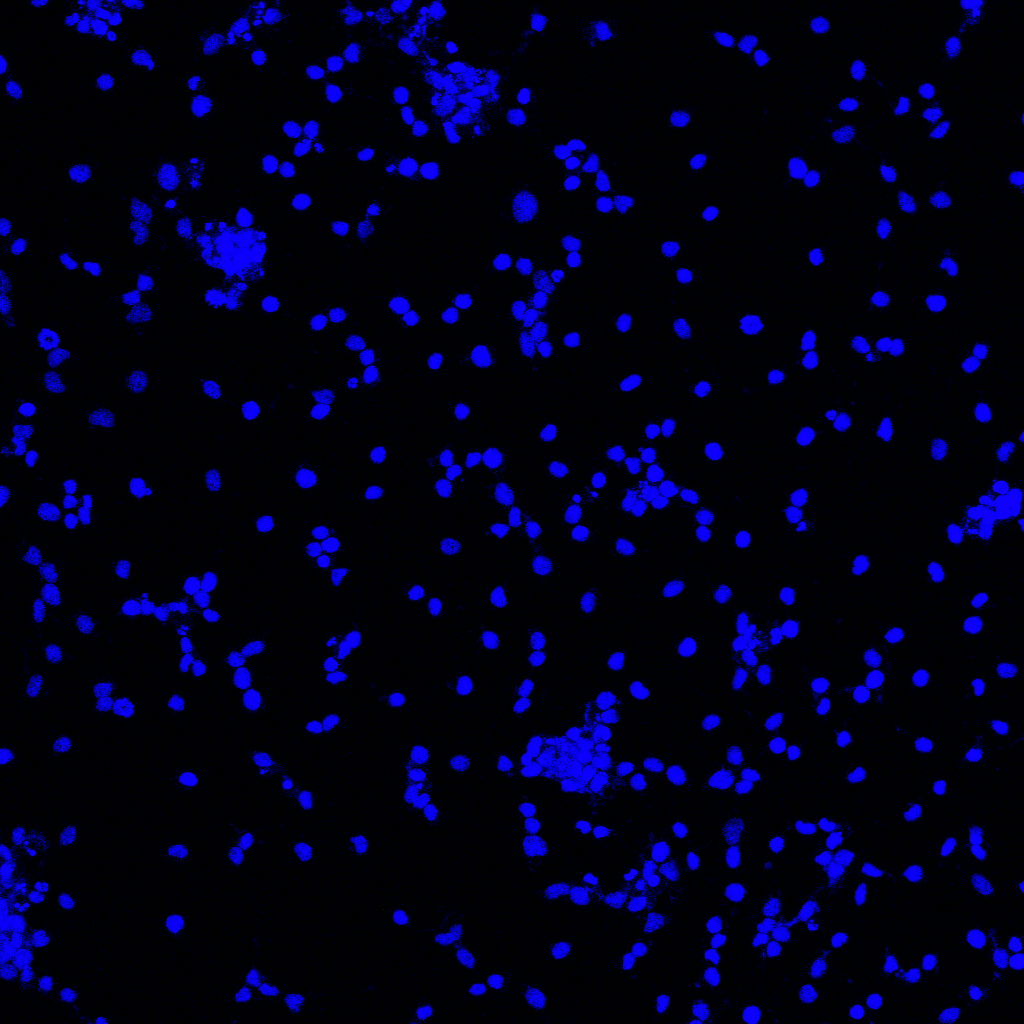

Supplement: Supplementary file 5 — Source data Fig. 3 [file 44319_2024_205_MOESM5_ESM.zip › Source_data_Figure3/3B/Diazepam/siControl/Hoechst.tif]

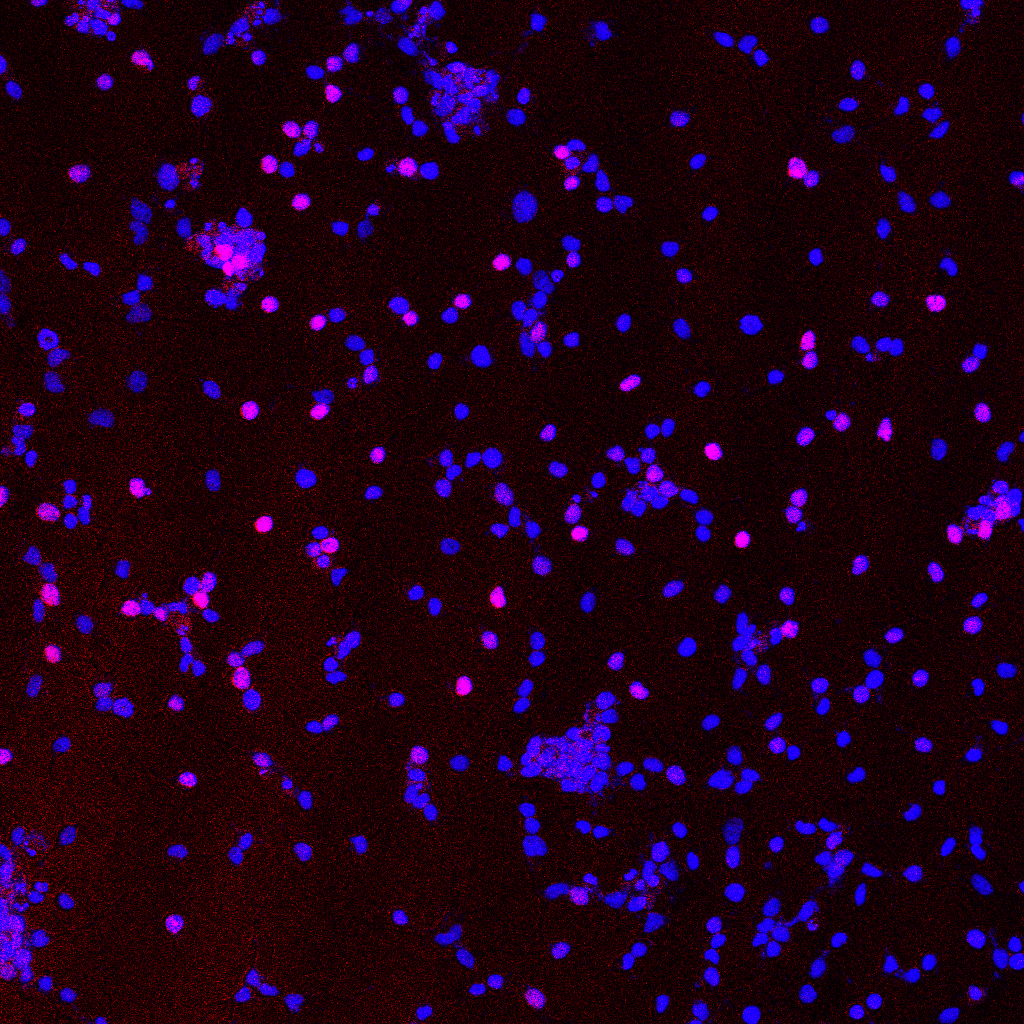

Supplement: Supplementary file 5 — Source data Fig. 3 [file 44319_2024_205_MOESM5_ESM.zip › Source_data_Figure3/3B/Diazepam/siControl/Merge.tif]

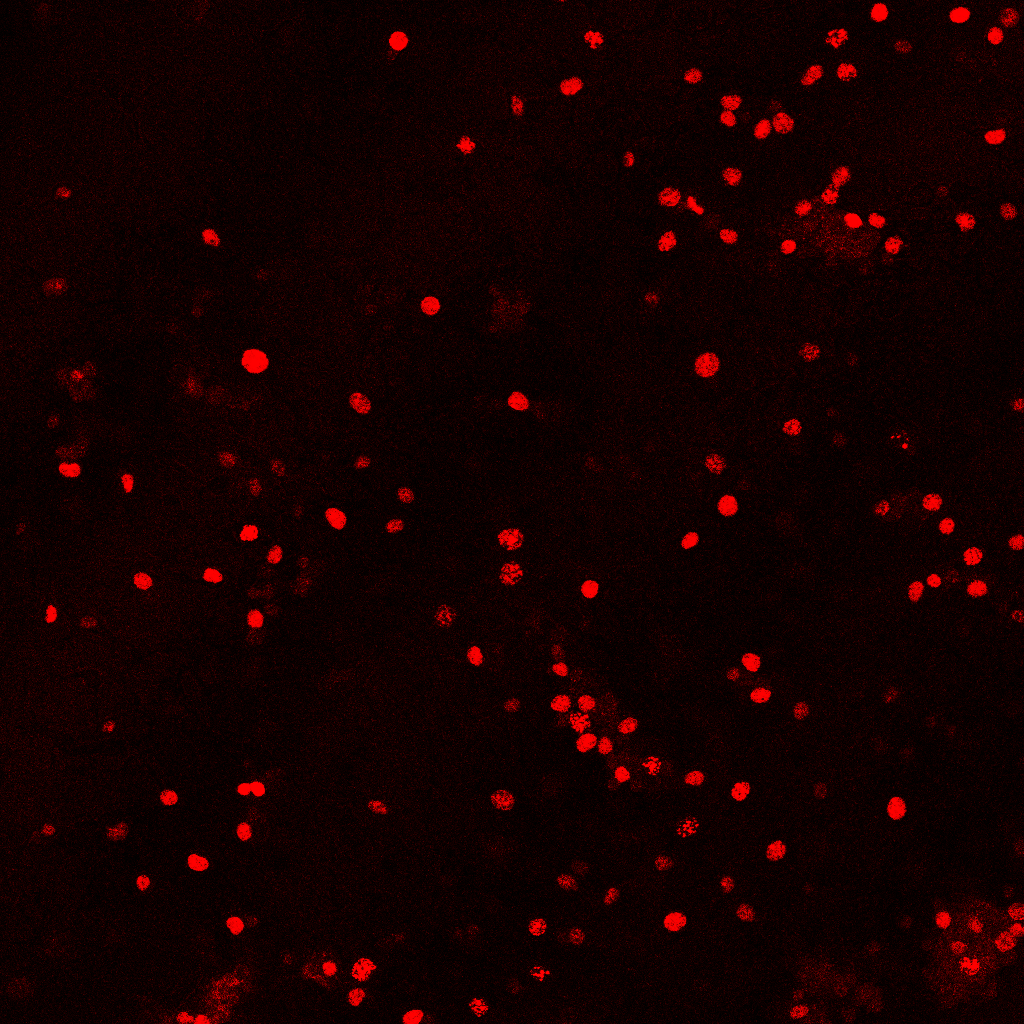

Supplement: Supplementary file 5 — Source data Fig. 3 [file 44319_2024_205_MOESM5_ESM.zip › Source_data_Figure3/3B/Diazepam/siDerl1/EdU.tif]

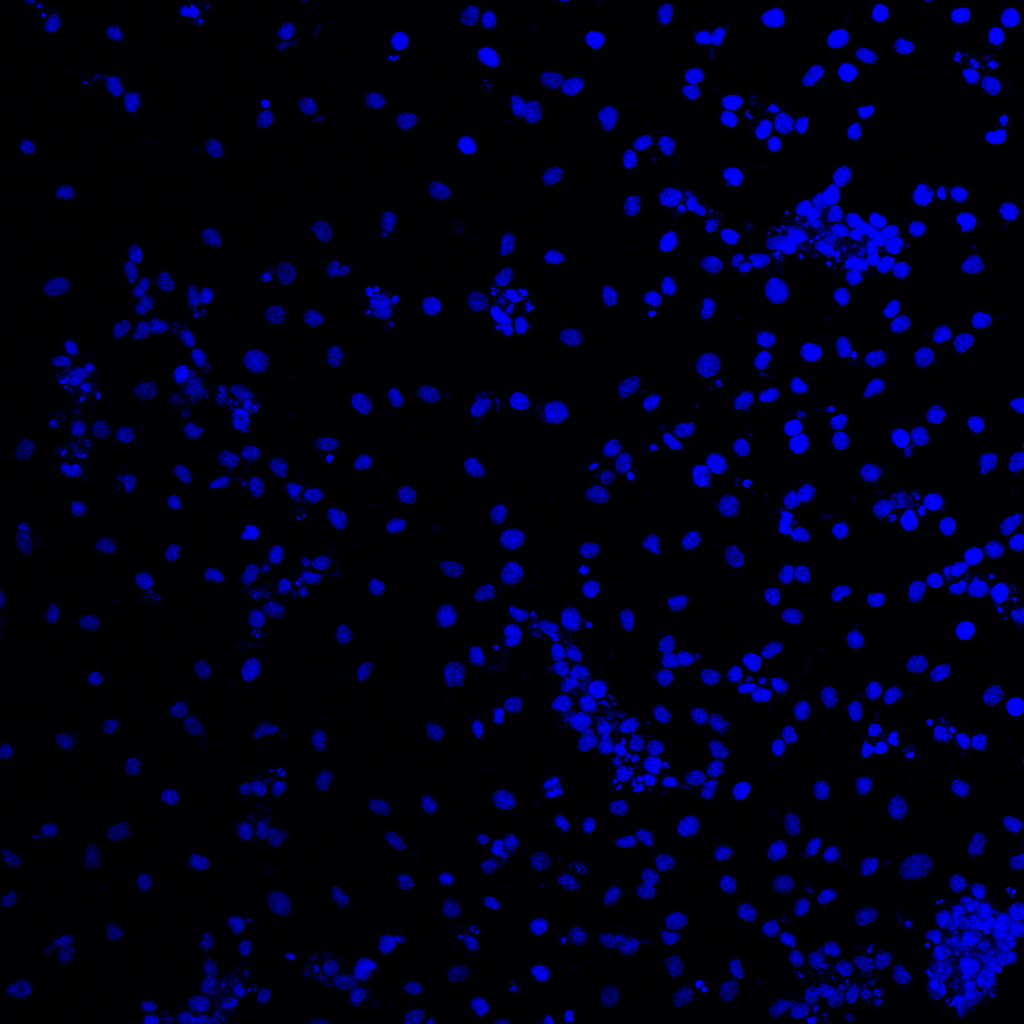

Supplement: Supplementary file 5 — Source data Fig. 3 [file 44319_2024_205_MOESM5_ESM.zip › Source_data_Figure3/3B/Diazepam/siDerl1/Hoechst.tif]

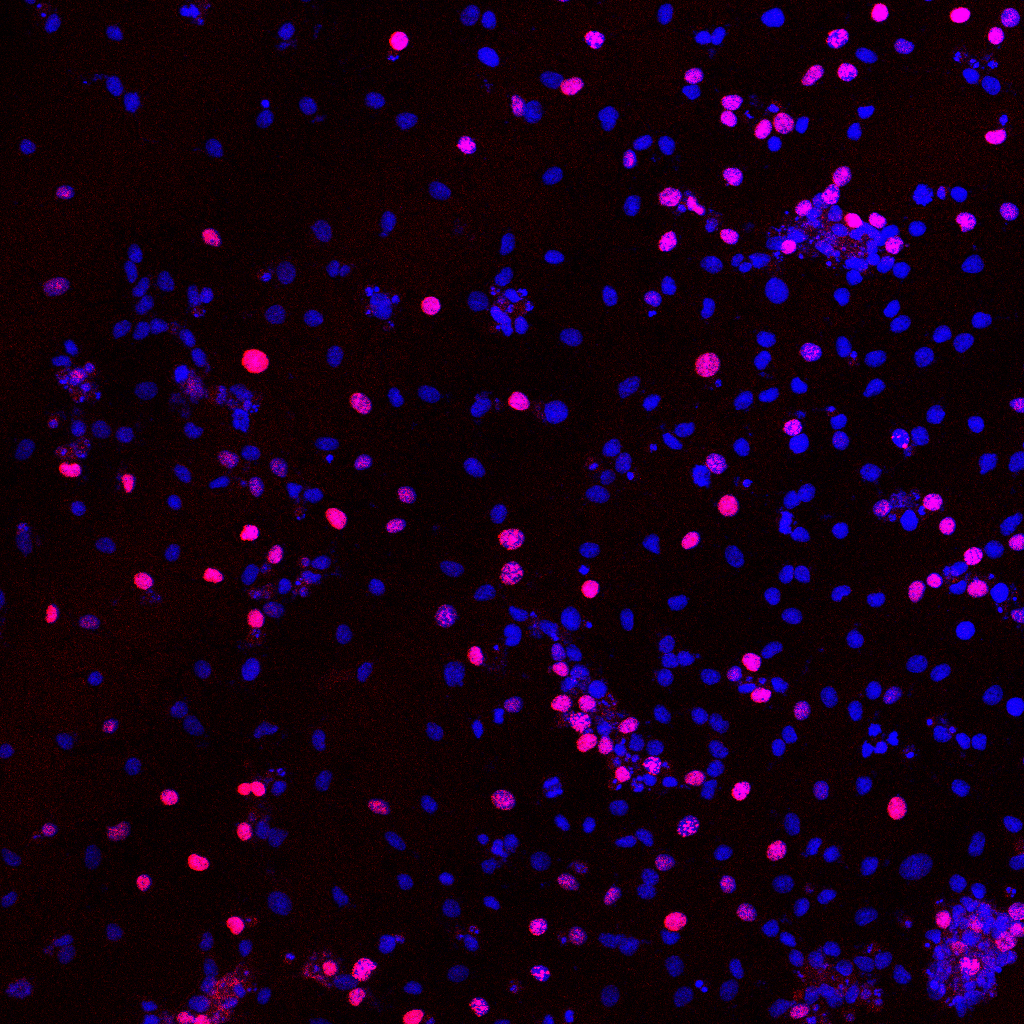

Supplement: Supplementary file 5 — Source data Fig. 3 [file 44319_2024_205_MOESM5_ESM.zip › Source_data_Figure3/3B/Diazepam/siDerl1/Merge.tif]

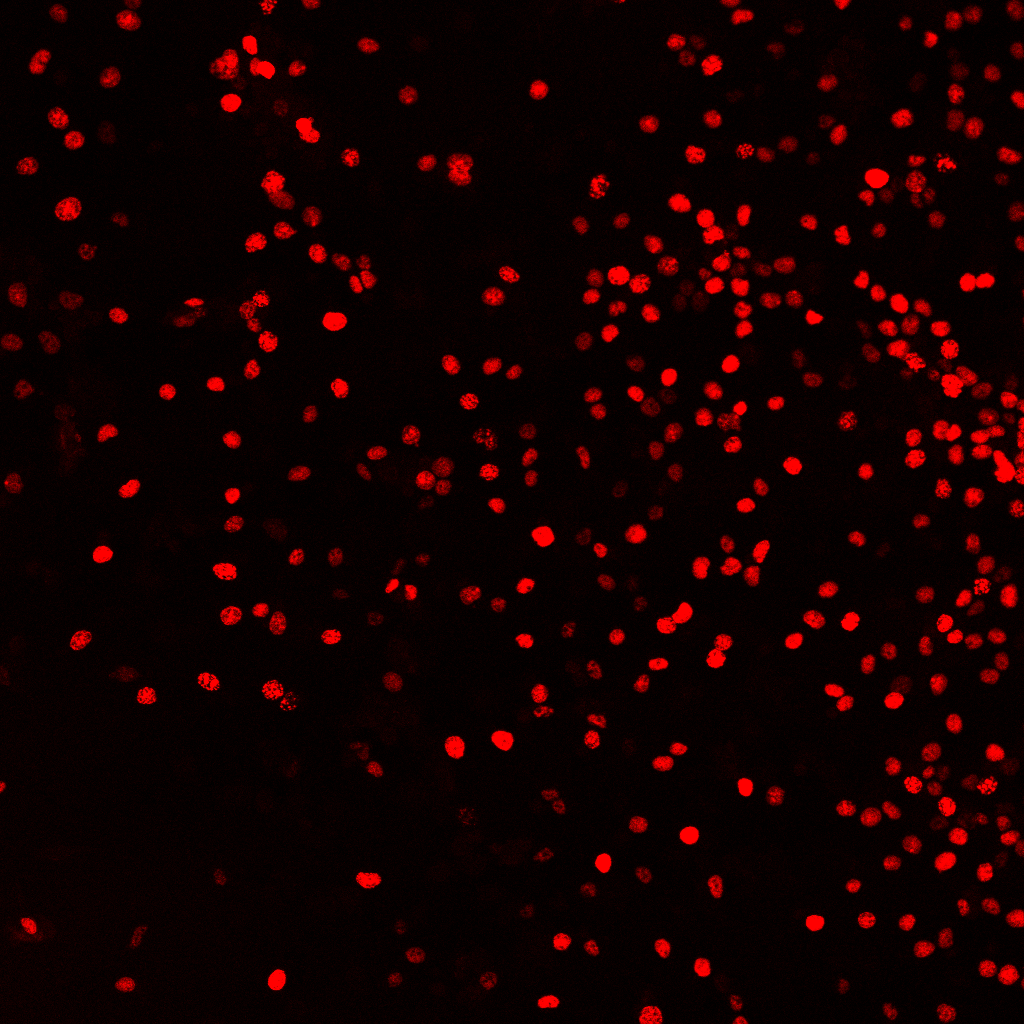

Supplement: Supplementary file 5 — Source data Fig. 3 [file 44319_2024_205_MOESM5_ESM.zip › Source_data_Figure3/3B/Proliferation/siControl/EdU.tif]

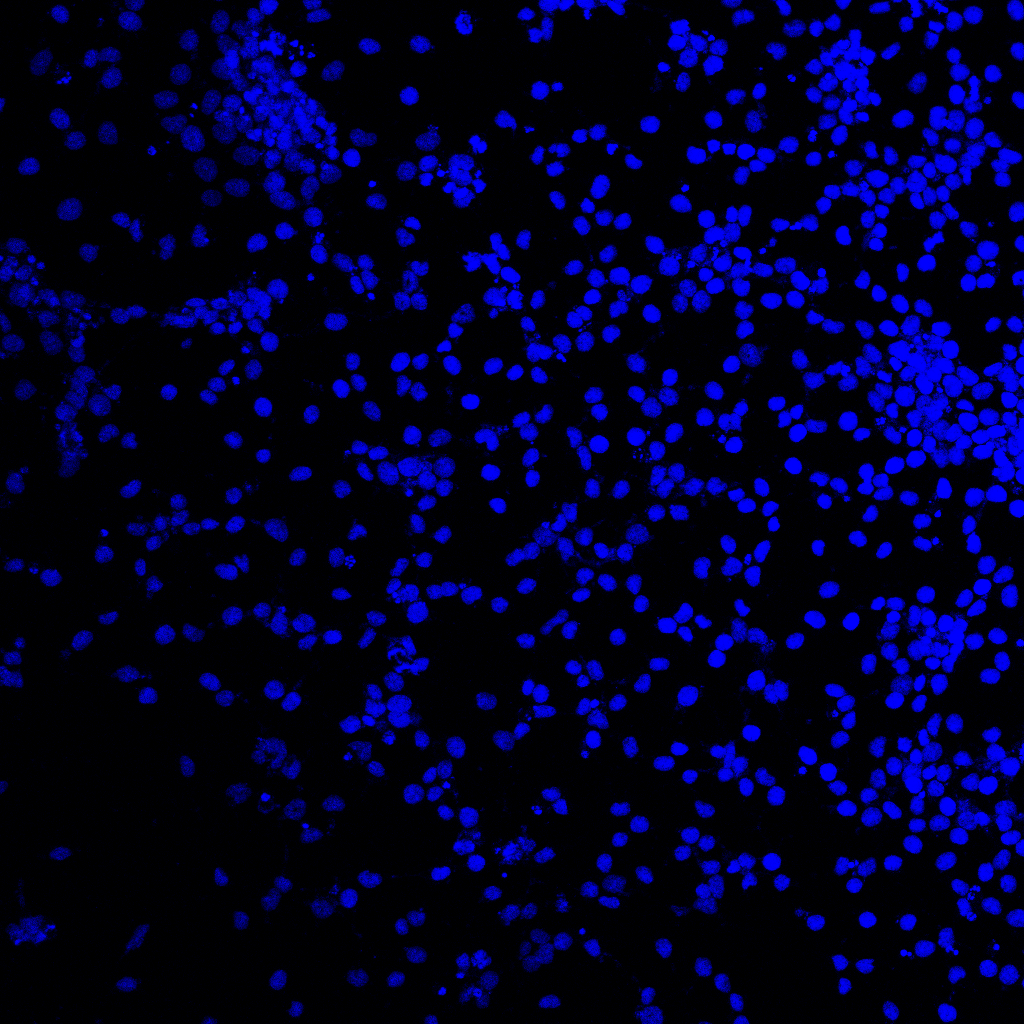

Supplement: Supplementary file 5 — Source data Fig. 3 [file 44319_2024_205_MOESM5_ESM.zip › Source_data_Figure3/3B/Proliferation/siControl/Hoechst.tif]

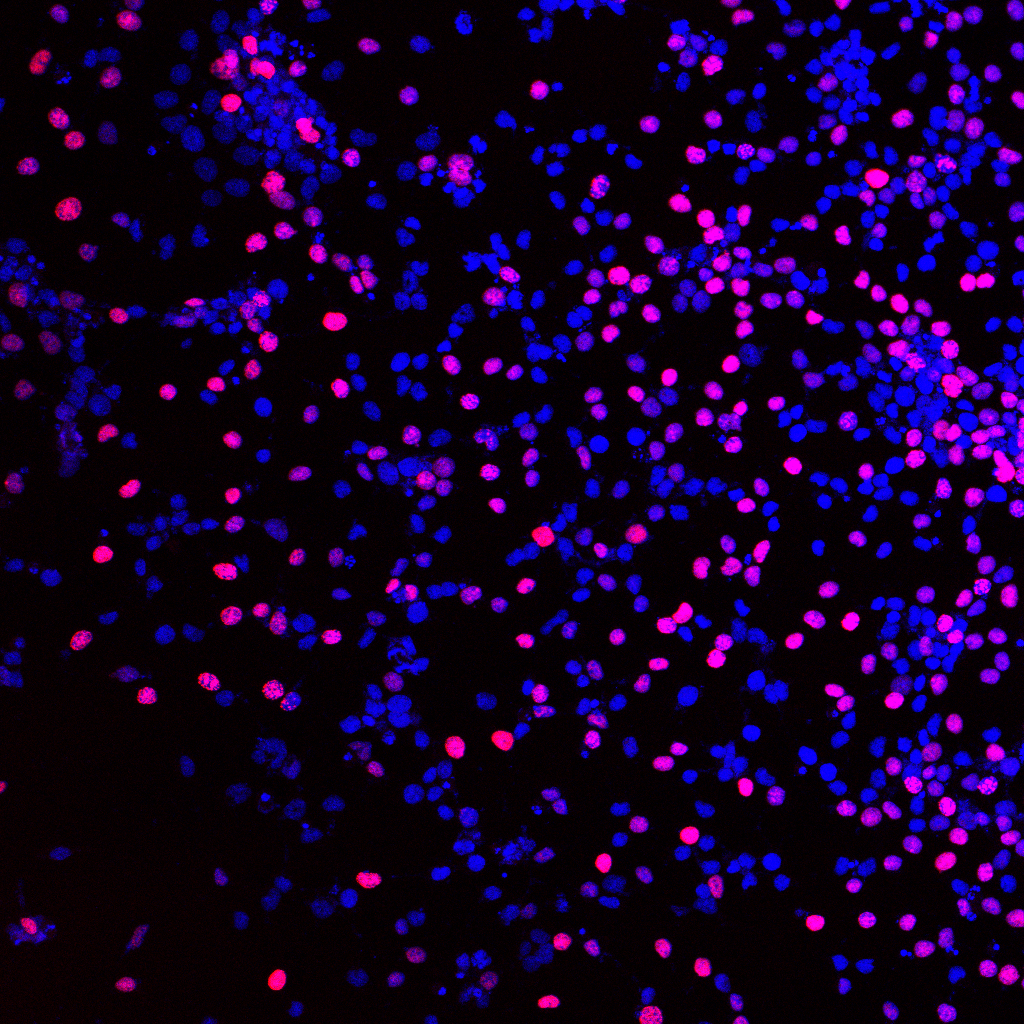

Supplement: Supplementary file 5 — Source data Fig. 3 [file 44319_2024_205_MOESM5_ESM.zip › Source_data_Figure3/3B/Proliferation/siControl/Merge.tif]

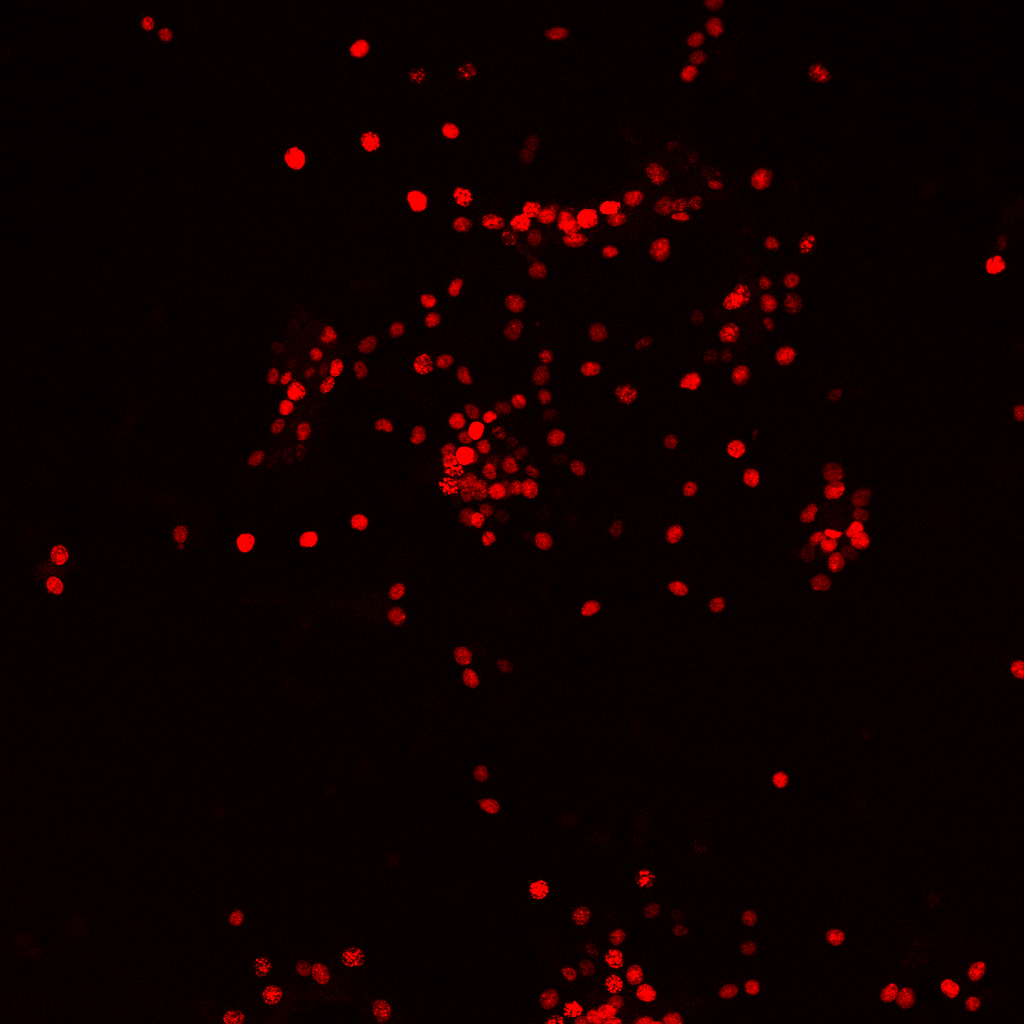

Supplement: Supplementary file 5 — Source data Fig. 3 [file 44319_2024_205_MOESM5_ESM.zip › Source_data_Figure3/3B/Proliferation/siDerl1/EdU.tif]

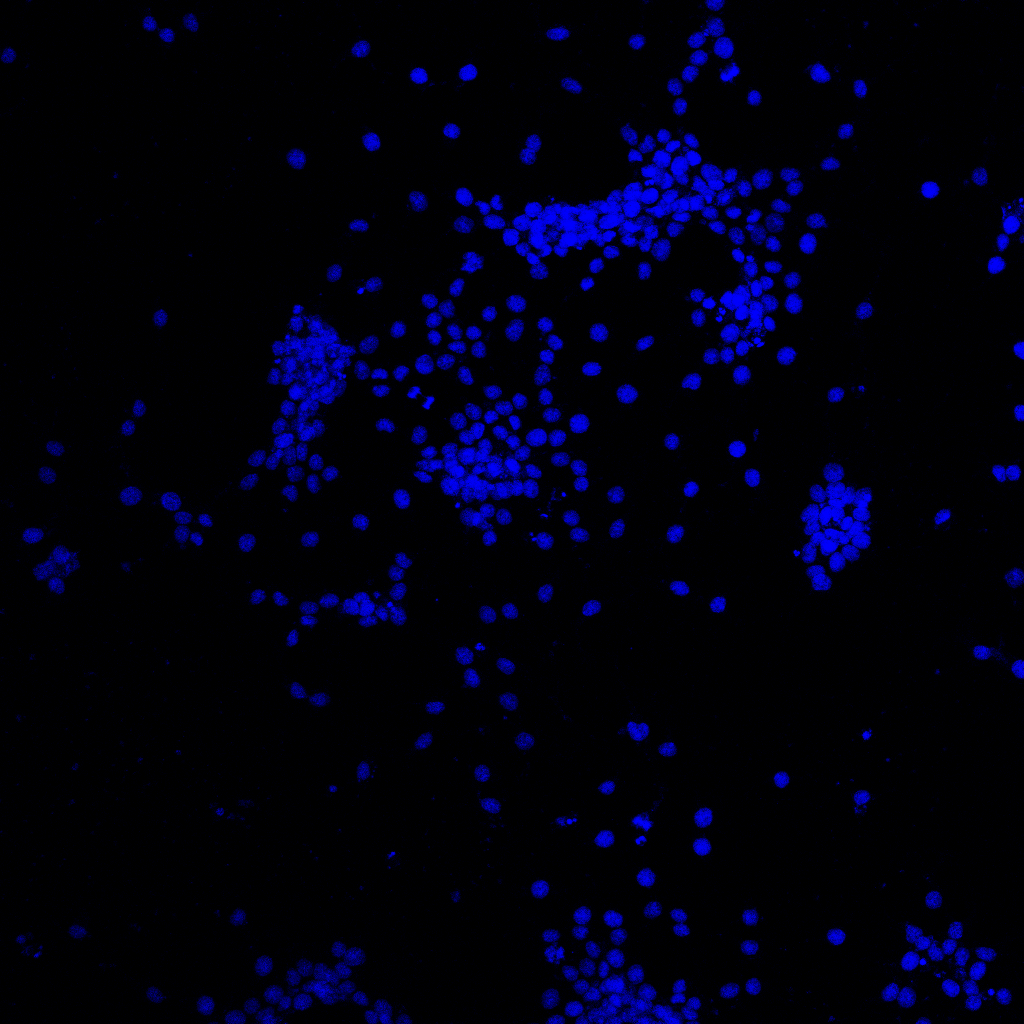

Supplement: Supplementary file 5 — Source data Fig. 3 [file 44319_2024_205_MOESM5_ESM.zip › Source_data_Figure3/3B/Proliferation/siDerl1/Hoechst.tif]

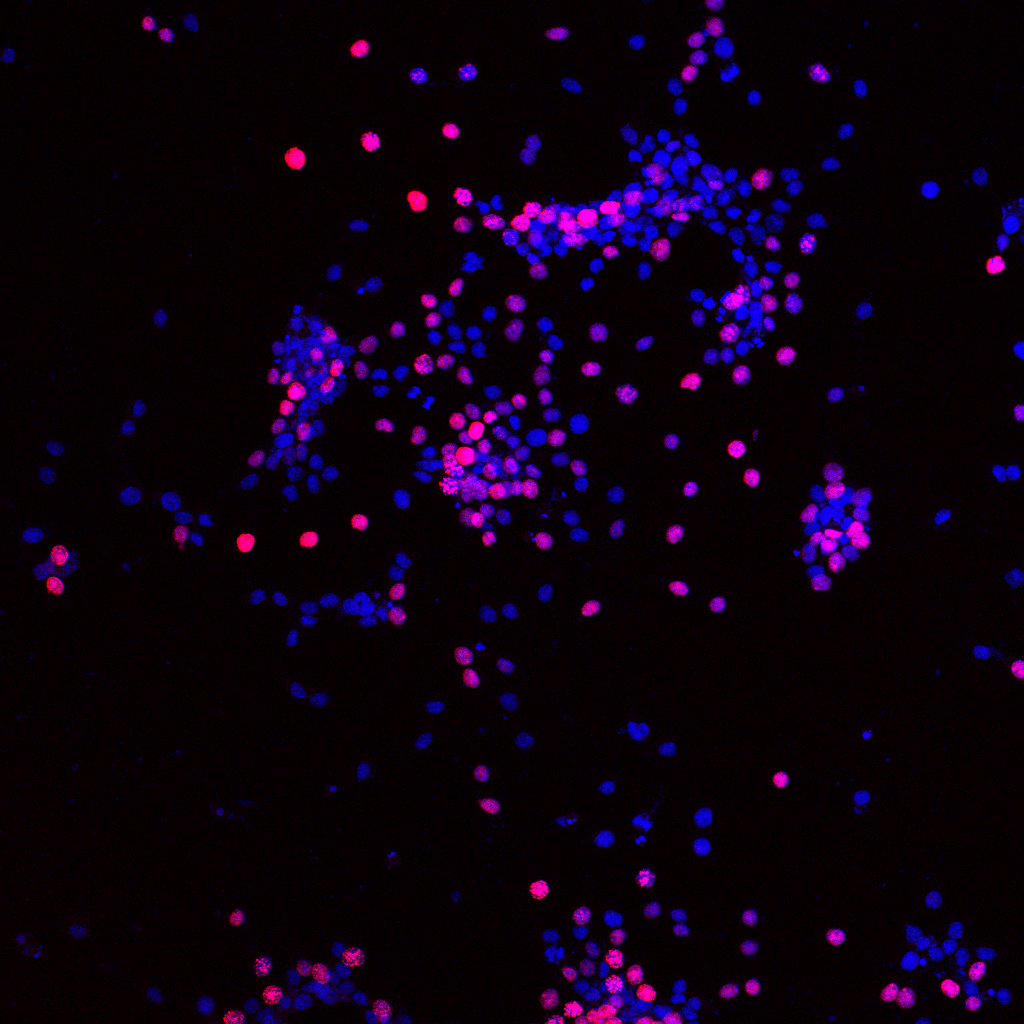

Supplement: Supplementary file 5 — Source data Fig. 3 [file 44319_2024_205_MOESM5_ESM.zip › Source_data_Figure3/3B/Proliferation/siDerl1/Merge.tif]

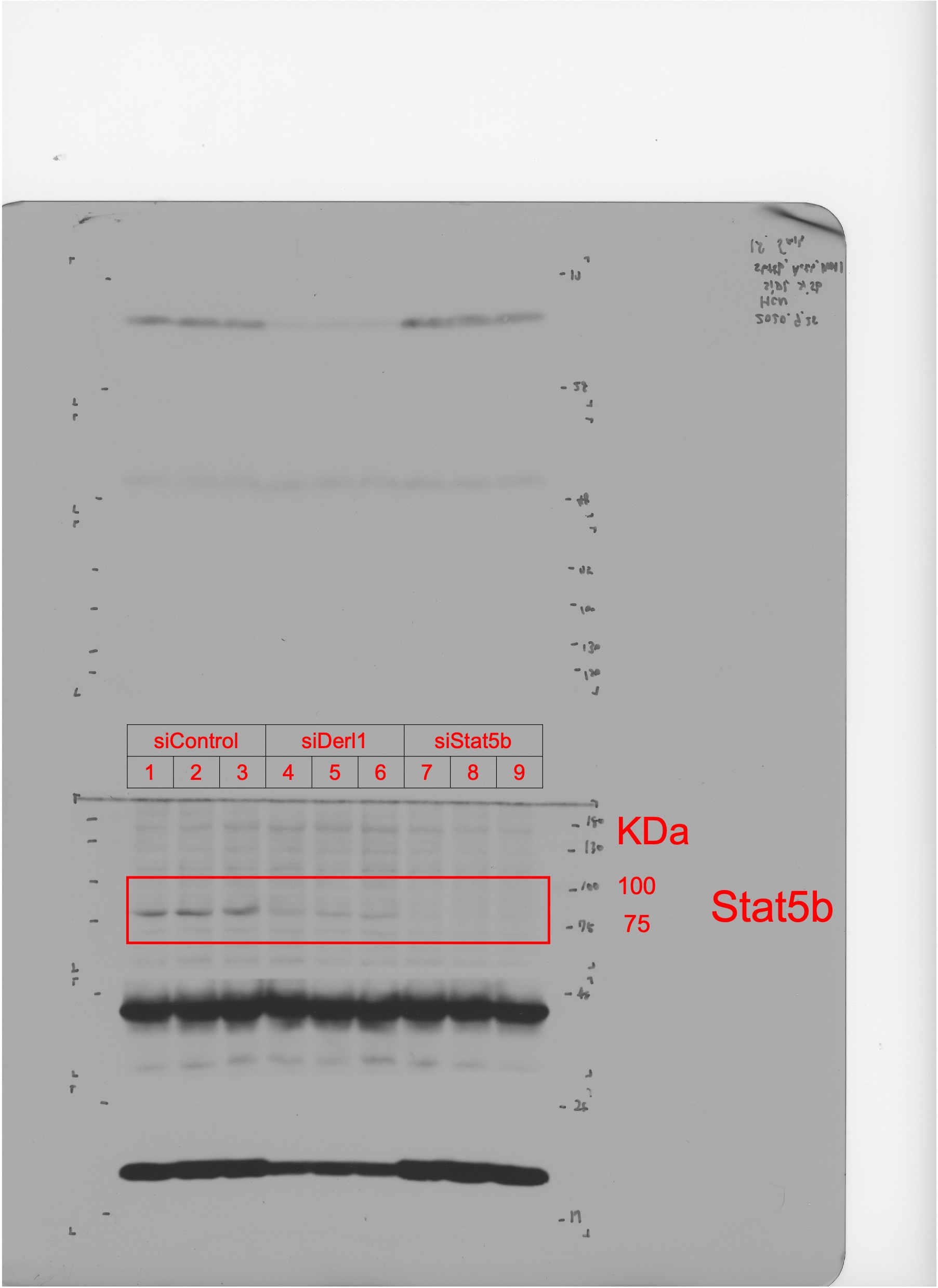

Supplement: Supplementary file 6 — Source data Fig. 4 [file 44319_2024_205_MOESM6_ESM.zip › Source_data_Figure4/4C/western Stat5b.tif]

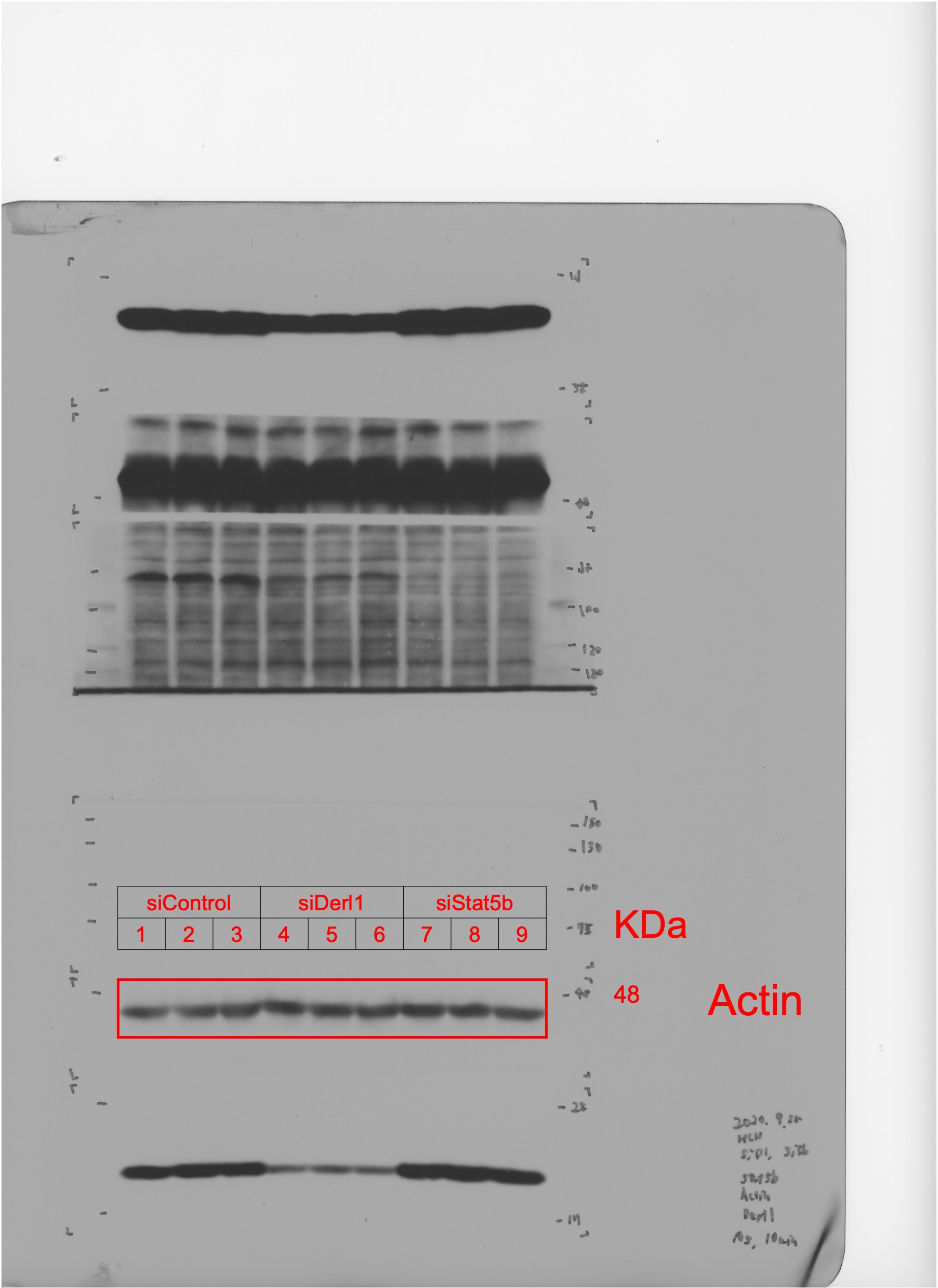

Supplement: Supplementary file 6 — Source data Fig. 4 [file 44319_2024_205_MOESM6_ESM.zip › Source_data_Figure4/4C/western Actin.tif]

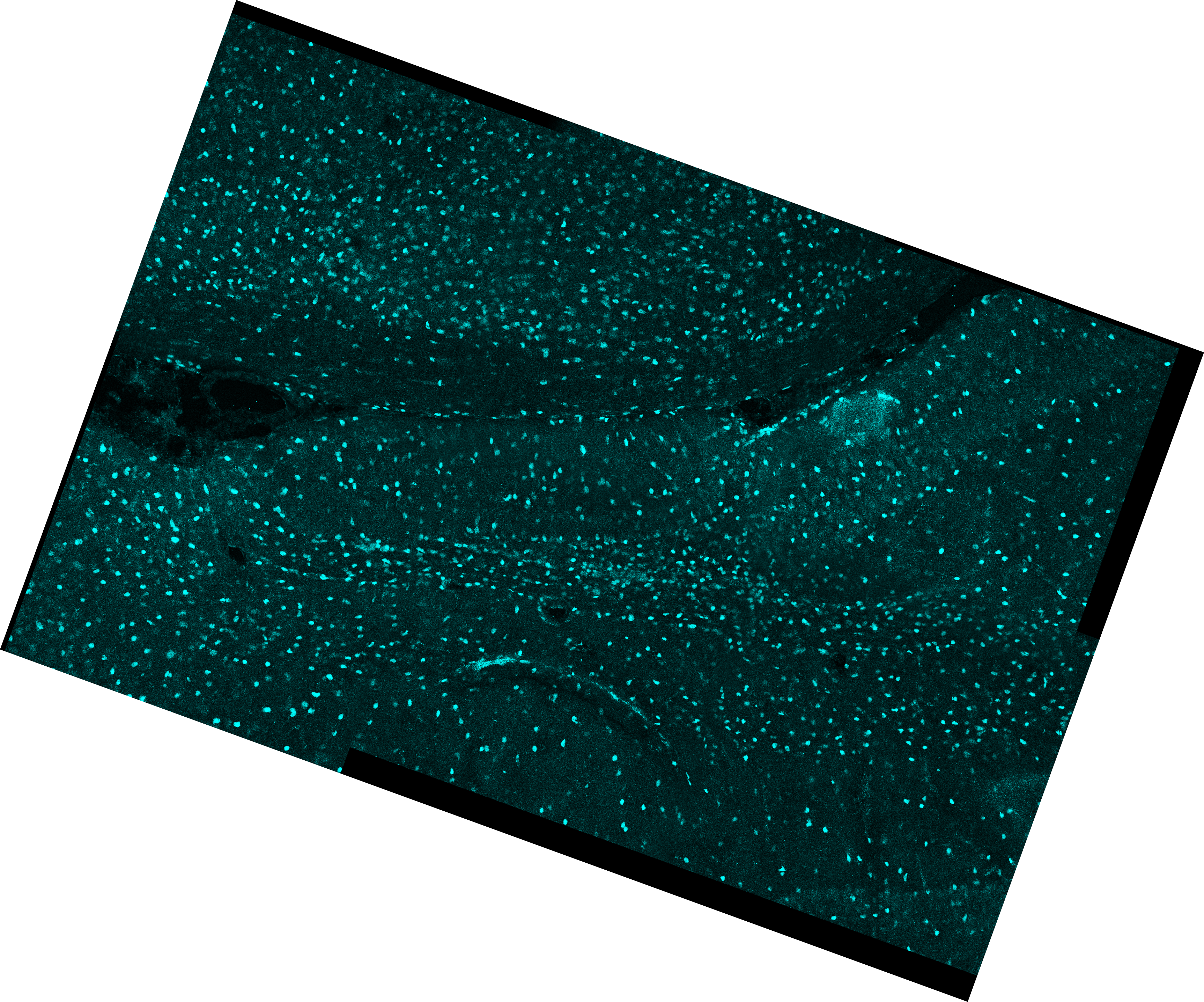

Supplement: Supplementary file 6 — Source data Fig. 4 [file 44319_2024_205_MOESM6_ESM.zip › Source_data_Figure4/4K/Derl1NesCre + Control virus/Sox2.tif]

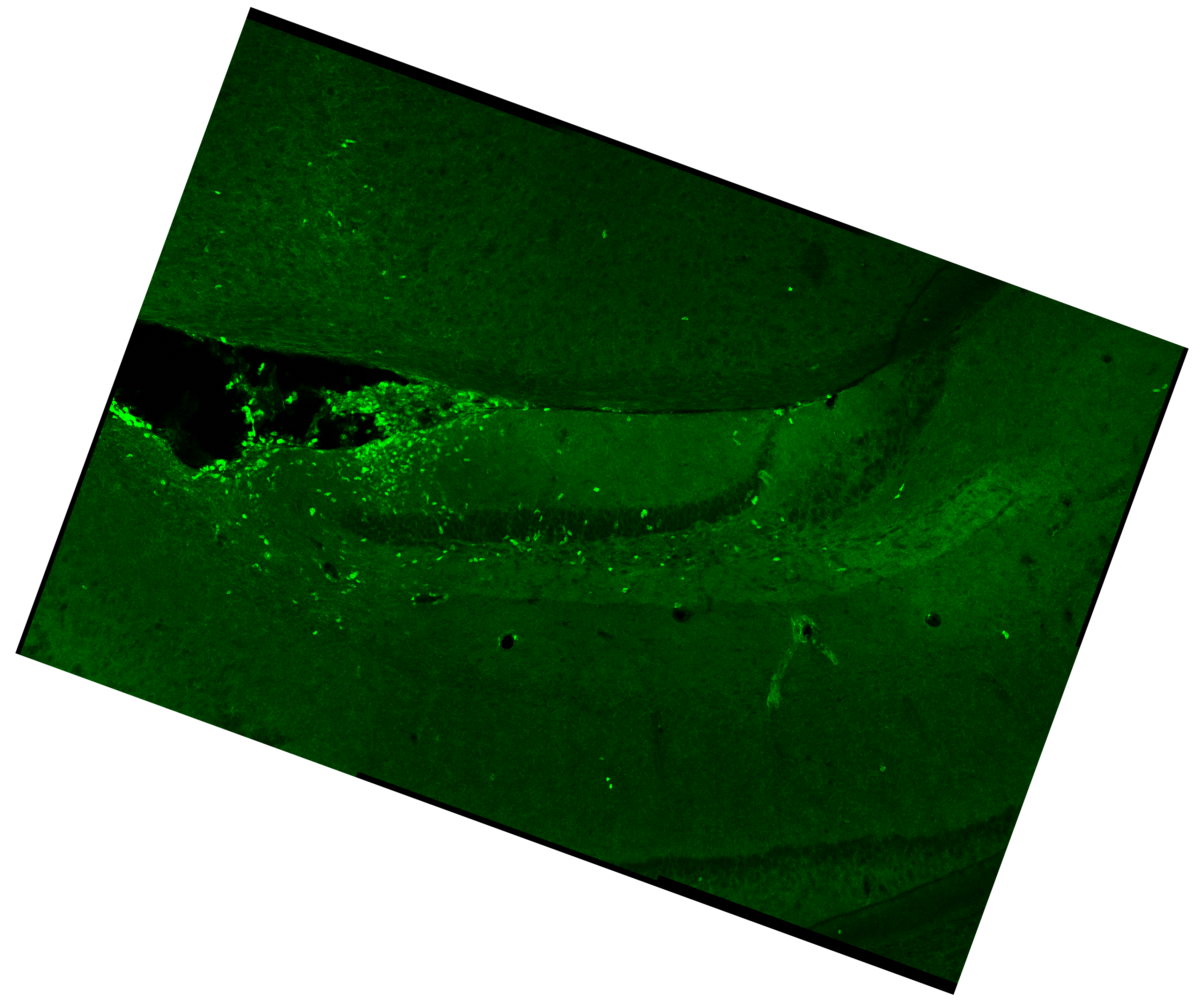

Supplement: Supplementary file 6 — Source data Fig. 4 [file 44319_2024_205_MOESM6_ESM.zip › Source_data_Figure4/4K/Derl1NesCre + Control virus/Ki67.tif]

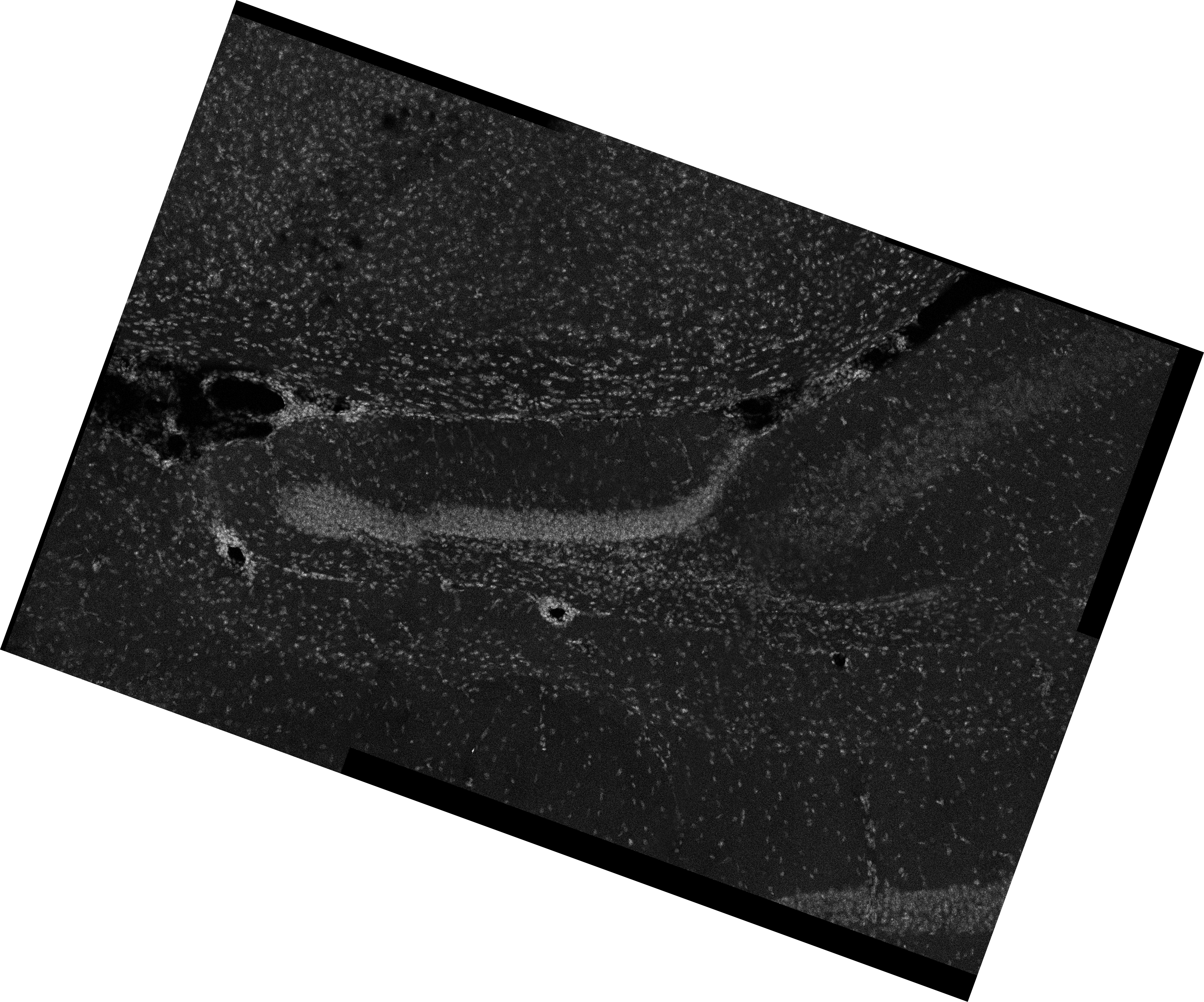

Supplement: Supplementary file 6 — Source data Fig. 4 [file 44319_2024_205_MOESM6_ESM.zip › Source_data_Figure4/4K/Derl1NesCre + Control virus/Hoechst.tif]

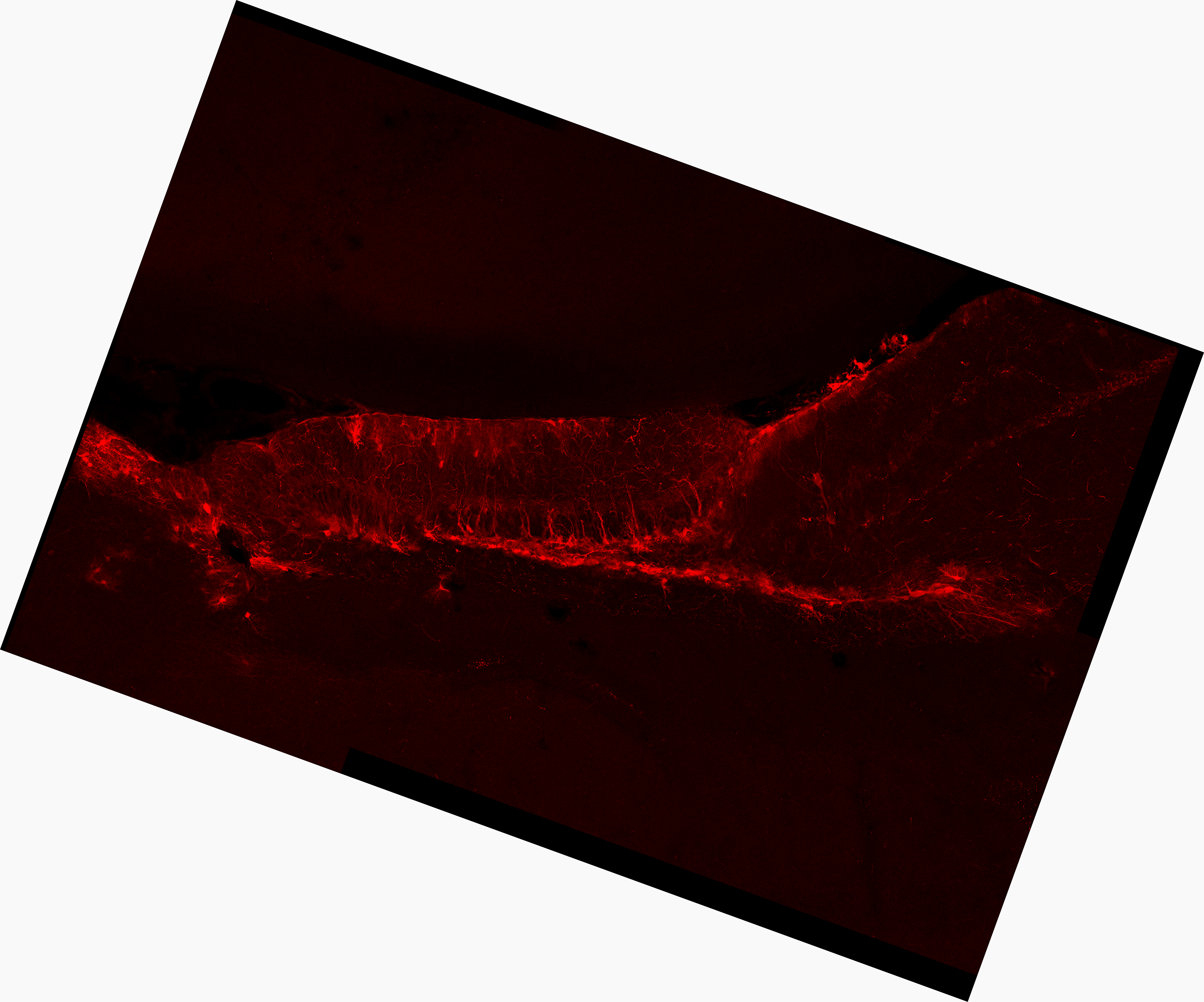

Supplement: Supplementary file 6 — Source data Fig. 4 [file 44319_2024_205_MOESM6_ESM.zip › Source_data_Figure4/4K/Derl1NesCre + Control virus/HA.tif]

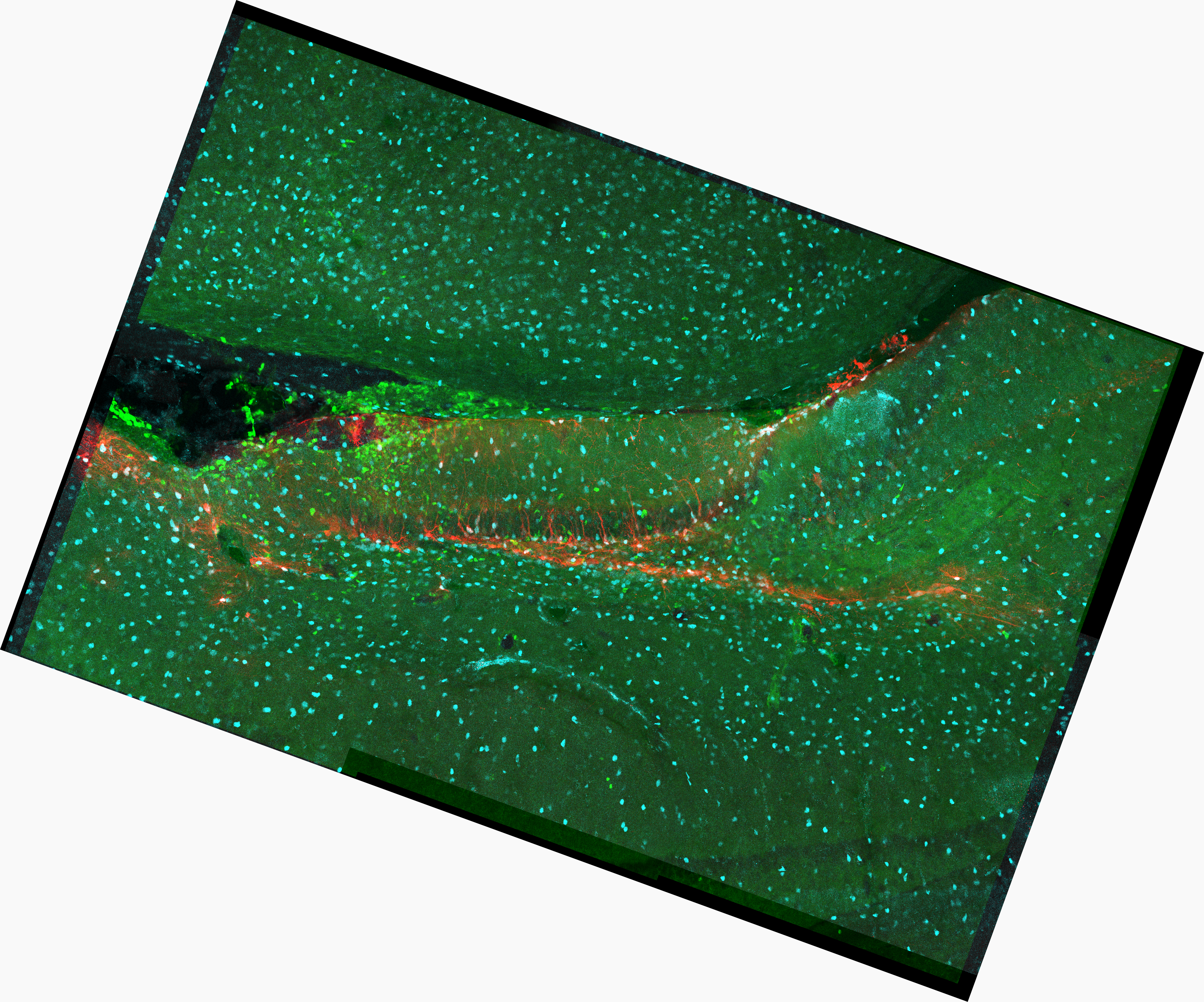

Supplement: Supplementary file 6 — Source data Fig. 4 [file 44319_2024_205_MOESM6_ESM.zip › Source_data_Figure4/4K/Derl1NesCre + Control virus/Merge.tif]

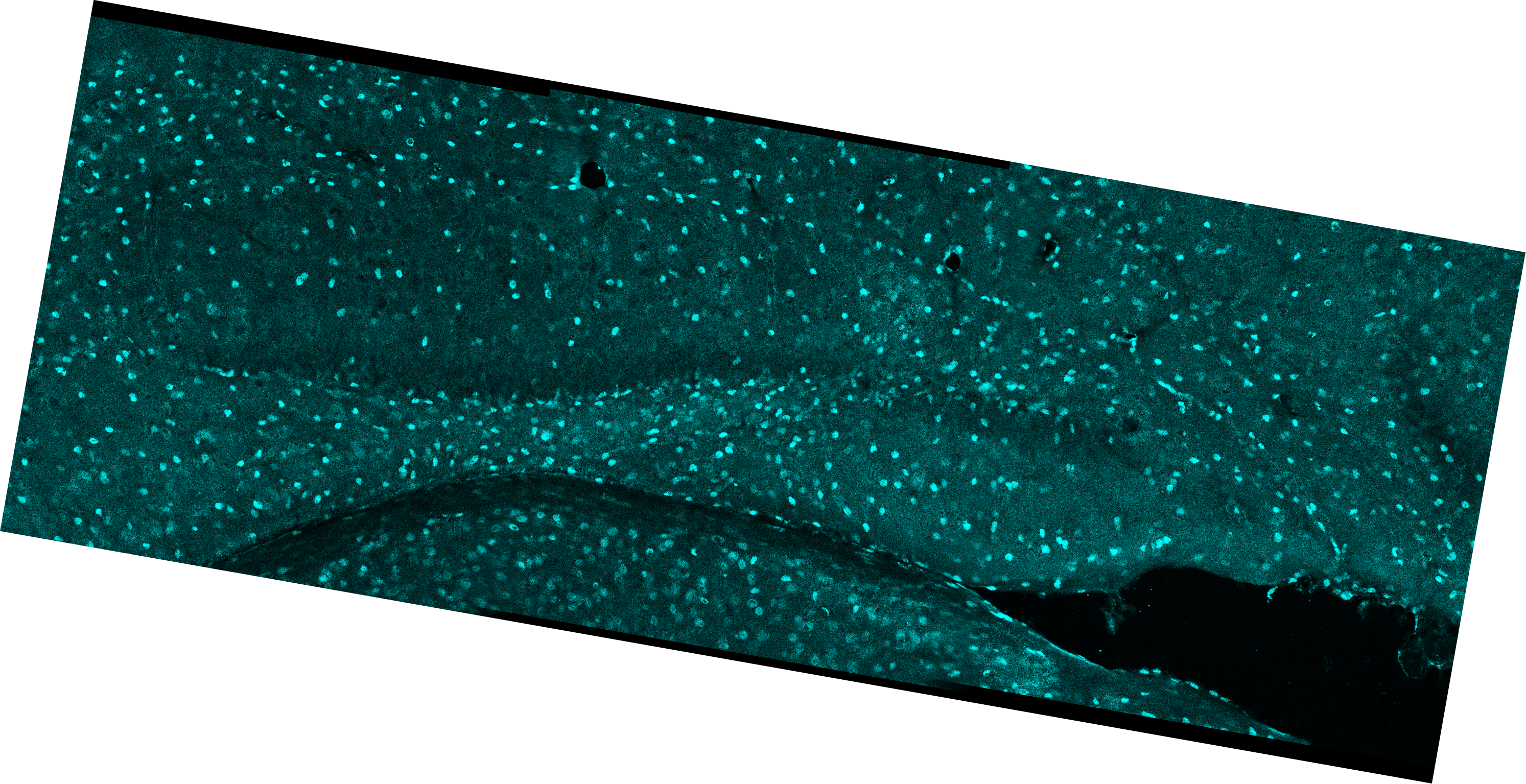

Supplement: Supplementary file 6 — Source data Fig. 4 [file 44319_2024_205_MOESM6_ESM.zip › Source_data_Figure4/4K/Derl1NesCre + Stat5b virus/Sox2.tif]

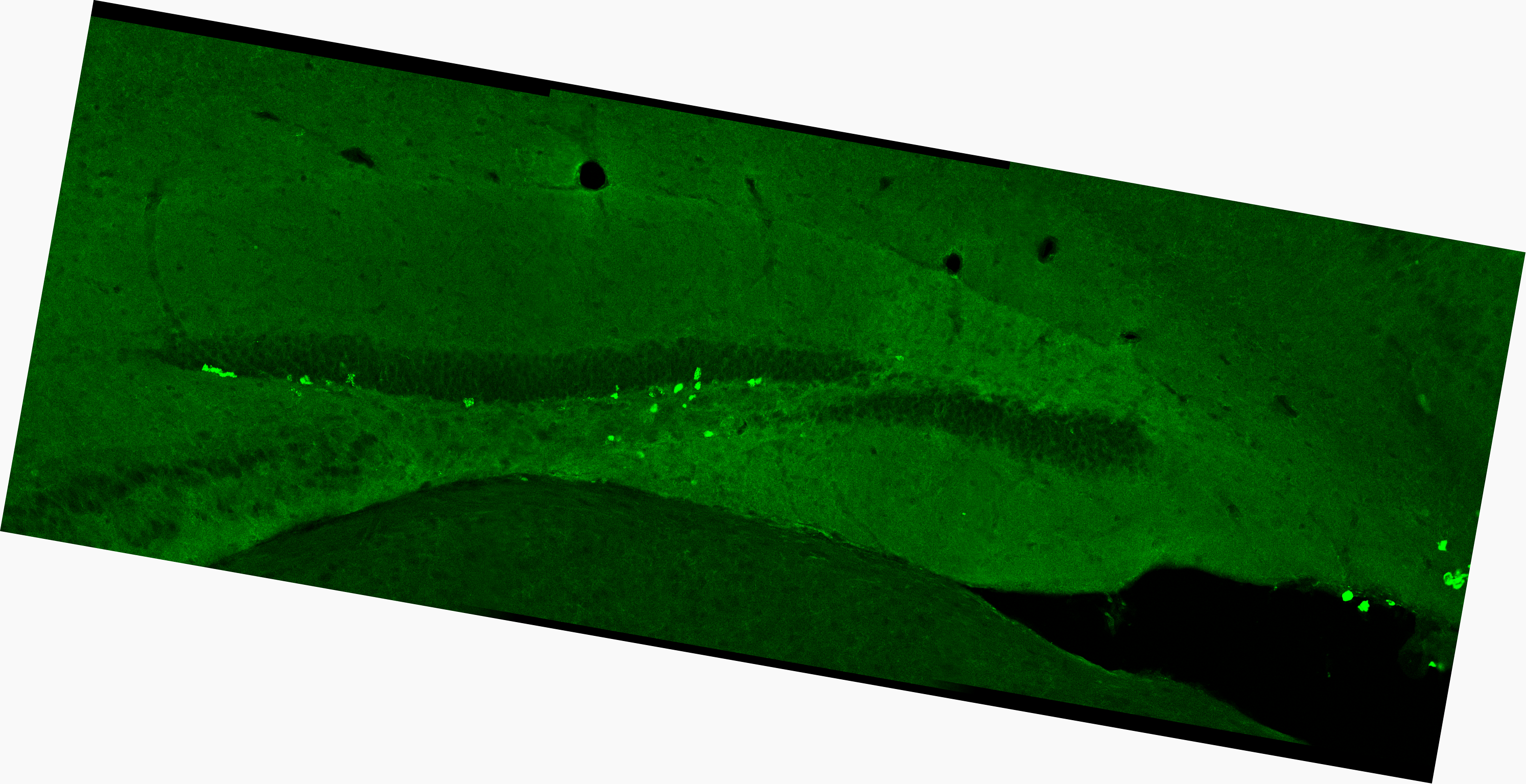

Supplement: Supplementary file 6 — Source data Fig. 4 [file 44319_2024_205_MOESM6_ESM.zip › Source_data_Figure4/4K/Derl1NesCre + Stat5b virus/Ki67.tif]

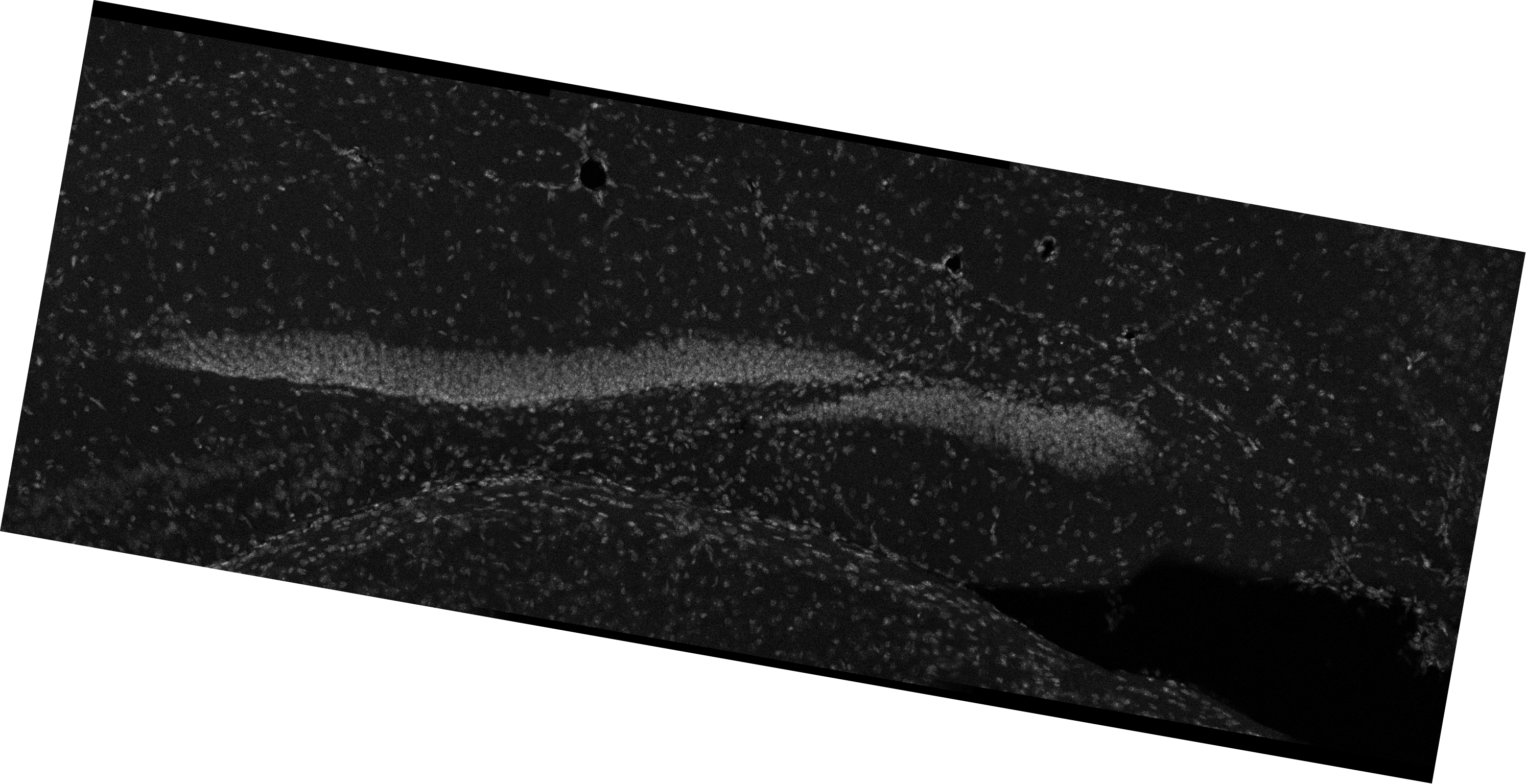

Supplement: Supplementary file 6 — Source data Fig. 4 [file 44319_2024_205_MOESM6_ESM.zip › Source_data_Figure4/4K/Derl1NesCre + Stat5b virus/Hoechst.tif]

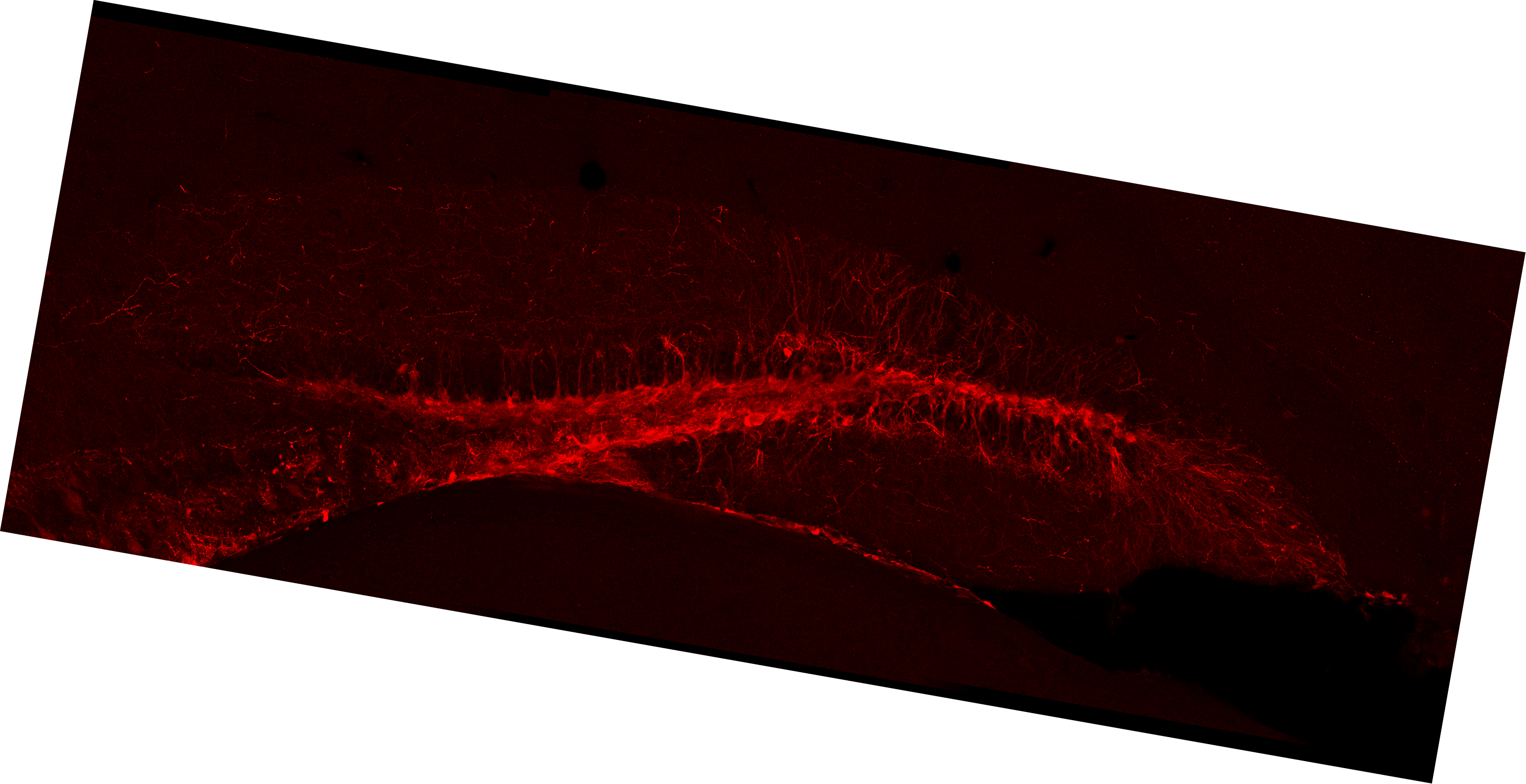

Supplement: Supplementary file 6 — Source data Fig. 4 [file 44319_2024_205_MOESM6_ESM.zip › Source_data_Figure4/4K/Derl1NesCre + Stat5b virus/HA.tif]

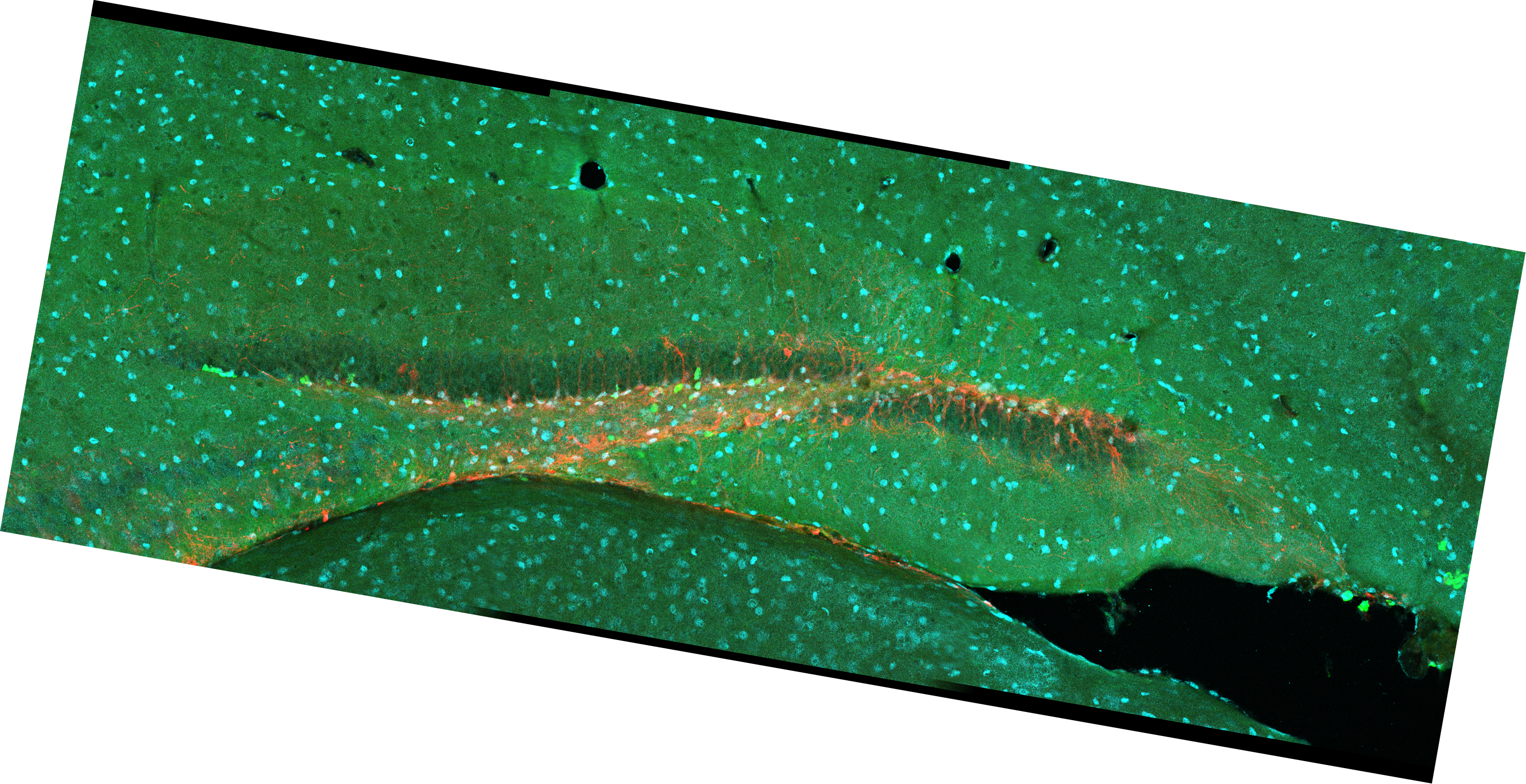

Supplement: Supplementary file 6 — Source data Fig. 4 [file 44319_2024_205_MOESM6_ESM.zip › Source_data_Figure4/4K/Derl1NesCre + Stat5b virus/Merge.tif]

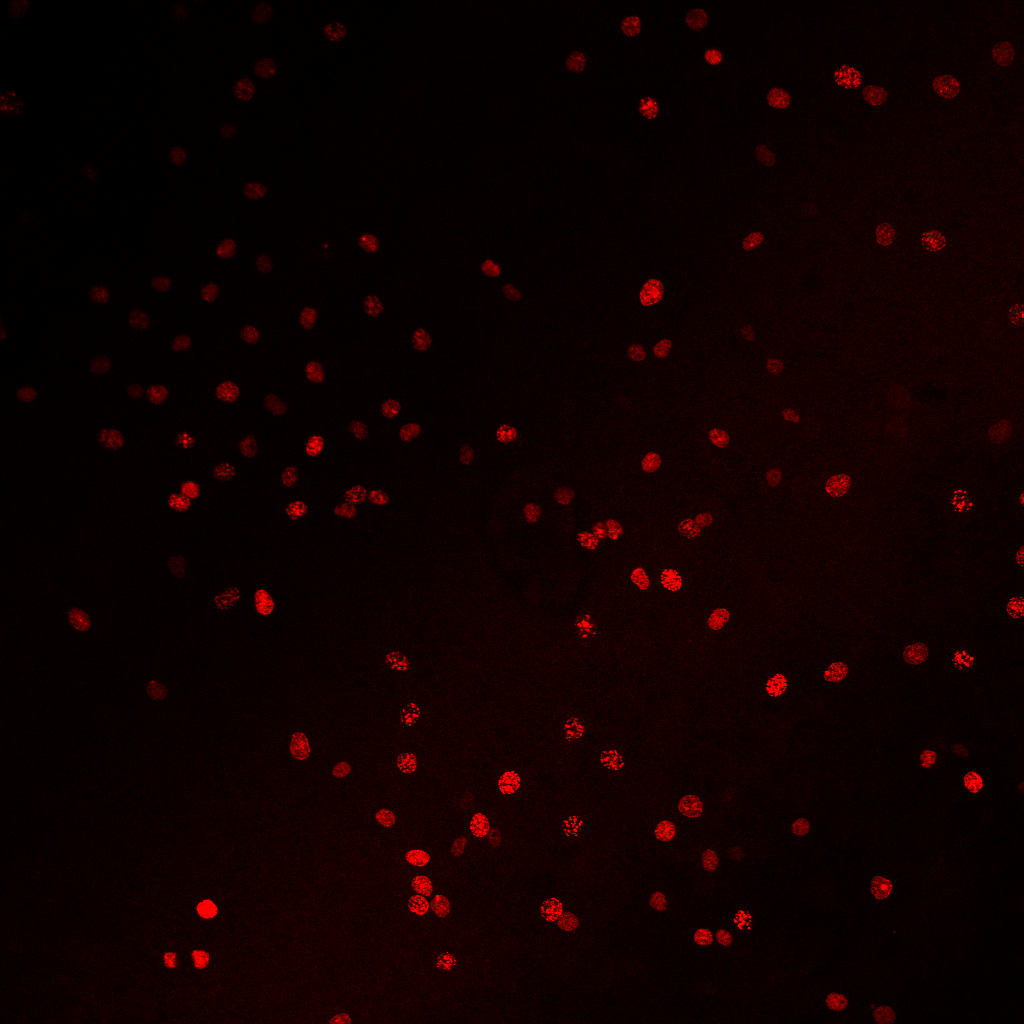

Supplement: Supplementary file 6 — Source data Fig. 4 [file 44319_2024_205_MOESM6_ESM.zip › Source_data_Figure4/4D/siStat5b/EdU.tif]

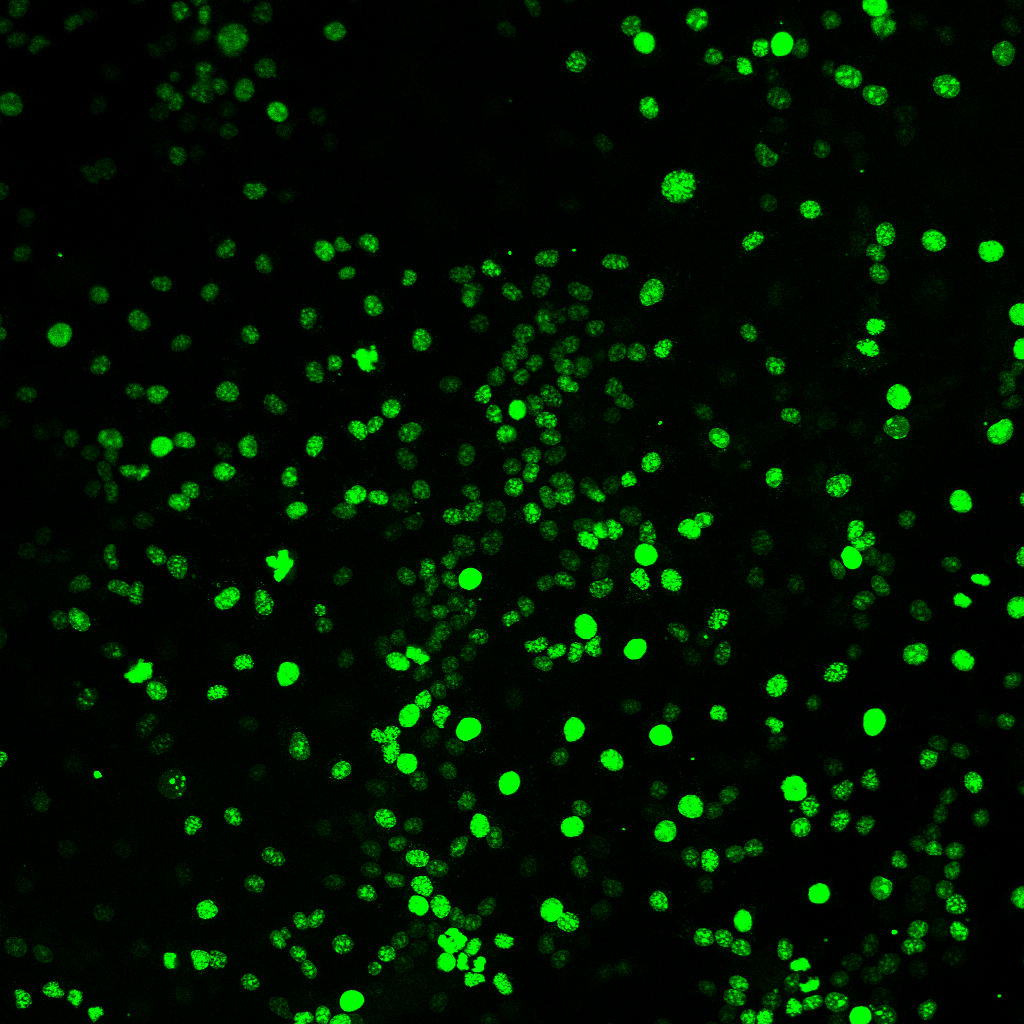

Supplement: Supplementary file 6 — Source data Fig. 4 [file 44319_2024_205_MOESM6_ESM.zip › Source_data_Figure4/4D/siStat5b/Ki67.tif]

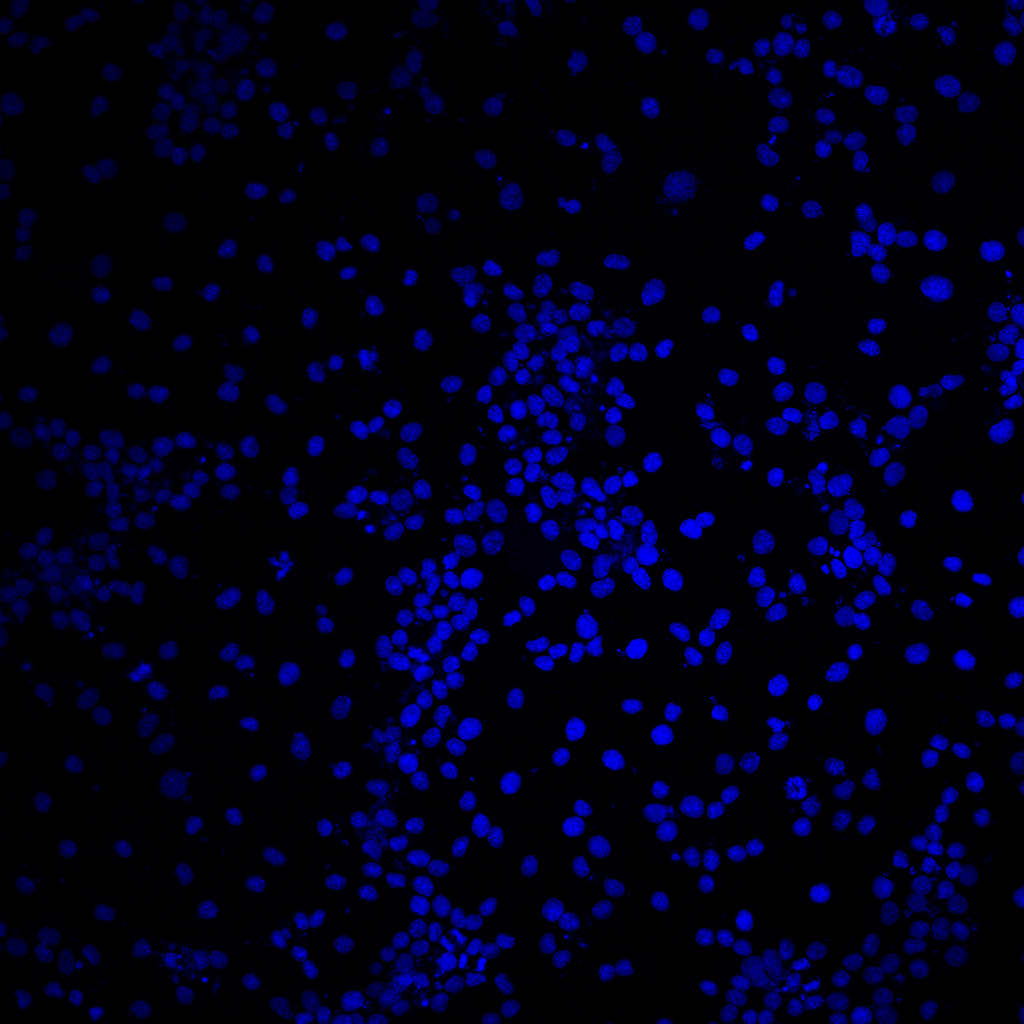

Supplement: Supplementary file 6 — Source data Fig. 4 [file 44319_2024_205_MOESM6_ESM.zip › Source_data_Figure4/4D/siStat5b/Hoechst.tif]

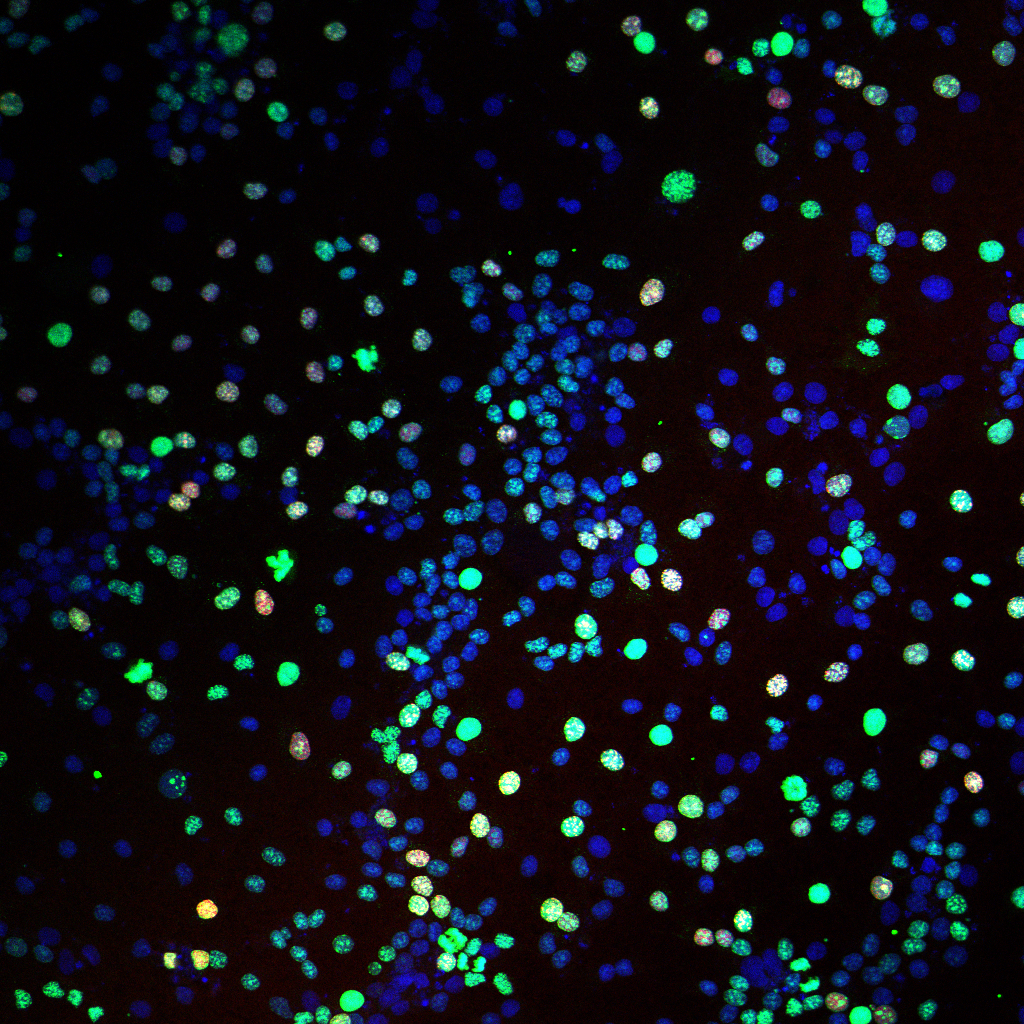

Supplement: Supplementary file 6 — Source data Fig. 4 [file 44319_2024_205_MOESM6_ESM.zip › Source_data_Figure4/4D/siStat5b/Merge.tif]

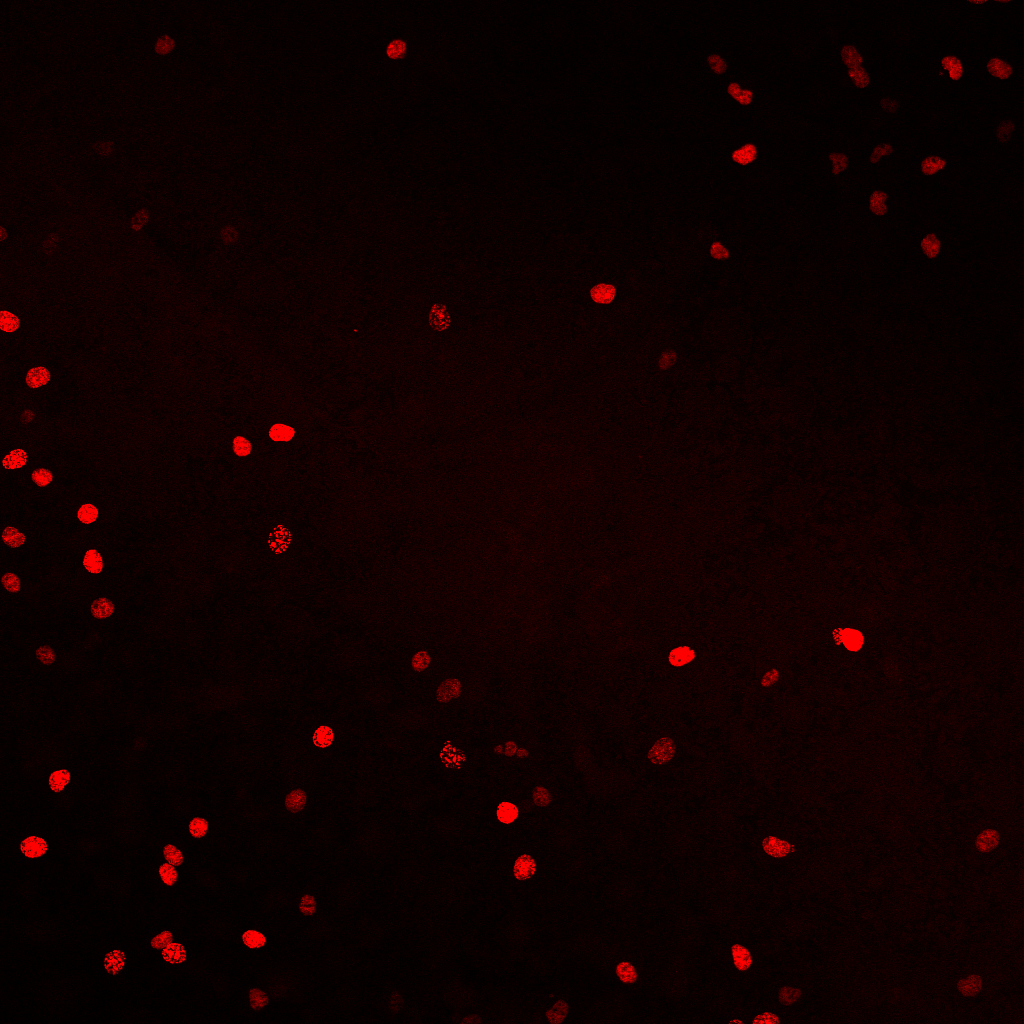

Supplement: Supplementary file 6 — Source data Fig. 4 [file 44319_2024_205_MOESM6_ESM.zip › Source_data_Figure4/4D/siControl/EdU.tif]

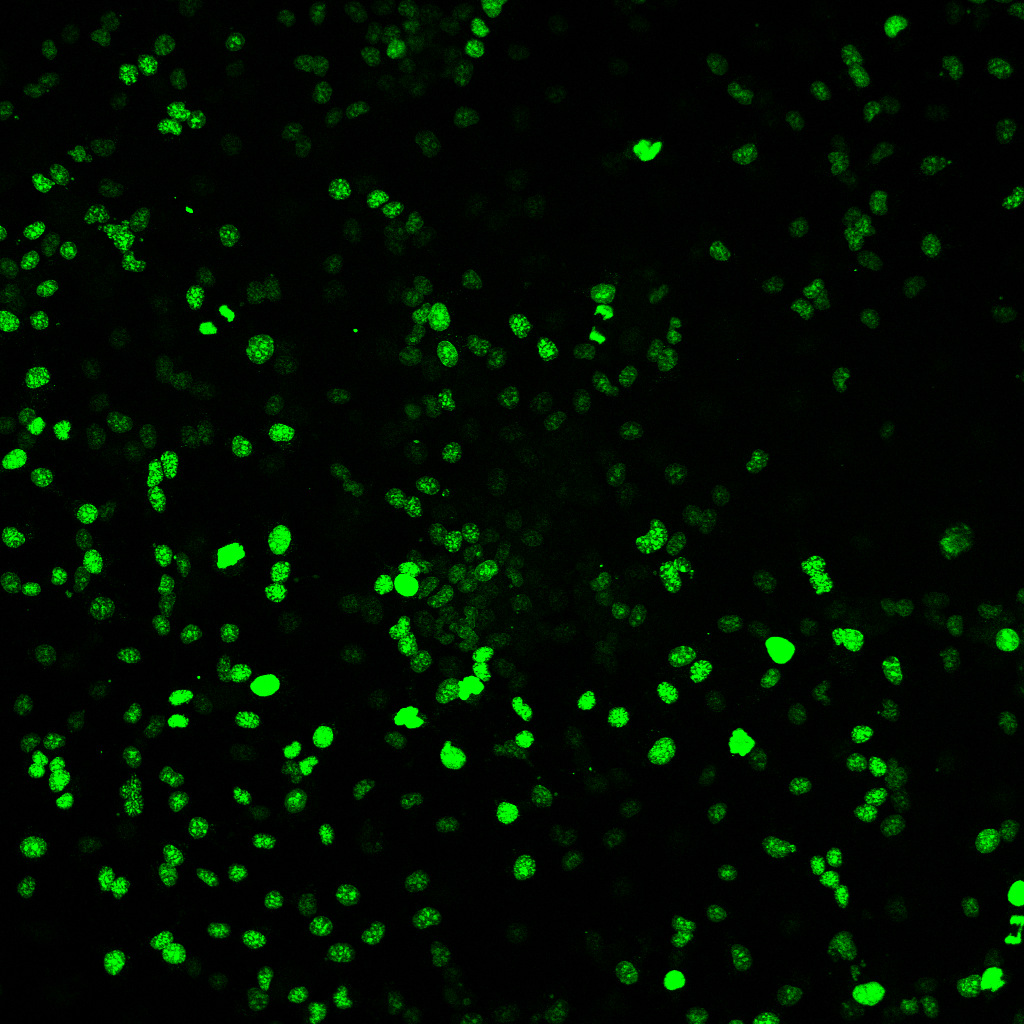

Supplement: Supplementary file 6 — Source data Fig. 4 [file 44319_2024_205_MOESM6_ESM.zip › Source_data_Figure4/4D/siControl/Ki67.tif]

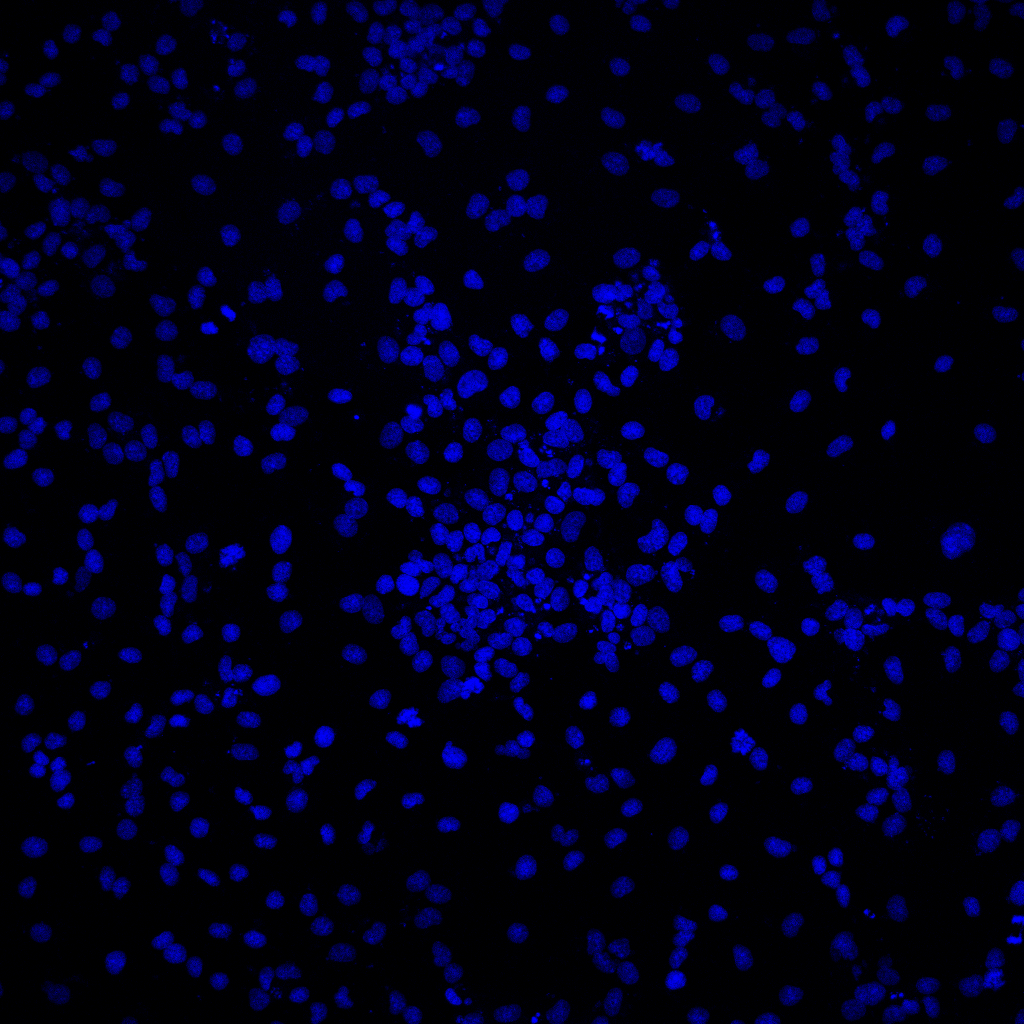

Supplement: Supplementary file 6 — Source data Fig. 4 [file 44319_2024_205_MOESM6_ESM.zip › Source_data_Figure4/4D/siControl/Hoechst.tif]

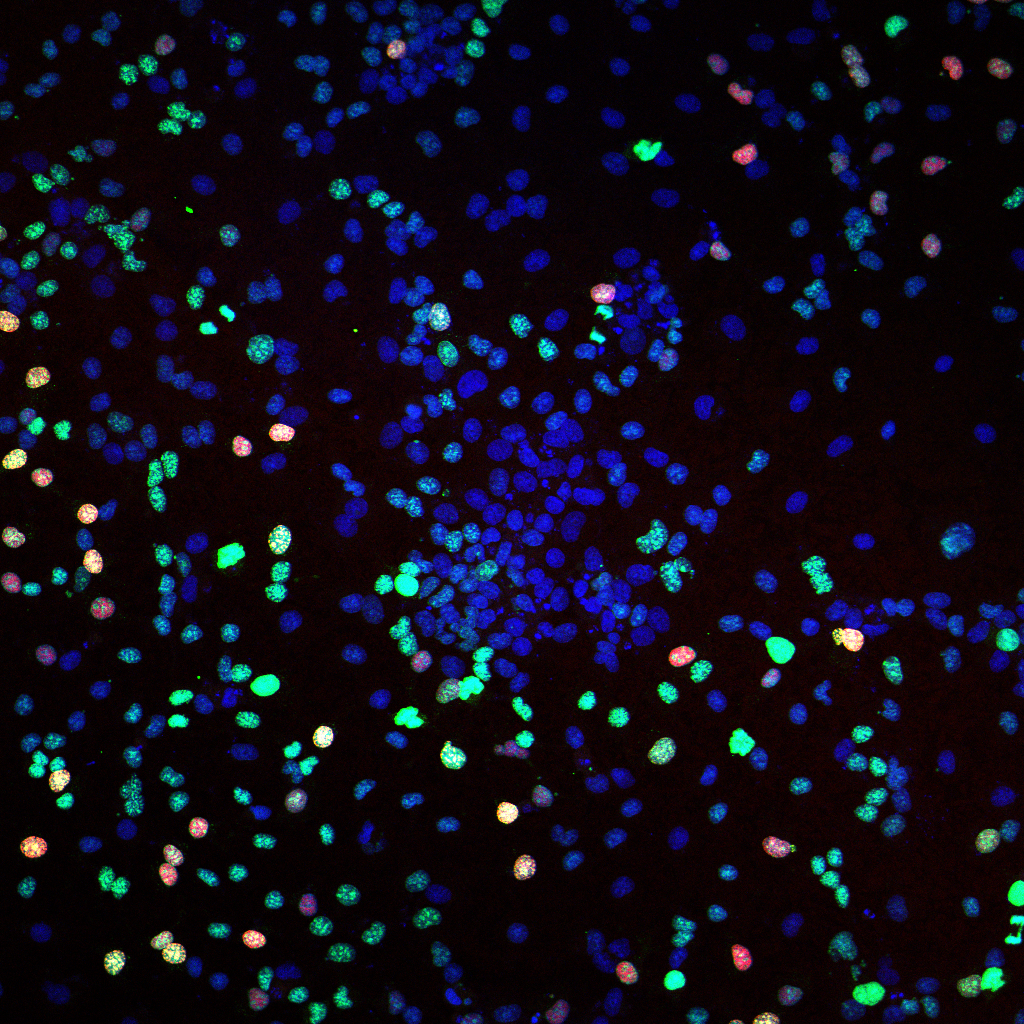

Supplement: Supplementary file 6 — Source data Fig. 4 [file 44319_2024_205_MOESM6_ESM.zip › Source_data_Figure4/4D/siControl/Merge.tif]

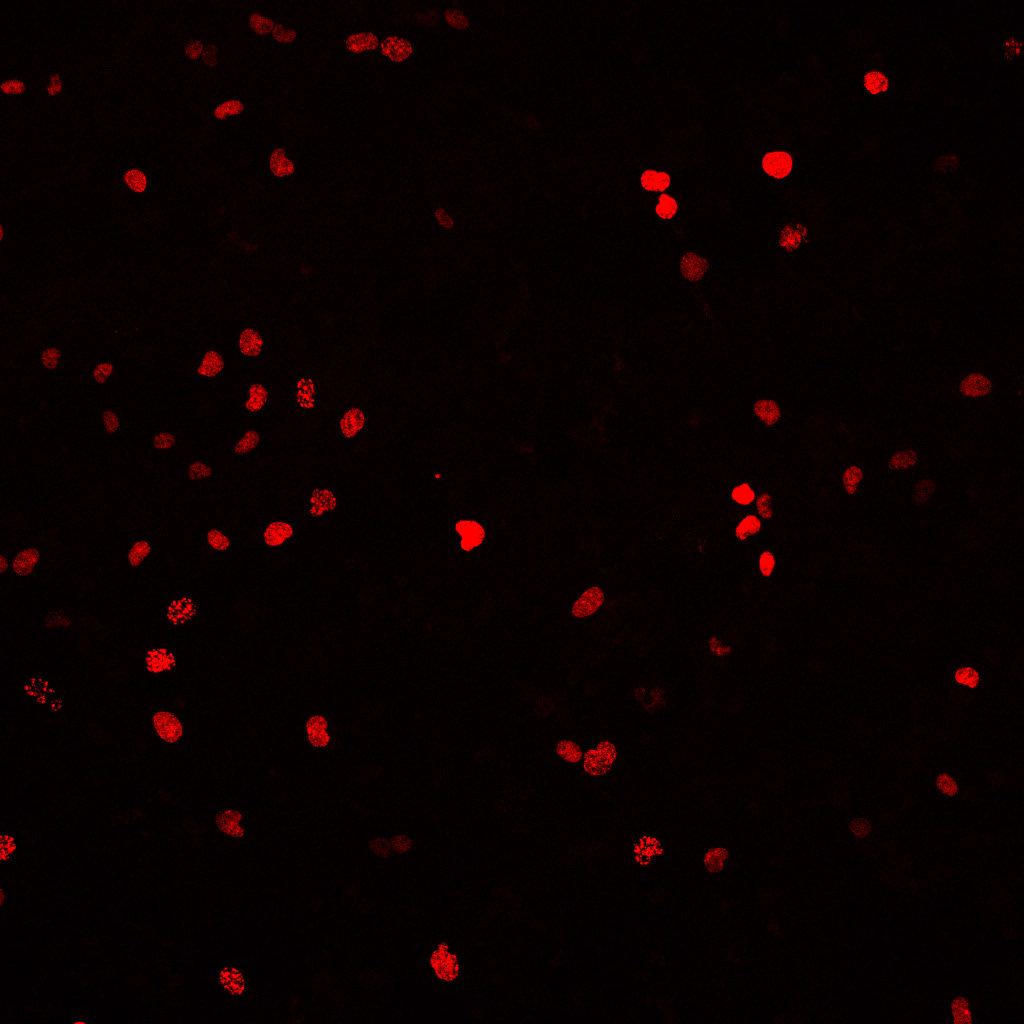

Supplement: Supplementary file 6 — Source data Fig. 4 [file 44319_2024_205_MOESM6_ESM.zip › Source_data_Figure4/4H/siControl + Stat5b/EdU.tif]

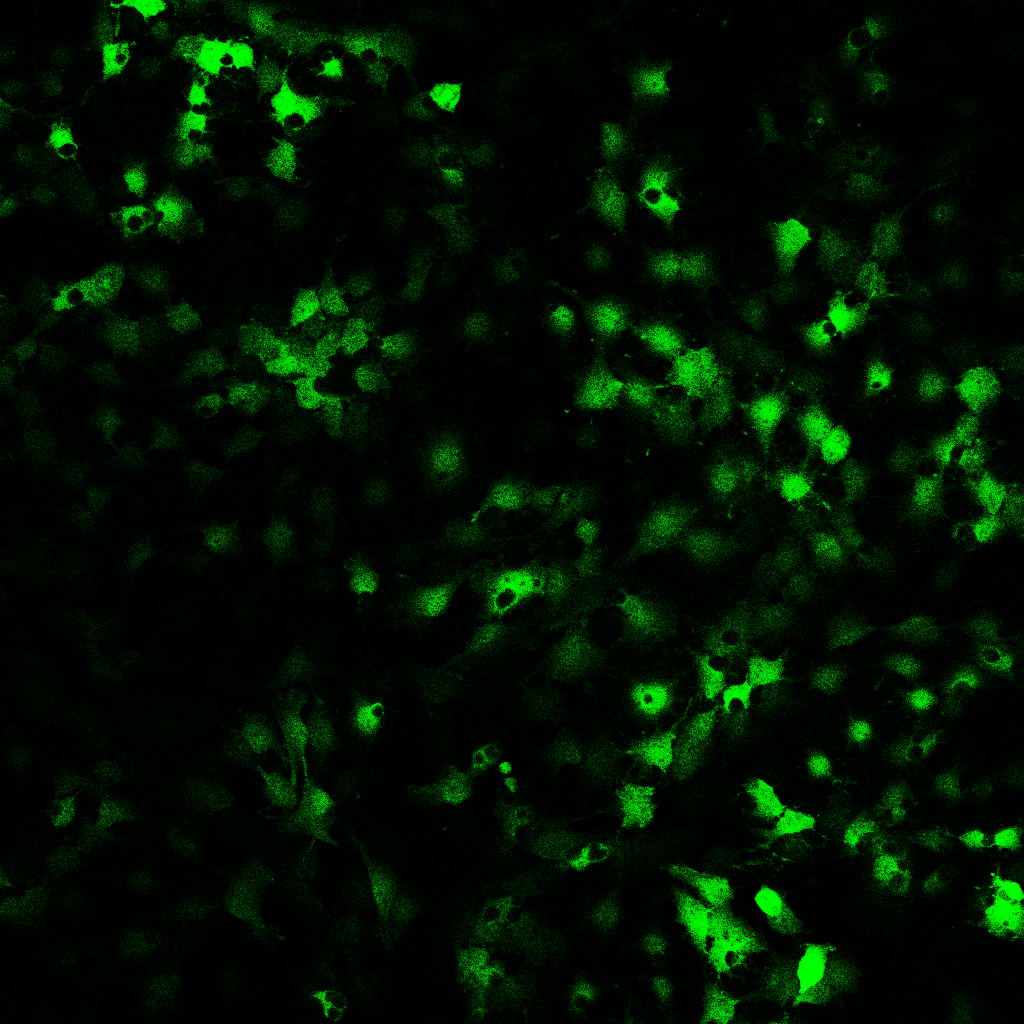

Supplement: Supplementary file 6 — Source data Fig. 4 [file 44319_2024_205_MOESM6_ESM.zip › Source_data_Figure4/4H/siControl + Stat5b/GFP.tif]

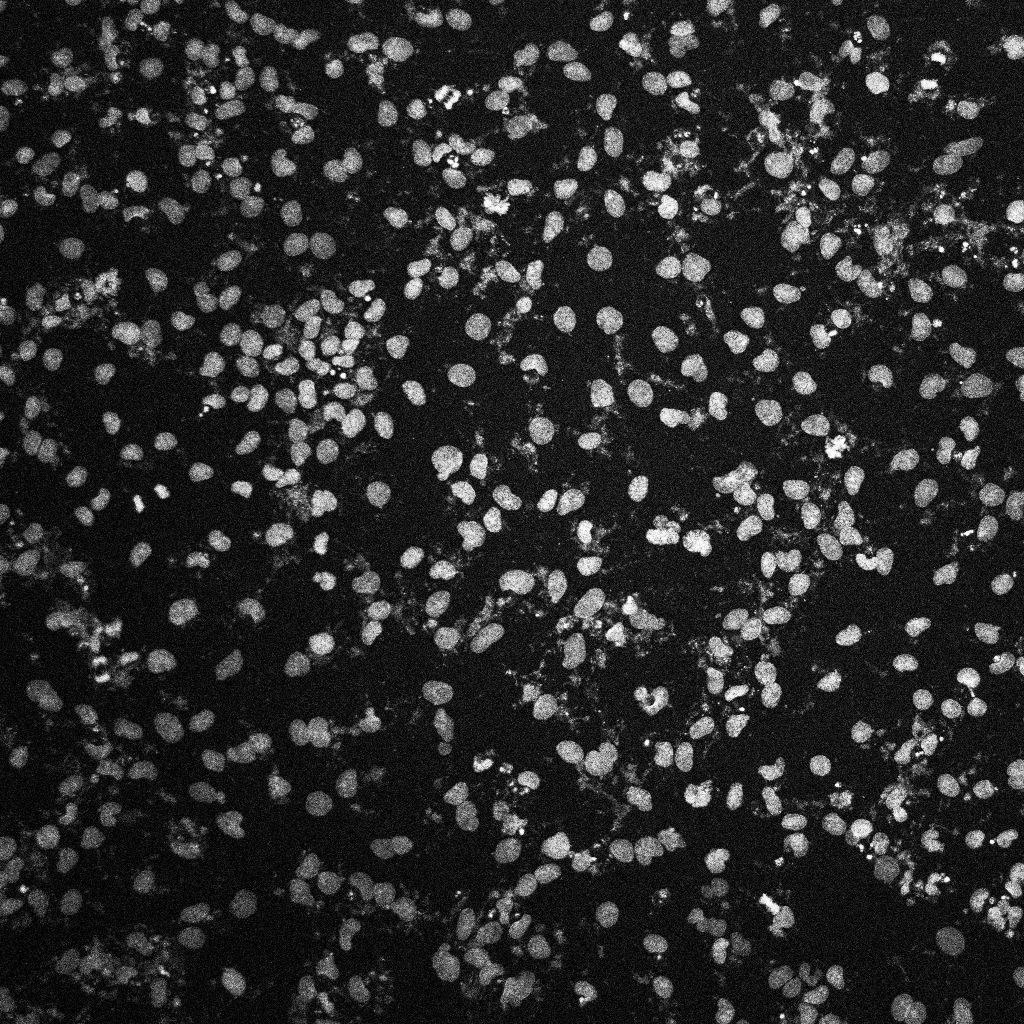

Supplement: Supplementary file 6 — Source data Fig. 4 [file 44319_2024_205_MOESM6_ESM.zip › Source_data_Figure4/4H/siControl + Stat5b/Hoechst.tif]

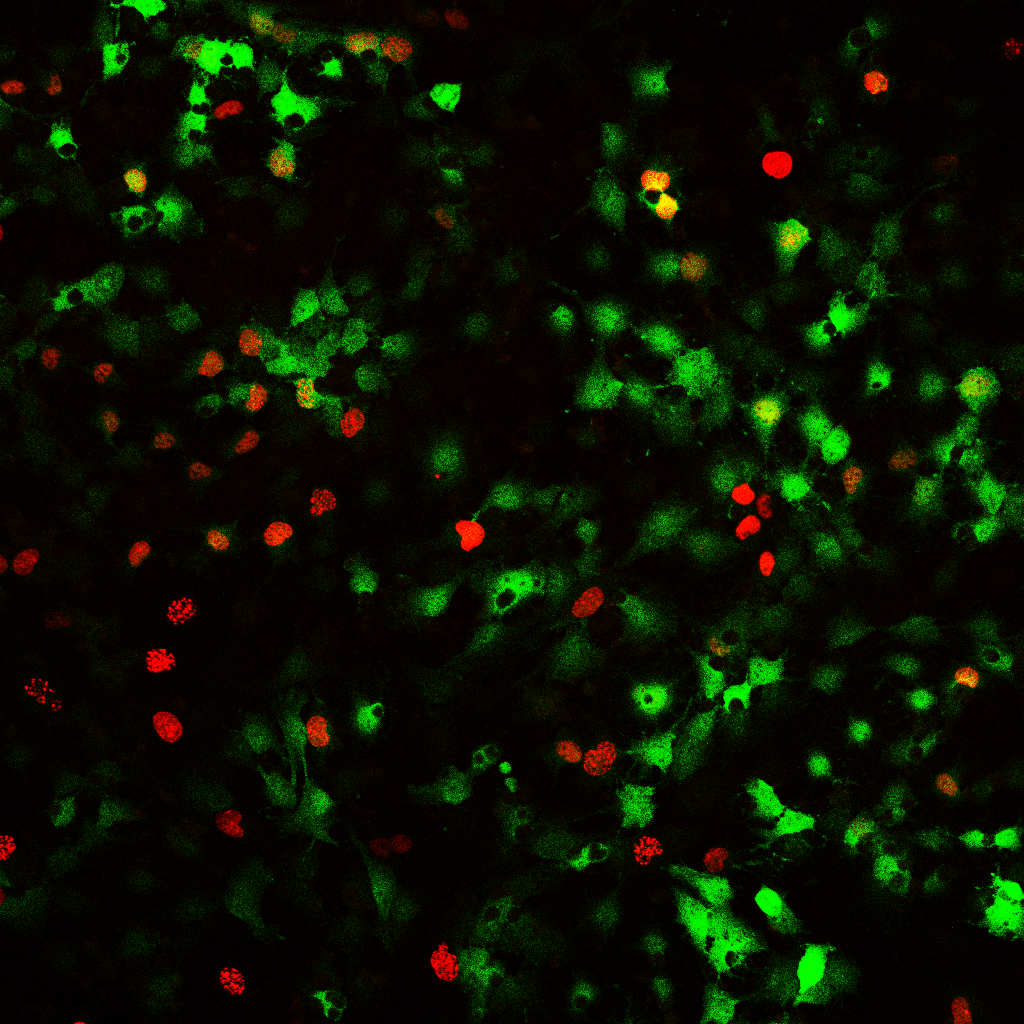

Supplement: Supplementary file 6 — Source data Fig. 4 [file 44319_2024_205_MOESM6_ESM.zip › Source_data_Figure4/4H/siControl + Stat5b/Merge.tif]

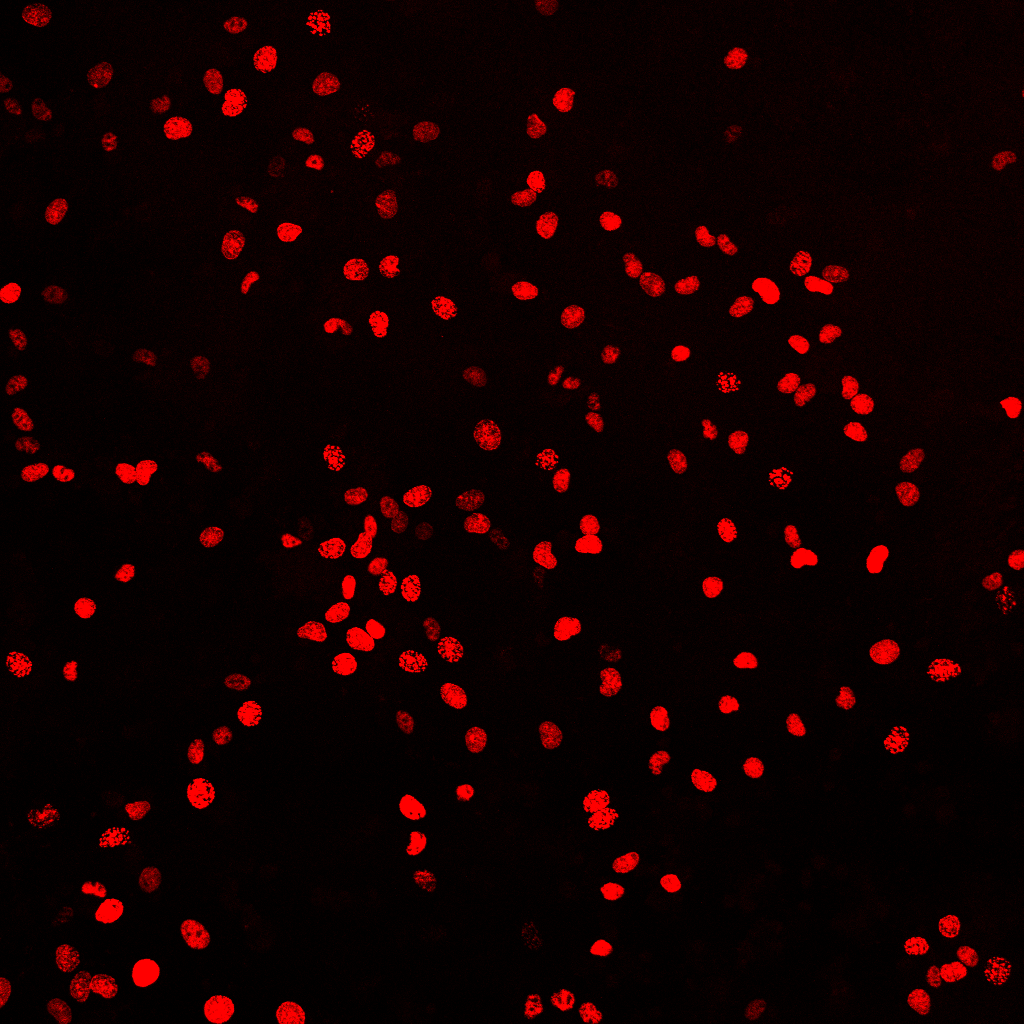

Supplement: Supplementary file 6 — Source data Fig. 4 [file 44319_2024_205_MOESM6_ESM.zip › Source_data_Figure4/4H/siDerl1 + Control/EdU.tif]

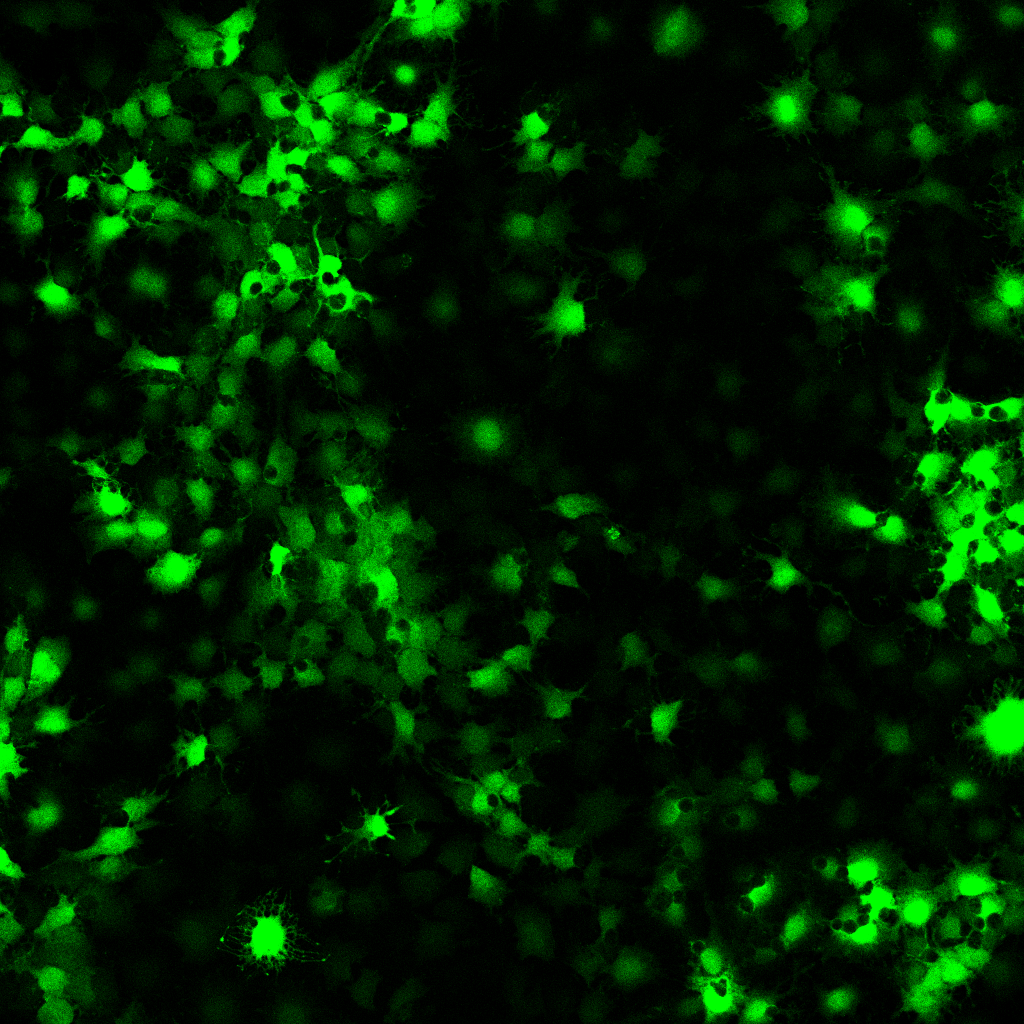

Supplement: Supplementary file 6 — Source data Fig. 4 [file 44319_2024_205_MOESM6_ESM.zip › Source_data_Figure4/4H/siDerl1 + Control/GFP.tif]

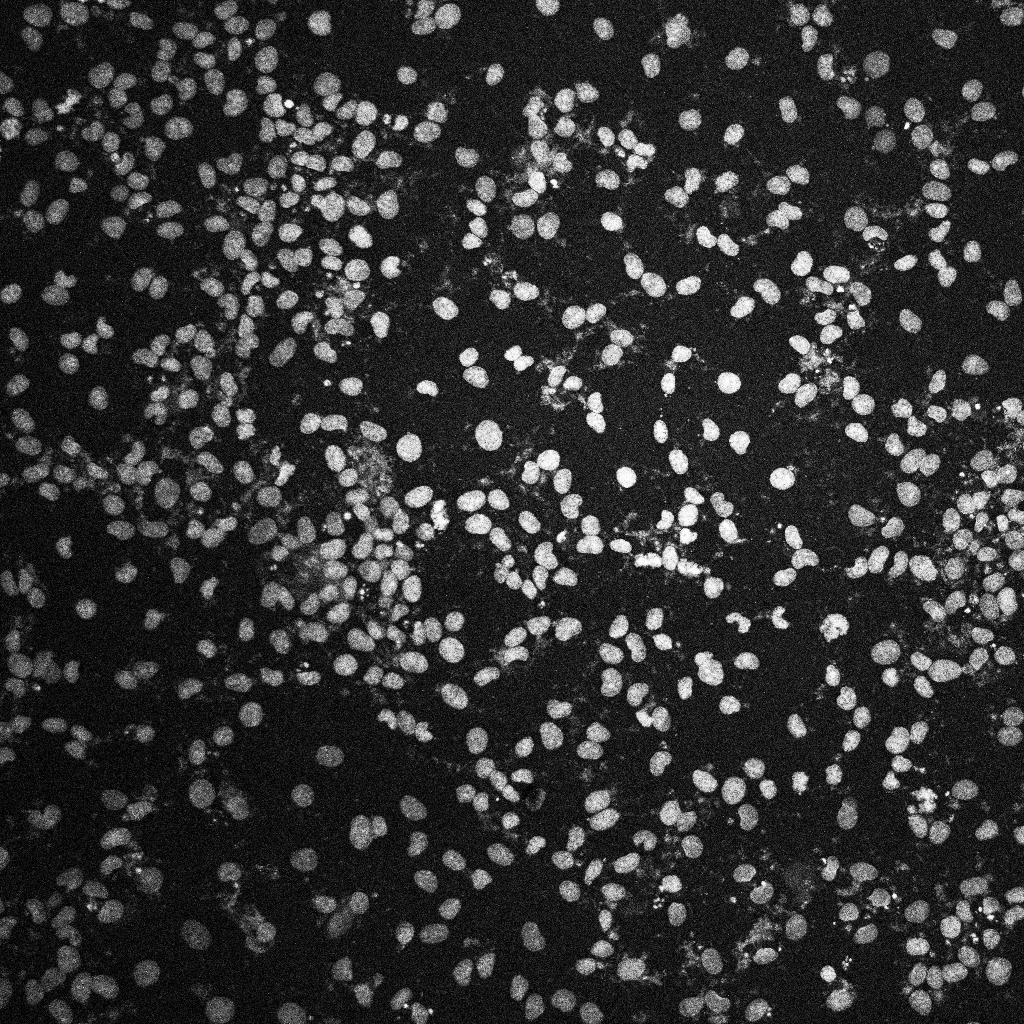

Supplement: Supplementary file 6 — Source data Fig. 4 [file 44319_2024_205_MOESM6_ESM.zip › Source_data_Figure4/4H/siDerl1 + Control/Hoechst.tif]

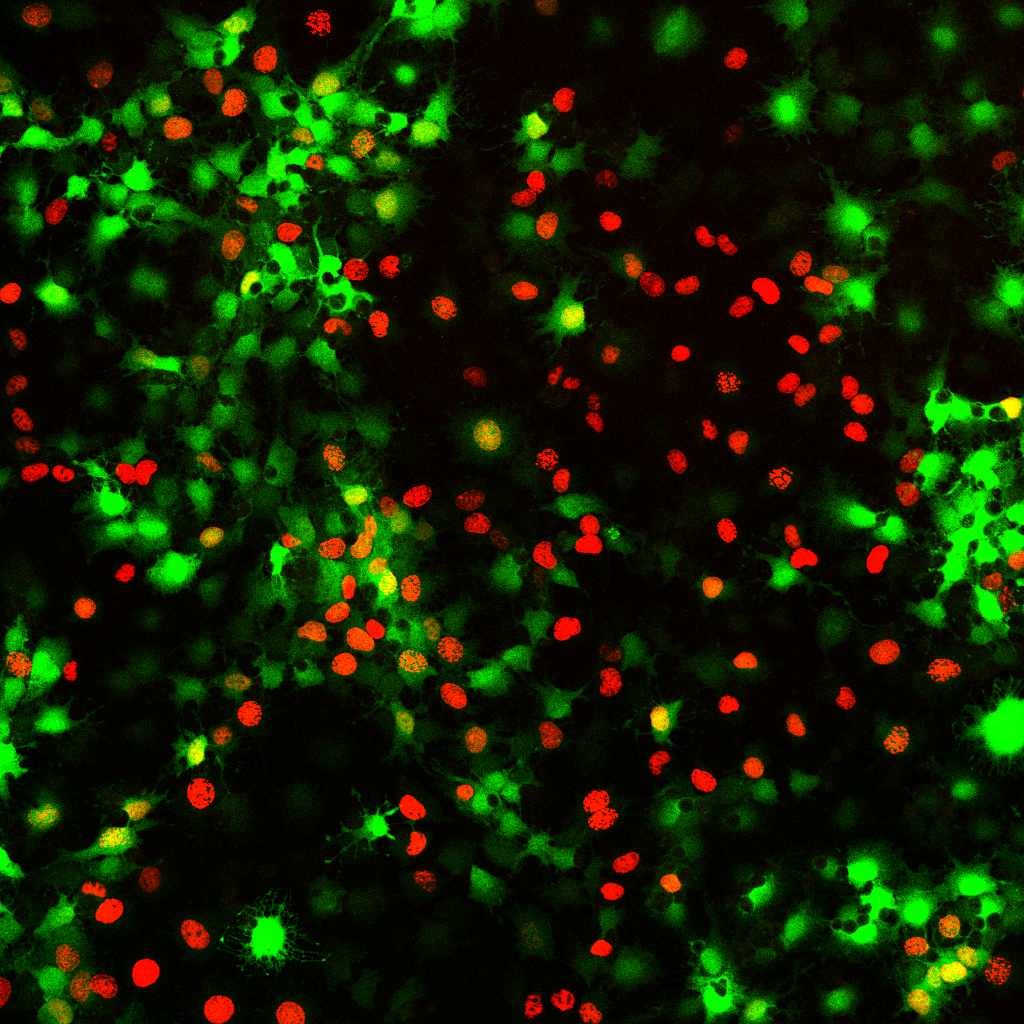

Supplement: Supplementary file 6 — Source data Fig. 4 [file 44319_2024_205_MOESM6_ESM.zip › Source_data_Figure4/4H/siDerl1 + Control/Merge.tif]

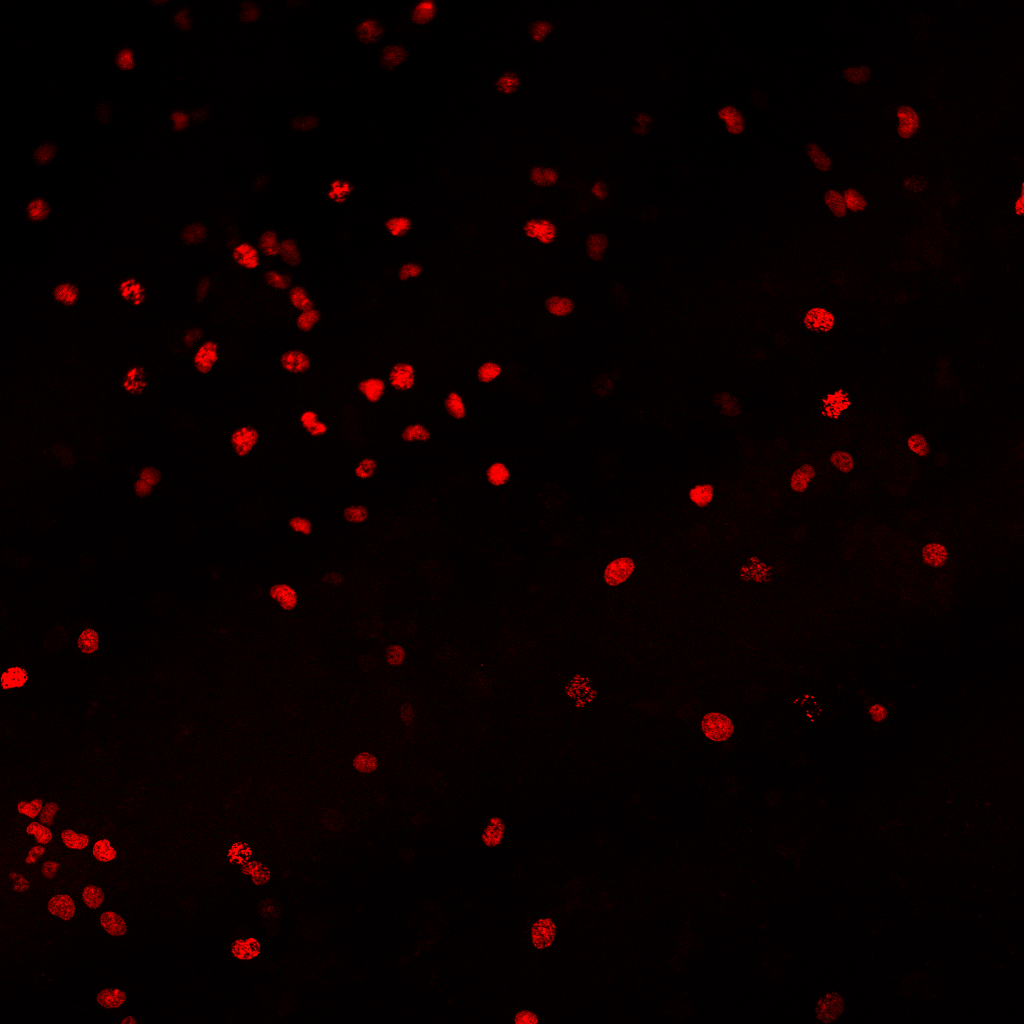

Supplement: Supplementary file 6 — Source data Fig. 4 [file 44319_2024_205_MOESM6_ESM.zip › Source_data_Figure4/4H/siControl + Control/EdU.tif]

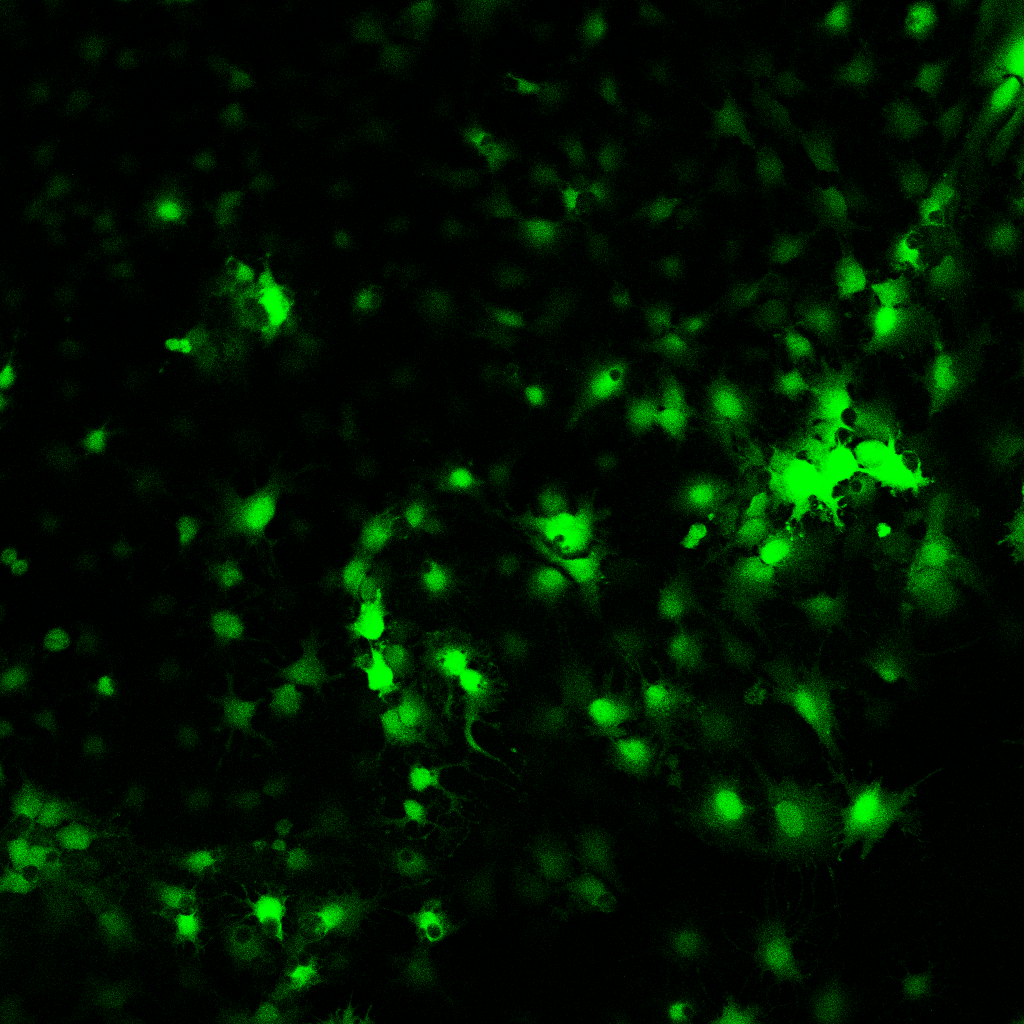

Supplement: Supplementary file 6 — Source data Fig. 4 [file 44319_2024_205_MOESM6_ESM.zip › Source_data_Figure4/4H/siControl + Control/GFP.tif]

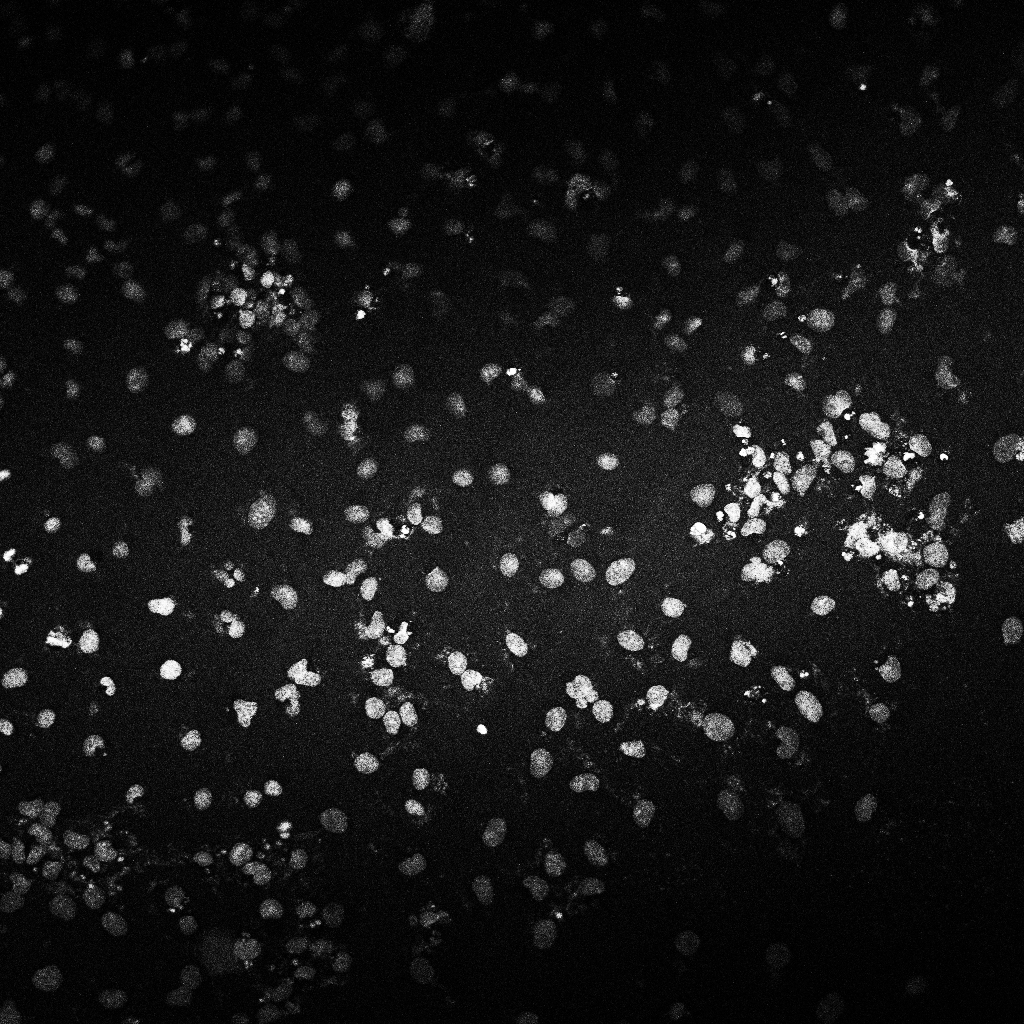

Supplement: Supplementary file 6 — Source data Fig. 4 [file 44319_2024_205_MOESM6_ESM.zip › Source_data_Figure4/4H/siControl + Control/Hoechst.tif]

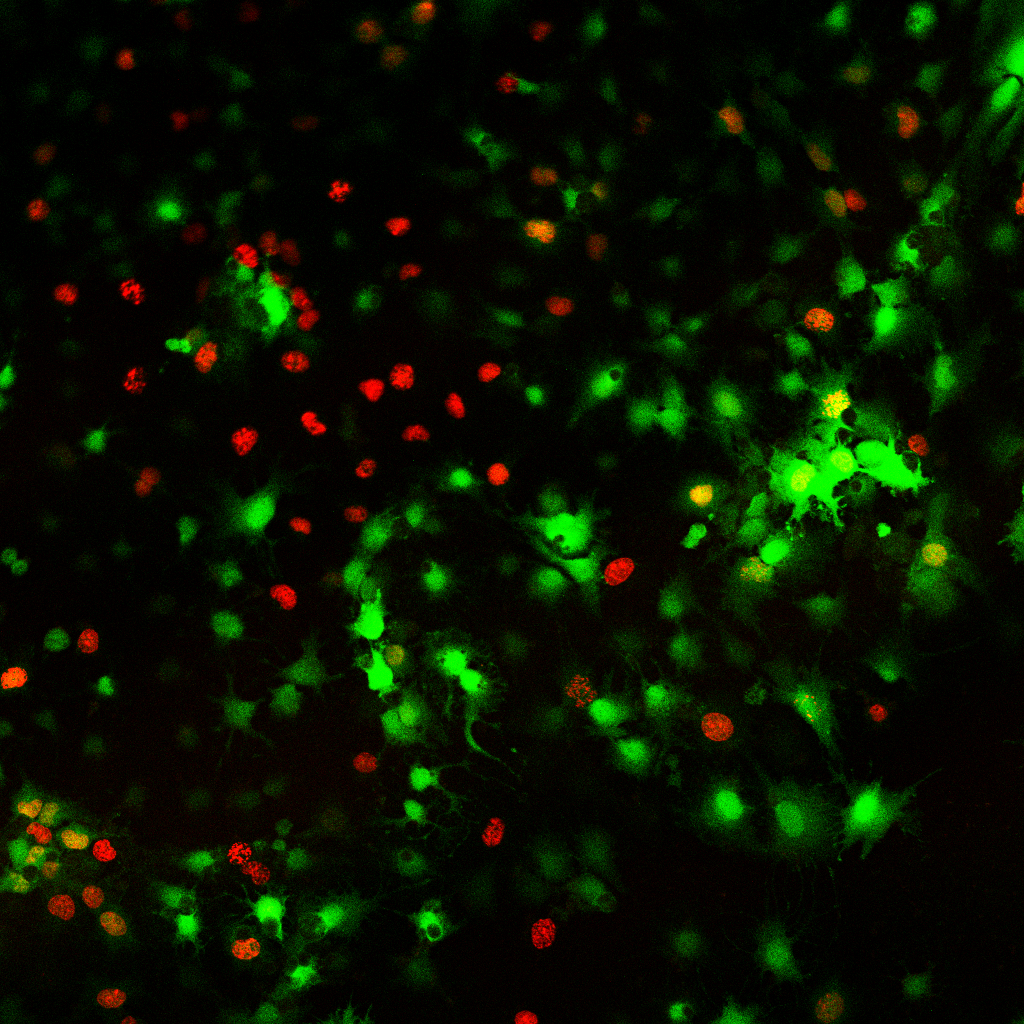

Supplement: Supplementary file 6 — Source data Fig. 4 [file 44319_2024_205_MOESM6_ESM.zip › Source_data_Figure4/4H/siControl + Control/Merge.tif]

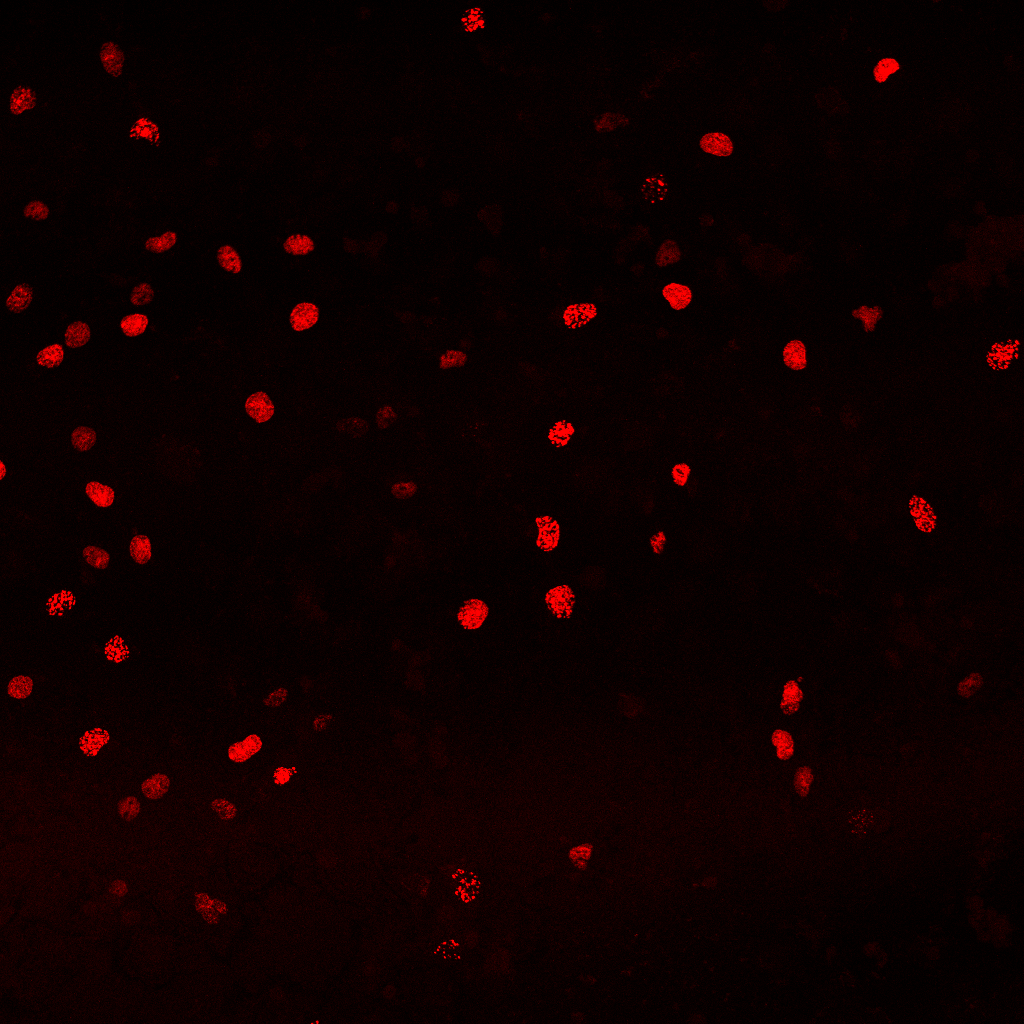

Supplement: Supplementary file 6 — Source data Fig. 4 [file 44319_2024_205_MOESM6_ESM.zip › Source_data_Figure4/4H/siDerl1 + Stat5b/EdU.tif]

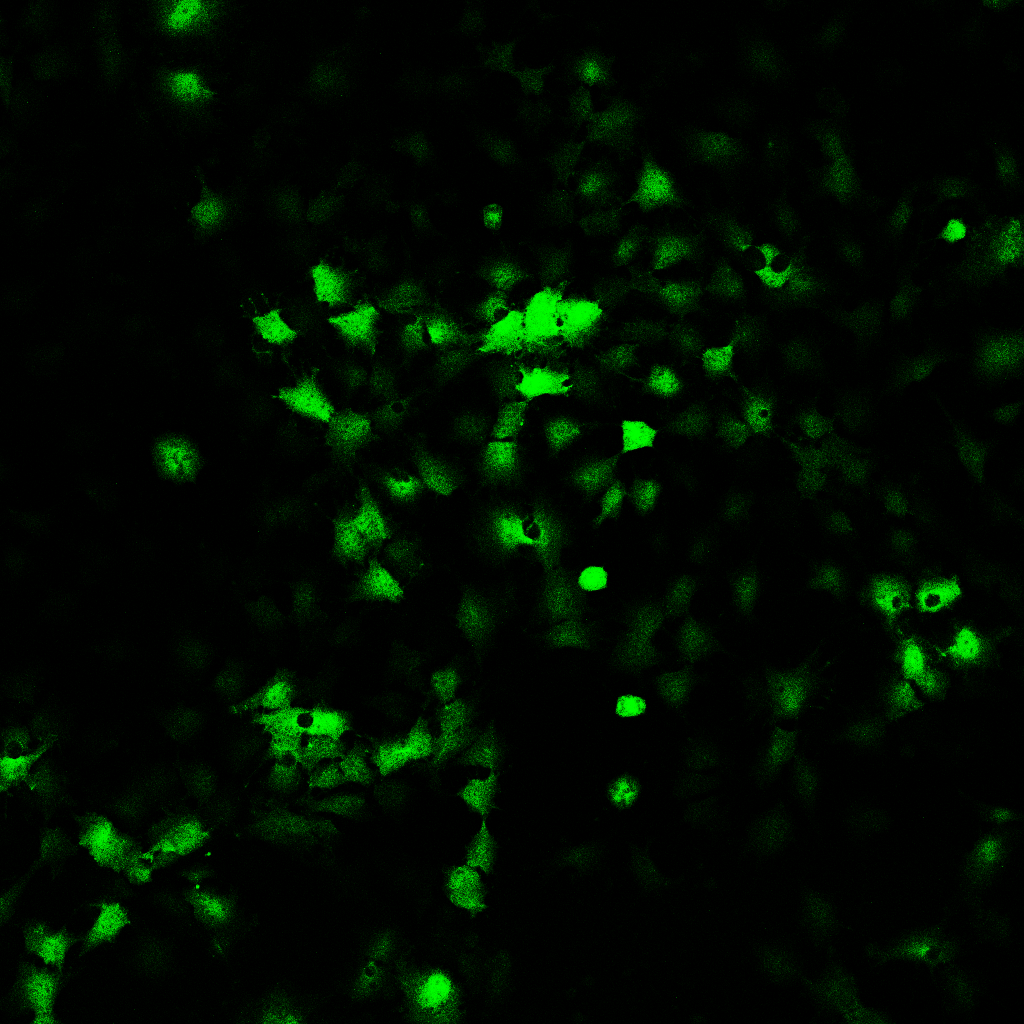

Supplement: Supplementary file 6 — Source data Fig. 4 [file 44319_2024_205_MOESM6_ESM.zip › Source_data_Figure4/4H/siDerl1 + Stat5b/GFP.tif]

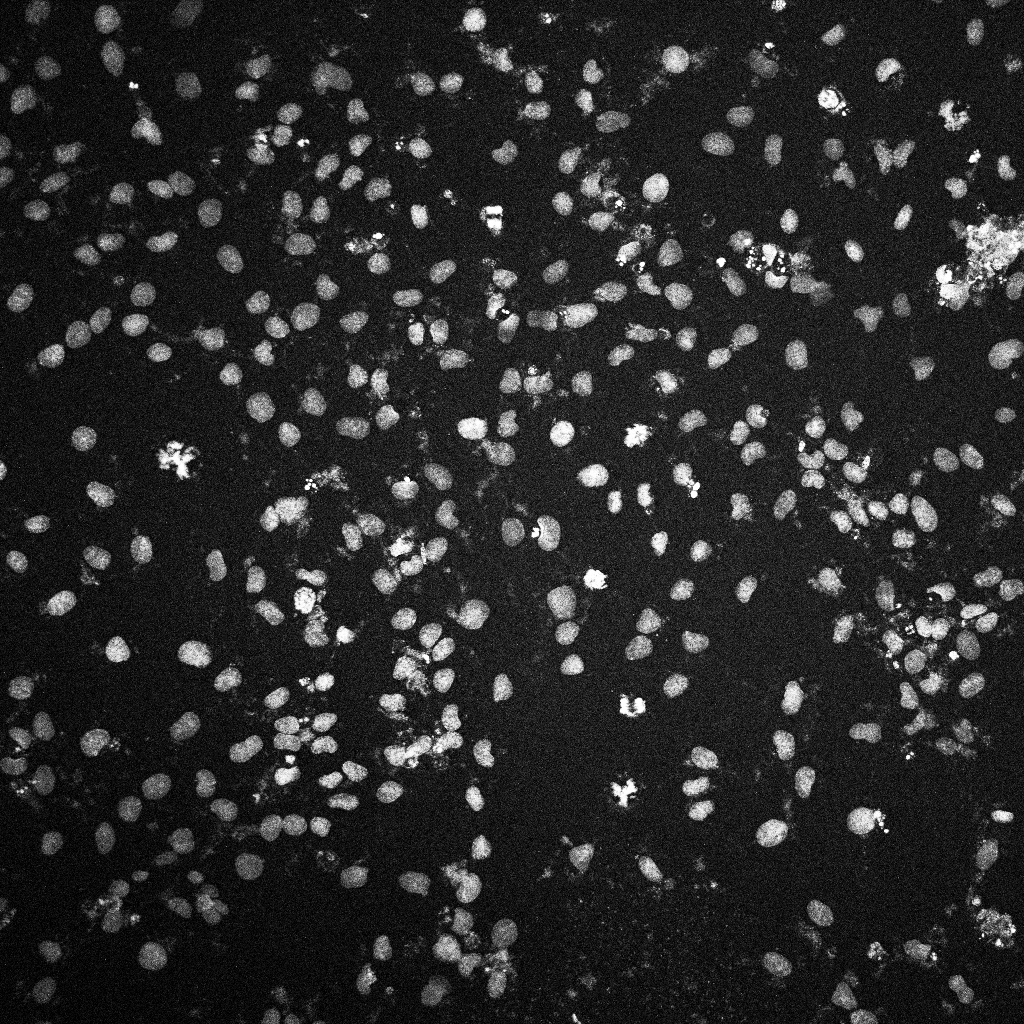

Supplement: Supplementary file 6 — Source data Fig. 4 [file 44319_2024_205_MOESM6_ESM.zip › Source_data_Figure4/4H/siDerl1 + Stat5b/Hoechst.tif]

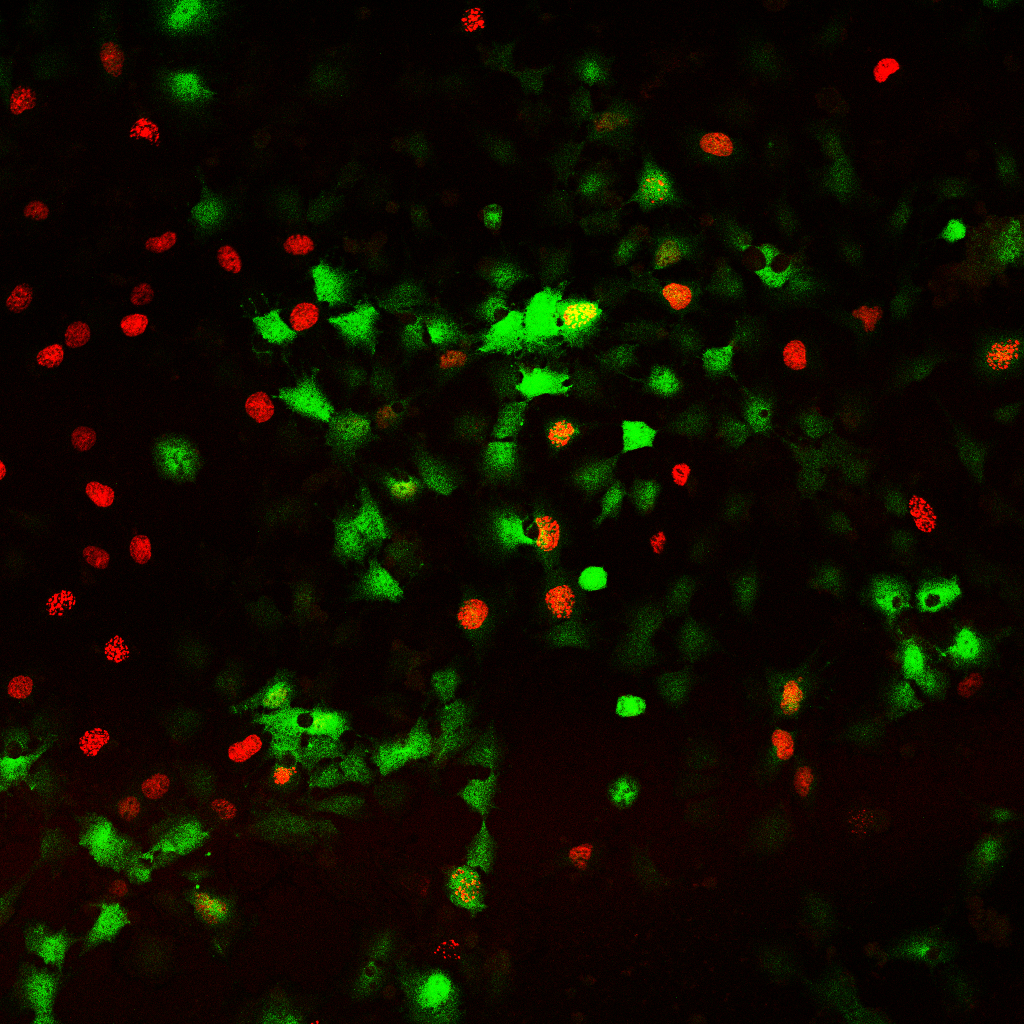

Supplement: Supplementary file 6 — Source data Fig. 4 [file 44319_2024_205_MOESM6_ESM.zip › Source_data_Figure4/4H/siDerl1 + Stat5b/Merge.tif]

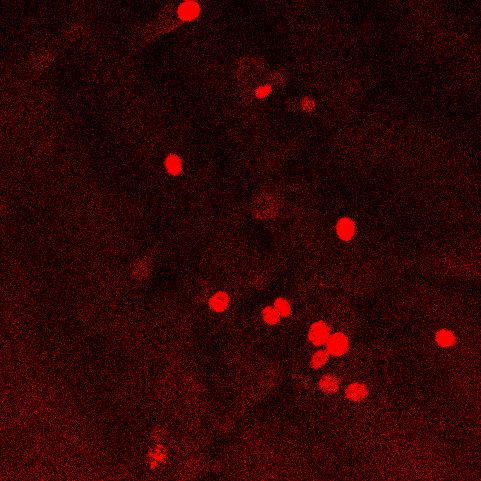

Supplement: Supplementary file 7 — Source data Fig. 5 [file 44319_2024_205_MOESM7_ESM.zip › Source_data_Figure5/5F/siDerl1,siControl + vehicle/EdU.tif]

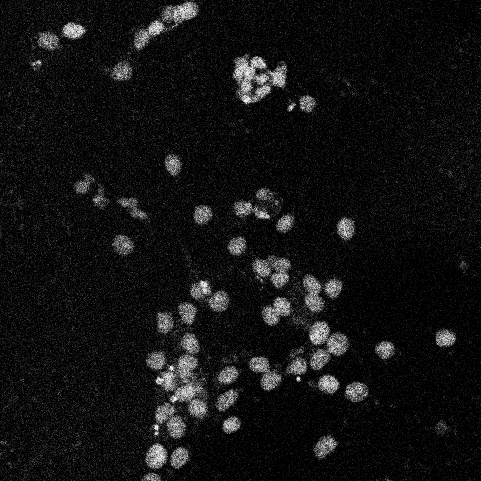

Supplement: Supplementary file 7 — Source data Fig. 5 [file 44319_2024_205_MOESM7_ESM.zip › Source_data_Figure5/5F/siDerl1,siControl + vehicle/Hoechst.tif]

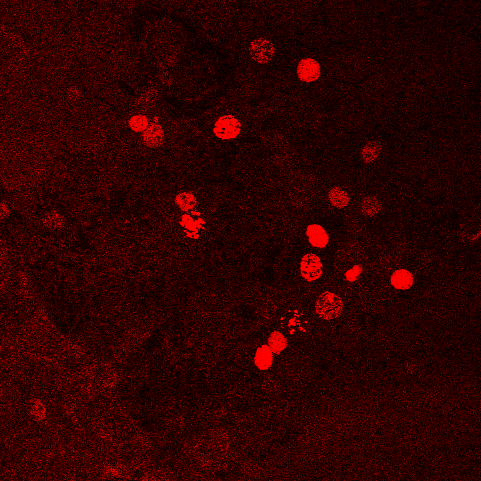

Supplement: Supplementary file 7 — Source data Fig. 5 [file 44319_2024_205_MOESM7_ESM.zip › Source_data_Figure5/5F/siDerl1,siStat5b + vehicle/EdU.tif]

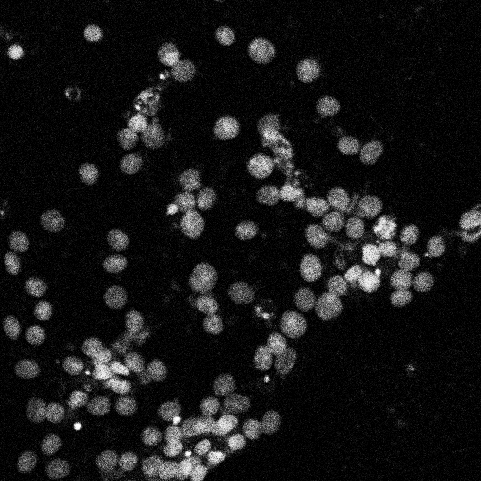

Supplement: Supplementary file 7 — Source data Fig. 5 [file 44319_2024_205_MOESM7_ESM.zip › Source_data_Figure5/5F/siDerl1,siStat5b + vehicle/Hoechst.tif]

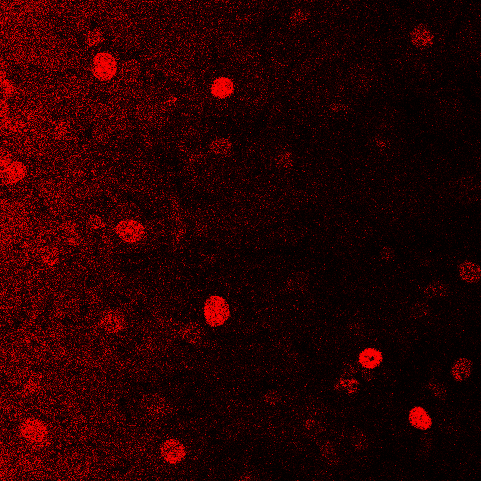

Supplement: Supplementary file 7 — Source data Fig. 5 [file 44319_2024_205_MOESM7_ESM.zip › Source_data_Figure5/5F/siDerl1,siStat5b + 4-PBA/EdU.tif]

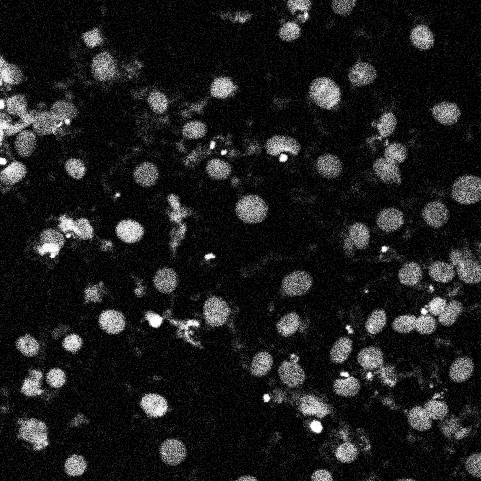

Supplement: Supplementary file 7 — Source data Fig. 5 [file 44319_2024_205_MOESM7_ESM.zip › Source_data_Figure5/5F/siDerl1,siStat5b + 4-PBA/Hoechst.tif]
