# Supplementary figures and images for: The Derlin-1-Stat5b axis maintains homeostasis of adult hippocampal neurogenesis (part 2 of 2)
Source: EMBO Rep. 2024 Jul 30;25(8):26. doi: 10.1038/s44319-024-00205-7 (PMC11316036; doi:10.1038/s44319-024-00205-7)

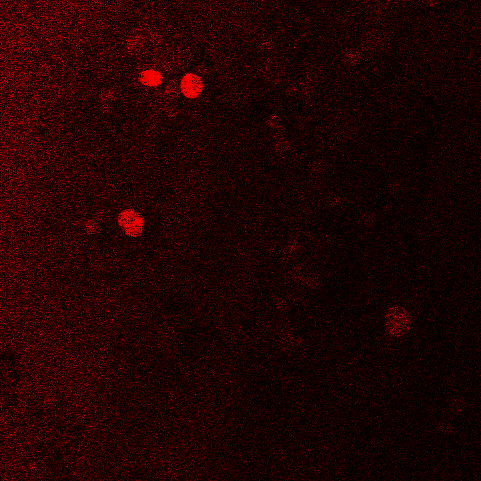

Supplement: Supplementary file 7 — Source data Fig. 5 [file 44319_2024_205_MOESM7_ESM.zip › Source_data_Figure5/5F/siDerl1,siControl + 4-PBA/EdU.tif]

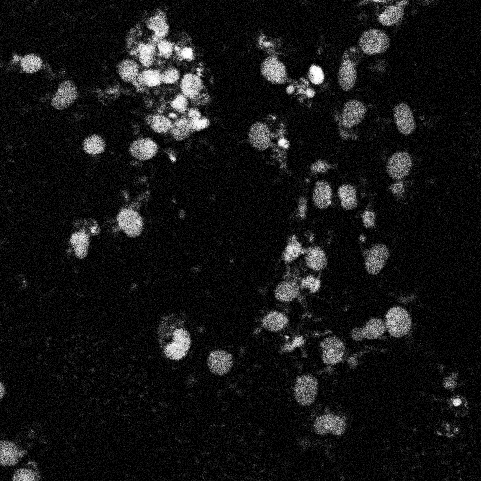

Supplement: Supplementary file 7 — Source data Fig. 5 [file 44319_2024_205_MOESM7_ESM.zip › Source_data_Figure5/5F/siDerl1,siControl + 4-PBA/Hoechst.tif]

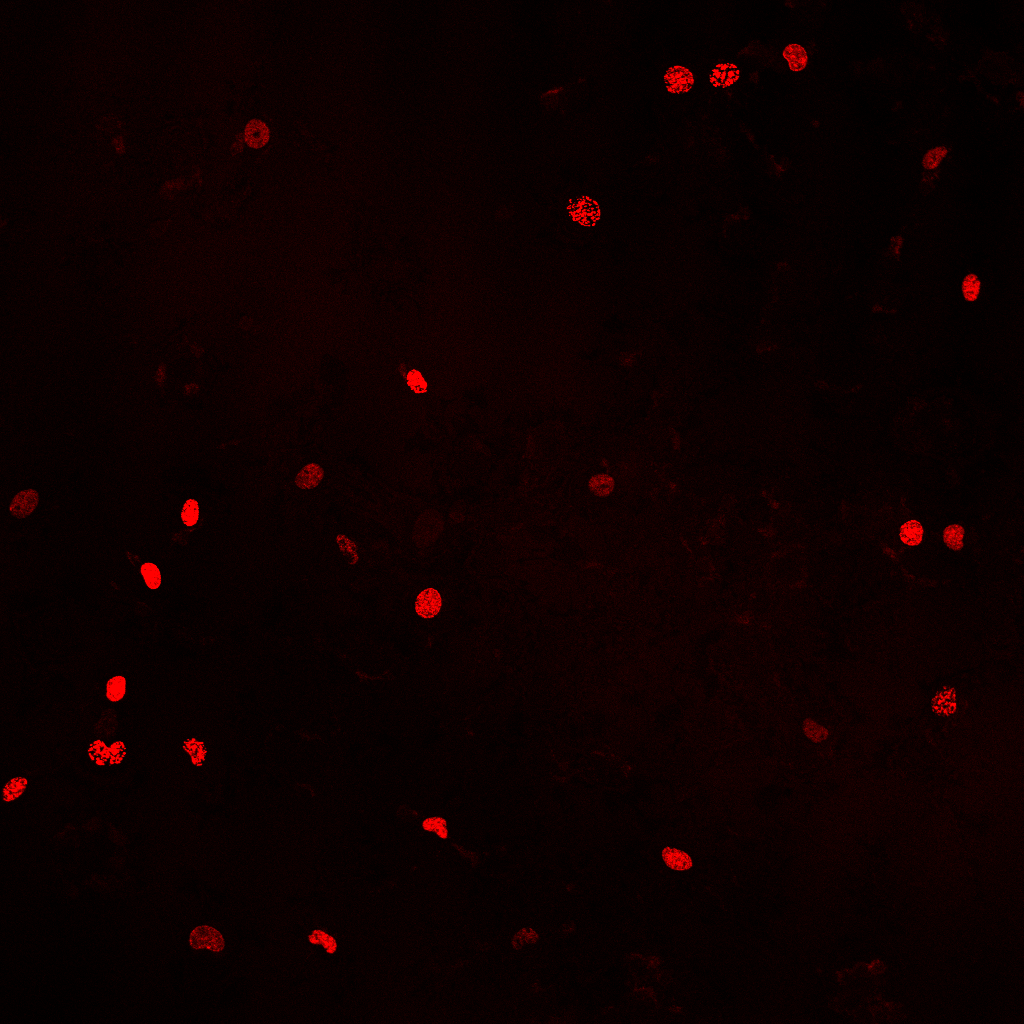

Supplement: Supplementary file 7 — Source data Fig. 5 [file 44319_2024_205_MOESM7_ESM.zip › Source_data_Figure5/5B/siControl + vehicle/EdU.tif]

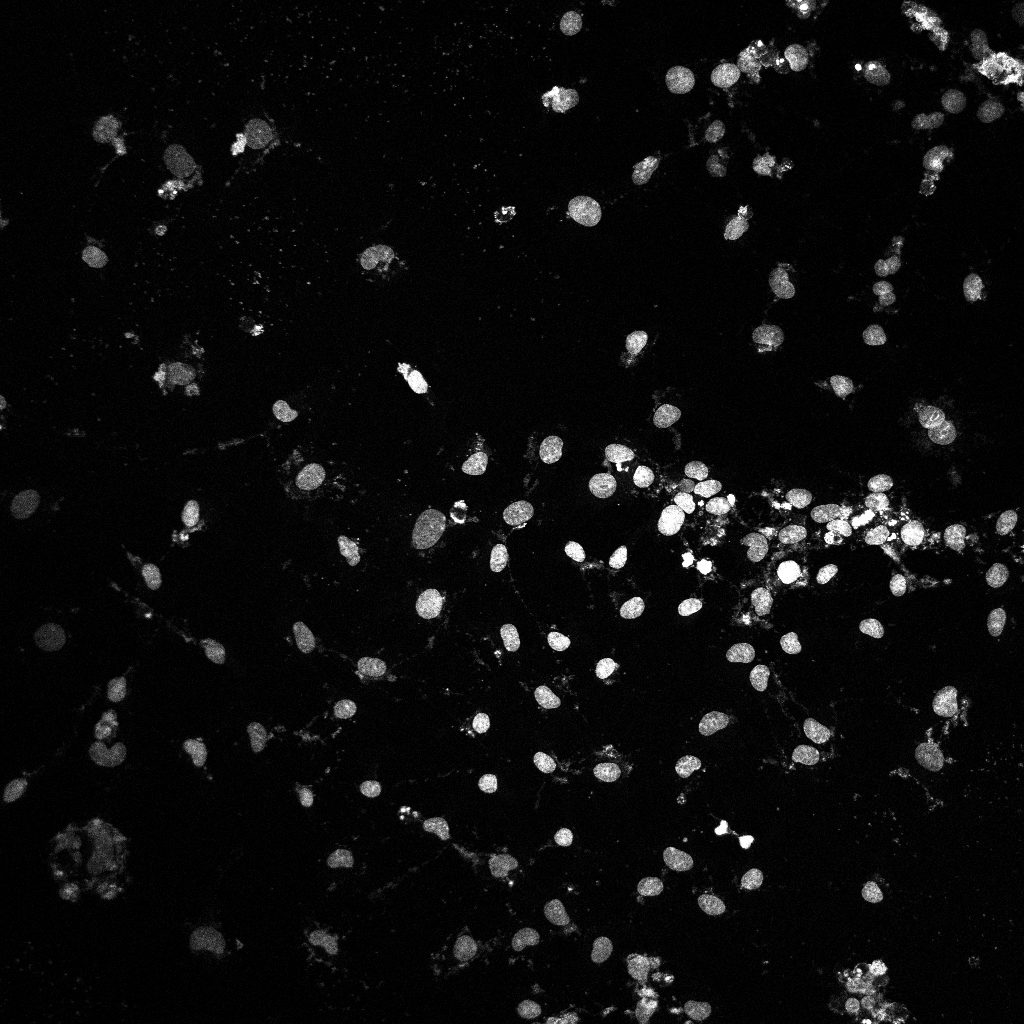

Supplement: Supplementary file 7 — Source data Fig. 5 [file 44319_2024_205_MOESM7_ESM.zip › Source_data_Figure5/5B/siControl + vehicle/Hoechst.tif]

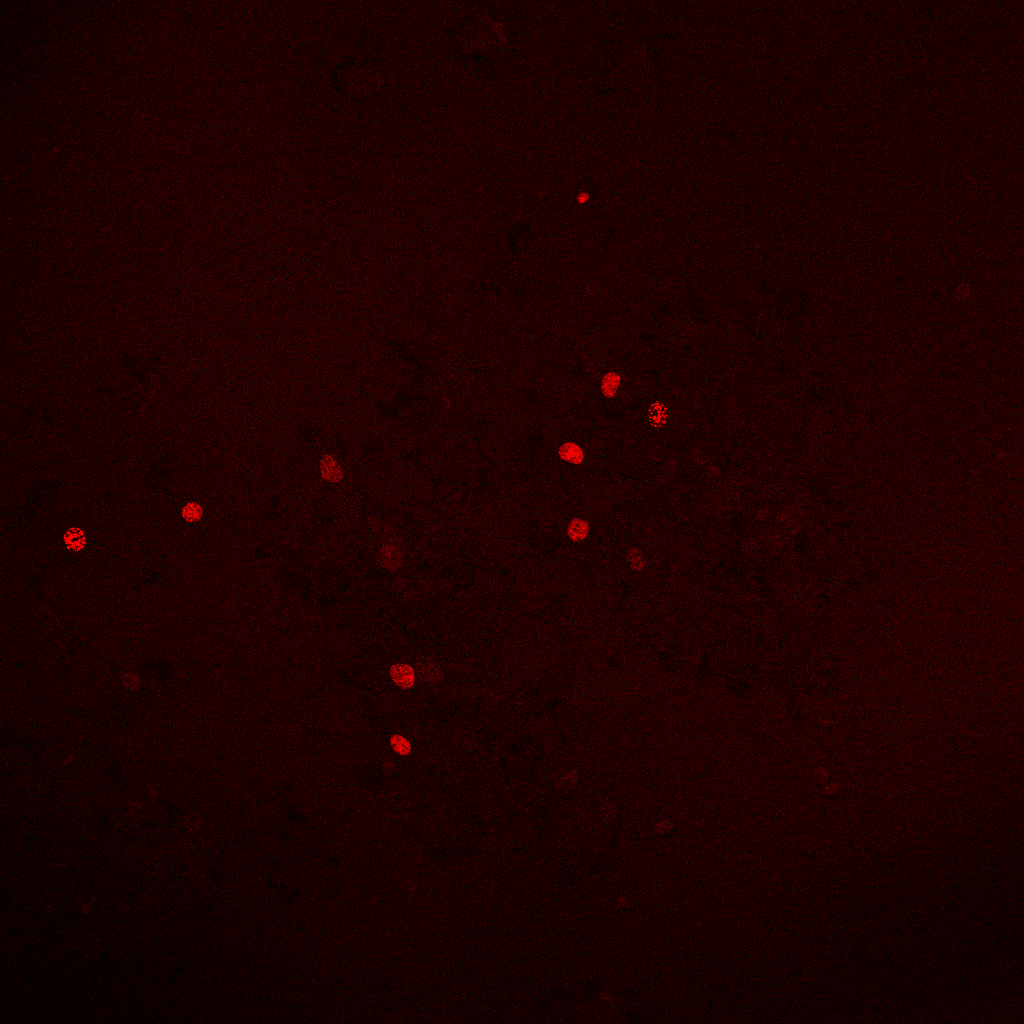

Supplement: Supplementary file 7 — Source data Fig. 5 [file 44319_2024_205_MOESM7_ESM.zip › Source_data_Figure5/5B/siControl + 4-PBA/EdU.tif]

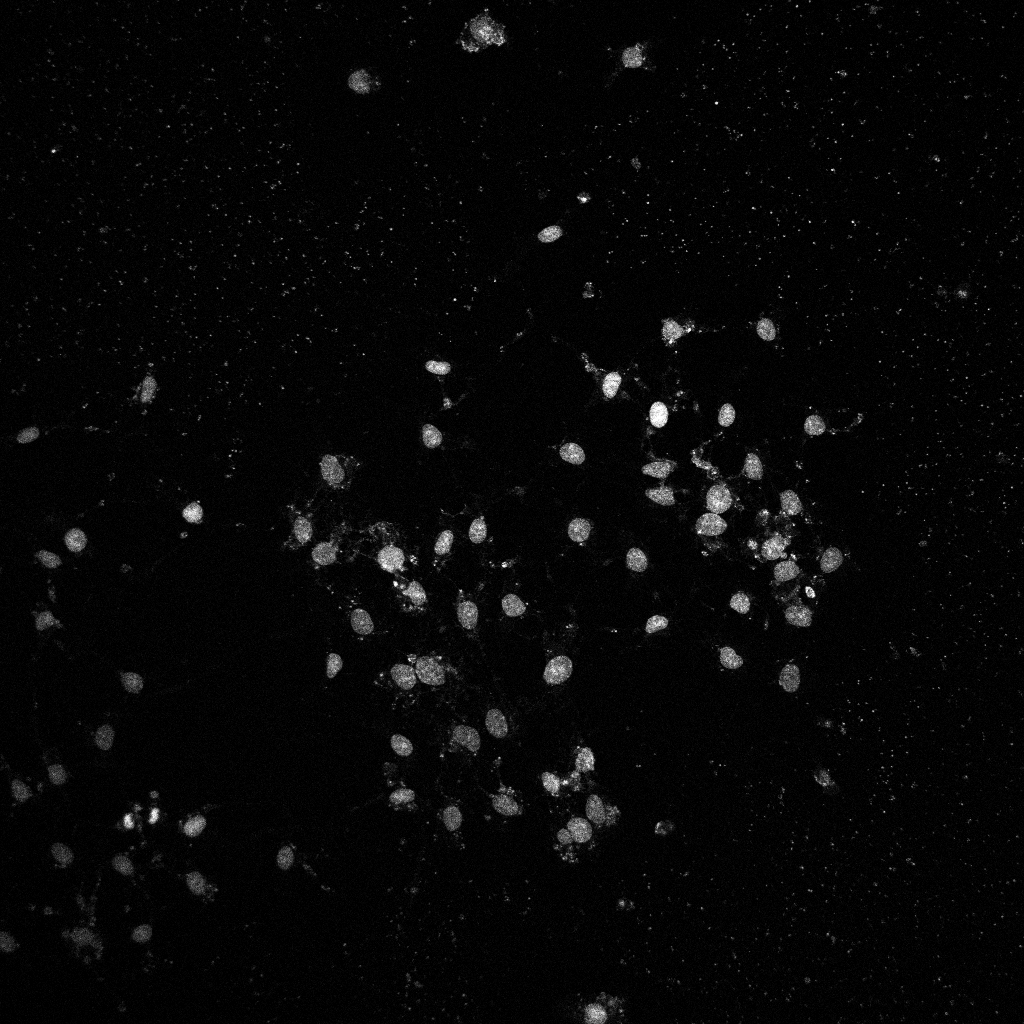

Supplement: Supplementary file 7 — Source data Fig. 5 [file 44319_2024_205_MOESM7_ESM.zip › Source_data_Figure5/5B/siControl + 4-PBA/Hoechst.tif]

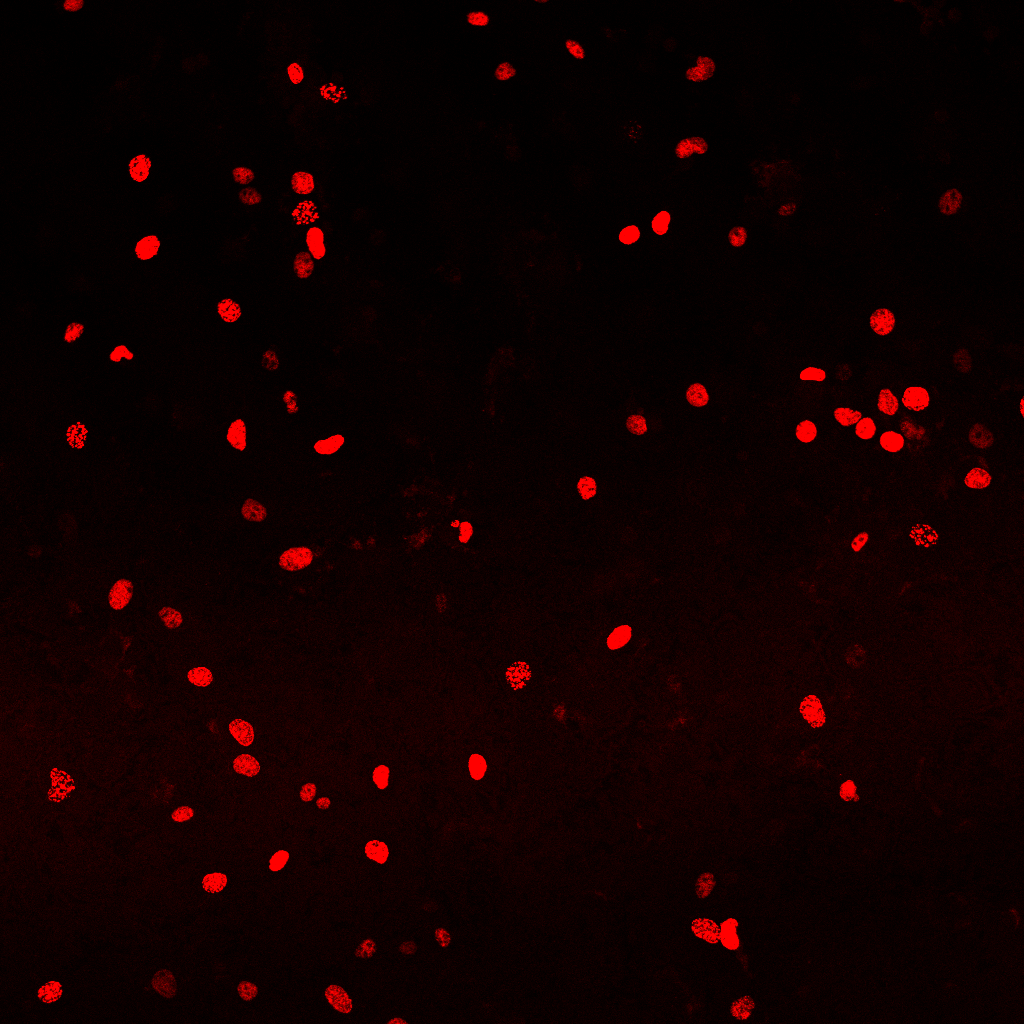

Supplement: Supplementary file 7 — Source data Fig. 5 [file 44319_2024_205_MOESM7_ESM.zip › Source_data_Figure5/5B/siDerl1 + vehicle/EdU.tif]

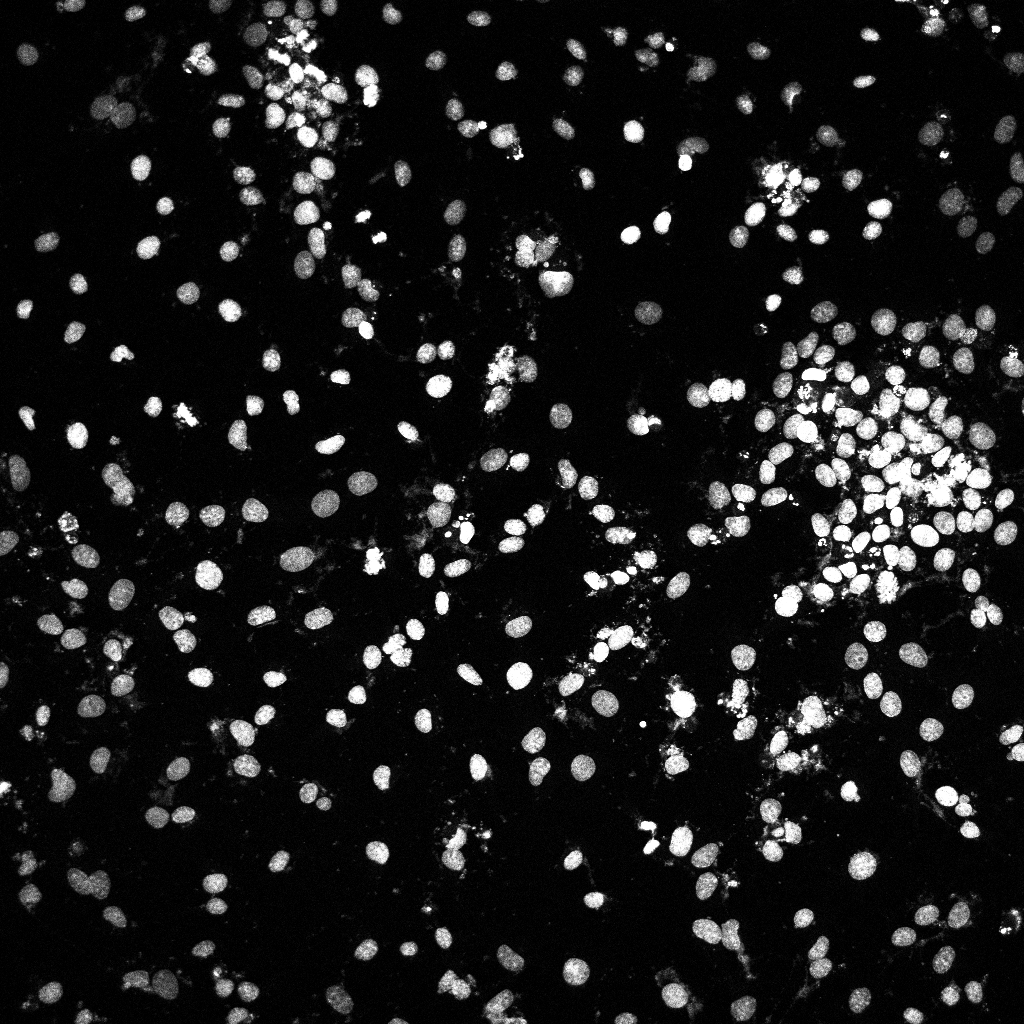

Supplement: Supplementary file 7 — Source data Fig. 5 [file 44319_2024_205_MOESM7_ESM.zip › Source_data_Figure5/5B/siDerl1 + vehicle/Hoechst.tif]

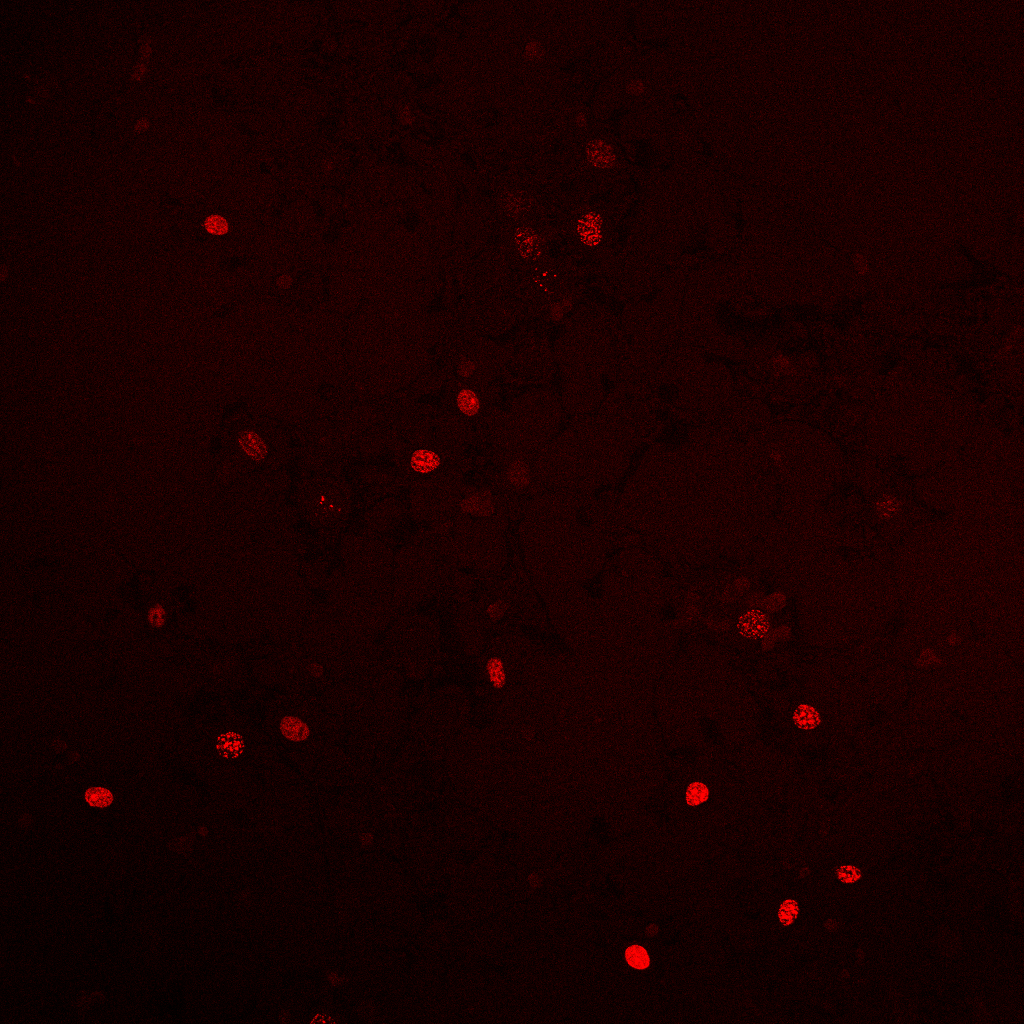

Supplement: Supplementary file 7 — Source data Fig. 5 [file 44319_2024_205_MOESM7_ESM.zip › Source_data_Figure5/5B/siDerl1 + 4-PBA/EdU.tif]

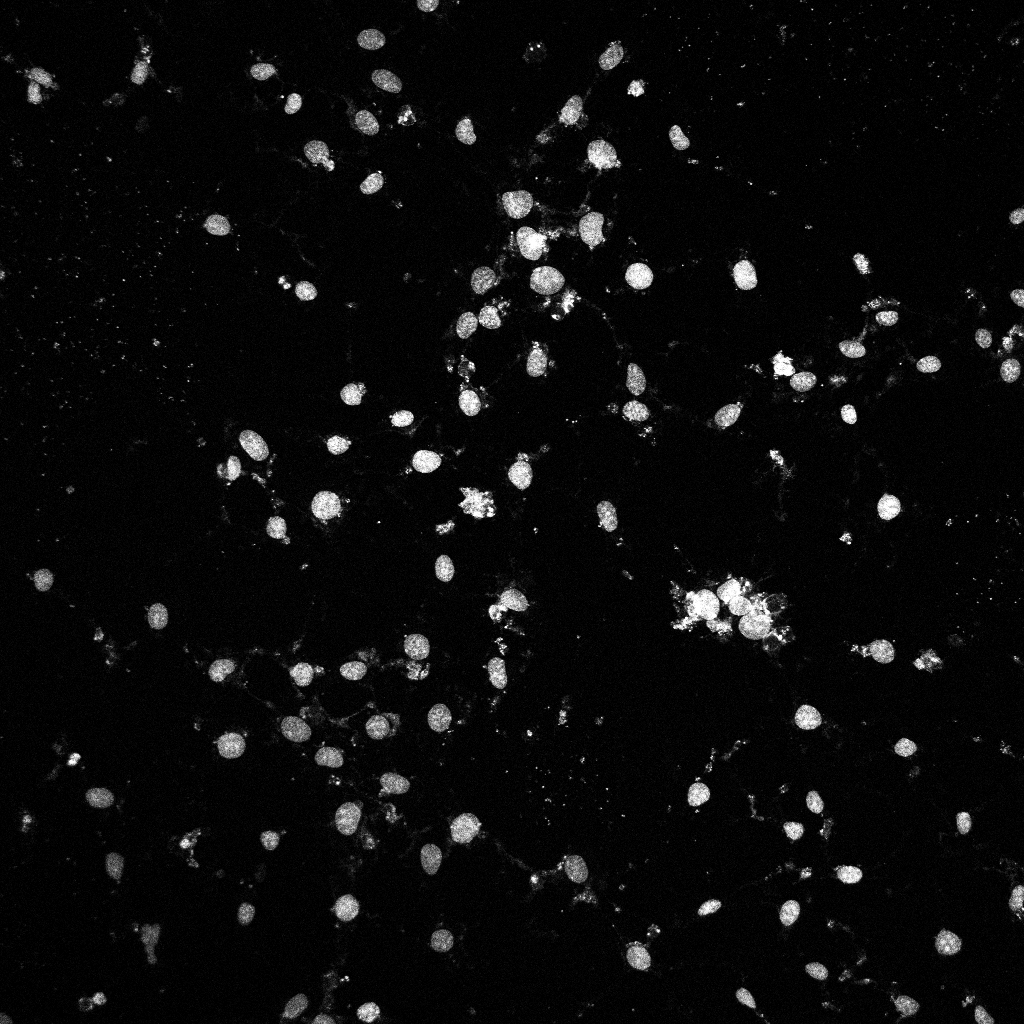

Supplement: Supplementary file 7 — Source data Fig. 5 [file 44319_2024_205_MOESM7_ESM.zip › Source_data_Figure5/5B/siDerl1 + 4-PBA/Hoechst.tif]

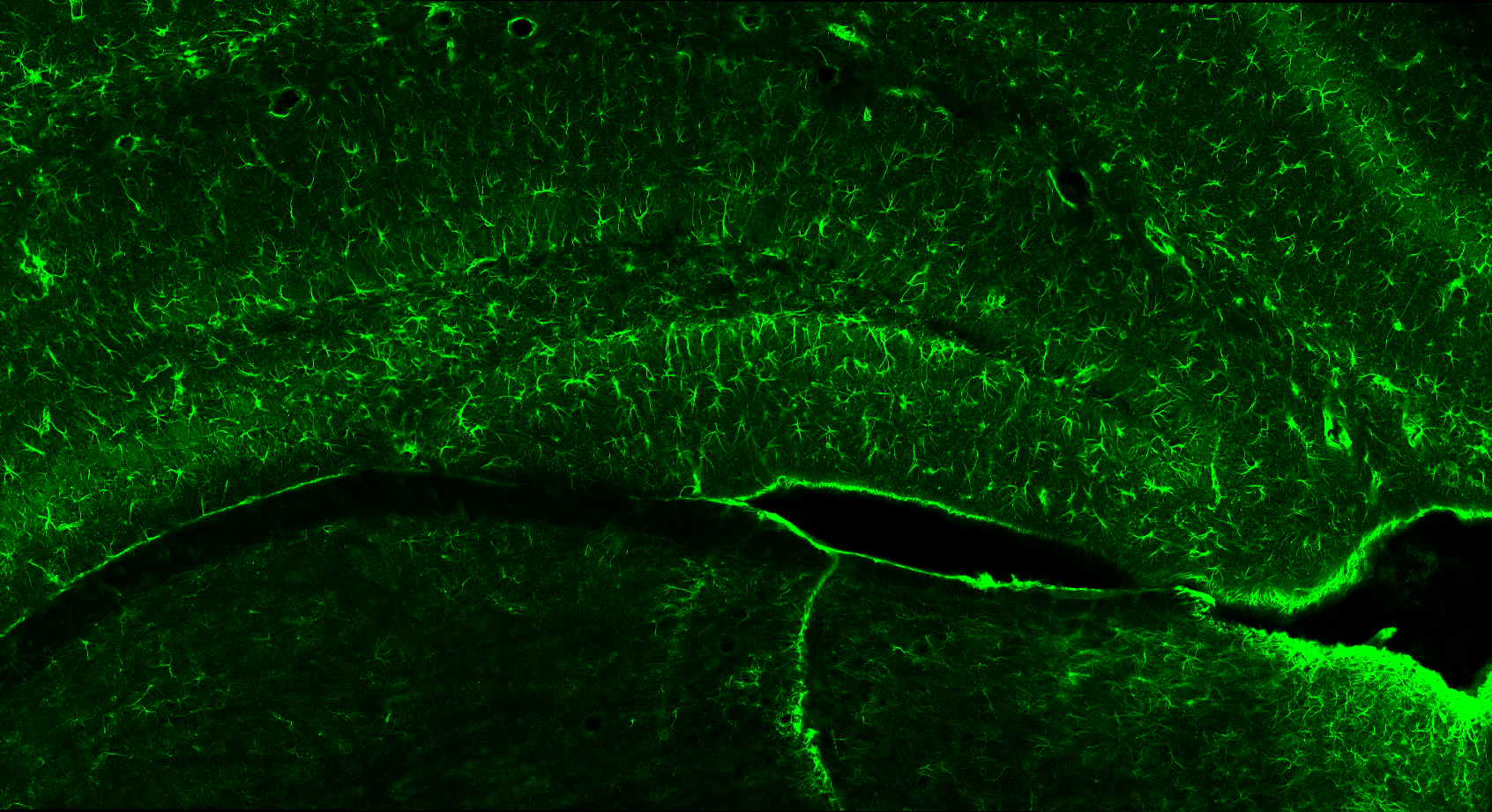

Supplement: Supplementary file 8 — Source data Fig. 6 [file 44319_2024_205_MOESM8_ESM.zip › Source_data_Figure6/6G/Derl1NesCre + vehicle/GFAP.tif]

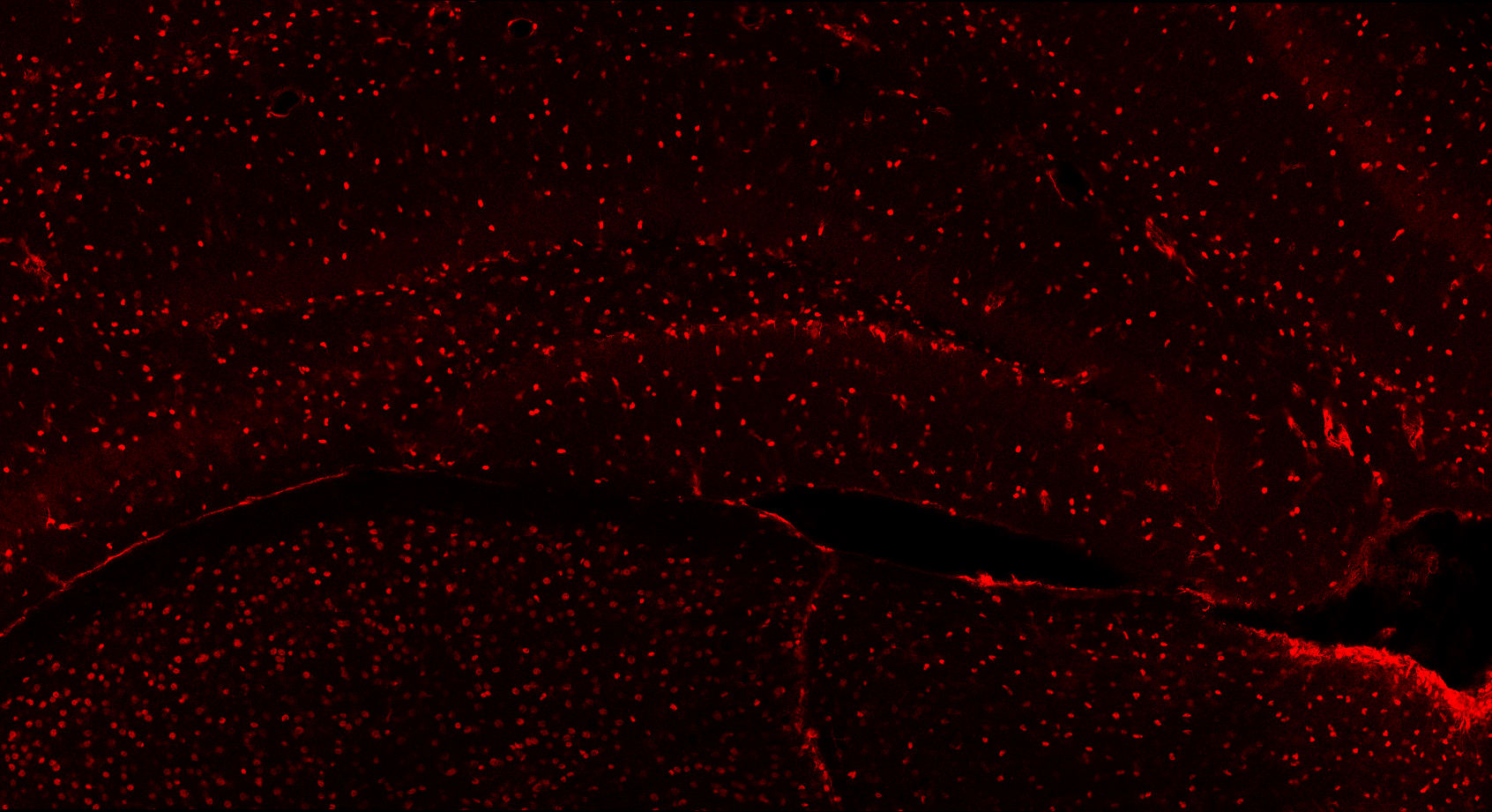

Supplement: Supplementary file 8 — Source data Fig. 6 [file 44319_2024_205_MOESM8_ESM.zip › Source_data_Figure6/6G/Derl1NesCre + vehicle/Sox2.tif]

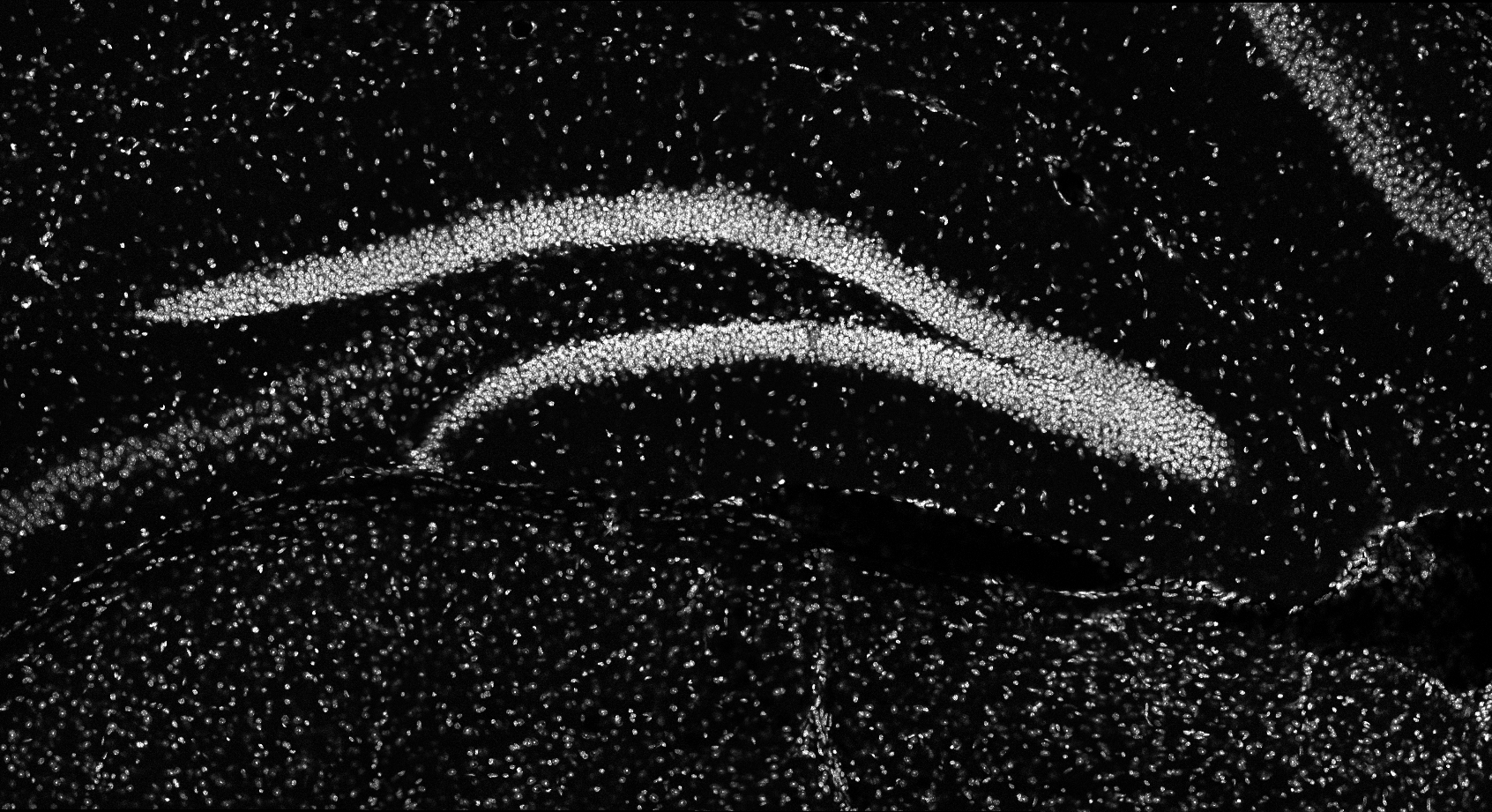

Supplement: Supplementary file 8 — Source data Fig. 6 [file 44319_2024_205_MOESM8_ESM.zip › Source_data_Figure6/6G/Derl1NesCre + vehicle/Hoechst.tif]

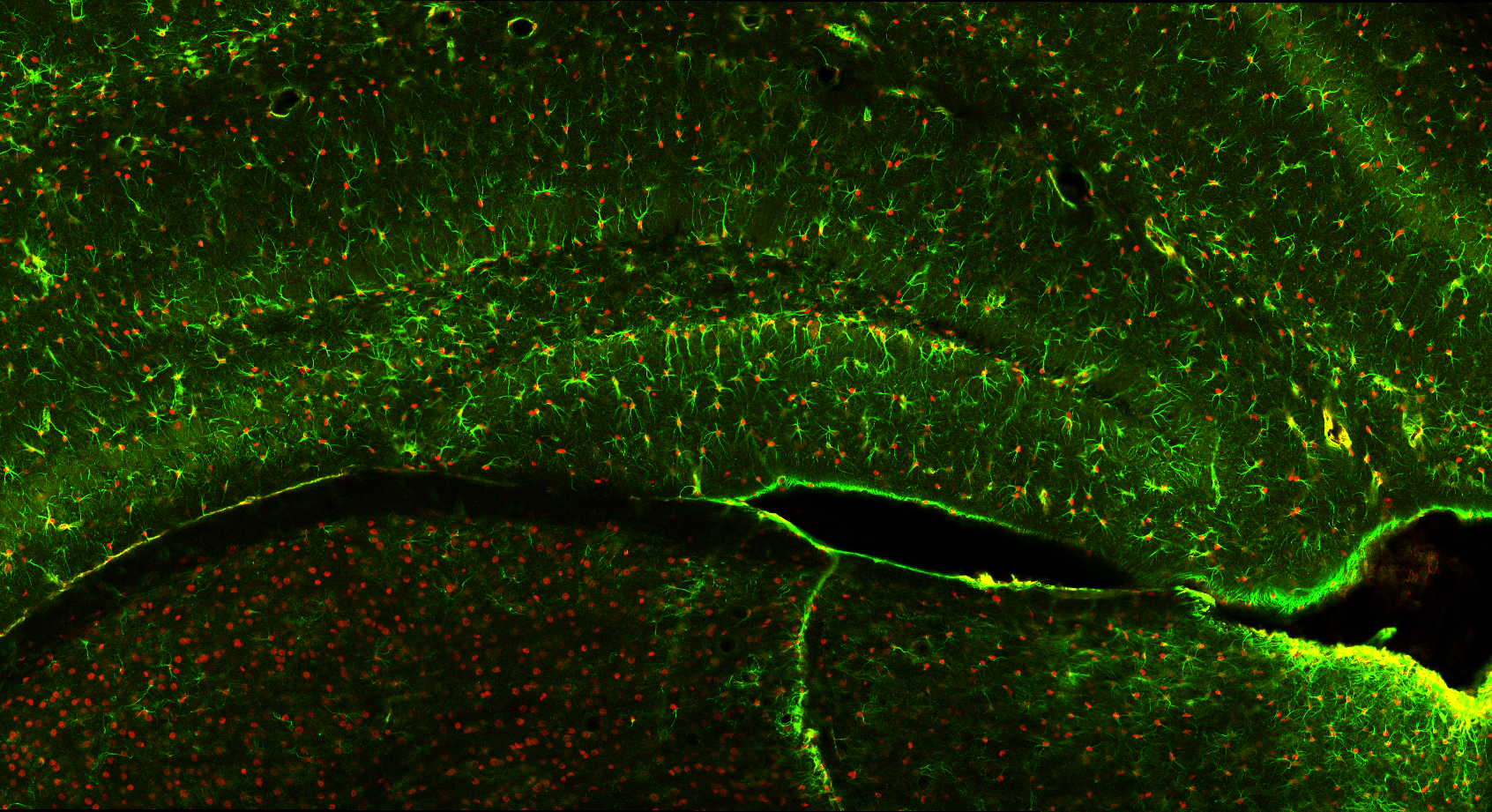

Supplement: Supplementary file 8 — Source data Fig. 6 [file 44319_2024_205_MOESM8_ESM.zip › Source_data_Figure6/6G/Derl1NesCre + vehicle/Merge.tif]

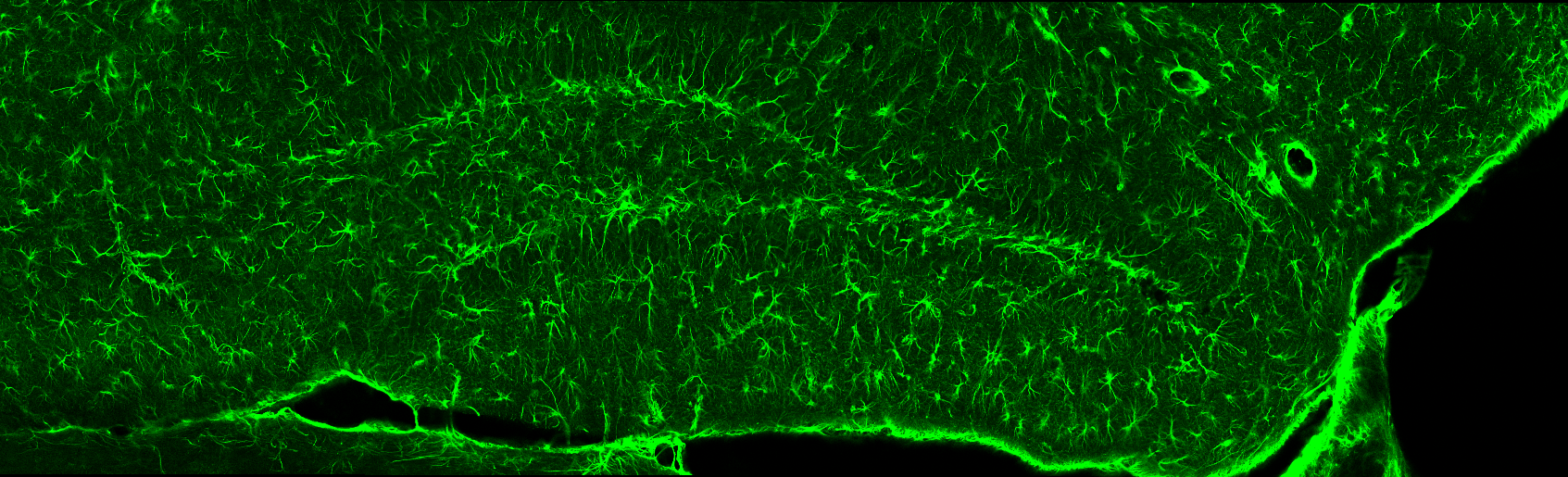

Supplement: Supplementary file 8 — Source data Fig. 6 [file 44319_2024_205_MOESM8_ESM.zip › Source_data_Figure6/6G/Derl1f:f + 4-PBA/GFAP.tif]

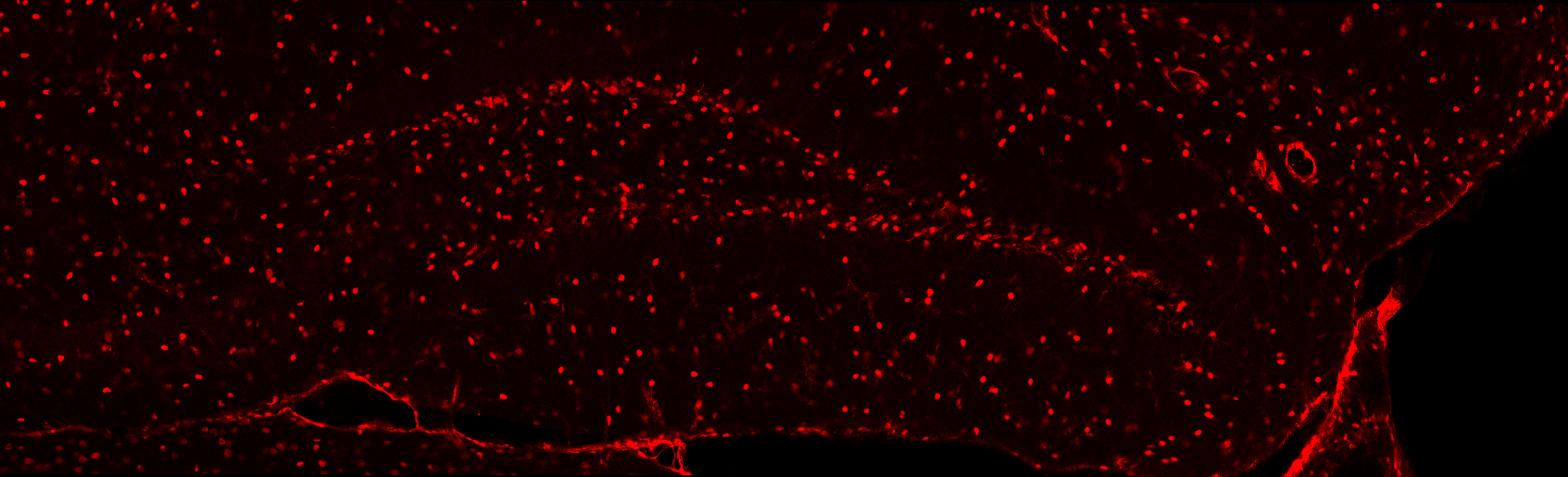

Supplement: Supplementary file 8 — Source data Fig. 6 [file 44319_2024_205_MOESM8_ESM.zip › Source_data_Figure6/6G/Derl1f:f + 4-PBA/Sox2.tif]

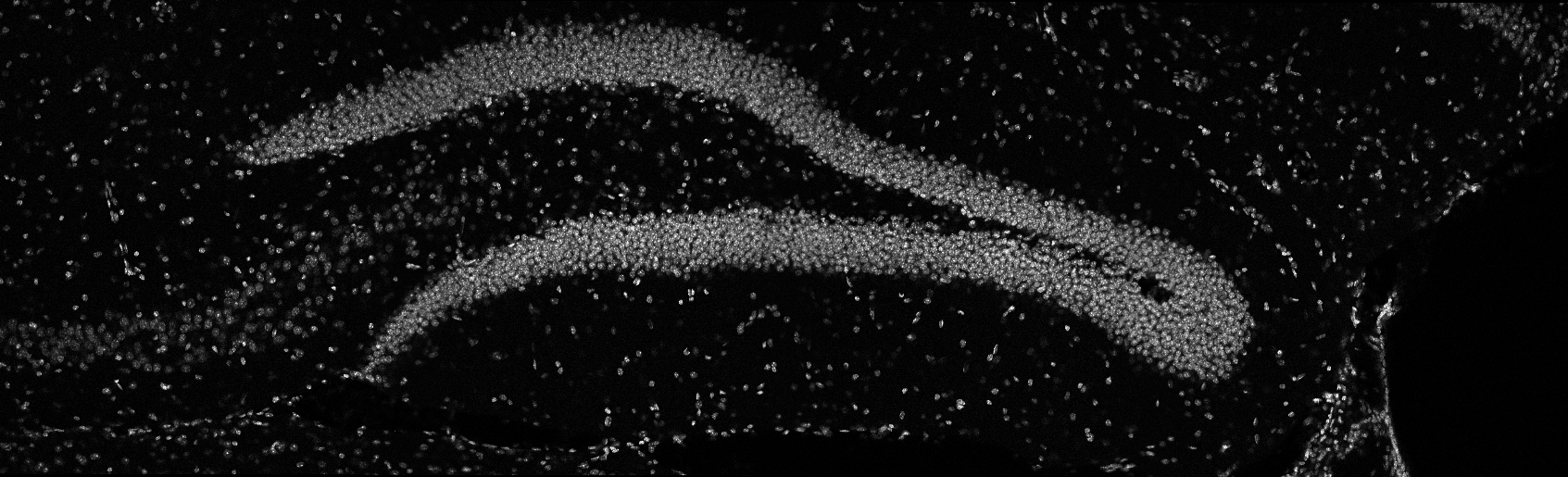

Supplement: Supplementary file 8 — Source data Fig. 6 [file 44319_2024_205_MOESM8_ESM.zip › Source_data_Figure6/6G/Derl1f:f + 4-PBA/Hoechst.tif]

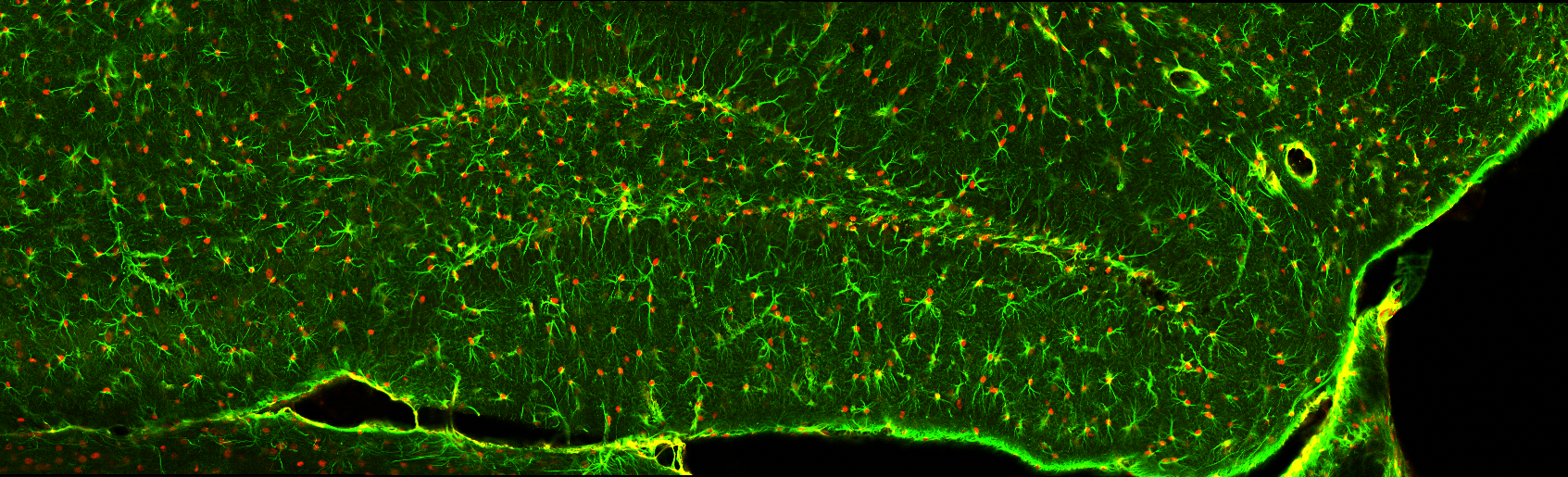

Supplement: Supplementary file 8 — Source data Fig. 6 [file 44319_2024_205_MOESM8_ESM.zip › Source_data_Figure6/6G/Derl1f:f + 4-PBA/Merge.tif]

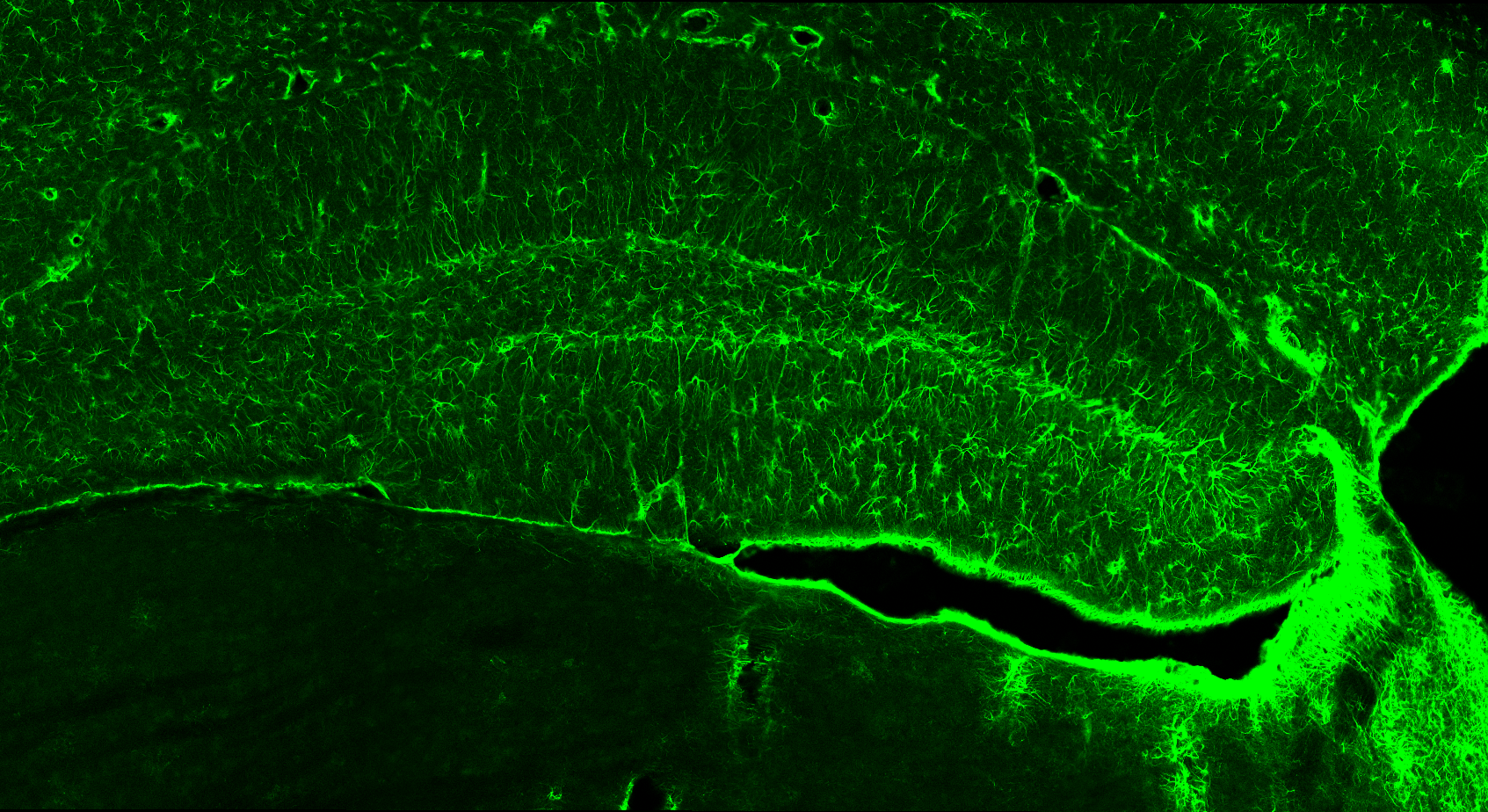

Supplement: Supplementary file 8 — Source data Fig. 6 [file 44319_2024_205_MOESM8_ESM.zip › Source_data_Figure6/6G/Derl1f:f + vehicle/GFAP.tif]

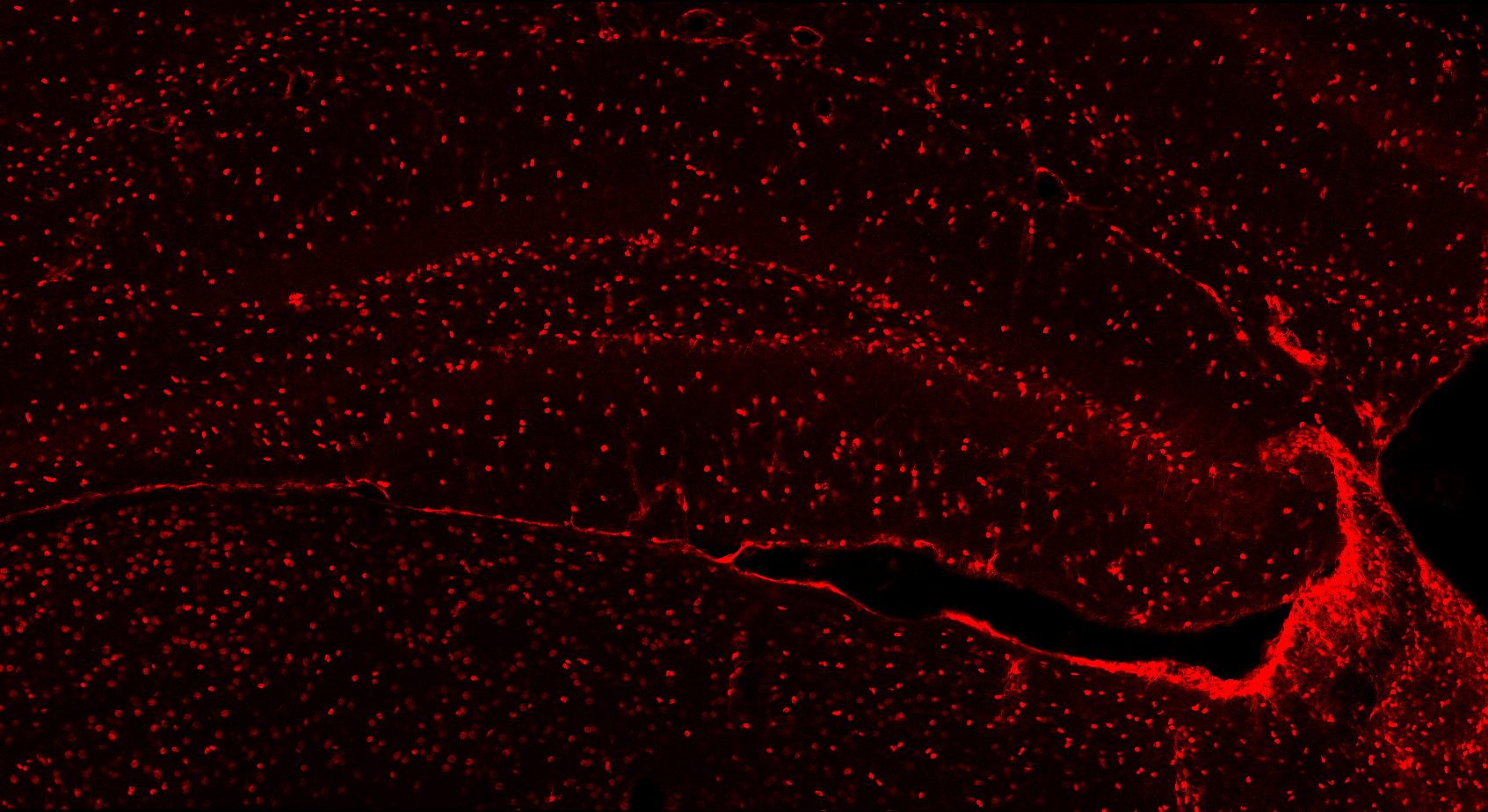

Supplement: Supplementary file 8 — Source data Fig. 6 [file 44319_2024_205_MOESM8_ESM.zip › Source_data_Figure6/6G/Derl1f:f + vehicle/Sox2.tif]

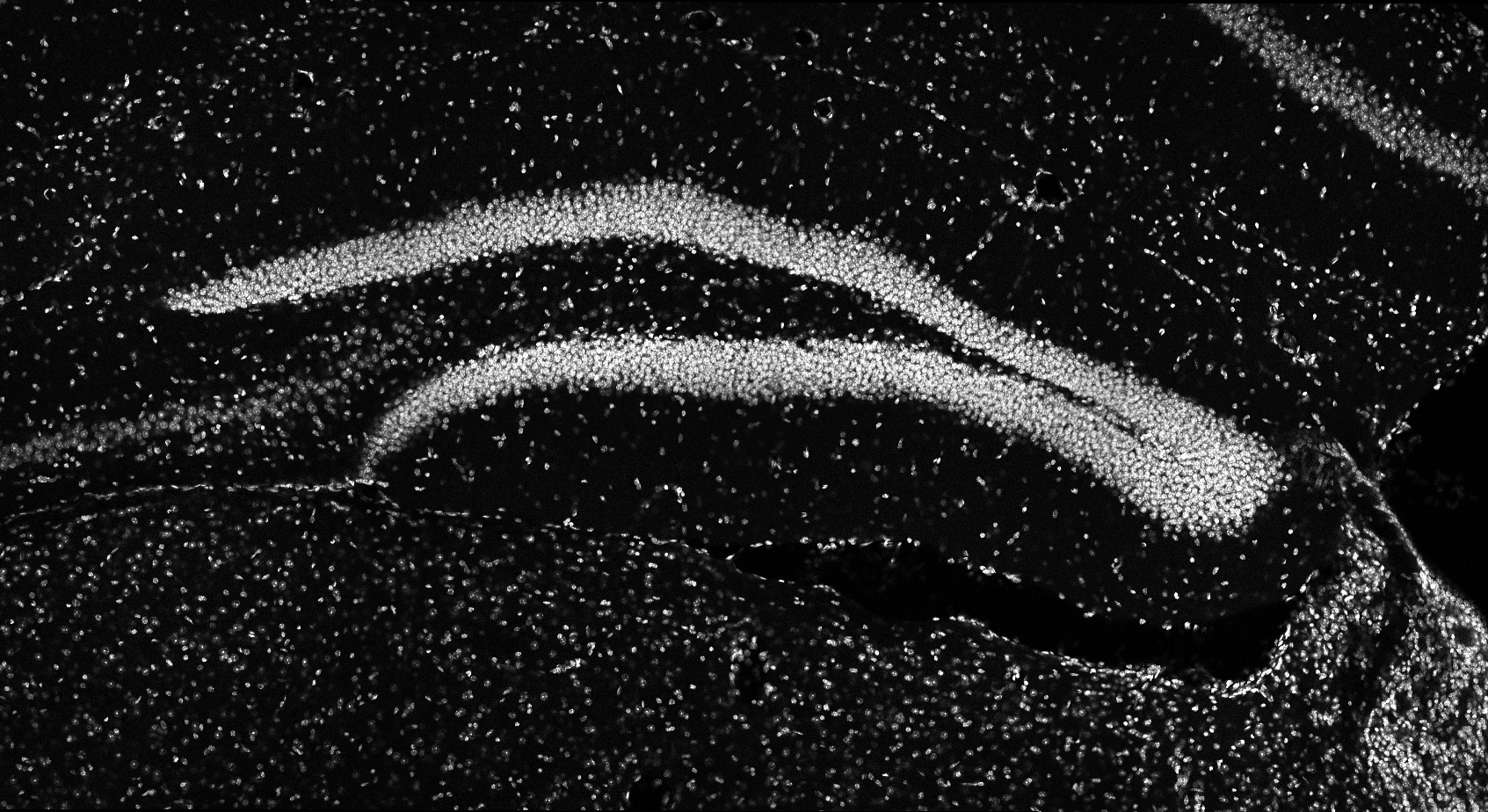

Supplement: Supplementary file 8 — Source data Fig. 6 [file 44319_2024_205_MOESM8_ESM.zip › Source_data_Figure6/6G/Derl1f:f + vehicle/Hoechst.tif]

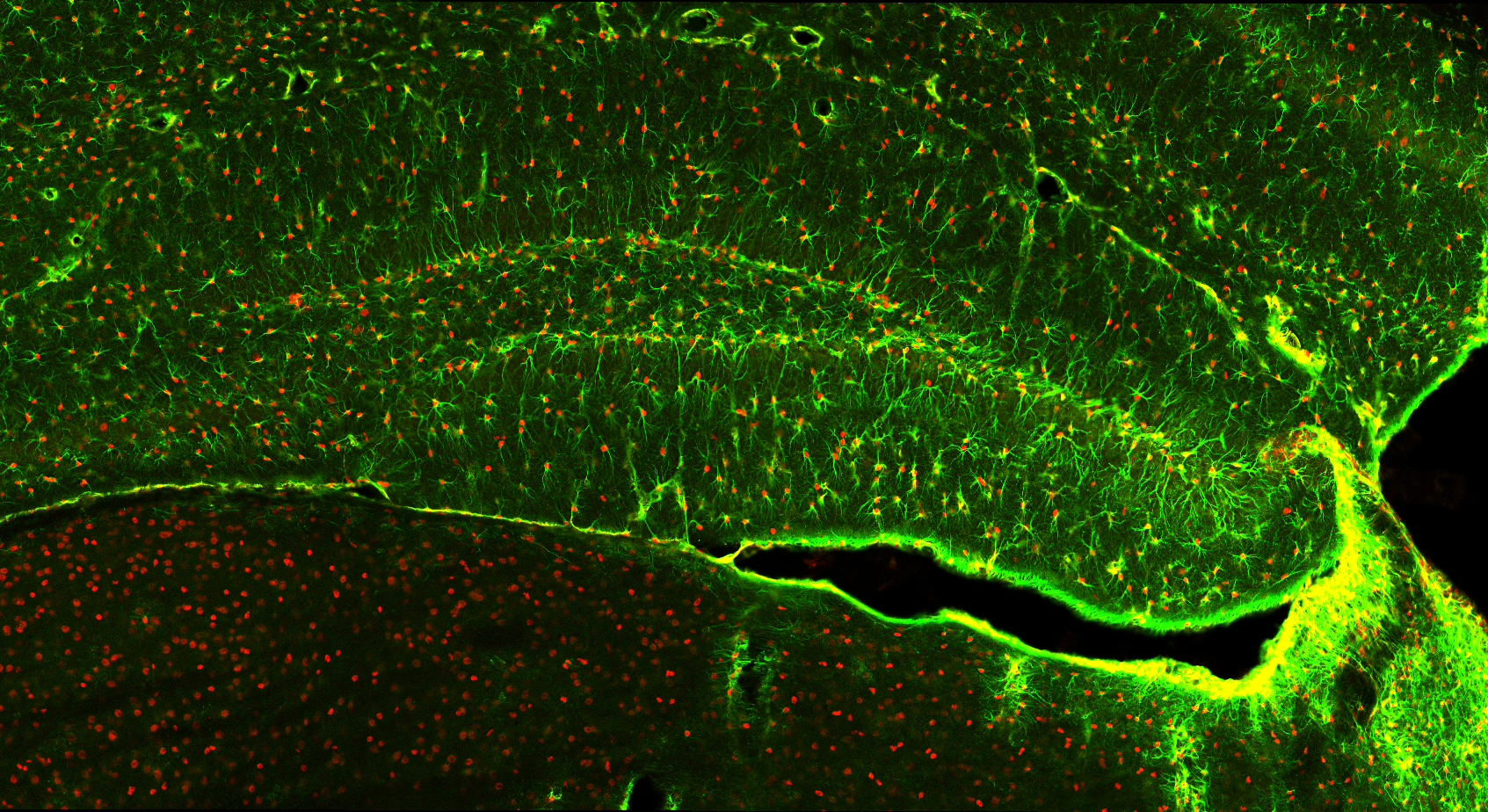

Supplement: Supplementary file 8 — Source data Fig. 6 [file 44319_2024_205_MOESM8_ESM.zip › Source_data_Figure6/6G/Derl1f:f + vehicle/Merge.tif]

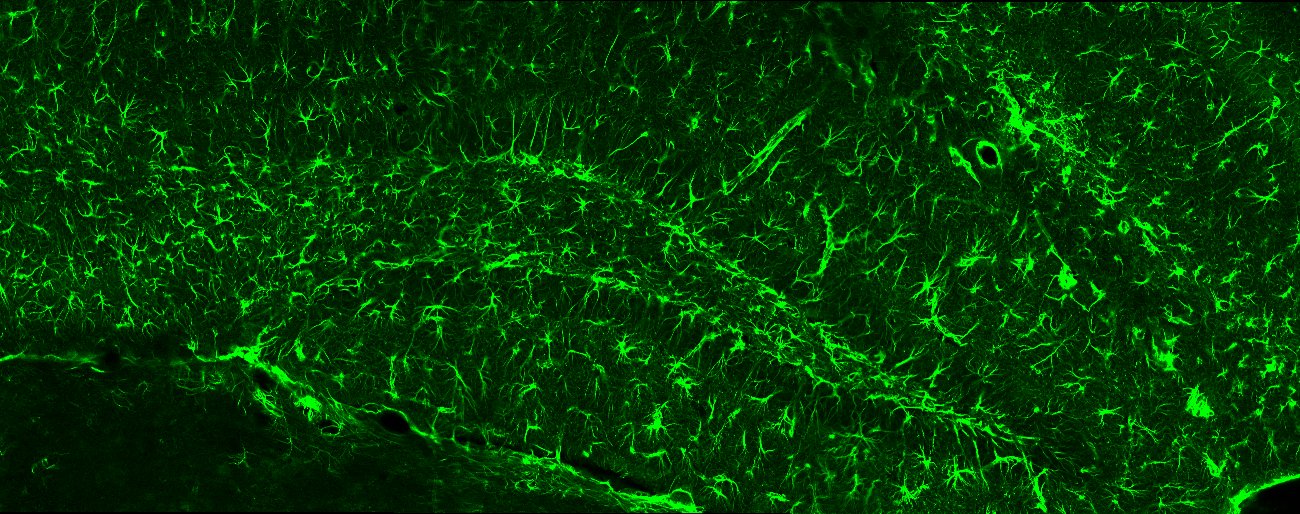

Supplement: Supplementary file 8 — Source data Fig. 6 [file 44319_2024_205_MOESM8_ESM.zip › Source_data_Figure6/6G/Derl1NesCre + 4-PBA/GFAP.tif]

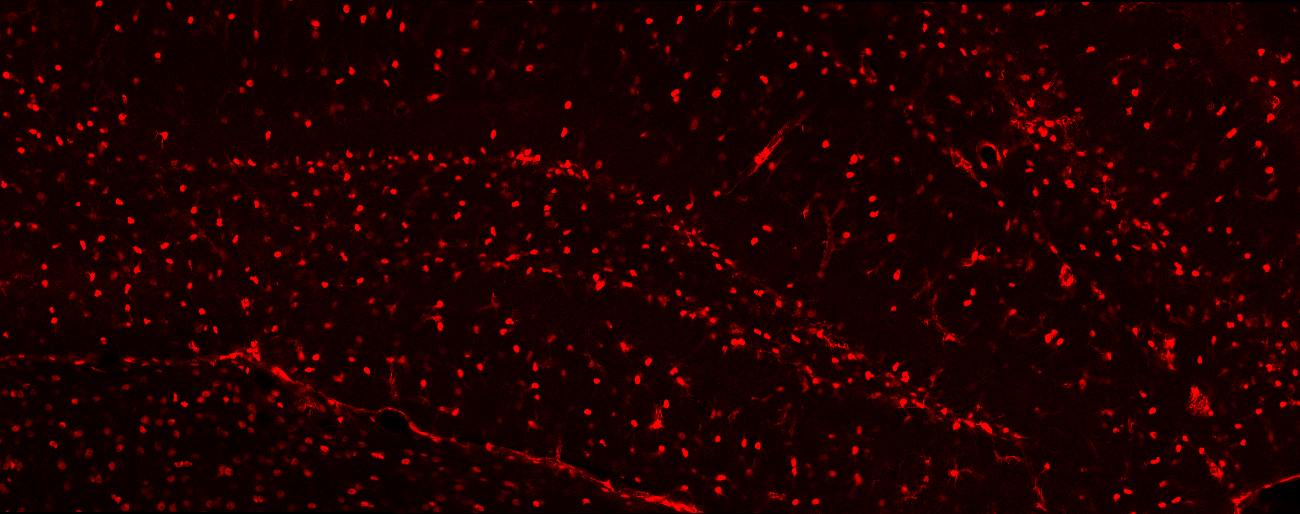

Supplement: Supplementary file 8 — Source data Fig. 6 [file 44319_2024_205_MOESM8_ESM.zip › Source_data_Figure6/6G/Derl1NesCre + 4-PBA/Sox2.tif]

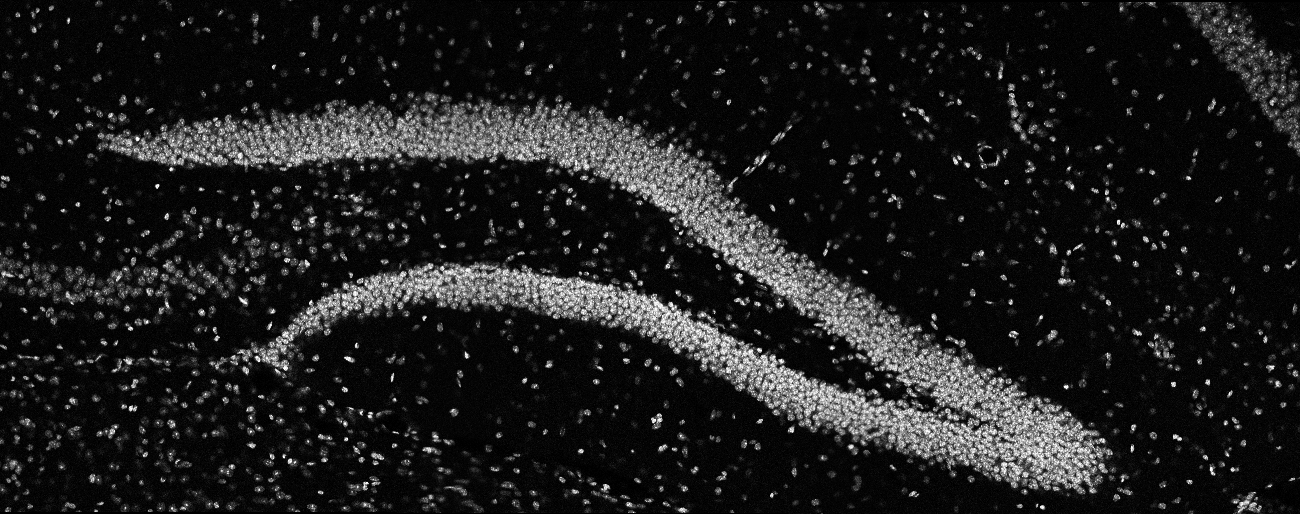

Supplement: Supplementary file 8 — Source data Fig. 6 [file 44319_2024_205_MOESM8_ESM.zip › Source_data_Figure6/6G/Derl1NesCre + 4-PBA/Hoechst.tif]

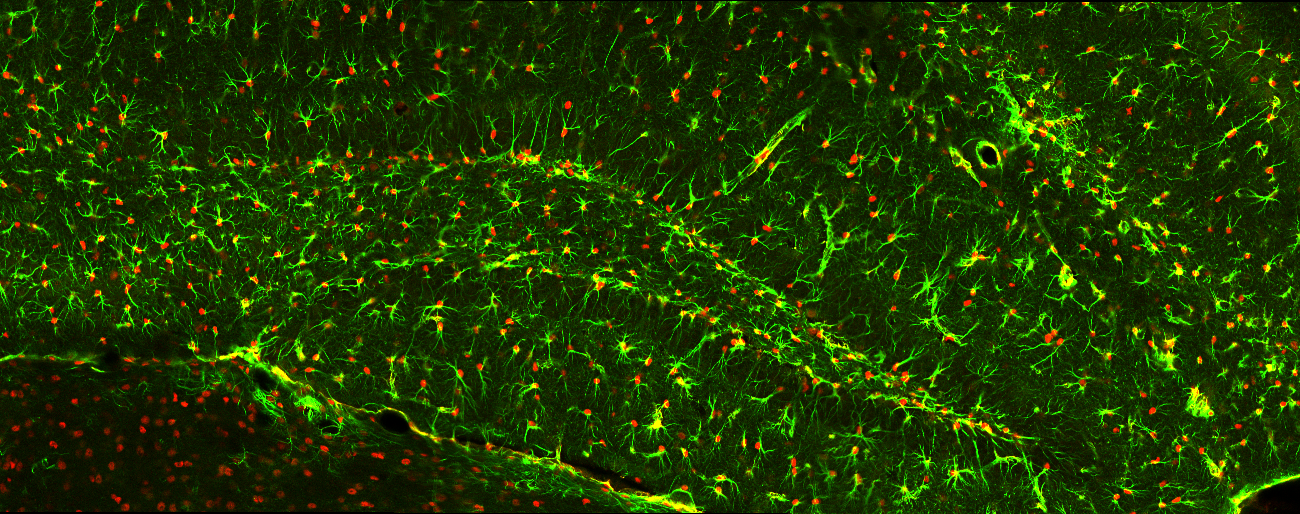

Supplement: Supplementary file 8 — Source data Fig. 6 [file 44319_2024_205_MOESM8_ESM.zip › Source_data_Figure6/6G/Derl1NesCre + 4-PBA/Merge.tif]

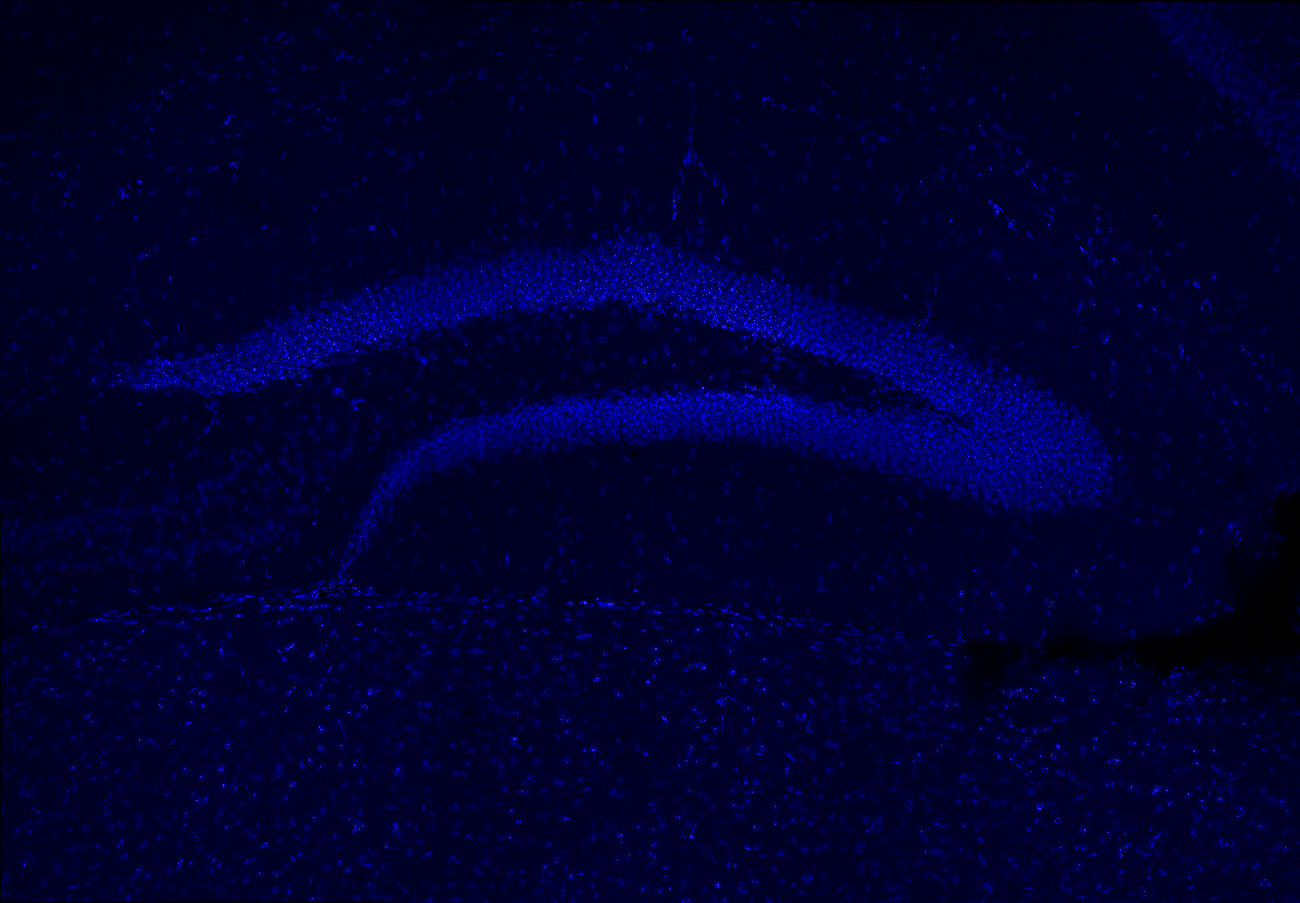

Supplement: Supplementary file 8 — Source data Fig. 6 [file 44319_2024_205_MOESM8_ESM.zip › Source_data_Figure6/6B/Derl1NesCre + vehicle/Hoechst.tif]

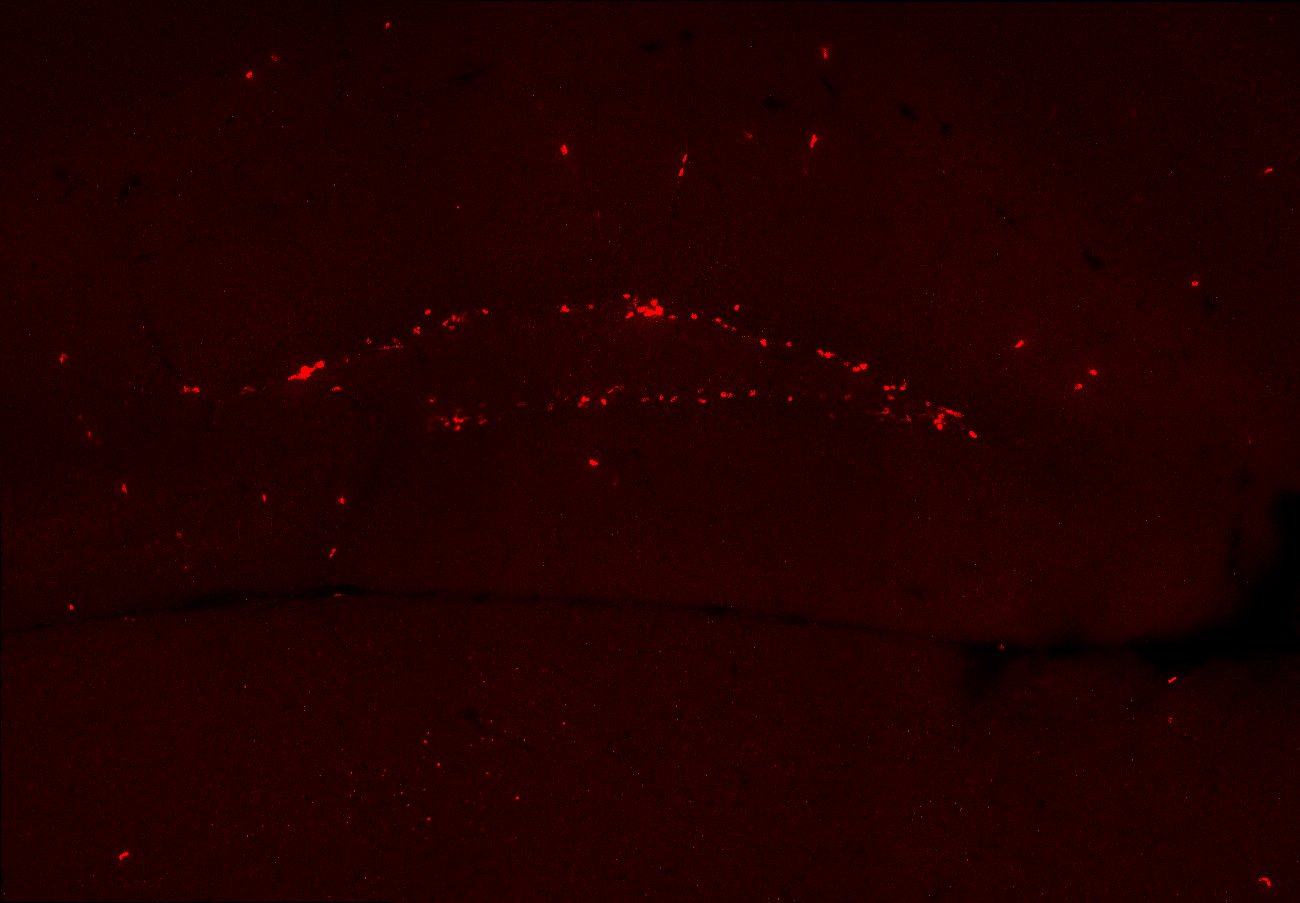

Supplement: Supplementary file 8 — Source data Fig. 6 [file 44319_2024_205_MOESM8_ESM.zip › Source_data_Figure6/6B/Derl1NesCre + vehicle/BrdU.tif]

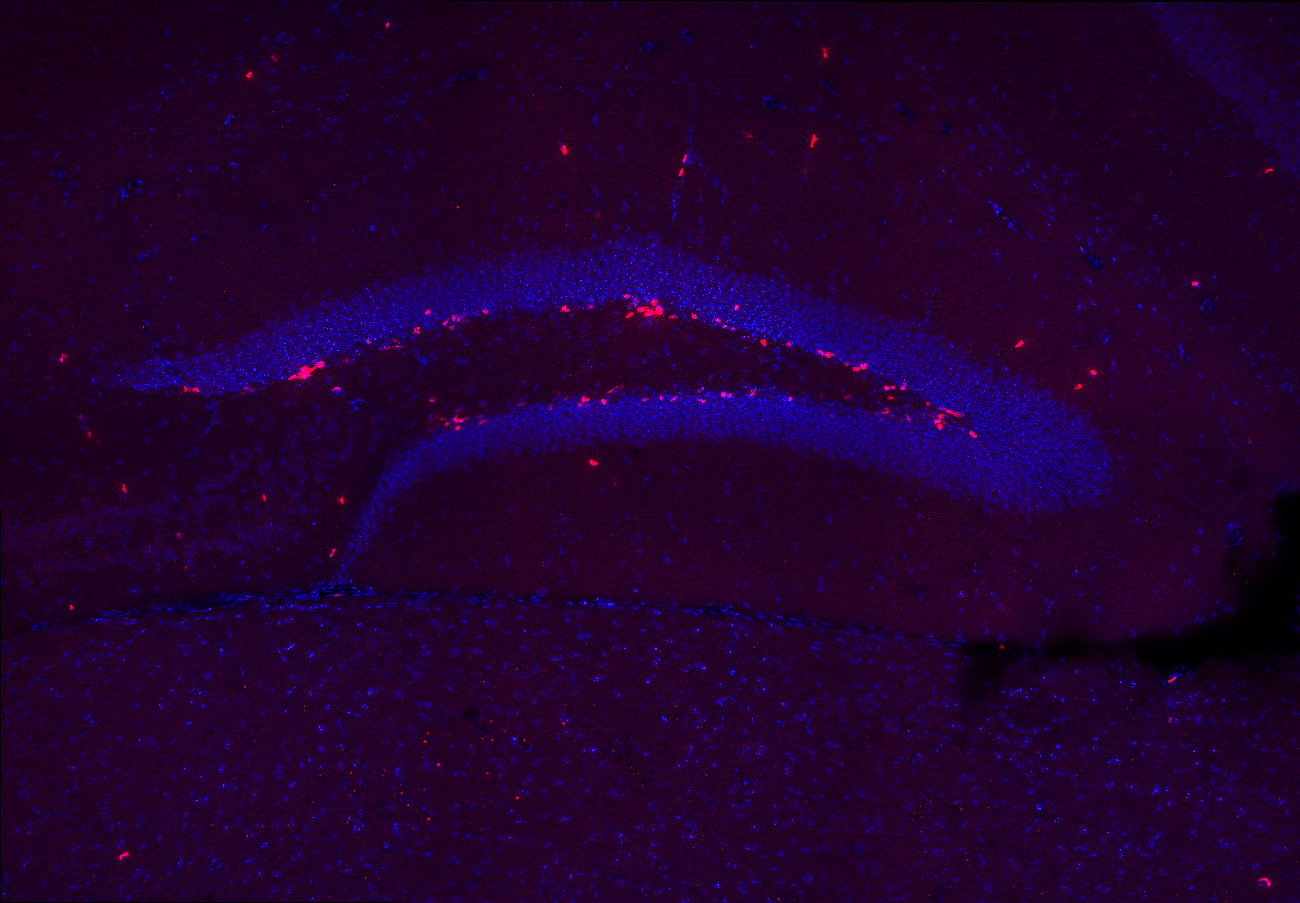

Supplement: Supplementary file 8 — Source data Fig. 6 [file 44319_2024_205_MOESM8_ESM.zip › Source_data_Figure6/6B/Derl1NesCre + vehicle/Merge.tif]

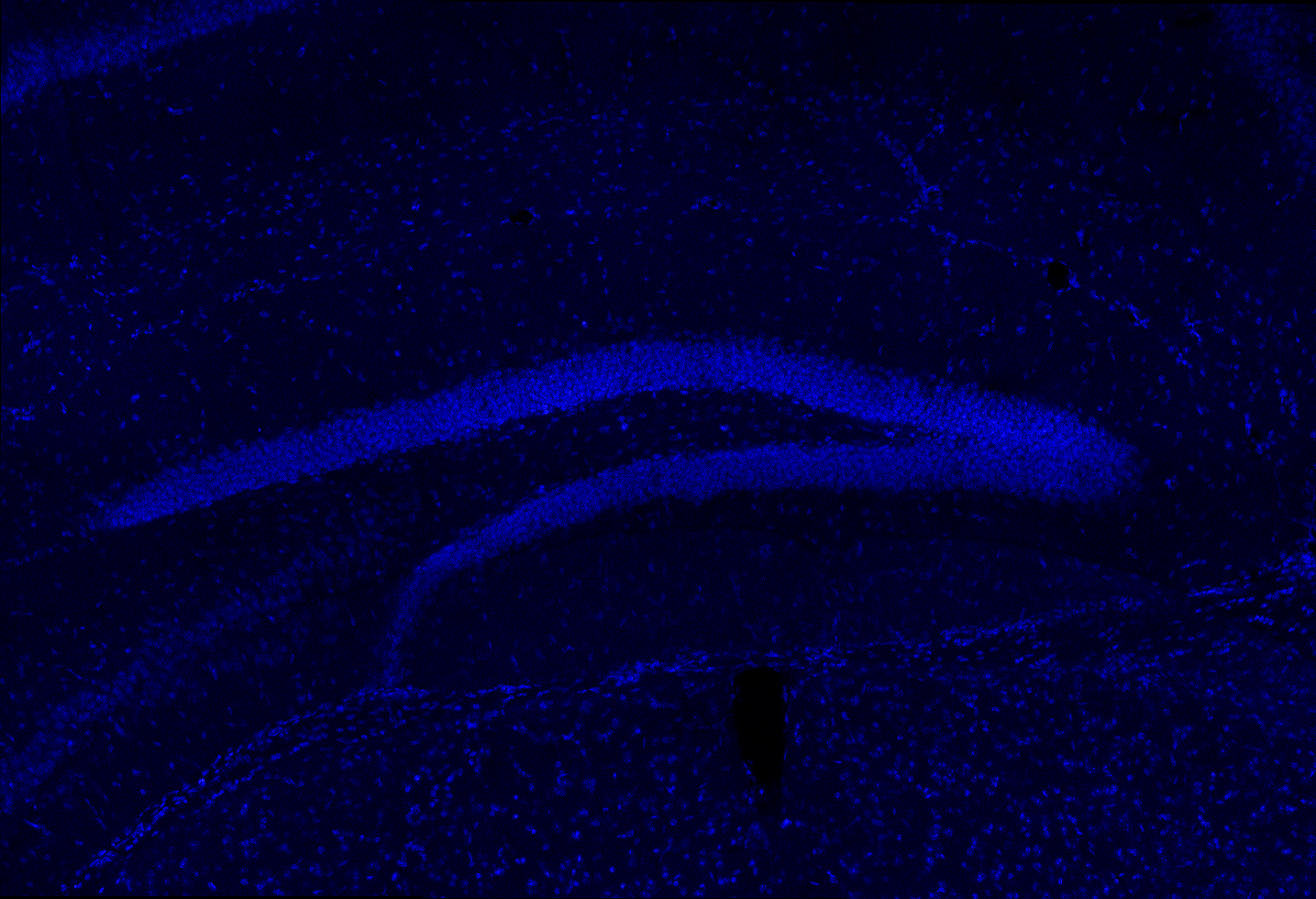

Supplement: Supplementary file 8 — Source data Fig. 6 [file 44319_2024_205_MOESM8_ESM.zip › Source_data_Figure6/6B/Derl1f:f + 4-PBA/Hoechst.tif]

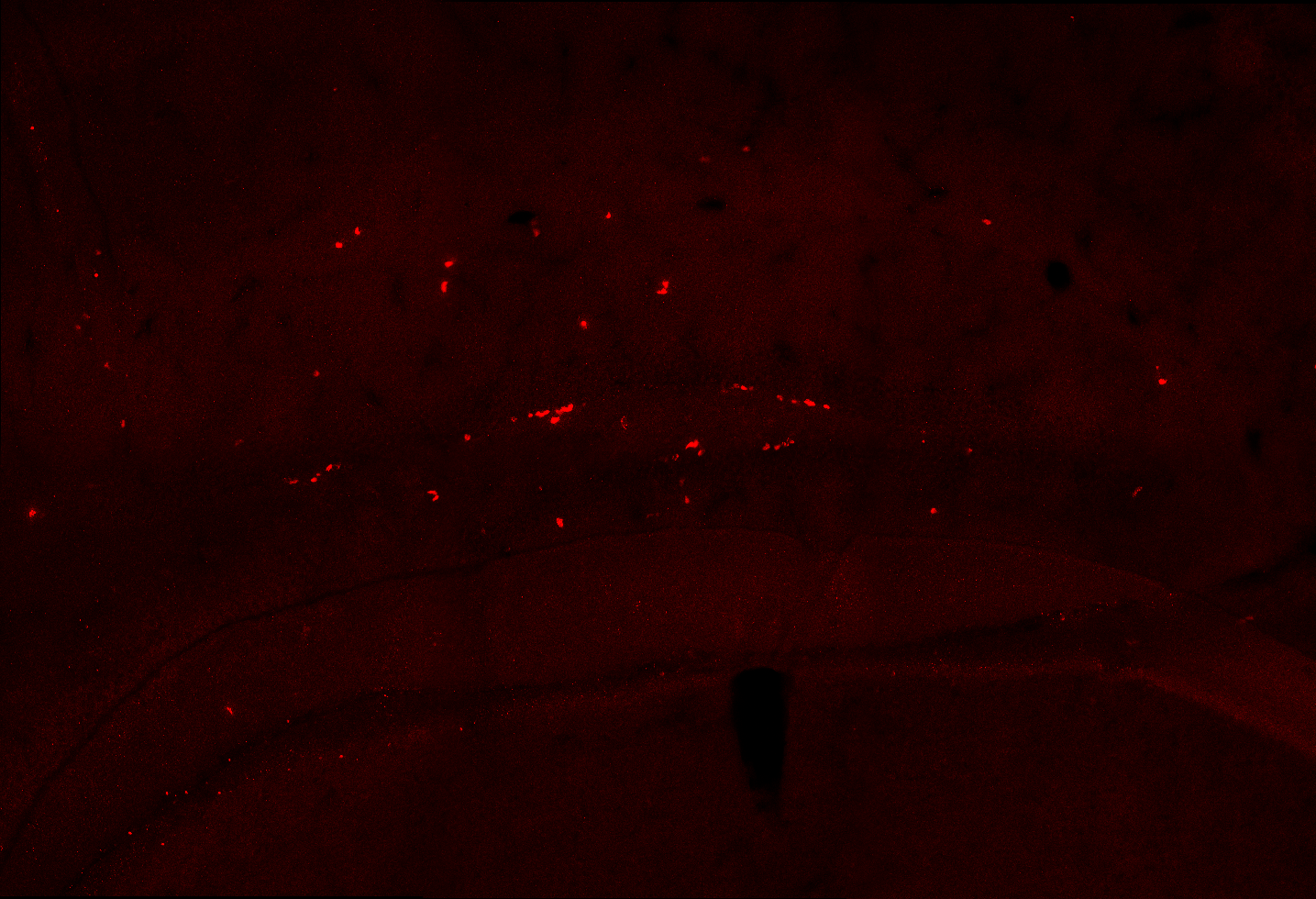

Supplement: Supplementary file 8 — Source data Fig. 6 [file 44319_2024_205_MOESM8_ESM.zip › Source_data_Figure6/6B/Derl1f:f + 4-PBA/BrdU.tif]

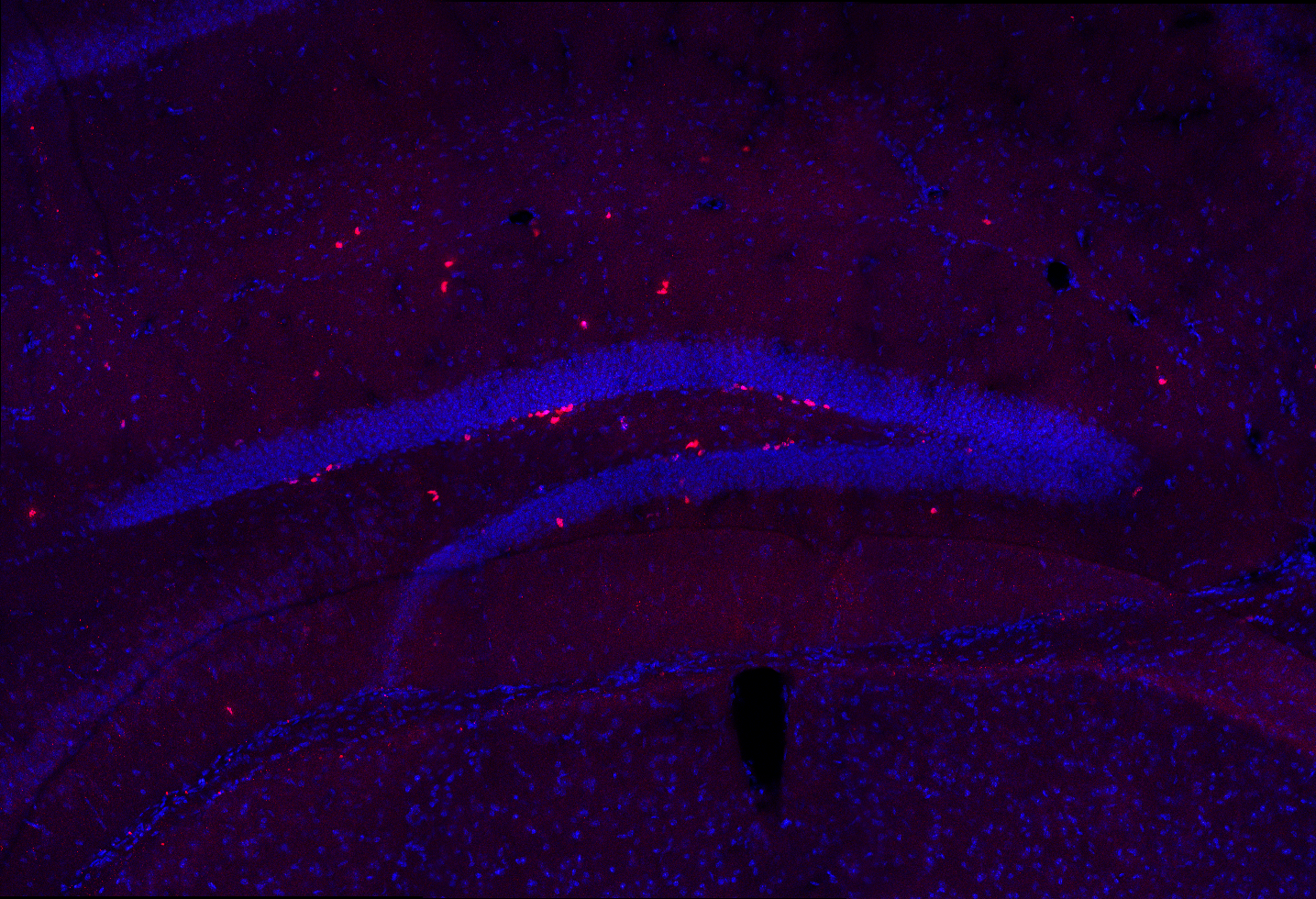

Supplement: Supplementary file 8 — Source data Fig. 6 [file 44319_2024_205_MOESM8_ESM.zip › Source_data_Figure6/6B/Derl1f:f + 4-PBA/Merge.tif]

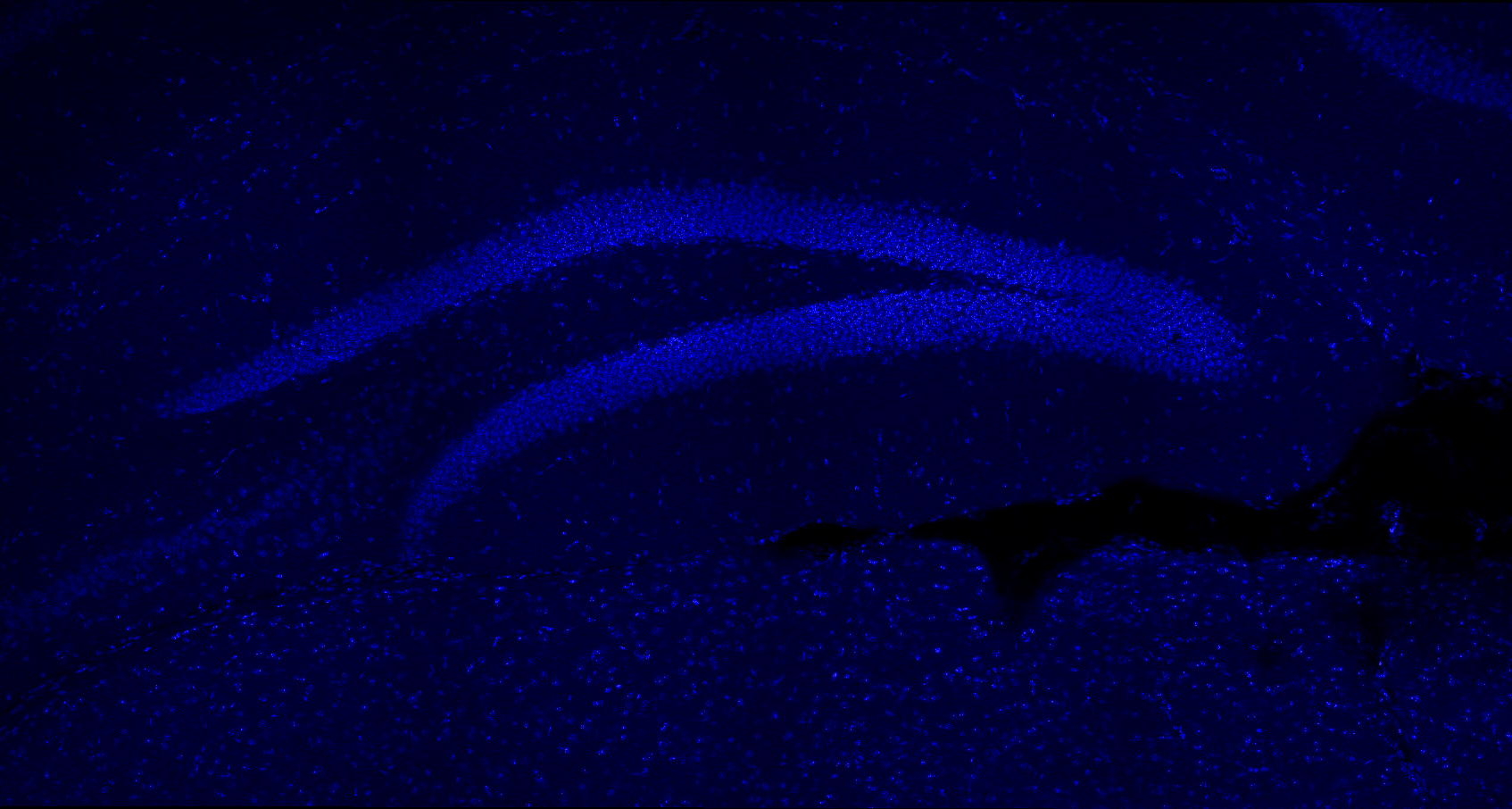

Supplement: Supplementary file 8 — Source data Fig. 6 [file 44319_2024_205_MOESM8_ESM.zip › Source_data_Figure6/6B/Derl1f:f + vehicle/Hoechst.tif]

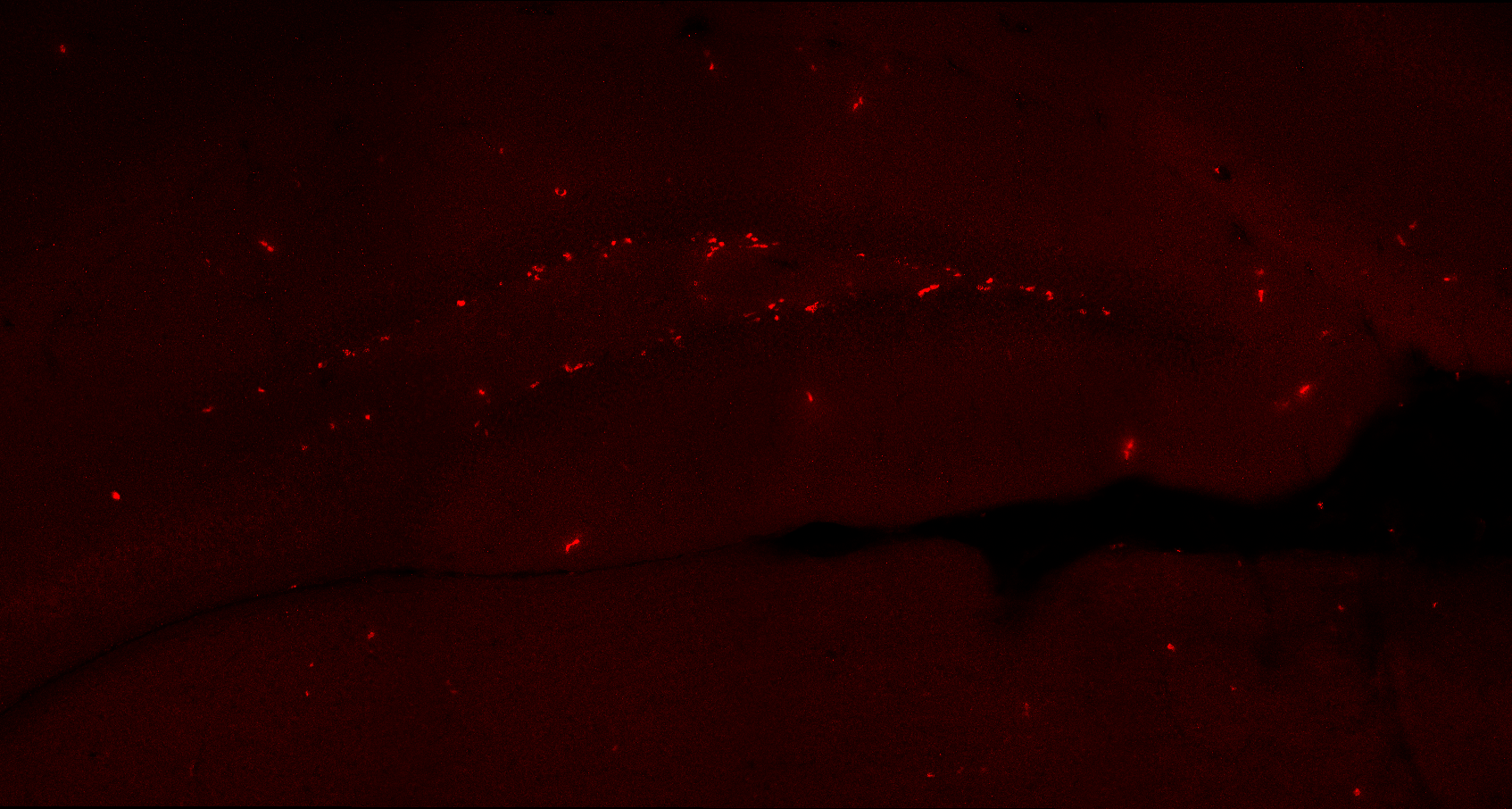

Supplement: Supplementary file 8 — Source data Fig. 6 [file 44319_2024_205_MOESM8_ESM.zip › Source_data_Figure6/6B/Derl1f:f + vehicle/BrdU.tif]

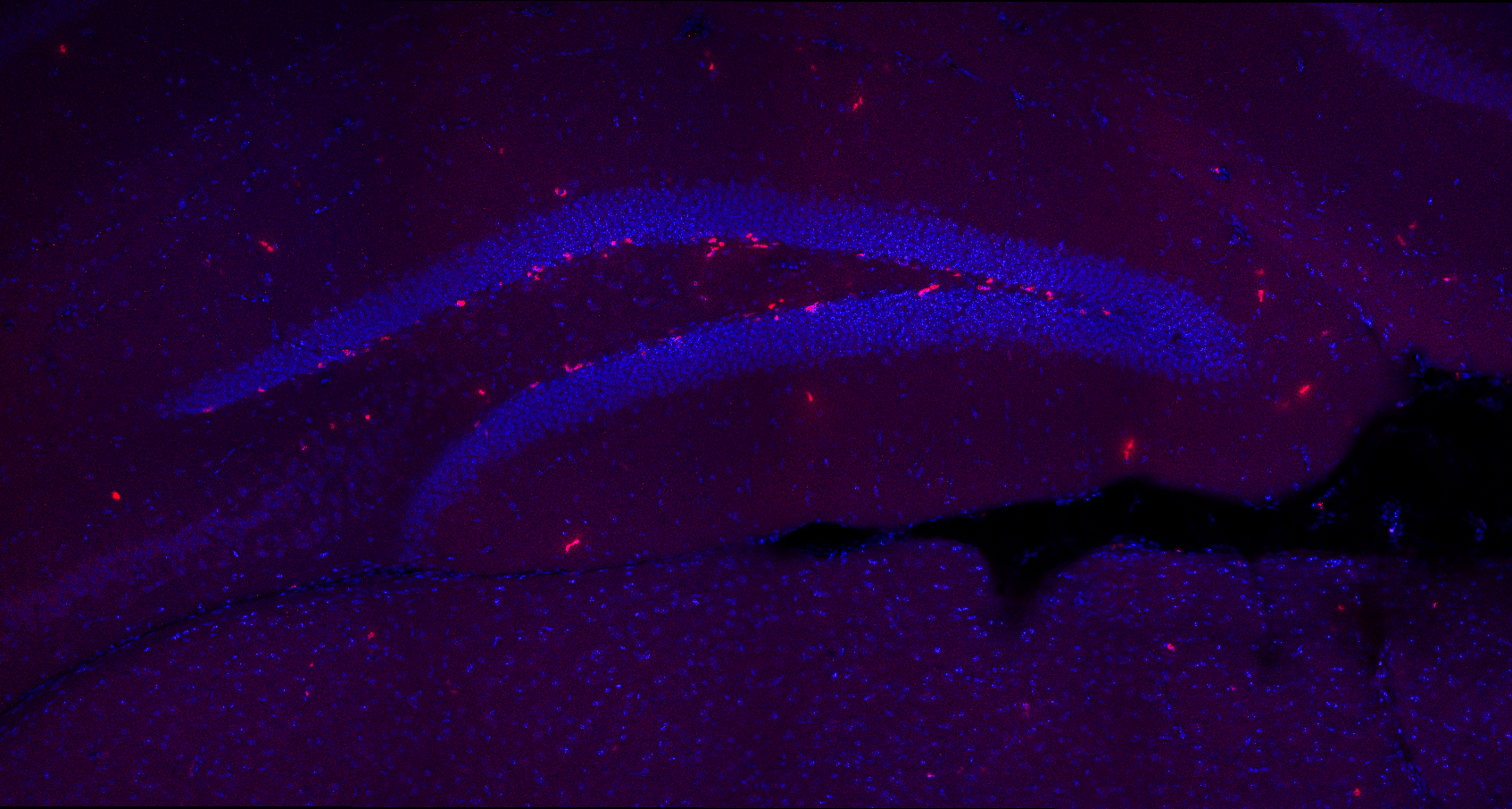

Supplement: Supplementary file 8 — Source data Fig. 6 [file 44319_2024_205_MOESM8_ESM.zip › Source_data_Figure6/6B/Derl1f:f + vehicle/Merge.tif]

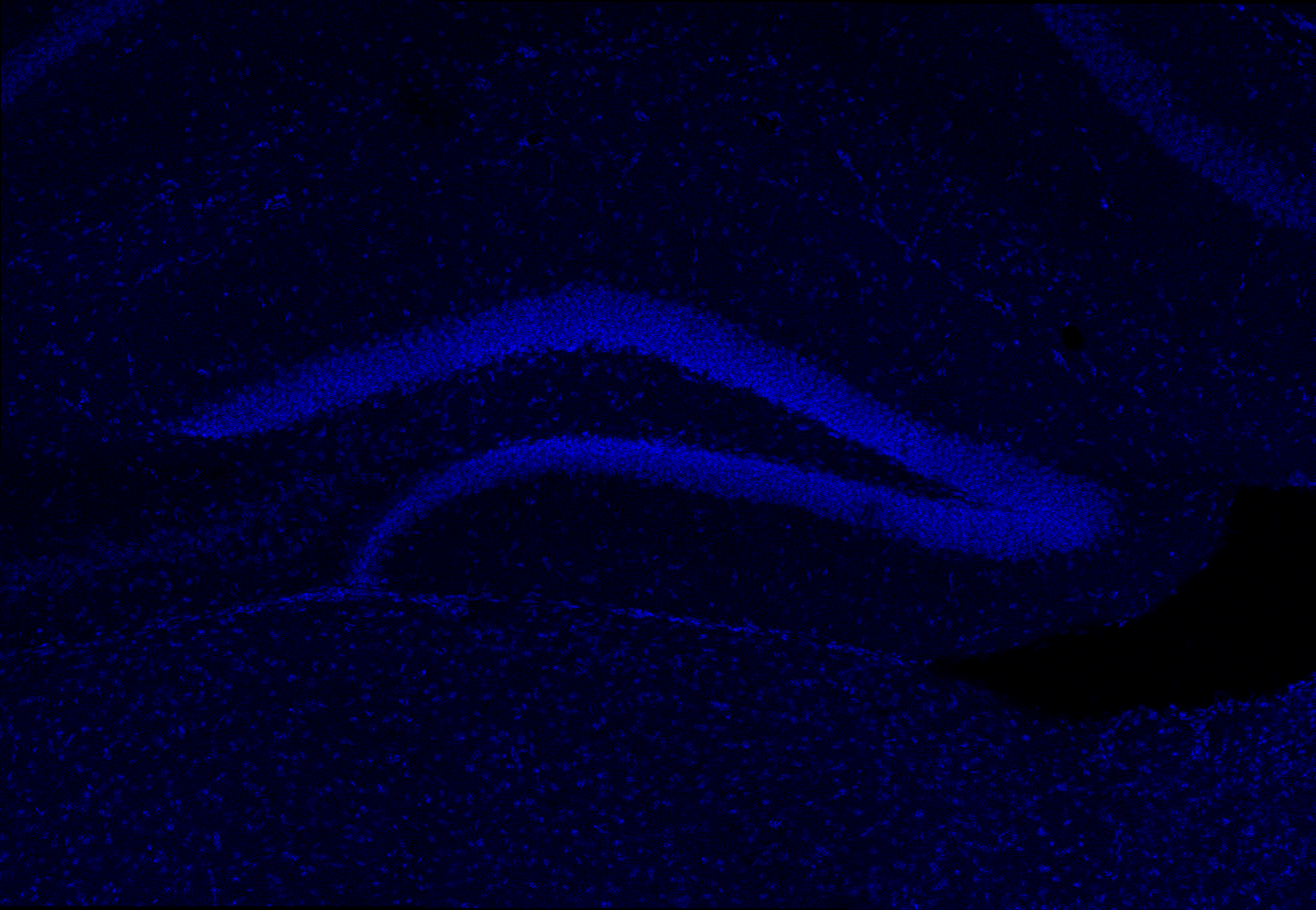

Supplement: Supplementary file 8 — Source data Fig. 6 [file 44319_2024_205_MOESM8_ESM.zip › Source_data_Figure6/6B/Derl1NesCre + 4-PBA/Hoechst.tif]

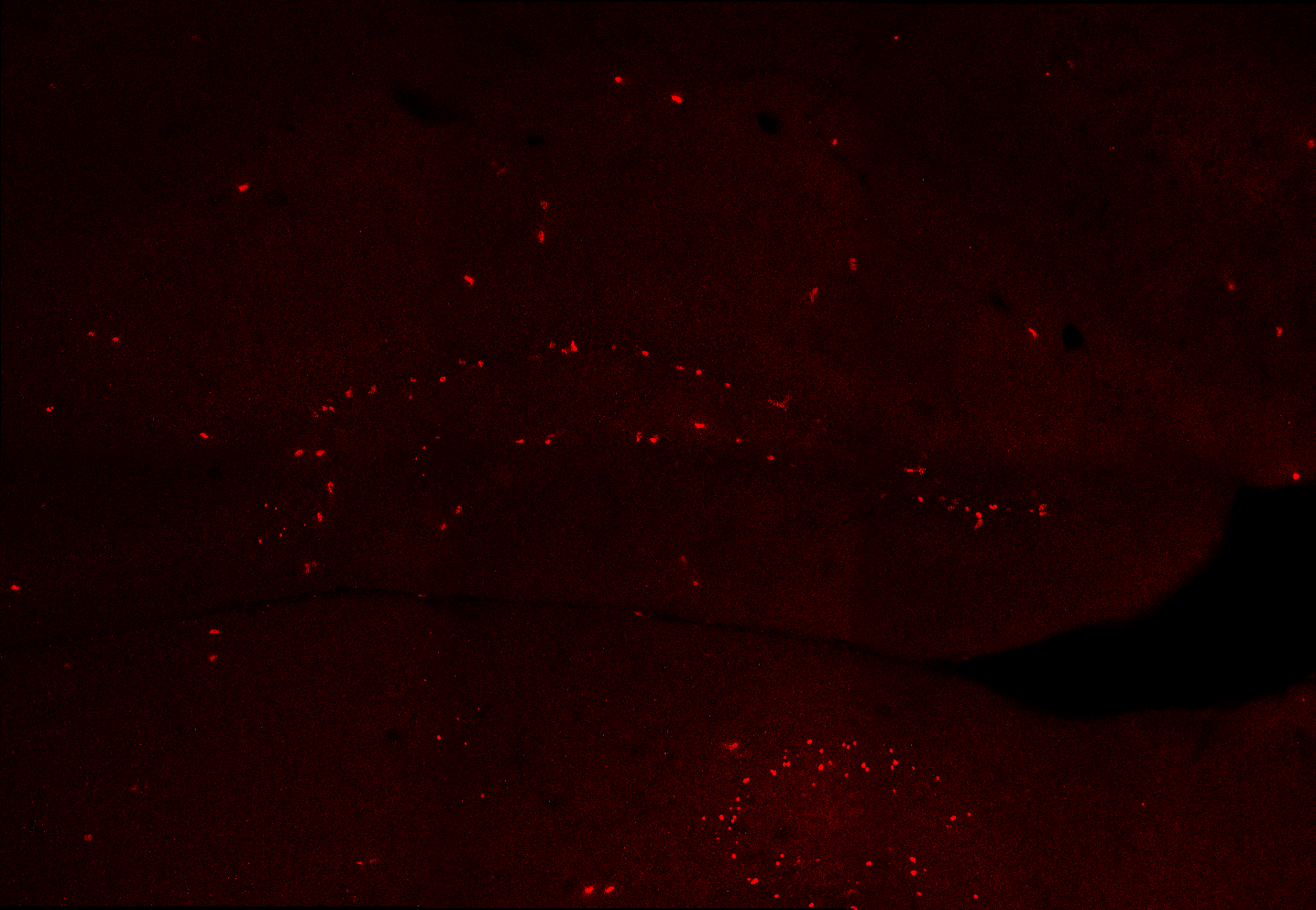

Supplement: Supplementary file 8 — Source data Fig. 6 [file 44319_2024_205_MOESM8_ESM.zip › Source_data_Figure6/6B/Derl1NesCre + 4-PBA/BrdU.tif]

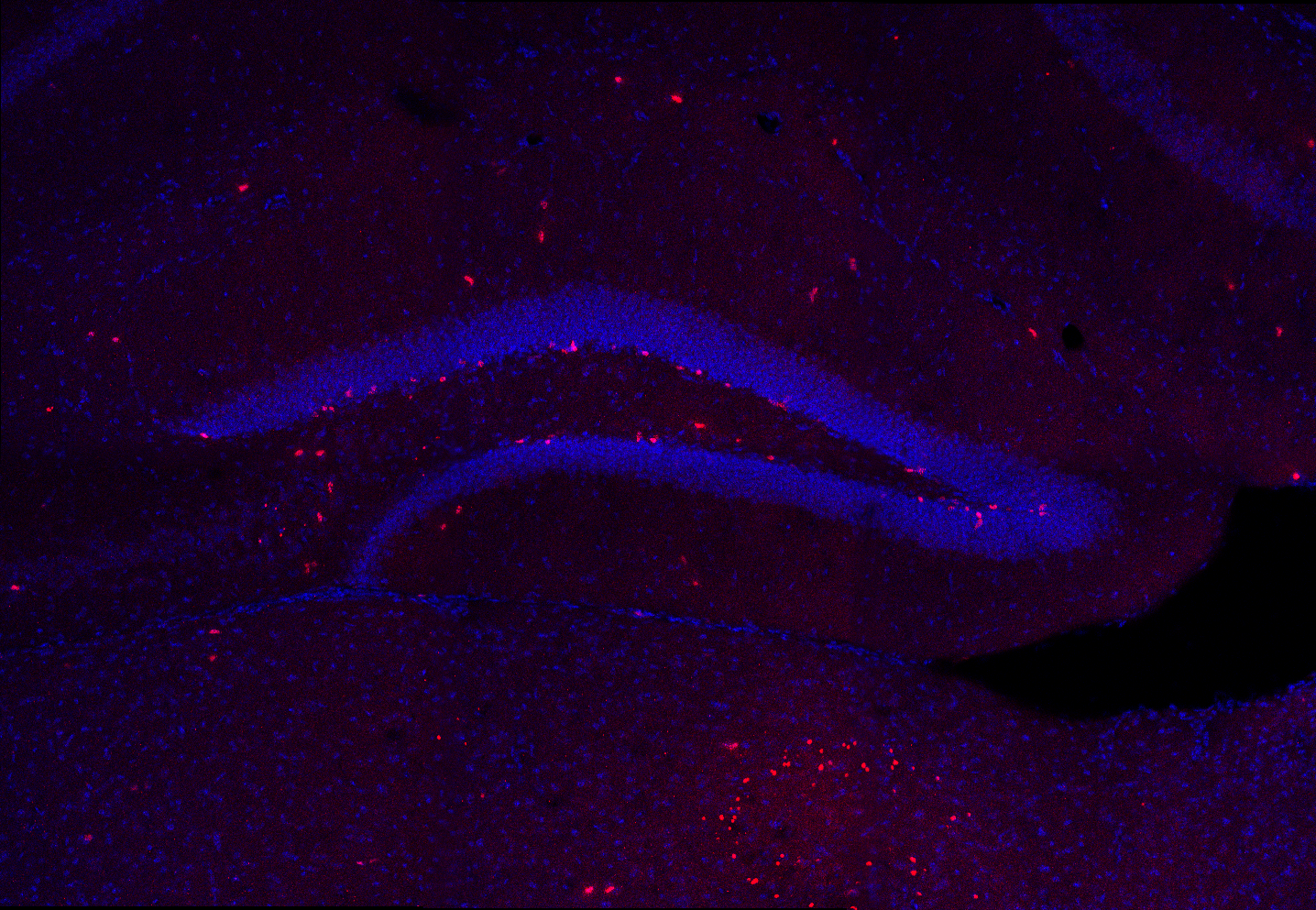

Supplement: Supplementary file 8 — Source data Fig. 6 [file 44319_2024_205_MOESM8_ESM.zip › Source_data_Figure6/6B/Derl1NesCre + 4-PBA/Merge.tif]

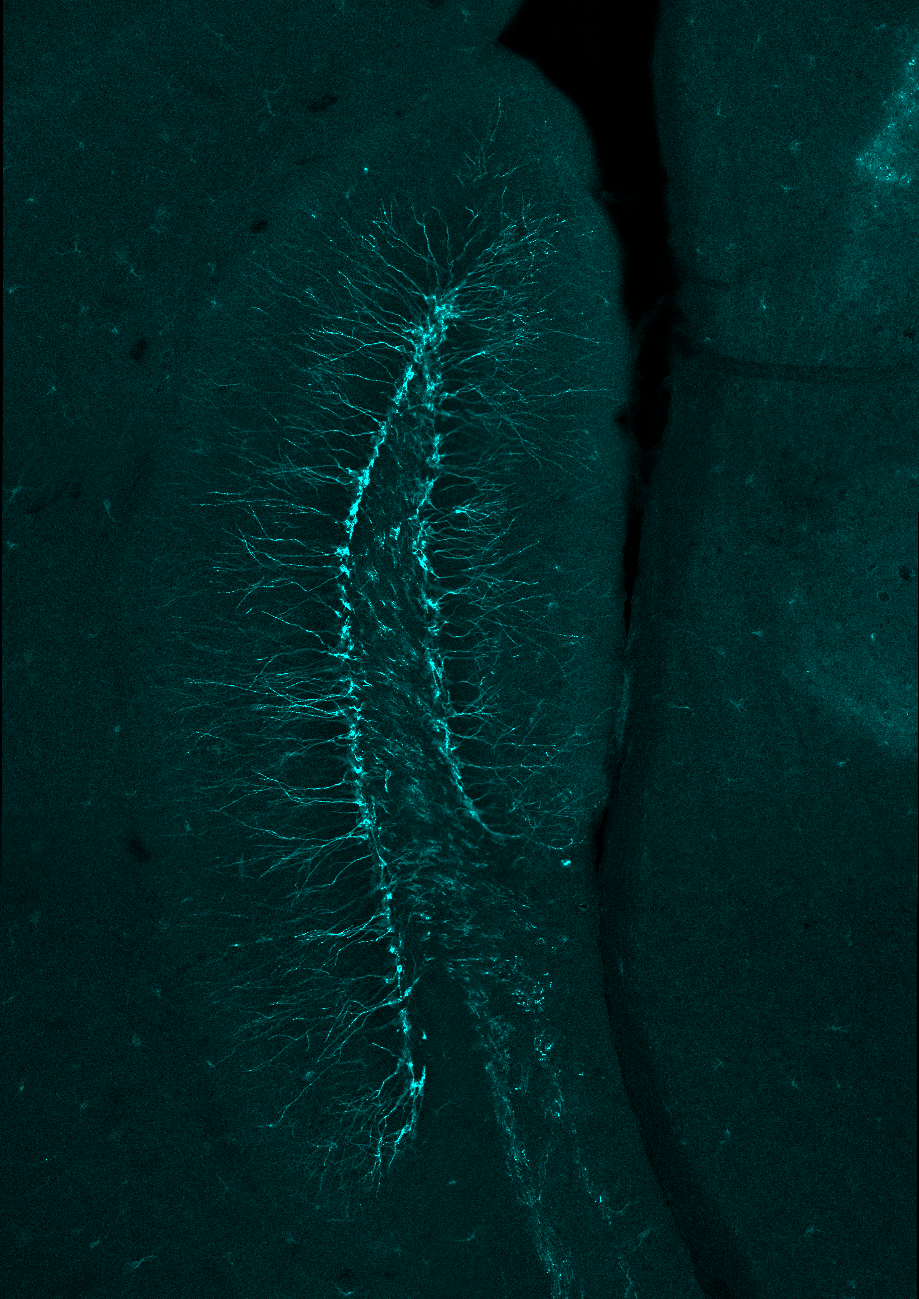

Supplement: Supplementary file 8 — Source data Fig. 6 [file 44319_2024_205_MOESM8_ESM.zip › Source_data_Figure6/6C/Derl1NesCre + vehicle/DCX.tif]

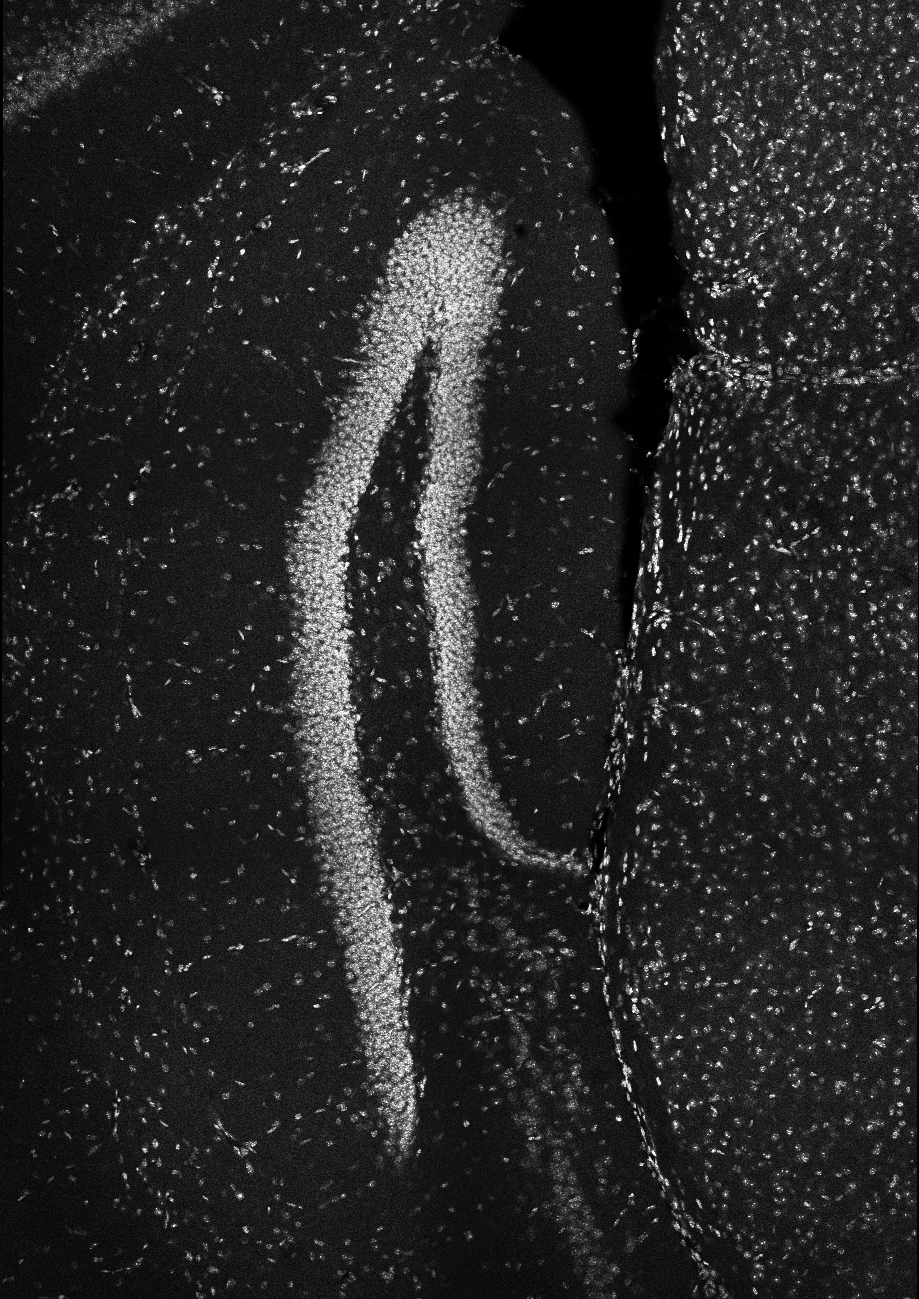

Supplement: Supplementary file 8 — Source data Fig. 6 [file 44319_2024_205_MOESM8_ESM.zip › Source_data_Figure6/6C/Derl1NesCre + vehicle/Hoechst.tif]

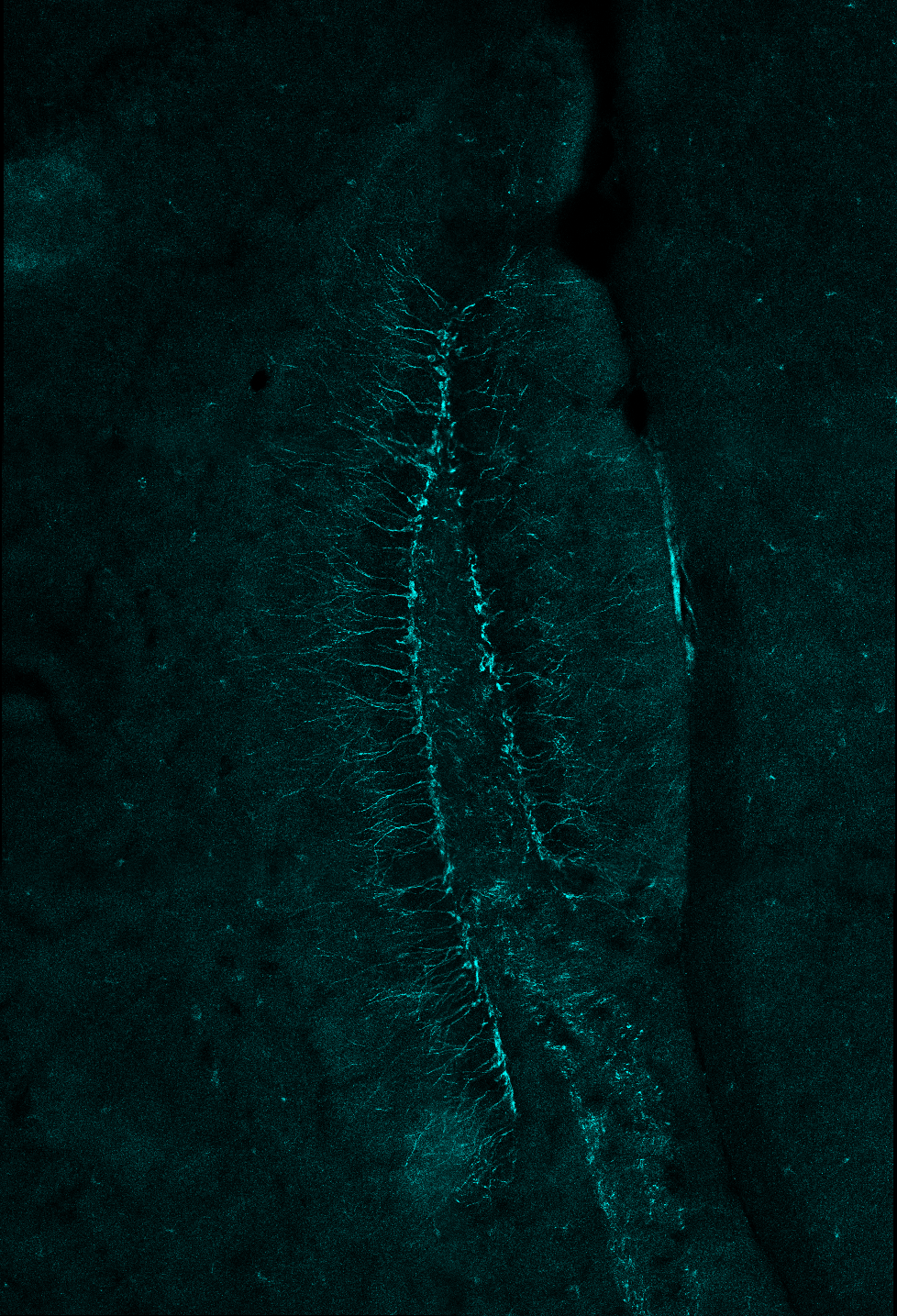

Supplement: Supplementary file 8 — Source data Fig. 6 [file 44319_2024_205_MOESM8_ESM.zip › Source_data_Figure6/6C/Derl1f:f + 4-PBA/DCX.tif]

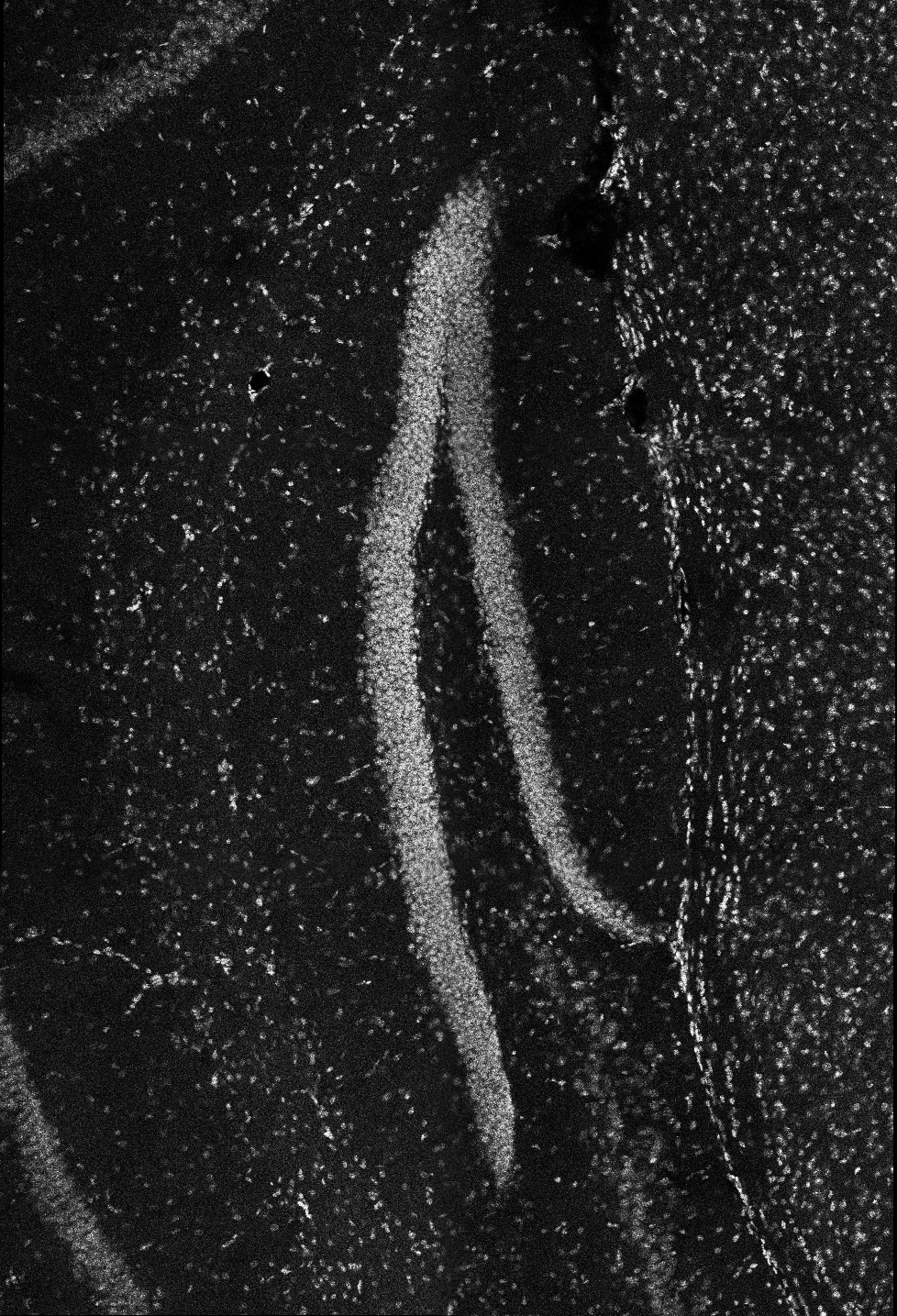

Supplement: Supplementary file 8 — Source data Fig. 6 [file 44319_2024_205_MOESM8_ESM.zip › Source_data_Figure6/6C/Derl1f:f + 4-PBA/Hoechst.tif]

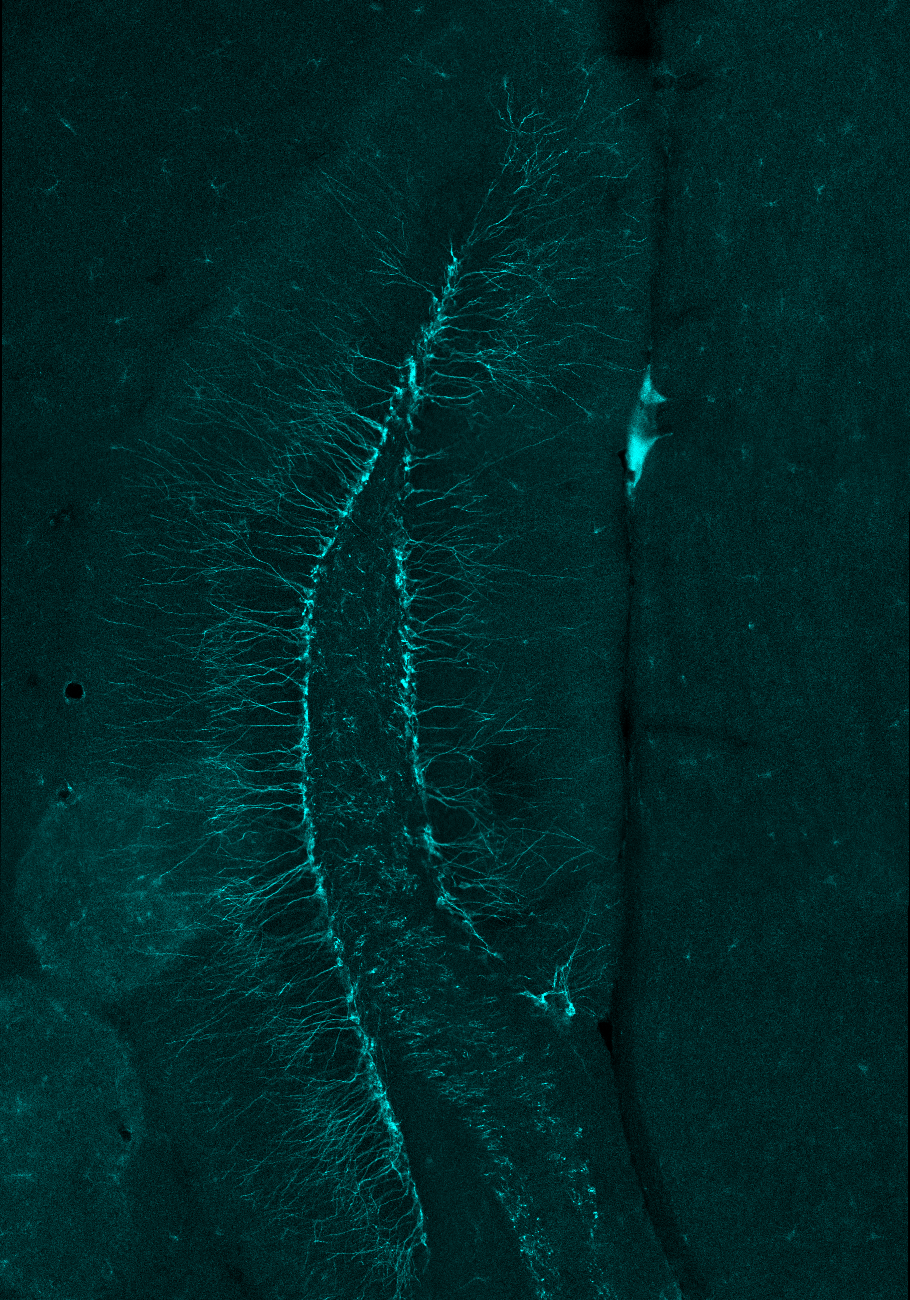

Supplement: Supplementary file 8 — Source data Fig. 6 [file 44319_2024_205_MOESM8_ESM.zip › Source_data_Figure6/6C/Derl1f:f + vehicle/DCX.tif]

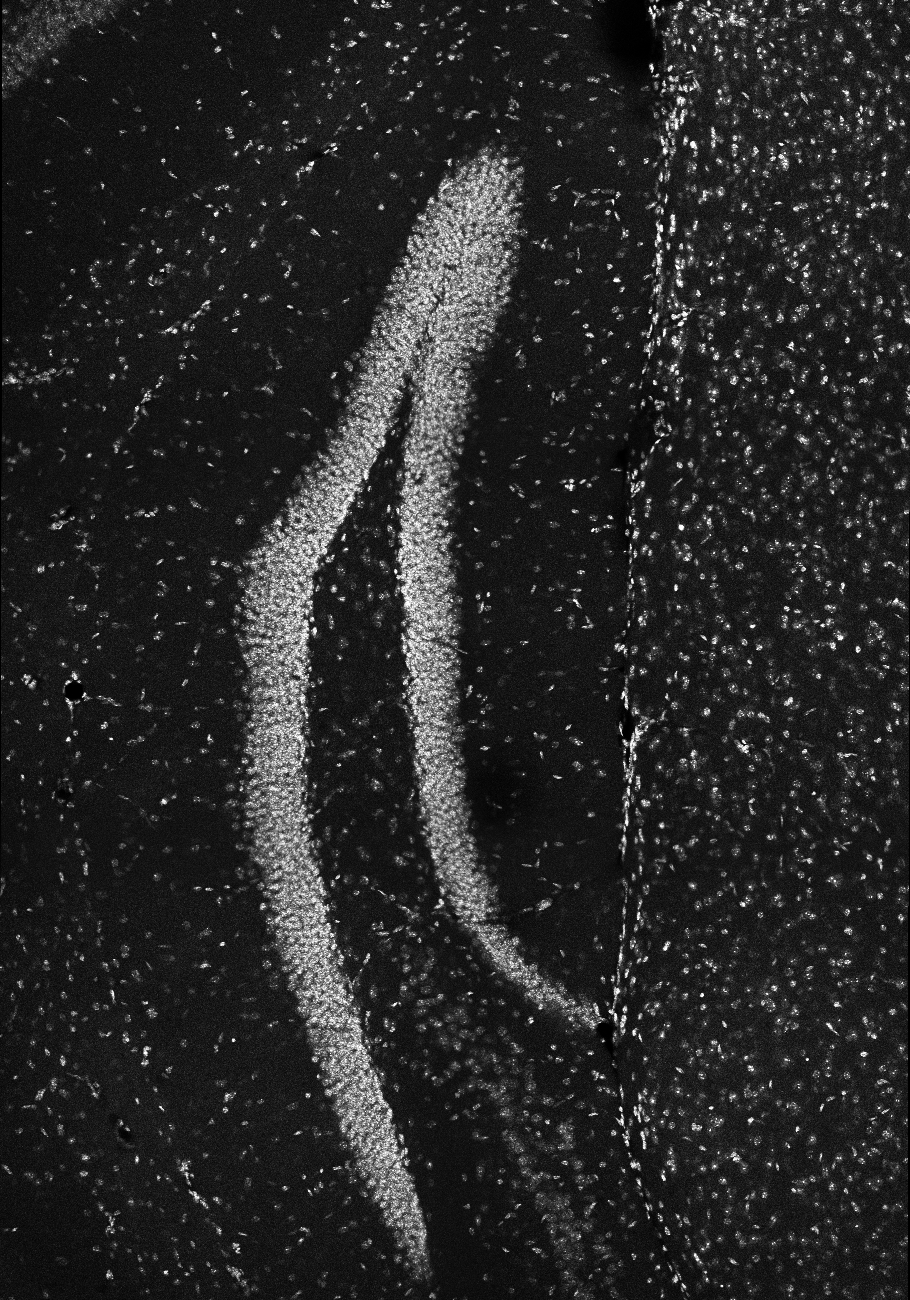

Supplement: Supplementary file 8 — Source data Fig. 6 [file 44319_2024_205_MOESM8_ESM.zip › Source_data_Figure6/6C/Derl1f:f + vehicle/Hoechst.tif]

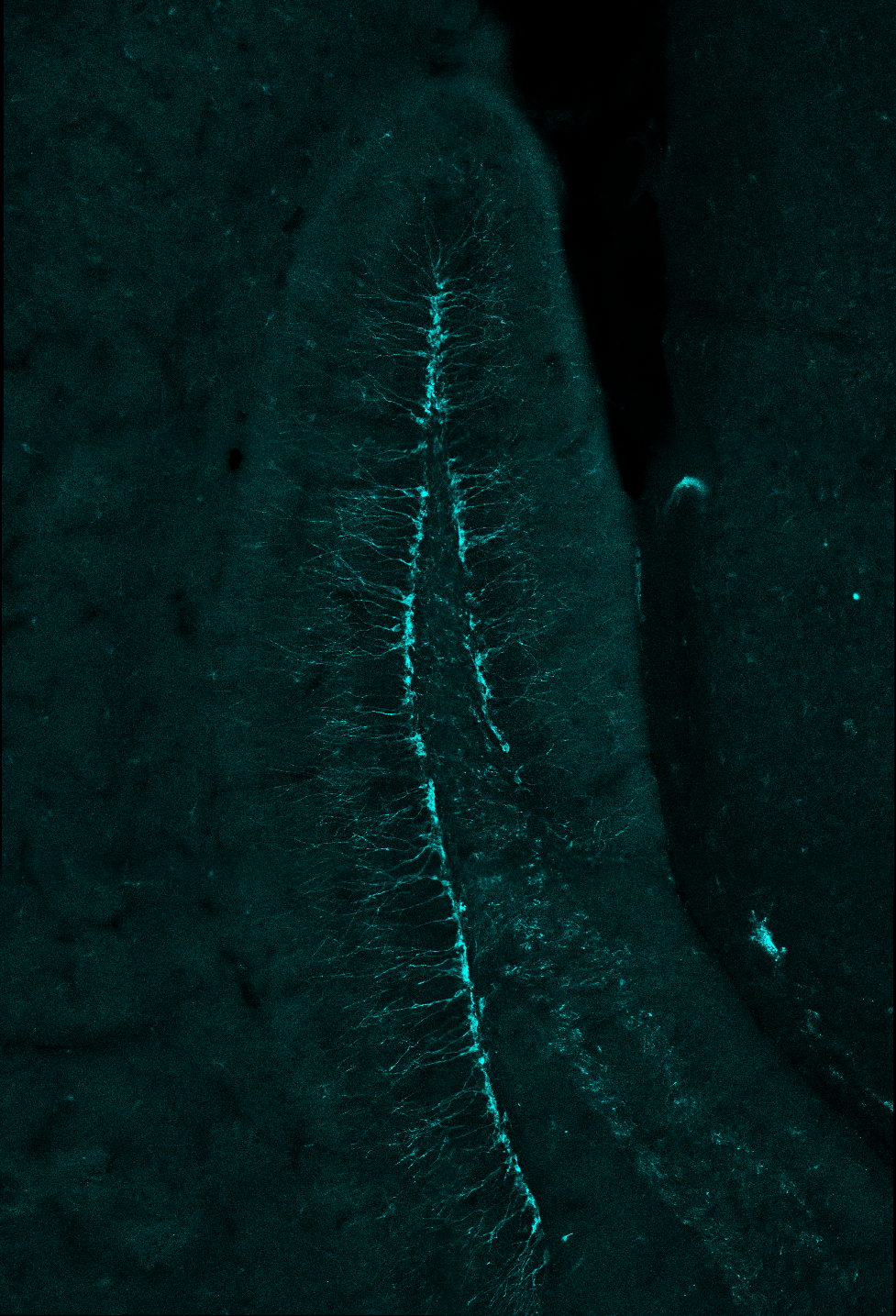

Supplement: Supplementary file 8 — Source data Fig. 6 [file 44319_2024_205_MOESM8_ESM.zip › Source_data_Figure6/6C/Derl1NesCre + 4-PBA/DCX.tif]

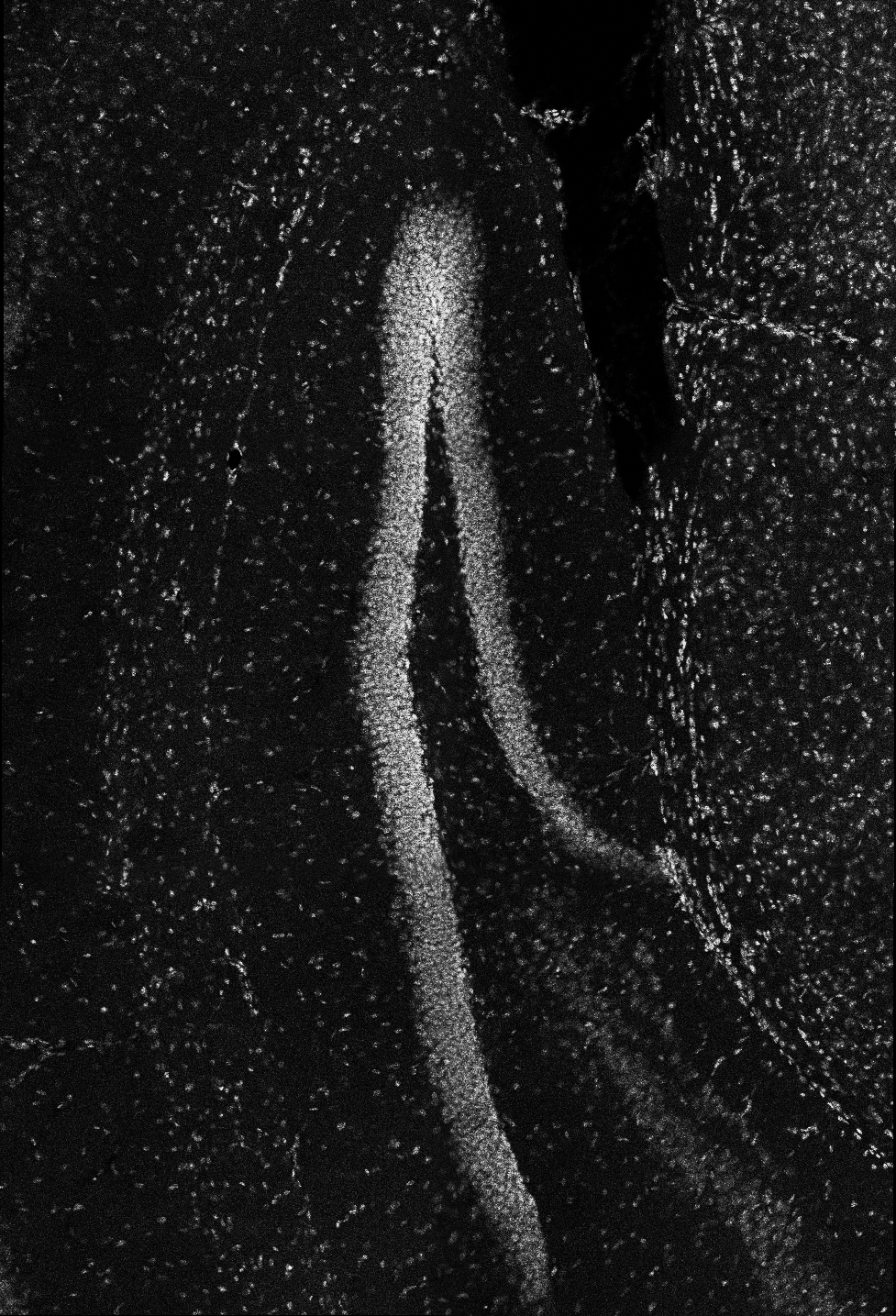

Supplement: Supplementary file 8 — Source data Fig. 6 [file 44319_2024_205_MOESM8_ESM.zip › Source_data_Figure6/6C/Derl1NesCre + 4-PBA/Hoechst.tif]
