# Supplementary material for: The Derlin-1-Stat5b axis maintains homeostasis of adult hippocampal neurogenesis
Source: EMBO Rep. 2024 Jul 30;25(8):26. doi: 10.1038/s44319-024-00205-7 (PMC11316036; doi:10.1038/s44319-024-00205-7)
Supplement: Supplementary file 9 — Expanded View Figures [file 44319_2024_205_MOESM9_ESM.pdf]

Expanded View Figures

Figure EV1. Loss of *Derl1*, but not *Derl2*, specifically promotes NSC activation in the adult DG.

(**A**) Representative immunofluorescence images with Hoechst (gray) and Derlin-1 staining (red) in the adult hippocampus of *Derl1*$^{f/f}$ and *Derl1*^{NesCre} mice. Scale bars: 100 μm. (**B**) GSEA showing differential expression of 238 genes in the DG categorized by the GO term "Response to ER stress." GSEA shows gene expression changes in the DG of *Derl1*^{NesCre} mice relative to *Derl1*$^{f/f}$ mice. The enrichment plot shows the distribution of genes in each set that are positively (red) or negatively (blue) correlated with Derlin-1 deficiency. (**C**) Representative immunofluorescence images with Ki67 (green), Tbr2 (red), and Hoechst (gray) staining of the DG in postnatal day 14 (P14) *Derl1*$^{f/f}$ and *Derl1*^{NesCre} mice. Scale bars: 100 μm. (**D–F**) Quantification of the numbers of Ki67^{+} cells (**D**) and Tbr2^{+} cells (**E**) in the DG as well as the number of Tbr2^{+} cells (**F**) in the molecular layer (ML) of the DG ($n = 3$ mice). (**G**) The percentage of Tbr2^{+} cells in ML among total Tbr2^{+} cells in the DG of P14 *Derl1*$^{f/f}$ and *Derl1*^{NesCre} mice ($n = 3$ mice). (**H**) Experimental scheme for investigating the cell proliferation in the DG of P14 *Derl1*$^{f/f}$ and *Derl1*^{NesCre} mice. (**I**) Representative immunofluorescence images with BrdU (red) and Hoechst (blue) staining of the DG in P14 *Derl1*$^{f/f}$ and *Derl1*^{NesCre} mice. Scale bars: 100 μm. (**J**) Quantification of the numbers of BrdU^{+} cells in the DG of P14 *Derl1*$^{f/f}$ and *Derl1*^{NesCre} mice ($n = 3$ mice). (**K**) Comparison of P14 *Derl1*$^{f/f}$ and *Derl1*^{NesCre} mice brain weights ($n = 3$ mice). (**L**) Representative immunofluorescence images with NeuN (green) and Hoechst (gray) staining of the cerebral cortex in P14 *Derl1*$^{f/f}$ and *Derl1*^{NesCre} mice. Scale bars: 100 μm. (**M**) Experimental scheme for investigating the proliferation of NS/PCs and neurogenesis in *Derl2*$^{f/f}$, *Derl2*^{NesCre}, *Derl1*$^{CaMKIIaCre(hetero)}$ (Control), and *Derl1*^{CaMKIIaCre} mice. (**N**) GSEA showing differential expression of 238 genes in the DG categorized by the GO term "Response to ER stress." GSEA shows gene expression changes in the DG of *Derl2*^{NesCre} mice relative to *Derl2*$^{f/f}$ mice. The enrichment plot shows the distribution of genes in each set that are positively (red) or negatively (blue) correlated with Derlin-2 deficiency. (**O–T**) Representative immunofluorescence images of the DG with BrdU (red), DCX (cyan), and Hoechst staining (gray; insets) (**O**, **R**) and quantification of BrdU^{+} proliferating cells (**P**, **S**) or BrdU^{+} DCX^{+} newborn immature neurons (**Q**, **T**) in mice of each genotype ($n = 3$ mice). Scale bars: 100 μm. (**U**) Representative immunofluorescence images with GFAP (green), S100β (red), Iba1 (cyan), and Hoechst (gray; insets) staining of the DG in 4-week-old *Derl1*$^{f/f}$ and *Derl1*^{NesCre} mice. Scale bars: 100 μm. (**V–Y**) Quantification of the numbers of GFAP^{+} S100β^{+} astrocytes (**V**, **X**) and Iba1^{+} microglia (**W**, **Y**) in the ML (**V**, **W**) or hilus (**X**, **Y**) of 4-week-old *Derl1*$^{f/f}$ and *Derl1*^{NesCre} mice ($n = 3$ mice). (**Z**) Experimental scheme for assessing astrogenesis in the DG of *Derl1*$^{f/f}$ and *Derl1*^{NesCre} mice. (**AA**) Representative immunofluorescence images of the DG with BrdU (red), S100β (green), and Hoechst staining (gray; insets). The areas outlined by a white rectangle are enlarged to the right. Scale bars, 100 μm (left images) and 20 μm (right images). The white arrows indicate merged cells. (**AB**) Quantification of the number of BrdU^{+} S100β^{+} newborn astrocytes in the SGZ and GCL of *Derl1*$^{f/f}$ and *Derl1*^{NesCre} mice ($n = 3$ mice). (**AC**) Representative immunofluorescence images of the DG with active caspase3 (cyan), S100β (green), and Hoechst staining (gray; insets). The areas outlined by a white rectangle are enlarged to the right. Scale bars, 100 μm (left images) and 20 μm (right images). The white arrows indicate merged cells. (**AD–AE**) Quantification of the number of active caspase3^{+} S100β^{+} dying astrocytes in the SGZ (AD) and GCL (AE) of *Derl1*$^{f/f}$ and *Derl1*^{NesCre} mice ($n = 3$ mice). (**AF**) Representative images of Nestin (green), GFAP (red), Sox2 (cyan), and Hoechst (gray; insets) staining of the DG in 2-month-old wild-type mice. (**AG**) Quantification of the number of radial Nestin^{+} Sox2^{+} and radial GFAP^{+} Sox2^{+} cells in the SGZ ($n = 3$ mice). Bar graphs are presented as the mean ± SEM. Significance was determined using Student's *t*-test (**D–G**, **J**, **K**, **P**, **Q**, **S**, **T**, **V**, **W**, **X**, **Y**, **AB**, **AD**, **AE**, **AG**). $^{*}P < 0.05$ and $^{**}P < 0.01$ determined by nominal GSEA *P* value (**B**, **N**). n.s. not significant.

© The Author(s)

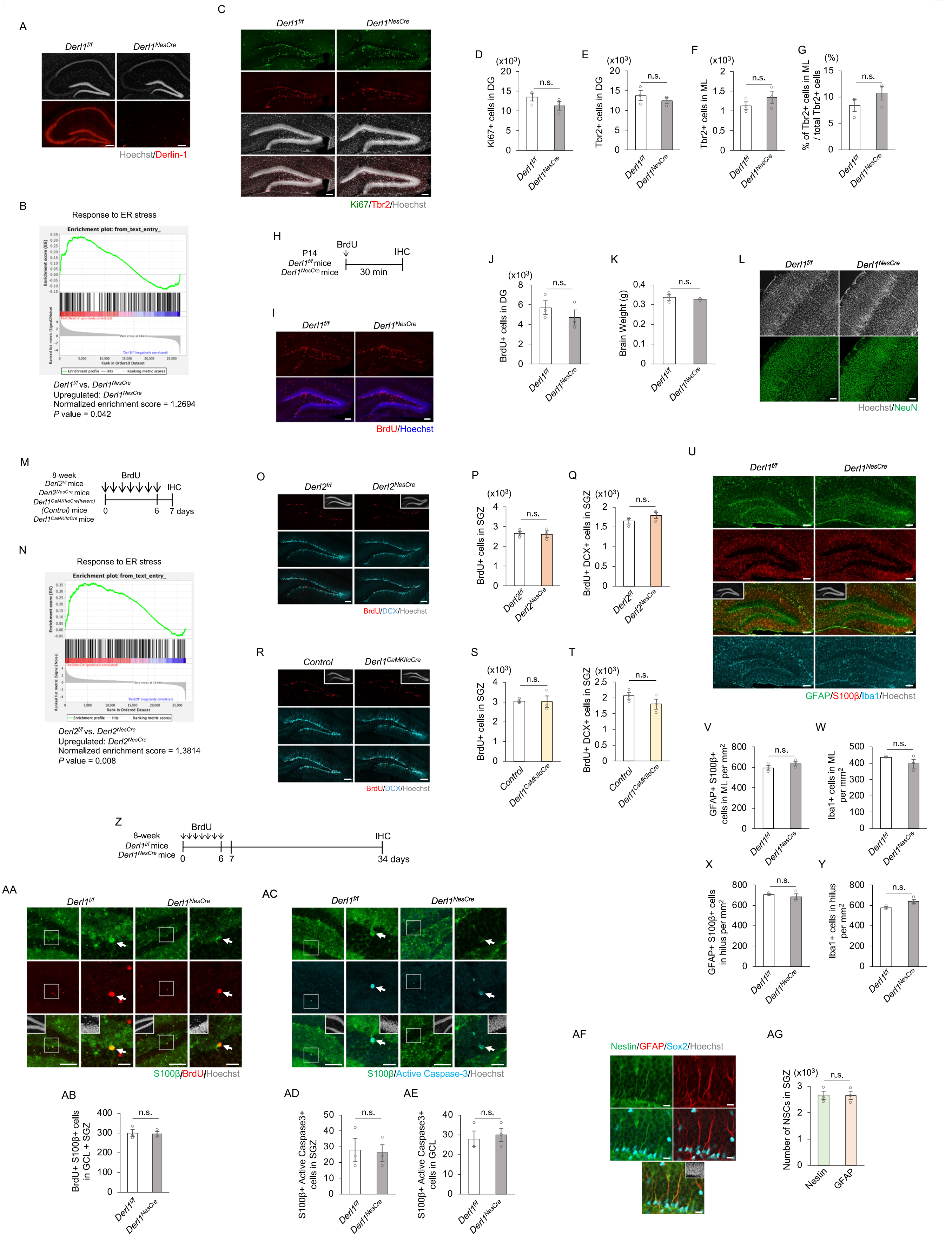

A
Derl1f/f
Derl1NesCre
Hoechst/Derlin-1
B
Response to ER stress
Enrichment plot: from_text_entry_
Derl1f/f vs. Derl1NesCre
Upregulated: Derl1NesCre
Normalized enrichment score = 1.2694
P value = 0.042
C
Ki67/Tbr2/Hoechst
D
Ki67+ cells in DG
E
Tbr2+ cells in DG
F
Tbr2+ cells in ML
G
% of Tbr2+ cells in ML / total Tbr2+ cells
n.s.
H
P14
Derl1f/f mice
Derl1NesCre mice
BrdU
IHC
30 min
I
BrdU/Hoechst
J
BrdU+ cells in DG
K
Brain Weight (g)
L
Hoechst/NeuN
M
8-week
Derl2f/f mice
Derl2NesCre mice
Derl1CaMKIIαCre(hetero) (Control) mice
Derl1CaMKIIαCre mice
7 days
N
Response to ER stress
Derl2f/f vs. Derl2NesCre
Upregulated: Derl2NesCre
Normalized enrichment score = 1.3814
P value = 0.008
O
Derl2f/f
Derl2NesCre
BrdU/DCX/Hoechst
P
BrdU+ cells in SGZ
Q
BrdU+ DCX+ cells in SGZ
R
Control
Derl1CaMKIIαCre
BrdU/DCX/Hoechst
S
T
U
GFAP/S100β/Iba1/Hoechst
V
GFAP+ S100β+ cells in ML per mm2
W
Iba1+ cells in ML per mm2
X
GFAP+ S100β+ cells in hilus per mm2
Y
Iba1+ cells in hilus per mm2
Z
8-week
Derl1f/f mice
Derl1NesCre mice
34 days
AA
S100β/BrdU/Hoechst
AB
BrdU+ S100β+ cells in GCL + SGZ
AC
S100β/Active Caspase-3/Hoechst
AD
S100β+ Active Caspase3+ cells in SGZ
AE
S100β+ Active Caspase3+ cells in GCL
AF
Nestin/GFAP/Sox2/Hoechst
AG
Number of NSCs in SGZ
Nestin
GFAP

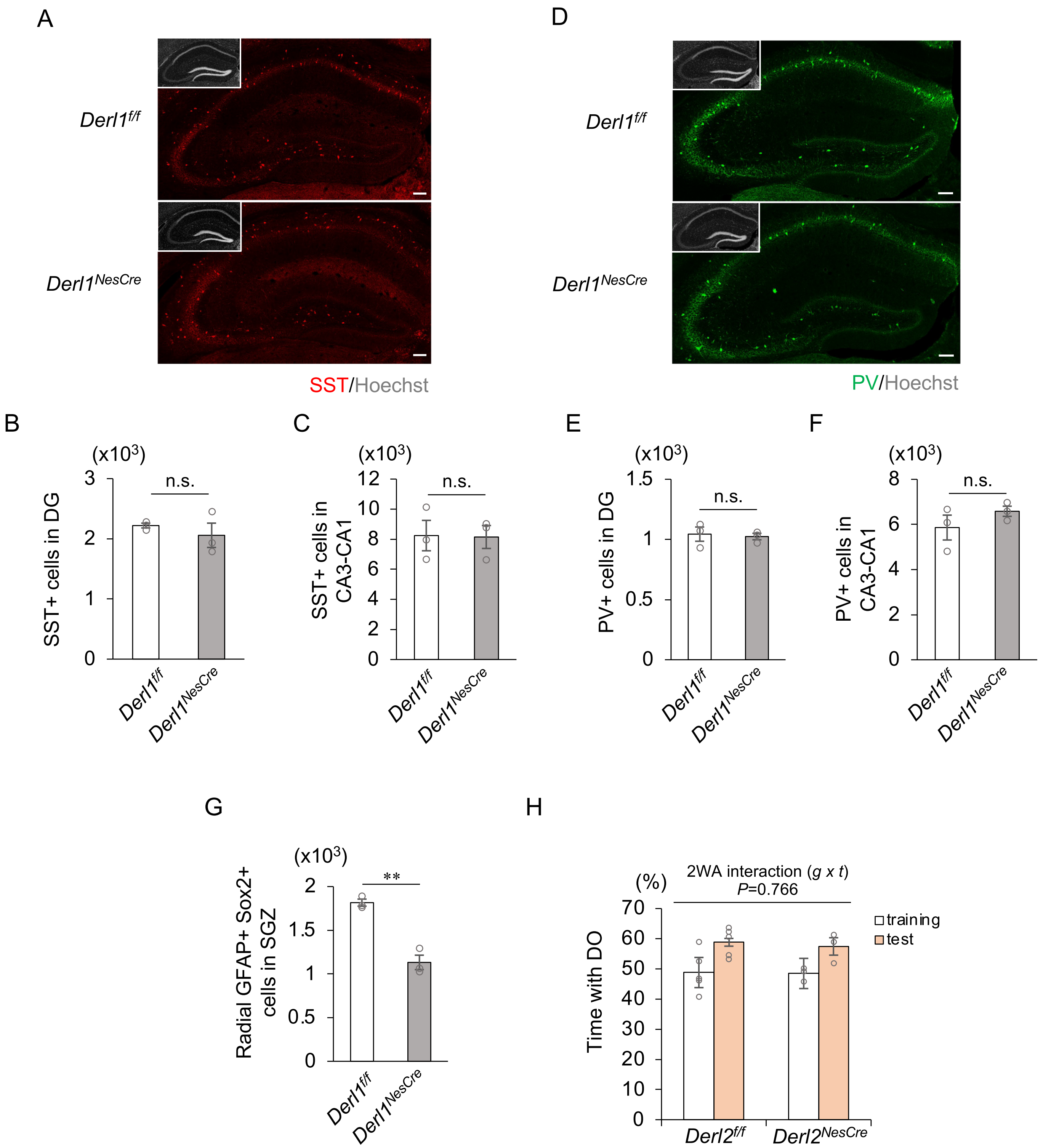
A
Derl1f/f
Derl1NesCre
SST/Hoechst
D
Derl1f/f
Derl1NesCre
PV/Hoechst
B
(x10³)
SST+ cells in DG
n.s.
3
2
1
0
Derl1f/f
Derl1NesCre
C
(x10³)
SST+ cells in CA3-CA1
n.s.
12
10
8
6
4
2
0
Derl1f/f
Derl1NesCre
E
(x10³)
PV+ cells in DG
n.s.
1.5
1
0.5
0
Derl1f/f
Derl1NesCre
F
(x10³)
PV+ cells in CA3-CA1
n.s.
8
6
4
2
0
Derl1f/f
Derl1NesCre
G
(x10³)
Radial GFAP+ Sox2+ cells in SGZ
**
2
1.5
1
0.5
0
Derl1f/f
Derl1NesCre
H
(%)
2WA interaction (g x t)
P=0.766
Time with DO
70
60
50
40
30
20
10
0
training
test
Derl2f/f
Derl2NesCre

Figure EV2. Loss of *Derl1* does not alter the number of GABAergic interneurons in the hippocampus.

(**A**) Representative immunofluorescence images of the hippocampus with somatostatin (SST) (red) and Hoechst staining (gray; insets). Scale bars: 100 μm. (**B**, **C**) Quantification of the number of SST^+ cells in the DG (**B**) and CA3-CA1 (**C**) regions of 2-month-old *Derl1*$^{f/f}$ and *Derl1*^{NesCre} mice ($n = 3$ mice). (**D**) Representative immunofluorescence images of the hippocampus with parvalbumin (PV) (green) and Hoechst staining (gray; insets). Scale bars: 100 μm. (**E**, **F**) Quantification of the number of PV^+ cells in the DG (**E**) and CA3-CA1 (**F**) regions of 2-month-old *Derl1*$^{f/f}$ and *Derl1*^{NesCre} mice ($n = 3$ mice). (**G**) Quantification of the number of radial $GFAP^+$ $Sox2^+$ NSCs in the SGZ of 4-month-old *Derl1*$^{f/f}$ and *Derl1*^{NesCre} mice ($n = 3$ mice). (**H**) Percentage of time spent with the displaced object (DO) during the training and testing phase in 4-month-old *Derl2*$^{f/f}$ and *Derl2*^{NesCre} mice ($n = 5$; *Derl2*$^{f/f}$ mice, $n = 3$; *Derl2*^{NesCre} mice). 2WA two-way ANOVA, g genotype, t trial. Bar graphs are presented as the mean ± SEM. Significance was determined using Student's *t*-test (**B**, **C**, **E**–**G**) or two-way ANOVA (**H**). $^{**}P < 0.01$ by Student's *t*-test (**G**). n.s. not significant.

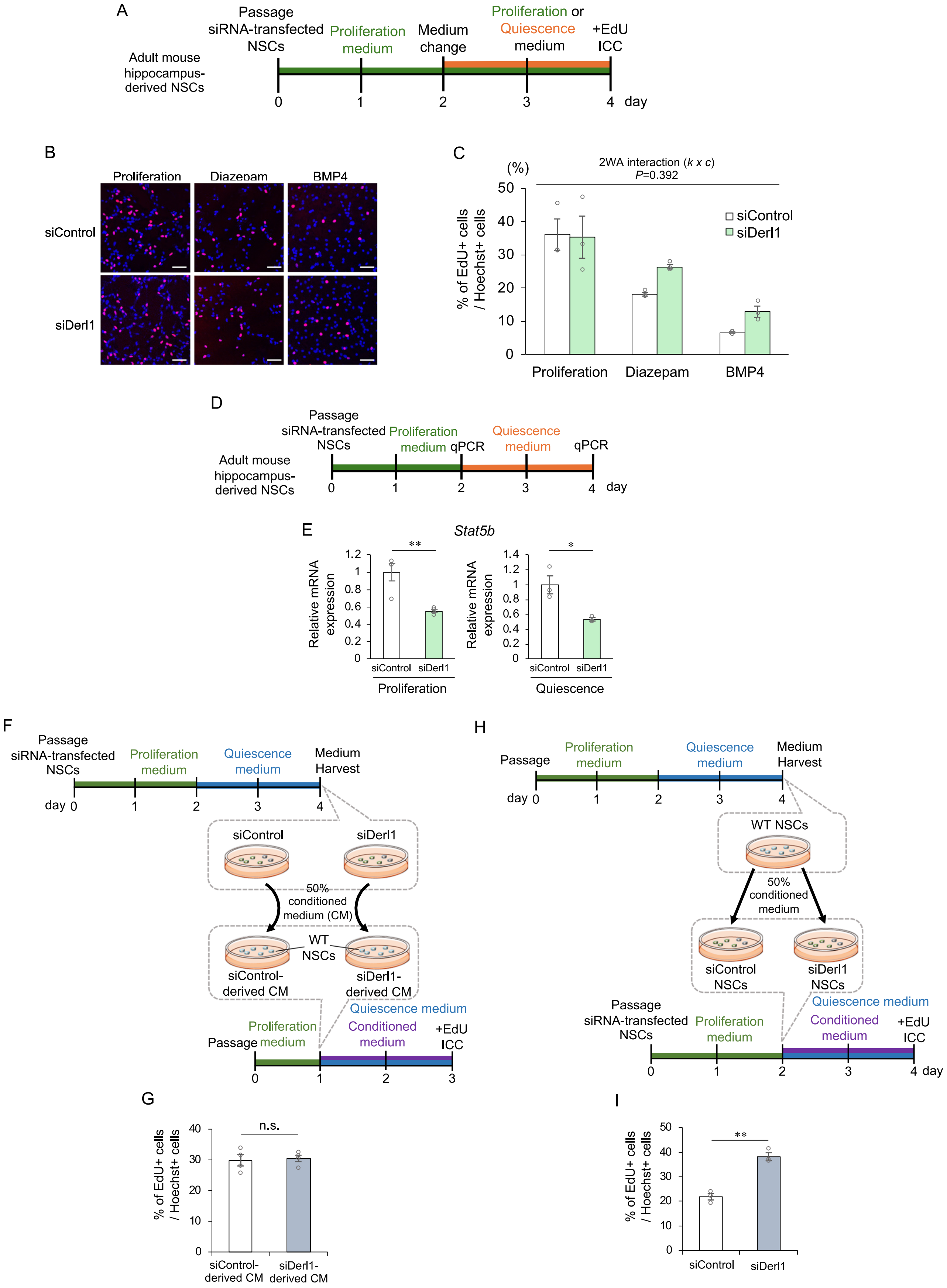
A
Adult mouse hippocampus-derived NSCs
Passage siRNA-transfected NSCs
Proliferation medium
Medium change
Proliferation or Quiescence medium
+EdU ICC
0 1 2 3 4 day
B
Proliferation
Diazepam
BMP4
siControl
siDerl1
C
2WA interaction (k x c) P=0.392
(%)
% of EdU+ cells / Hoechst+ cells
siControl
siDerl1
Proliferation
Diazepam
BMP4
D
Adult mouse hippocampus-derived NSCs
Passage siRNA-transfected NSCs
Proliferation medium
qPCR
Quiescence medium
qPCR
0 1 2 3 4 day
E
Stat5b
Relative mRNA expression
siControl siDerl1
Proliferation
Quiescence
F
Passage siRNA-transfected NSCs
Proliferation medium
Quiescence medium
Medium Harvest
siControl
siDerl1
50% conditioned medium (CM)
WT NSCs
siControl-derived CM
siDerl1-derived CM
Passage
Proliferation medium
Quiescence medium
Conditioned medium
+EdU ICC
G
n.s.
% of EdU+ cells / Hoechst+ cells
siControl-derived CM
siDerl1-derived CM
H
Passage
Proliferation medium
Quiescence medium
Medium Harvest
WT NSCs
50% conditioned medium
siControl NSCs
siDerl1 NSCs
Passage siRNA-transfected NSCs
Proliferation medium
Quiescence medium
Conditioned medium
+EdU ICC
0 1 2 3 4 day
I
% of EdU+ cells / Hoechst+ cells
siControl
siDerl1

 © The Author(s)

Figure EV3. Inhibition of the transition from active to quiescent states in Derlin-1-deficient NSCs is cell-autonomously regulated.

(**A**) Experimental scheme to induce the transition of control and *Derl1* knockdown mouse hippocampal NSCs from active to quiescent states. (**B**) Representative images of EdU (red) and Hoechst (blue) staining in siControl and siDerl1 mouse NSCs with or without induction of quiescence with diazepam (100 μM) or BMP4 (50 ng/mL) for 2 days. NSCs were fixed 30 min after the addition of EdU. Scale bars: 50 μm. (**C**) Quantification of the percentage of EdU^+ proliferating NSCs among total $Hoechst^+$ cells in siControl and siDerl1 mouse NSCs with proliferative conditions or induction of quiescence with diazepam or BMP4 for 2 days ($n = 3$ biological replicates). 2WA two-way ANOVA, k knockdown, c condition. (**D**) Experimental scheme for investigating the *Stat5b* expression of control and *Derl1* knockdown mouse NSCs from active to quiescent states. (**E**) Expression of *Stat5b* in siControl and siDerl1 mouse NSCs under proliferation and quiescent conditions. Gene expression levels were estimated by qPCR and normalized to that of *S18* ($n = 4$ biological replicates; Proliferation, $n = 3$ biological replicates; Quiescence). (**F**) Experimental scheme to investigate NSC proliferation with conditioned medium derived from control and *Derl1* knockdown NSCs over 2 days. (**G**) Quantification of the percentage of EdU^+ proliferating NSCs among total $Hoechst^+$ cells cultured for 2 days in siControl and siDerl1 NSC–derived conditioned quiescence medium ($n = 4$ biological replicates). (**H**) Experimental scheme to investigate NSC proliferation of control and *Derl1* knockdown NSCs with conditioned medium derived from wild-type (WT) NSCs over 2 days. (**I**) Quantification of the percentage of EdU^+ proliferating NSCs among total $Hoechst^+$ cells cultured for 2 days in siControl and siDerl1 NSCs with WT NSC–derived conditioned quiescence medium ($n = 3$ biological replicates). Bar graphs are presented as the mean ± SEM. Significance was determined using Student's *t*-test (**E**, **G**, **I**) or two-way ANOVA (**C**). $^*P < 0.05$ and $^{**}P < 0.01$ by Student's *t*-test (**E**, **I**). n.s. not significant.

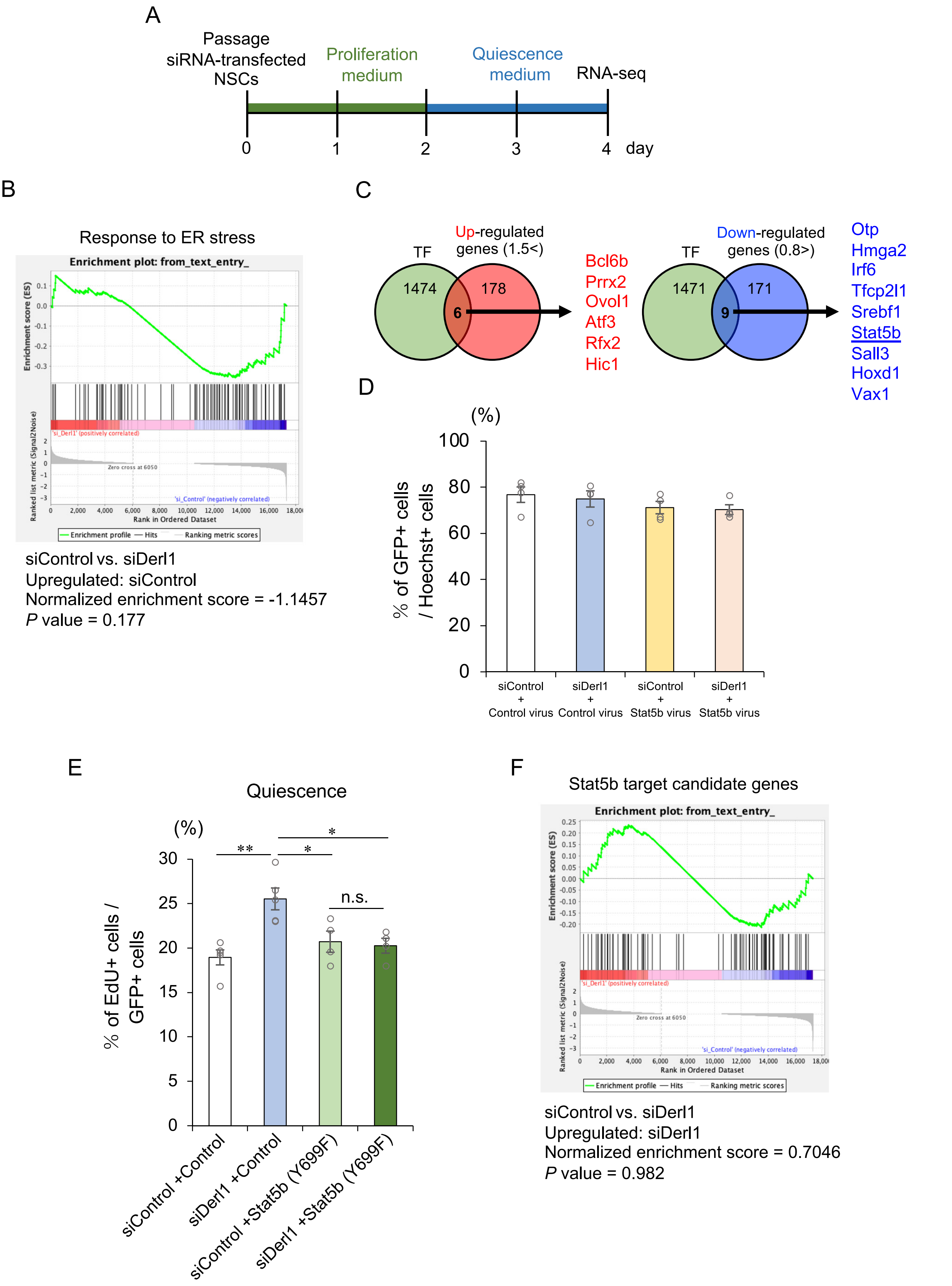
A
Passage
siRNA-transfected
NSCs
Proliferation
medium
Quiescence
medium
RNA-seq
0
1
2
3
4
day
B
Response to ER stress
Enrichment plot: from_text_entry_
Enrichment score (ES)
Ranked list metric (Signal2Noise)
'si_Derl1' (positively correlated)
Zero cross at 6050
'si_Control' (negatively correlated)
Rank in Ordered Dataset
Enrichment profile
Hits
Ranking metric scores
siControl vs. siDerl1
Upregulated: siControl
Normalized enrichment score = -1.1457
P value = 0.177
C
TF
Up-regulated
genes (1.5<)
1474
178
6
Bcl6b
Prrx2
Ovol1
Atf3
Rfx2
Hic1
TF
Down-regulated
genes (0.8>)
1471
171
9
Otp
Hmga2
Irf6
Tfcp2l1
Srebf1
Stat5b
Sall3
Hoxd1
Vax1
D
(%)
% of GFP+ cells
/ Hoechst+ cells
100
80
60
40
20
0
siControl
+
Control virus
siDerl1
+
Control virus
siControl
+
Stat5b virus
siDerl1
+
Stat5b virus
E
Quiescence
(%)
% of EdU+ cells /
GFP+ cells
30
25
20
15
10
5
0
**
*
*
n.s.
siControl +Control
siDerl1 +Control
siControl +Stat5b (Y699F)
siDerl1 +Stat5b (Y699F)
F
Stat5b target candidate genes
Enrichment plot: from_text_entry_
Enrichment score (ES)
Ranked list metric (Signal2Noise)
'si_Derl1' (positively correlated)
Zero cross at 6050
'si_Control' (negatively correlated)
Rank in Ordered Dataset
Enrichment profile
Hits
Ranking metric scores
siControl vs. siDerl1
Upregulated: siDerl1
Normalized enrichment score = 0.7046
P value = 0.982

 © The Author(s)

Figure EV4. Stat5b expression is decreased in Derlin-1-deficient NSCs, and the phosphorylation of Stat5b (Y699) is not required for the rescue of abnormal proliferation of Derlin-1-deficient NSCs.

(**A**) Experimental scheme for investigating the molecular mechanism underlying the impairment of NSC transition to quiescence by *Derl1* knockdown. (**B**) GSEA showing differential expression of 92 genes in the NSCs categorized by the GO term "Response to ER stress." GSEA shows gene expression changes in siDerl1 NSCs relative to siControl NSCs. The enrichment plot shows the distribution of genes in each set that are positively (red) or negatively (blue) correlated with *Derl1* knockdown. (**C**) Venn diagrams showing the overlap between transcription factor (TF) genes and upregulated (left) or downregulated (right) genes in siDerl1 NSCs. (**D**) Quantification of the efficiency of each viral infection in siControl and siDerl1 NSCs ($n = 4$ biological replicates). (**E**) Quantification of the percentage of EdU^{+} proliferating NSCs among total GFP^{+} cells in siControl and siDerl1 NSCs with or without exogenous expression of mutant Stat5b (Y699F) [$n = 5$ biological replicates; + Control, $n = 4$ biological replicates; + Stat5b (Y699F)]. (**F**) GSEA showing differential expression of 80 candidate Stat5b target genes. GSEA shows gene expression changes in siDerl1 NSCs relative to siControl NSCs. The enrichment plot shows the distribution of genes in each set that are positively (red) or negatively (blue) correlated with *Derl1* knockdown. Bar graphs are presented as the mean ± SEM. Significance was determined using the nominal *P* value of GSEA software (**B**, **F**) or one-way ANOVA (**E**). $*P < 0.05$ and $**P < 0.01$ by one-way ANOVA followed by Bonferroni's post hoc test (**E**). n.s. not significant.

© The Author(s)

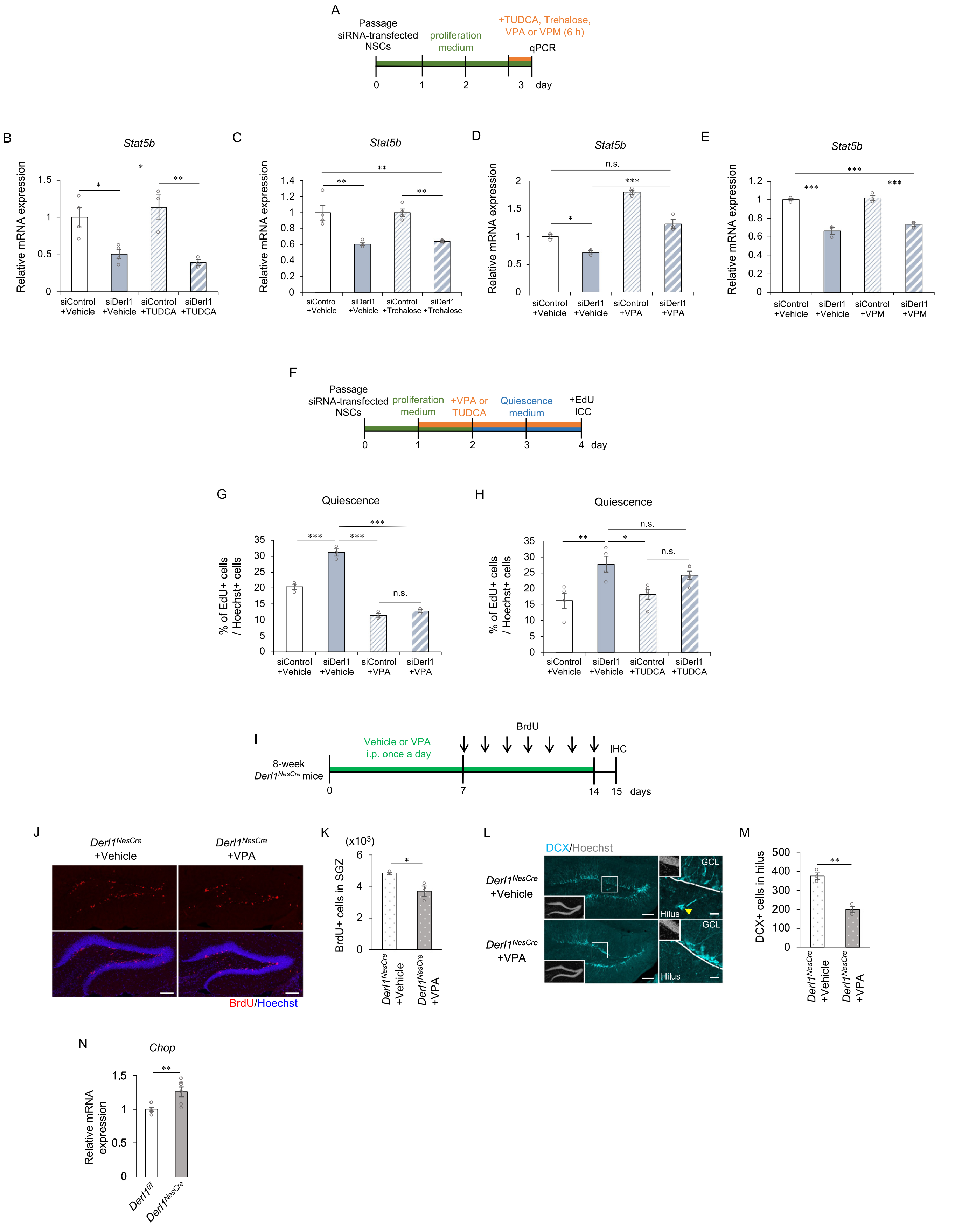
A
Passage siRNA-transfected NSCs
proliferation medium
+TUDCA, Trehalose, VPA or VPM (6 h)
qPCR
0 1 2 3 day
B Stat5b
C Stat5b
D Stat5b
E Stat5b
Relative mRNA expression
siControl +Vehicle
siDerl1 +Vehicle
siControl +TUDCA
siDerl1 +TUDCA
siControl +Trehalose
siDerl1 +Trehalose
siControl +VPA
siDerl1 +VPA
siControl +VPM
siDerl1 +VPM
n.s.
F
Passage siRNA-transfected NSCs
proliferation medium
+VPA or TUDCA
Quiescence medium
+EdU ICC
0 1 2 3 4 day
G Quiescence
H Quiescence
% of EdU+ cells / Hoechst+ cells
I
8-week Derl1NesCre mice
Vehicle or VPA i.p. once a day
BrdU
IHC
0 7 14 15 days
J
Derl1NesCre +Vehicle
Derl1NesCre +VPA
BrdU/Hoechst
K
(x10^3)
BrdU+ cells in SGZ
L
DCX/Hoechst
GCL
Hilus
M
DCX+ cells in hilus
N Chop
Relative mRNA expression
Derl1f/f
Derl1NesCre

 © The Author(s)

Figure EV5. HDAC inhibitory activity, but not chaperone activity, increases *Stat5b* expression and inhibits the proliferation of NSCs.

(**A**) Experimental scheme for assessing the expression of *Stat5b* in siControl and siDerl1 NSCs treated with or without TUDCA (50 μM), trehalose (10 mM), VPA (1 mM), or VPM (1 mM). (**B–E**) Expression of *Stat5b* in siControl and siDerl1 NSCs with or without TUDCA (**B**) ($n = 4$ biological replicates; Vehicle, $n = 3$ biological replicates; TUDCA), trehalose (**C**) ($n = 4$ biological replicates), VPA (**D**) ($n = 3$ biological replicates), or VPM (**E**) treatment ($n = 3$ biological replicates). Gene expression levels were estimated by qPCR and normalized to that of *β-actin*. (**F**) Experimental scheme for evaluating the effect of VPA (1 mM) or TUDCA (50 μM) on the impairment of the transition of NSCs to quiescence by *Derl1* knockdown. (**G**, **H**) Quantification of the percentage of EdU^+ proliferating NSCs among total $Hoechst^+$ cells in VPA-treated (**G**) ($n = 3$ biological replicates) or TUDCA-treated (**H**) ($n = 4$ biological replicates; Vehicle, $n = 5$ biological replicates; TUDCA) siControl and siDerl1 NSCs induced to enter the quiescent state by the administration of BMP4 for 2 days. (**I**) Experimental scheme for investigating the proliferation of NS/PCs in $Derl1^{NesCre}$ mice with or without VPA treatment. $Derl1^{NesCre}$ mice treated with vehicle or VPA daily for 2 weeks were simultaneously injected with BrdU daily for 7 days during the latter and fixed 1 day after the last BrdU injection. (**J**) Representative immunofluorescence images of the DG stained for BrdU (red) and Hoechst (blue) in $Derl1^{NesCre}$ mice treated with or without VPA. Scale bars: 100 μm. (**K**) Quantification of the number of $BrdU^+$ proliferating cells in the SGZ of $Derl1^{NesCre}$ mice treated with or without VPA ($n = 3$ mice). (**L**) Representative immunofluorescence images of the DG stained for DCX (cyan) and Hoechst (gray; insets) in $Derl1^{NesCre}$ mice treated with or without VPA. The areas outlined by a white rectangle are enlarged to the right. The yellow arrowhead indicates DCX^+ ectopic immature neurons in the hilus, and dashed white lines indicate the boundaries between the GCL and hilus. Scale bars, 100 μm (left images) and 20 μm (right images). (**M**) Quantification of the number of DCX^+ cells in the hilus in $Derl1^{NesCre}$ mice treated with or without VPA ($n = 3$ mice). (**N**) Expression of *Chop* in the DG of 2-month-old $Derl1^{f/f}$ and $Derl1^{NesCre}$ mice. Gene expression levels were estimated by qPCR and normalized to that of *S18* ($n = 7$; $Derl1^{f/f}$ mice, $n = 6$; $Derl1^{NesCre}$ mice). Bar graphs are presented as the mean ± SEM. *$P < 0.05$, **$P < 0.01$, and ***$P < 0.001$ by one-way ANOVA followed by Bonferroni's post hoc test (**B–E**, **G**, **H**) or Student's *t*-test (**K**, **M**, **N**). n.s. not significant.

© The Author(s)
